# Understanding pneumococcal serotype 1 biology through population genomic analysis

**DOI:** 10.1186/s12879-016-1987-z

**Published:** 2016-11-08

**Authors:** Chrispin Chaguza, Jennifer E. Cornick, Simon R. Harris, Cheryl P. Andam, Laura Bricio-Moreno, Marie Yang, Feyruz Yalcin, Sani Ousmane, Shanil Govindpersad, Madikay Senghore, Chinelo Ebruke, Mignon Du Plessis, Anmol M. Kiran, Gerd Pluschke, Betuel Sigauque, Lesley McGee, Keith P. Klugman, Paul Turner, Jukka Corander, Julian Parkhill, Jean-Marc Collard, Martin Antonio, Anne von Gottberg, Robert S. Heyderman, Neil French, Aras Kadioglu, William P. Hanage, Dean B. Everett, Stephen D. Bentley, for the PAGe Consortium

**Affiliations:** 1 Department of Clinical Infection, Microbiology and Immunology, Institute of Infection and Global Health, University of Liverpool, Liverpool, L69 7BE UK; 2 Malawi-Liverpool-Wellcome Trust Clinical Research Programme, Queen Elizabeth Central Hospital, Blantyre, Malawi; 3 Pathogen Genomics, Wellcome Trust Sanger Institute, Wellcome Trust Genome Campus, Cambridge, CB10 1SA UK; 4 Department of Epidemiology, Center for Communicable Disease Dynamics, Harvard T. H. Chan School of Public Health, Massachusetts, MA 02115 USA; 5 Unité de Biologie, Centre de Recherche Médicale et Sanitaire (CERMES), Niamey, Niger; 6 National Institute for Communicable Diseases (NICD), Johannesburg, South Africa; 7 Bacterial Diseases Programme, Medical Research Council (MRC), Banjul, The Gambia; 8 Division of Translational and Systems Medicine, Warwick Medical School, University of Warwick, Coventry, CV4 7AL UK; 9 Faculty of Infectious and Tropical Diseases, London School of Hygiene and Tropical Medicine, London, WC1E 7HT UK; 10 Swiss Tropical and Public Health Institute, Basel, Switzerland; 11 Centro de Investigação em Saúde da Manhiça, Maputo, Mozambique; 12 Respiratory Diseases Branch, Centers for Disease Control and Prevention, Atlanta, Georgia GA 30329 USA; 13 Hubert Department of Global Health, Rollins School of Public Health, Emory University, Atlanta, GA 30322 USA; 14 Bill and Melinda Gates Foundation, Seattle, WA 98109 USA; 15 Cambodia Oxford Medical Research Unit, Angkor Hospital for Children, Siem Reap, Cambodia; 16 Centre for Tropical Medicine and Global Health, Nuffield Department of Medicine, University of Oxford, Oxford, OX3 7FZ UK; 17 Department of Mathematics and Statistics, University of Helsinki, Helsinki, Finland; 18 School of Pathology, University of the Witwatersrand, Johannesburg, South Africa; 19 Division of Infection and Immunity, University College London, London, WC1E 6BT UK; 20 Pneumococcal African Genomics (PAGe) Consortium, http://www.pagegenomes.org/

**Keywords:** Pneumococcal serotype 1, ST217, Phylogeography, Evolution, Antibiotic resistance

## Abstract

**Background:**

Pneumococcus kills over one million children annually and over 90 % of these deaths occur in low-income countries especially in Sub-Saharan Africa (SSA) where HIV exacerbates the disease burden. In SSA, serotype 1 pneumococci particularly the endemic ST217 clone, causes majority of the pneumococcal disease burden. To understand the evolution of the virulent ST217 clone, we analysed ST217 whole genomes from isolates sampled from African and Asian countries.

**Methods:**

We analysed 226 whole genome sequences from the ST217 lineage sampled from 9 African and 4 Asian countries. We constructed a whole genome alignment and used it for phylogenetic and coalescent analyses. We also screened the genomes to determine presence of antibiotic resistance conferring genes.

**Results:**

Population structure analysis grouped the ST217 isolates into five sequence clusters (SCs), which were highly associated with different geographical regions and showed limited intracontinental and intercontinental spread. The SCs showed lower than expected genomic sequence, which suggested strong purifying selection and small population sizes caused by bottlenecks. Recombination rates varied between the SCs but were lower than in other successful clones such as PMEN1. African isolates showed higher prevalence of antibiotic resistance genes than Asian isolates. Interestingly, certain West African isolates harbored a defective chloramphenicol and tetracycline resistance-conferring element (Tn*5253*) with a deletion in the loci encoding the chloramphenicol resistance gene (*cat*_pC194_), which caused lower chloramphenicol than tetracycline resistance. Furthermore, certain genes that promote colonisation were absent in the isolates, which may contribute to serotype 1’s rarity in carriage and consequently its lower recombination rates.

**Conclusions:**

The high phylogeographic diversity of the ST217 clone shows that this clone has been in circulation globally for a long time, which allowed its diversification and adaptation in different geographical regions. Such geographic adaptation reflects local variations in selection pressures in different locales. Further studies will be required to fully understand the biological mechanisms which makes the ST217 clone highly invasive but unable to successfully colonise the human nasopharynx for long durations which results in lower recombination rates.

**Electronic supplementary material:**

The online version of this article (doi:10.1186/s12879-016-1987-z) contains supplementary material, which is available to authorized users.

## Background

*Streptococcus pneumoniae*, (or ‘the pneumococcus’), is a Gram-positive, α-hemolytic bacterium which is a cause of significant disease morbidity and mortality worldwide [1]. Invasive pneumococcal diseases (IPD) causes ~1 million deaths in children less than 5 years old every year [1] with the highest IPD burden i.e. ~90 % of the total global death toll, occurring in low-income countries such as Sub-Saharan Africa, Latin America and Asia [1, 2].

Nearly 100 pneumococcal serotypes have been reported to date [3–9], which vary geographically in both prevalence and distribution [2] and in their propensity to cause IPD [10]. Serotype 1 ranks as one of the most prevalent serotypes that cause IPD globally although it is rarely isolated from nasopharyngeal carriage in humans [10, 11], which results in high invasive potential with odds ratio of ~10 for causing IPD relative to carriage [10]. Longitudinal studies have reported that serotype 1 is carried for a period of ~9 days, which is the second shortest human carriage duration reported to date after serotype 33B [12]. Pneumococcal isolates undergo genetic recombination where by competent isolates acquire and incorporate external DNA into their chromosomes. This occurs more efficiently during carriage than invasive disease [13]. The rarity of serotype 1 isolates in carriage has led to the hypothesis it has a lower rate of recombination due to limited opportunities for genetic exchange during carriage [14]. A recent report has identified the presence of genetic recombination in a global serotype 1 population [15].

Multilocus sequence typing (MLST) [16] and phylogenetic analysis of a global collection of serotype 1 isolates, showed that serotype 1 isolates cluster into three distinct clades predominantly associated with continent [17]. European and American isolates clustered into clades A and C respectively, whilst clade B associated with African isolates. Recently, using a whole genome phylogeny, we have reported an additional clade named clade D, which consisted of isolates from Asia [15].

Serotype 1 pneumococci cause a high IPD burden especially in Africa [2] where it is endemic in most countries particularly within the African Meningitis belt [18]. The high burden led to the incorporation of serotype 1 capsular polysaccharides in the pneumococcal conjugate vaccines (PCV); PCV10 [19] and PCV13 [20], which have now been introduced globally. Currently, studies to assess the effectiveness of PCV10 and PCV13 in the African population are underway. In Africa, the majority of the serotype 1 IPD is caused by the ST217 clone (also known as Sweden^1^-27 (PMEN27) as defined by the Pneumococcal Molecular Epidemiology Network) [17]. This lineage accounts for over 95 % of serotype 1 IPD in Malawi and 98 % of such cases in South Africa [21–23] and causes pneumococcal meningitis outbreaks in West Africa [24, 25].

We have previously described the global population structure of multiple serotype 1 STs [15]. In the present study, we collected 226 ST217 serotype 1 isolates from multiple African and Asian countries to gain further insights into the biology of the clone. We used whole genome sequencing to determine the population structure, genomic diversity, geographic spread, population size changes through time and identify key pneumococcal virulence genes associated with the ST217 endemic virulent clone. Our findings provide further insights into the biological mechanisms that may have driven the success of the ST217 clone especially in Africa.

## Methods

### Isolate collection, DNA extraction and sequencing

We collected pneumococcal serotype 1 ST217 isolates from 1994 to 2011. Isolates were obtained from both carriage and invasive disease in individuals of all age groups from hospitals in different countries collaborating in the Pneumococcal African Genomics (PAGe) Consortium (http://www.pagegenomes.org) (Additional file 1). The Global Pneumococcal Strain Bank at the Centers for Disease Control and Prevention (CDC) provided additional isolates from additional nine countries. In total, the study dataset comprised of 226 isolates; invasive (*n* = 206), nasopharynx (*n* = 6) and other body sites or unknown source (*n* = 15), from nine African countries (*n* = 200) and four Asian countries (*n* = 26). Because carriage of serotype 1 pneumococci is extremely rare, the dataset constituted predominantly of invasive isolates. Genomic DNA libraries were prepared from the isolates and sequenced at the Wellcome Trust Sanger Institute using Illumina Genome Analyzer II (Illumina, CA, USA).

### Detection of serotypes and sequence types

We subjected the isolates to molecular serotyping by PCR [26]. The inferred serotypes agreed with in silico identified serotypes using short read mapping against reference capsule biosynthetic locus genes [27]. Sequence types (STs) were determined using the pneumococcal multilocus sequence typing (MLST) scheme [16, 28].

### Recombination detection and phylogeny construction

We obtained consensus sequences for the study isolates through mapping short paired-end sequence reads against a published serotype 1 reference whole genome sequence (Spn1041) [Genbank: CACE00000000] using SMALT v0.7.4 (http://www.sanger.ac.uk/resources/software/smalt/). To realign the insertion and deletion (indel) sites, we used GATK v3.3.0 [29]. From the realigned mapping files, we generated consensus whole genome sequences, which were aligned to generate whole genome alignment. We identified sequence clusters (SCs) for the study isolates using the whole genome sequence alignment generated as described in the previous section. Following this, we generated an alignment of only the polymorphic (variable) sites using Snp-Sites [30]. The hierBAPS module in the BAPS v6.0 s software clustered the isolates into unique subpopulations or SCs [31, 32]. We detected recombination events for each SC whole genome alignment using Gubbins v1.1.1 [33] and inferred the maximum likelihood phylogeny using RAxML v7.0.4 using a generalised time reversible (GTR) model with Gamma heterogeneity among nucleotide sites [34] and 100 bootstrap replicates. Closely related serotype 1 ST615 isolates were included in the alignment and later used to root the phylogeny as an outgroup. The tree visualisations were done using BioPython [35] and iToL v3.2.4 [36]. The sequence reads were also assembled into contigs using an automated sequence assembly pipeline developed at the Wellcome Trust Sanger Institute [37] that uses Velvet v1.2.09 [38], SSPACE Basic v2.0 [39] and BWA v0.7.12-r1039 [40]. The assembled genes were annotated using Prokka v1.11 [41] and core and accessory genome analysis was done using Roary [42].

### Within-sequence cluster genetic diversity

We calculated the pairwise distances between isolates in the phylogeny and the terminal branch distances in the phylogeny using BioPython [35], Phylobase and Phytools packages in R [43]. Additional evolution parameters namely the number of segregating or polymorphic alignment sites (S), proportion of polymorphic sites (P_s_), observed nucleotide sequence diversity (π), expected nucleotide sequence diversity (Θ) and Tajima’s D, which is the scaled difference of π and Θ, were inferred using MEGA v6.0 [44].

### Most recent common ancestors and mutation rates

We used Path-O-Gen v1.4 to investigate the temporal evolution patterns within the inferred SCs (http://tree.bio.ed.ac.uk/software/pathogen/). Coalescent analyses were done using BEAUti v1.7.5 and BEAST v1.8 [45, 46] with the following parameter specifications; lognormal relaxed uncorrelated clock model [47], constant size coalescent tree prior, Hasegawa-Kishino-Yano (HKY85) nucleotide substitution model with estimated base frequencies [48] and a Gamma (Y) site heterogeneity model with 4 rate categories [49] and the prior mutation rate (μ) from a previous study [50]. 200,000,000 Markov Chain Monte Carlo (MCMC) iterations were performed and sampled every 5000 steps with a burn-in of 20,000,000 iterations discarded from each independent MCMC analysis. We resampled the MCMC runs at 20,000 steps using LogCombiner v1.7.5 (http://beast.bio.ed.ac.uk/logcombiner) to estimate the mean values and 95 % highest posterior densities (HPD) for different parameters of interest in Tracer v1.6 (http://beast.bio.ed.ac.uk/tracer) [51]. The relative genetic diversity over time or the effective population size (N_e_τ), was estimated using BEAST with a Bayesian skyline plot (BSP) model with 10 groups and a piecewise constant skyline model variant [52].

### Presence of virulence and antibiotic resistance genes

We determined pneumococcal virulence genes from the published studies [53] and obtained the sequences for each virulence gene from the Virulence Factors Database [54] while antibiotic resistance conferring genes and integrative conjugative elements (ICEs) transposons were obtained from Genbank [55]. We checked the presence and absence of the virulence genes and the resistance elements using nucleotide BLASTN v2.2.30 [56]. To determine the presence of a genomic feature, we considered only the highest scoring pairs (HSPs) from BLAST comparisons with an E-value less than 1E-3. Such HSPs represented significant sequence matches and were joined. We considered the feature present when the combined HSPs covered at least 70 % of the query feature sequence and showed at least 80 % nucleotide identity. We visualised the BLAST comparisons using ACT v13.0.0 [57]. To validate the absence of genomic features, we used mapped short sequence reads against reference features using BWA v0.7.12-r1039 [40].

### Statistical analysis

All tests were performed using R v3.1.2 (R Core Team) and GraphPad Prism v6.0 (www.graphpad.com). We checked conformity of the observations to the standard normal (Gaussian) distribution using the Shapiro-Wilk and Kolmogorov-Smirnov tests and compared differences between multiple groups with small sample sizes using the Kruskal-Wallis test otherwise analysis of variance (ANOVA) and unpaired Student’s t tests were used. We compared differences in proportions using the two-tailed two-sample proportions test with a Yates continuity correction.

## Results

### Phylogenetic clusters and geographic structure

**Fig. 1:**
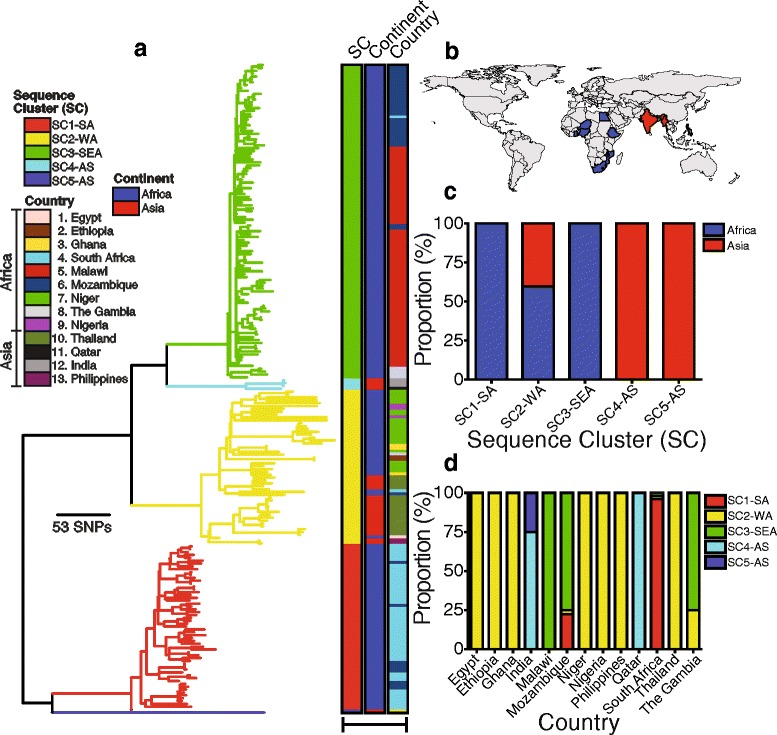
Phylogenetic relationships of the ST217 serotype 1 isolates*.* **a** Maximum likelihood phylogeny showing genetic relationships of the ST217 isolates rooted using isolates from ST615 as an outgroup (not shown). The colored strips after the phylogenetic tips shows the inferred SCs, continent and country of origin. A full interactive phylogeny of the ST217 isolates and associated metadata was uploaded to Microreact webserver and is available here https://microreact.org/project/PMEN27_TREE. **b** Countries from which the ST217 isolates originated. **c** Association between continent of origin of the ST217 isolates with SCs and **d** association between country of origin with SCs

Phylogenetic analysis of the 226 ST217 isolates from Africa (*n* = 200) and Asia (*n* = 26) clearly showed five distinct clades (Fig. 1a and b). We identified the underlying genetic population structure of the isolates by clustering the isolates into genetically distinct subpopulations known as sequence clusters (SC). The identified SCs matched the phylogenetic clades from the phylogeny in Fig. 1 which was constructed from a recombination free alignment. The identified SCs predominantly associated with geographical origin of the isolates and were named to reflect the origin as follows SC1-SA, SC2-WA, SC3-SEA, SC4-AS and SC5-AS. The suffix denotes the origin of the majority isolates in the SC where SA denotes South Africa, WA denotes West Africa, SEA denotes South East Africa and AS denotes Asia. Almost half the samples fell into SC3-SEA (*n* = 110), with SC1-SA (*n* = 58), and SC2-WA (*n* = 53) accounting for the majority of the remaining samples. Both country and continent of origin of the isolates associated with the SCs (*p* < 0.0001). Except for two instances where isolates from South Africa (*n* = 2) and Mozambique (*n* = 1) were identified in the West African clade as intracontinental clonal spread, all the isolates from southern Africa, which together represent the best-sampled region in the analysis, fell into clades SC1-SA and SC3-SEA. Clade SC1-SA predominantly comprised of South African isolates with a few from Mozambique that spread from South Africa, while clade SC3-SEA comprised of isolates from Malawi and Mozambique with a few isolates from South Africa and The Gambia. The West African clade showed highest geographical distribution and consisted of isolates from both Africa and Asia. The remaining two smaller Asian SCs contained few isolates but showed a clear association with Middle East countries and their deep branching suggests ancient divergence maintained by geographic separation.

### Geographical spread of the ST217 clone

The association of some SCs such as the West African clade with multiple continents and the association of some countries with multiple SCs suggested geographical spread of the clone between regions (Fig. 1c and d). Further analysis of the intermixing of the isolates in the SCs and genetic relatedness based on the phylogenetic branch lengths allowed for inference of the potential spread of the ST217 clone between countries and continents. Overall, closely located countries shared closely related but genetically distinguishable isolates with few mutations separating them. In some instances, isolates from one country clustered on a different branch with isolates from a different country. Such patterns allowed for determination of the not only occurrences and where applicable the directionality of the spread of the clone. For example, four instances of spread were identified in the typically South African clade (SC1-SA) whereby isolates spread from Mozambique grouped with South African isolates but not the rest of the Mozambican isolates in clade SC3-SEA, which demonstrates a spread from South Africa to Mozambique where they were eventually sampled (Fig. 1a and Additional file 2a). Although clade SC3-SEA was typically Malawian and Mozambican isolates, isolates from each country formed very tight genetically related but distinct clusters in the SC. In depth analysis of this clade showed that some Mozambican isolates clustered with the Malawian isolates within the clade, which suggest very recent and short-range spread between these closely located countries. We also identified several long-range intracontinental and intercontinental spread of the clone. Spread of isolates from West Africa to Mozambique and South Africa (Additional file 2b) and spread from Malawi to the Gambia in SC3-SEA provides examples of such long-range intracontinental spread events (Additional file 2c). On the other hand, the clustering of Asian isolates with the West African counterparts in clade SC2-WA clearly demonstrates intercontinental spread of the clone.

### Distribution of alleles in sequence clusters

**Fig. 2:**
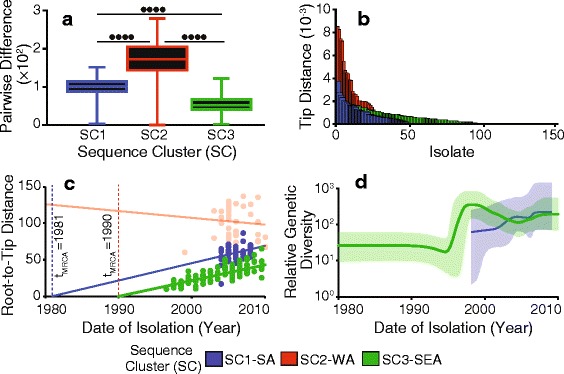
Estimated evolution parameters in the ST217 SCs. Distribution of **a** cophenetic distances between pairs of the ST217 isolates in each SC and **b** Terminal phylogenetic branch lengths for the isolates in the clades. **c** Graph showing relationship between the phylogenetic root-to-tip distances and isolation years of the isolates for each clade: SC1-SA (blue), SC2-SA (light red) and SC3-SEA (green). Isolates in clade SC2-SA showed no evidence of molecular clock-like evolution and was not subjected to coalescent analysis. **d** Bayesian skyline plot showing the temporal effective population sizes (N_e_τ) of clades SC1-SA and SC3-SEA. *P*-values are shown as follows: *p* ≤ 0.05 (•), *p* ≤ 0.01 (••), *p* ≤ 0.001 (•••), *p* ≤ 0.0001 (••••) and *p* > 0.05 (ns)

Differences between the three largest SCs in terms of sequence variation were evident from the branch lengths in Fig. 1. We calculated the cophenetic distances between pairs of isolates in each SC, terminal taxon tip branch lengths in the phylogeny and the distribution of SNPs in each subpopulation to precisely quantify the within and between SC sequence variation and diversity (Fig. 2a-b and Additional file 3). The mean cophenetic distances were significantly different between the three SCs (*p* < 0.0001). Clade SC3-SEA showed the lowest mean cophenetic distance (mean = 97.35) followed by clade SC1-SA (mean = 167.90). On the other hand, clade SC2-WA (mean = 721.1) showed the highest cophenetic distance, which reflects the greater geographical diversity in this SC. Furthermore, clade SC2-WA showed longer terminal branches than SC1-SA (*p* < 0.0001) and SC3-SEA (*p* = 0.003), which suggest higher mutation rates in the West African SC. Although SC1-SA showed slightly longer tip distances than SC3-SEA, no significant differences (*p* = 0.636) were observed. Our findings indicate that the ST217 isolates from West Africa show higher sequence diversity than isolates from Southern African region whereby the observed lower diversity maybe a consequence of recent introduction or potentially small population sizes due to population bottlenecks.

### Temporal evolution of the ST217 sequence clusters

To understand the changes over time in the ST217 SCs, we studied sequence variation over the sampling period (1994 to 2011). Horizontally acquired mutations were removed to study ancestral changes alone [33]. We checked the SCs for molecular clock-like evolution determined as significant linear accumulation of mutations with time. This was done using a linear regression analysis of the phylogenetic root-to-tip distances and the isolation years for the isolates. We observed a significant relationship in clades SC1-SA (*R* ^*2*^ = 0.1176*, p* = 0.0084) and SC3-SEA (*R* ^*2*^ = 0.5530, *p* < 0.0001) but not clade SC2-WA (*R* ^*2*^ = 0.0079*, p* = 0.548) (Fig. 2c). The lack of molecular-clock-like evolution in clade SC2-WA, which showed the highest diversity in terms of both sequence variation and geographical origin and suggests that deeper sampling is required to study this SC.

From the regression of the root-to-tip distance and isolation years, we calculated mutation rates (μ) of 1.12 × 10^-6^ and 1.01 × 10^-6^ nucleotide substitutions per site per year for SC1-SA and SC3-SEA respectively (Fig. 2c). The estimates from BEAST [45, 46] were consistent with the regression analysis and showed mutation rates of 2.05 × 10^-6^ and 4.58 × 10^-6^ nucleotide substitutions per site per year for SC1-SA and SC3-SEA respectively. Such mutation rates suggest introduction of approximately 4 to 10 nucleotide changes per isolate annually. To determine the potential dates of emergence or importation of the SCs, we extrapolated the fitted linear regression backwards to determine the time corresponding to zero phylogenetic root-to-tip distance, which represents the time of divergence of the most recent common ancestor (TMRCA). The TMRCAs for the clades SC1-SA and SC3-SEA dated to 1981 (95 % CI: 1912 to 1992) and 1990 (95 % CI: 1987 to 1993) respectively (Fig. 2c). However, TMRCA estimates from BEAST were slightly different to the extrapolated regression estimates, with 1990 for SC1-SA (95 % highest posterior density (HPD): 1981 to 1998) and 1955 for SC3-SEA (95 % HPD: 1923 to 1979). Nevertheless, both methods unequivocally suggest recent emergence of the SCs with TMRCAs dating back to the last century. In terms of the population sizes and population dynamics, Bayesian skyline plots (BSP) showed that the relative genetic diversity which corresponds to the effective population size (N_e_τ), increased rapidly in the early 1990s for SC3-SEA possibly following its emergence and was higher than in SC1-SA (Fig. 2d). However, the effective population size in SC3-SEA decreased slightly from the mid-2000s but remained relatively stable but slightly lower than in SC1-SA until the last sampling point in 2010 consistent with the findings in Fig. 2a-b.

### Genetic recombination in the ST217 sequence clusters

We observed the distribution of genetic recombination events consistent with previous studies [15, 50, 58]. The mean sizes of the recombination events varied significantly (*p* < 0.0001) between the SCs and the mean sizes ranged from 4650 bp to 75156 bp (Table 1). The ratio of number of single nucleotide polymorphisms (SNPs) imported by recombination to those arising independently in the non-recombining regions (*r/m*) did not vary significantly between the ST217 SCs (*p* = 0.278) (Table 1). SC2-WA showed highest relative rate of recombination (*r/m =* 4.22) followed by SC3-SEA (*r/m* = 1.51) while SC1-SA (*r/m* = 0.05) showed the lowest recombination (Additional files 4, 5 and 6). The mean number of distinct recombination events in each SC per isolate ranged from 0.86 to 2.2 and up to 96 % of the isolates shared some recombination events. Surface proteins, antibiotic resistance and mobile elements were also common in the ‘hotspots’ of recombination as previously reported [15] (Additional files 7, 8 and 9). The findings showed lower recombination rates in the ST217 SCs than other successful pneumococcal clones such as Spain^23F^-1 (PMEN1, *r/m =* 7.2) [50], Spain^6B^-2 (PMEN2, *r/m* = 14.9) [59] and Taiwan^19F^-14 (PMEN14, *r/m* = 21.8) clones [59] but higher than the ST180 clone (*r/m* = 0.07) [60].

**Table 1:** Summary of the identified recombination events in the ST217 isolates in each SC

Clade	N_REC_^a^	Recombination Size (bp)			Recombination to Mutation (*r/m*)			SNPs per Recombination Event		
		Lower 95 % CI	Mean	Upper 95 % CI	Lower 95 % CI	Mean	Upper 95 % CI	Lower 95 % CI	Mean	Upper 95 % CI
SC1-SA	92	1736	4650	7565	0	0.05	0.12	0	24.80	57.67
SC2-WA	86	50111	75156	100201	0	4.22	7.80	102.20	348	593.90
SC3-SEA	61	12	21149	9490	0	1.51	2.72	48.00	176.40	304.70

CI designates confidence intervals

^a^The number of recombination events identified in each clade in designated by N_REC_

### Natural selection in genes

**Fig. 3:**
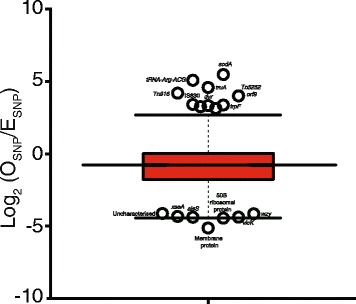
Distribution of the single nucleotide polymorphisms (SNPs) in the genes identified in the ST217 isolates. Boxplot showing the observed (O_SNP_) and expected (E_SNP_) number of SNPs identified in each gene in the ST217 isolates. Genes with log_2_ (O_SNP_/E_SNP_) less than -1 were considered to be under ‘negative selection’, greater than 1 under ‘positive selection’ and under ‘neutral evolution’ for those between -1 and 1

Comparing observed and expected number of SNPs revealed potential dominant selective forces acting on the genomes (Additional files 10). From the genome wide sequence alignment, we estimated a probability of ~0.012 for random occurrence of a single SNP at any position. Using this probability, we calculated the expected occurrences of SNPs for every gene and compared this to the observed number of SNPs form an alignment of each gene. Approximately 15 % of the genes showed higher number of SNPs than expected possibly due to diversifying or balancing selection (Fig. 3). On the other hand, ~44 and ~40 % of the genes showed evidence of purifying selection and neutral evolution. The higher number of genes under purifying selection than diversifying selection suggests that most genomic changes are either highly adaptive and strongly selected for or highly deleterious and negatively selected for. Examples of genes under purifying selection included the capsule biosynthesis genes while phage and transposon elements were under diversifying selection although the later may be driven by independent phage evolution rather than pneumococcal evolution. These findings suggest purifying selection and neutral evolution are dominant forces driving the evolution of the genes in the ST217 clone.

### Colonisation and adherence factors

We also investigated the distribution of key pneumococcal virulence factors in the ST217 clone (Additional file 9). There was near universal presence of the key virulence genes in the ST217 isolates (Additional file 9). These included surface exposed proteins such as autolysins and choline binding proteins (CBPs); competence proteins such as the competence stimulating peptides (CSP), mismatch repair genes, two-component system (TCS) genes and several others were universally present in the isolates. All the isolates lacked the *psrP* gene*,* which encodes an adhesin and facilitate biofilm formation [61], *iga* gene which encodes an immunoglobulin protease that cleaves the human Iga and promote colonisation [62], two zinc metalloproteinase genes namely *zmpB* and *zmpC*, important for colonisation [62] and both pilus operons (type I or II) which promote adherence to the epithelial surfaces [63–65] were also absent in all the study isolates (Additional files 11 and 12). The absence of the well-known adherence and colonisation factors may not be the sole cause but may contribute to the rarity of serotype 1 pneumococci in human nasopharyngeal carriage which would in turn limit its opportunities for recombination.

### Accessory genes and antibiotic resistance conferring elements

**Fig. 4:**
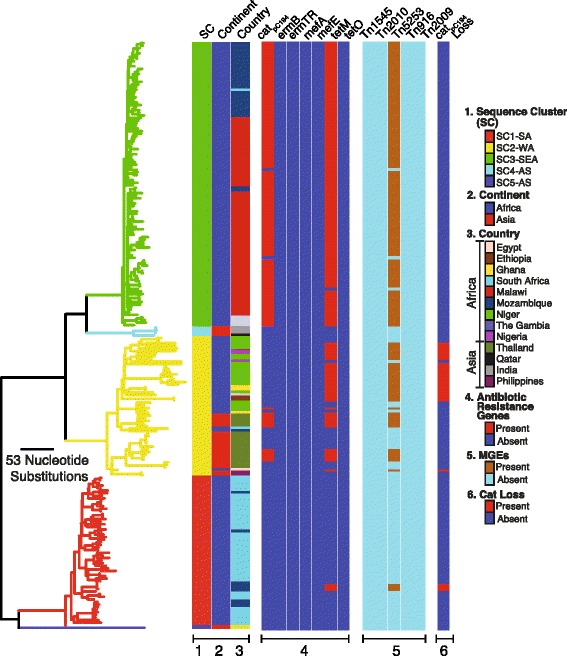
Distribution of antibiotic resistance conferring genes and elements in the ST217 isolates. The columns to the right of the phylogeny show presence and absence of the antibiotic resistance conferring elements and presence of the deletion in the region containing chloramphenicol resistance gene

**Fig. 5:**
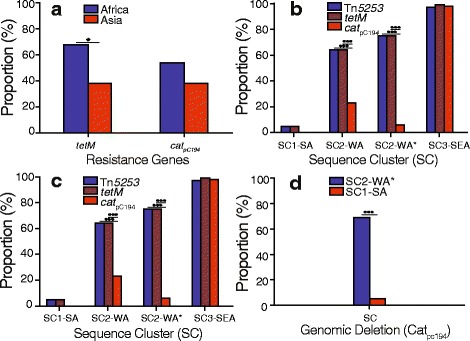
Prevalence of antibiotic resistance conferring genes and mobile genetic elements in the ST217 isolates. Prevalence of tetracycline (*tetM*) and chloramphenicol (*cat*_pC194_) resistance conferring genes in ST217 isolates from different **a** continents and **b** SCs. **c** Prevalence of the tetracycline, chloramphenicol and Tn*5253* element in different SCs. The SC marked with an asterisk shows prevalence of the features among only African isolates in the SC. **d** Prevalence of the Tn*5253* element containing isolates but with a deletion of the chloramphenicol resistance-conferring gene. Differences in prevalence were compared using the two-sample two-tailed proportions test and the *P*-values are labeled as follows: *p* ≤ 0.05 (•) and *p* ≤ 0.0001 (•••)

We identified 1520 core genes present in at least 99 % of the isolates and an overall gene repertoire of 4594 genes. Majority of the accessory genes were shared between isolates in the same SCs (Additional file 13). In terms of antibiotic resistance, the Tn*5253* integrative conjugative element (ICE) harbored both *tetM* and *cat*_pC194_ genes which encodes proteins conferring resistance to tetracycline and chloramphenicol antibiotics (Fig. 4). Furthermore, macrolide resistance conferring ICEs that carry the *ermB* and *mefA/E* genes were absent. Overall, African isolates showed a higher prevalence of the Tn*5253* element than the Asian ST217 isolates (Fig. 5a).

**Fig. 6:**
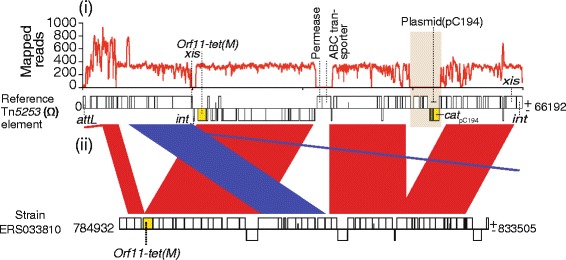
Deletion of the chloramphenicol resistance-conferring gene in the Tn*5253* element in West African isolates. A tetracycline resistant but chloramphenicol susceptible isolate (ERS033810) that harbored a Tn*5253* element was compared to the reference Tn*5253* with an intact *tetM* and *cat*_pC194_ resistance conferring genes. **i** Number of mapped short paired-end sequence reads against the reference Tn*5253* element. **ii** Comparative sequence analysis of the Tn*5253* element from the isolate against the reference Tn*5253* using BLAST

Within the West African clade (SC2-WA) prevalence of *tetM* gene was higher than *cat*_pC194_ gene despite both genes being harbored on only Tn*5253* element in this population (Fig. 5b). We did not observe such a disparity in the Asian isolates of the genetic background in the clade, which showed equal prevalence of both genes. Because no additional ICEs were identified that carried additional tetracycline resistance genes, we checked whether there was either high sequence diversity in the *cat*_pC194_ gene, which would make it difficult to detect it or whether the *cat*_pC194_ gene was completely deleted from the Tn*5253* element in the chloramphenicol susceptible but tetracycline resistant isolates. These isolates harbored a defective Tn*5253* ICE with an intact *tetM* gene but with an ~5Kb deletion across the pC194 plasmid loci that encodes and harbors the *cat*_pC194_ gene (Figs. 4, Fig. 5c). To confirm the deletion of the chloramphenicol resistance gene from Tn*5253* element, we mapped the short sequence reads from isolates containing deleted loci against a reference Tn*5253* sequence with an intact chloramphenicol resistance conferring loci. Consistent with the previously mentioned results, we observed no read mapping across the pC194 plasmid (Fig. 6). Furthermore, we observed no mapping on the phage attachment site (*attL*), excisionase (*xis*) and integrase (*int*) genes in the Tn*5253* element. Overall, 68.75 % of the West African ST217 isolates carried the Tn*5253* element without chloramphenicol resistance gene and in comparison 5.17 % of the South African isolates in clade (SC1-SA) and none in the South Eastern African clade (SC3-SEA) carried the defective element (Fig. 5d).

## Discussion

IPD due to serotype 1 pneumococcus in Sub Saharan Africa is predominantly caused by the endemic ST217 clone. Our pneumococcal population genomic dataset offers a unique opportunity for understanding how this clone has evolved and spread across and outside the continent. Our findings showed evolution of the ST217 clone into geographically distinct lineages with different characteristics. Previous studies have shown that serotype 1 is highly clonal [17] and exhibits strong phylogeographic structure [15]. These characteristics may allow for accurate inference of recent spread of the ST217 clone between countries. Our findings show with higher genomic resolution the recent spread of the ST217 clone. We identified both short-range and long-range transmission of the clone within closely located African countries and between West Africa and Southern Africa and between West African and Asia. In most instances we were able to establish the direction of spread of the clone between countries, but in others we were only able to detect potential spread of the ST217 clone without inferring the directionality. Nevertheless, our findings show the intracontinental and intercontinental spread of the ST217 clone with high resolution using whole genome sequencing.

The ST217 SCs restricted to different geographical regions may experience different selective pressures. Our findings showed consistently lower than expected amount of polymorphism under the mutation drift equilibrium. All the lineages showed negative estimates for the Tajima D statistic which suggested genome-wide selective sweeps and potentially population bottlenecks such as those that would be expected to accompany periodic epidemic waves [66]. Temporal coalescent divergence dating using isolation years and phylogenies revealed recent emergence of the SCs. The typically South Eastern Africa clade (SC3-SEA) and the Southern African clade (SC1-SA) emerged in the 1980s and the early 1990s respectively while the emergence of the West African clade (SC2-WA) could not be fully determined because of the lack of the molecular clock signal or linear evolution with time in this clade. Overall, our findings clearly suggest recent emergence and clonal expansion of the ST217 in SSA countries within the last century. In the South Eastern African clade, the relative genetic diversity or the effective population size (N_e_τ) of the lineage, increased rapidly in the 1990s until early 2000s followed by a decline around 2005. The skyline plot suggests that the population then remained stable until the last sampling point in 2010, but this must be treated with caution because of the possibility that in this more recent time period, the relatively scant data leads the analysis to recover the prior [66]. The decline in the population size in clade SC3-SEA coincided with the previously reported decline in IPD in Malawi after scale-up of anti-retroviral therapy and cotrimoxazole prophylaxis [67]. In the South African clade, the population size increased slowly from late 1990s and has consistently remained higher than in Malawi and Mozambique from mid-2000s until 2010, possibly reflecting the higher diversity of the South African population. In addition, the observed mutation rates in both clades were similar to previously published estimates which further suggests that differences in recombination rather than mutation rates drive variations in genetic diversity between pneumococcal lineages vary [68].

Serotype 1 pneumococci are rarely detected in carriage and our study provides an additional explanation for this. We show that certain key pneumococcal loci important for nasopharyngeal colonisation are absent from ST217 isolates including the pneumococcal pilus operon genes, *psrP, iga, zmpB* and *zmpC.* These genes promote adherence to the epithelial surfaces as adhesins [62–64, 69–71]. Of particular interest was the *iga* gene, which encodes a protein that cleaves human IgA1 protease and facilitate colonisation [62]. To determine the distribution of the absent genes in other pneumococcal lineages, we screened the genes in isolates from Massachussetts population [58]. Overall, both pilus operon genes, *psrP, iga, zmpB* and *zmpC* were present in between 5 and 20 % of the dataset suggesting that while these genes may indeed promote colonisation, they cannot fully explain serotype 1’s absence in carriage compared to other lineages because they were present in other lineages carried for longer durations than serotype 1. Interestingly, in the Massachusetts dataset, the pilus associated with highly carried lineages such as serogroup 6, 19 F and 35B while *iga* associated with lineages containing serotypes such as 11A, 15A,19A and 35 F and non-typeable pneumococci. On the other hand, *psrP* was sporadically distributed across various lineages in the Massachusetts dataset but was completely absent in serotype 1. Majority of the virulence genes such as pneumolysin (*ply*), choline-binding proteins such as *cbpA,* competence genes (*comA-E*), and neuraminidases (*nanA* and *nanB*) and various cell surface antigens such as *psaA-C* were ubiquitous in serotype 1 [53]. This is unsurprising because serotype 1 isolates are well-known for its high invasive capacity and inability to efficiently colonise the nasopharynx and sustain long durations of carriage. Such inability to colonise humans may be primarily a consequence of its polysaccharide capsule characteristics, which elicits phagocytic killing and rapid clearance [72] but the absence of the genes important for colonisation such as *iga* may also contribute to this effect. In turn, this may limit serotype 1’s exposure to potential donors of DNA thus causing the observed lower rates of recombination since recombination occurs efficiently during carriage [13, 73]. However, to validate this hypothesis, further in vitro and in vivo studies are required to determine the biological functions and pathways affected by the absent genes and the role of the capsule with regards to clearance by the immune system.

Occurrence of recombination events has been previously associated with acquisition of antibiotic resistance as such lower rates of recombination may have consequences in acquisition of the antibiotic resistance genes [74]. The South African clade (SC1-SA) contained the lowest amount of recombination and showed virtually no acquisition of the antibiotic resistance conferring elements compared to other SCs. This may have driven the lower acquisition of antibiotic resistance conferring mobile genetic elements in this population and lineage as evidenced by the lower (~27 %) resistance to tetracycline and chloramphenicol, which is among the lowest for this clone in Sub Saharan Africa [75]. On the other hand, no macrolide resistance conferring elements were identified in all the SCs, which is reassuring and suggests that macrolides may still be a preferable choice of treatment for the foreseeable future in patients infected with this clone. Clades SC2-WA, which predominantly comprised of West African isolates and clade SC3-SEA predominantly consisting of isolates from Southern East African countries such as Malawi, showed higher recombination rates and antibiotic resistance particularly for chloramphenicol and tetracycline. Interestingly, the observed higher resistance to tetracycline was much higher than to chloramphenicol in the West African lineage despite the presence of only Tn*5253* element which carries resistance conferring genes for both antibiotics [76]. This observation was consistent with previous study from the Gambia, which also reported such a discrepancy using in vitro phenotypic data [77]. Because resistance to both antibiotics was due to the presence of only the Tn*5253* element, this suggested that the chloramphenicol resistant isolates contained a deletion of the chloramphenicol resistance encoding loci. Interestingly, further comparative genomic analysis of the tetracycline resistant but chloramphenicol susceptible isolates revealed that these isolates harbored a defective Tn*5253* element with an intact tetracycline resistance conferring gene (*tetM*) and a large genomic deletion (~5Kb) across the pC194 plasmid, which harbors the chloramphenicol resistance conferring gene (*cat*_pC194_). We confirmed this deletion by mapping raw reads from the isolates with the putative deletion against an intact Tn*5253* reference sequence. However, Asian isolates in the West African clade, which represented intercontinentally spread isolates from West Africa, contained an intact Tn*5253* element despite having the same genetic background. This further suggests that the deletion of the chloramphenicol resistance encoding loci was restricted to the West African isolates. These findings explain why ST217 isolates from West Africa are more susceptible to chloramphenicol than tetracycline as previously reported [77].

However, an important yet unanswered question concerns what may have driven the loss of chloramphenicol resistance in the West African isolates but not Asian isolates of the same genetic background. Further analysis of previously published serotype 1 STs [15] showed that the deletion of the chloramphenicol gene was not restricted to only ST217 clone. Other closely related STs from West Africa such as ST303, which were single locus variants of the ST217 clone also showed widespread deletion of the chloramphenicol resistance conferring loci. This further suggests that the deletion was not recent and if it was recent it would imply that the defective Tn*5253* has spread at a high rate possibly as a consequence of selection in West Africa. During the sampling period, chloramphenicol was widely used in The Gambia [77] and possibly other West African countries while its use decreased in Southern African countries such as Malawi where all the resistant isolates harbored an intact Tn*5253* element. This may suggest that the observed widespread loss of chloramphenicol resistance mechanism in West African isolates may not be a consequence of low chloramphenicol usage.

## Conclusions

A potential limitation although not a concern for our study may be uneven number of ST217 isolates sampled from different countries, which does not imply higher incidence in some countries. Such differences are primarily because of deeper sampling in some countries. To avoid biases, we primarily focused our analysis based on the SCs rather than comparing their country-specific prevalence. Nevertheless, our study provides a comprehensive genomic portrait of the evolutionary dynamics of the hyper virulent ST217 serotype 1 clone. Overall we have demonstrated high genomic diversity and geographic structure of the clone, which despite recent emergence of the common ancestors for the SCs, suggests divergence of the clone into multiple SCs in the distant past evidenced by the deep branching between the SCs. Such geographical diversification of the SCs also reflects adaptations to local natural selective pressures. The higher acquisition of antibiotic resistance conferring elements in African isolates may explain why the ST217 clone has been remarkably successful in this continent despite spread of the clone to other continents. In West Africa, majority of the isolates harbour the defective Tn*5253* conjugative element with a large deletion in the locus harboring chloramphenicol resistance conferring gene but it still remains to be determined what may have driven the spread of this defective element in this region. The absence of certain genes known to promote nasopharyngeal colonisation may partly contribute to the rarity of serotype 1 during carriage in additional to its capsule, which is a major virulence factor. Such rarity in carriage may explain the observed lower recombination rates in the SCs compared to other globally successful clones such as PMEN1. However, additional in vivo and in vitro experiments coupled with whole genome sequencing are required to provide further insights into the biology of serotype 1 isolates.

## Additional files

Additional file 1: Characteristics and assembly statistics for the ST217 study isolates. (DOCX 166 kb)
Additional file 2: Clade specific phylogenies showing country of origin of the ST217 isolates. Maximum likelihood phylogenetic trees for clade (a) SC1-SA, (b) SC2-WA and (c) SC3-SEA. (PDF 292 kb)
Additional file 3: Estimated evolutionary parameters for all the clades. Each parameter estimate was calculated using MEGA. (DOC 80 kb)
Additional file 4: Recombination events identified in the South African clade (SC1-SA). (a) Maximum likelihood phylogeny showing genetic relationship of the isolates in the SC. Color strips labeled (b) and (c) shows country and continent of origin of the isolates respectively. (d) Annotations in the reference serotype 1 genome. (e) Horizontal tracks from the phylogenetic tips represent each genome. The coloured blocks shows locations of the recombination events in the chromosome. Red blocks show shared recombination events identified in at least two isolate while the blue blocks show strain specific (unique) recombination events. (PDF 641 kb)
Additional file 5: Recombination events identified in the West African clade (SC2-WA). (a) Maximum likelihood phylogeny showing genetic relationship of the isolates in the SC. Color strips labeled (b) and (c) shows country and continent of origin of the isolates respectively. (d) Annotations in the reference serotype 1 genome. (e) Horizontal tracks from the phylogenetic tips represent each genome. The coloured blocks shows locations of the recombination events in the chromosome. Red blocks show shared recombination events identified in at least two isolate while the blue blocks show strain specific (unique) recombination events. (PDF 1452 kb)
Additional file 6: Recombination events identified in the South East African clade (SC3-SEA). (a) Maximum likelihood phylogeny showing genetic relationship of the isolates in the SC. Color strips labeled (b) and (c) shows country and continent of origin of the isolates respectively. (d) Annotations in the reference serotype 1 genome. (e) Horizontal tracks from the phylogenetic tips represent each genome. The coloured blocks shows locations of the recombination events in the chromosome. Red blocks show shared recombination events identified in at least two isolate while the blue blocks show strain specific (unique) recombination events. (PDF 1077 kb)
Additional file 7: Summary of the genes found in the genetic recombination regions in clade SC1-SA. Genes present in the regions with recombination events in each clade are summarised. (DOCX 85 kb)
Additional file 8: Summary of the genes found in the genetic recombination regions in clade SC2-WA. Genes present in the regions with recombination events in each clade are summarised. (DOCX 124 kb)
Additional file 9: Summary of the genes found in the genetic recombination regions in clade SC3-SEA. Genes present in the regions with recombination events in each clade are summarised. (DOCX 133 kb)
Additional file 10: Intragenic number of observed and expected numbers of SNPs in the ST217 isolates. All the genes in the ST217 isolates, sizes, observed (O_SNP_) and expected (E_SNP_) number of SNPs, ratio O_SNP_ to E_SNP_ and products of each gene are summarised. (DOCX 315 kb)
Additional file 11: Distribution of genes associated with virulence and colonisation in the ST217 in serotype 1 isolates. Presence of the genes was screened in all the isolates using BLAST as described in the methods section. (PDF 1702 kb)
Additional file 12: Deletion of the immunoglobulin A (*iga*) protease gene in the ST217 isolates. Genomic comparison of one of the serotype 1 isolates (ERS194295) that lacked the *iga* gene against the TIGR4 reference *S. pneumoniae* genome with an intact *iga* gene in its chromosome to determine the location and structure of the genomic deletion in the ST217 isolates. Sequence comparison was performed by BLASTN and visualised with Artemis Comparison Tool (ACT). (PDF 528 kb)
Additional file 13: Distribution of the accessory genes and antibiotic resistance conferring elements in the ST217 isolates. a) Maximum likelihood phylogeny of the isolates rooted using isolates from ST615 as an outgroup (not shown). b) Number of shared accessory open reading frames (ORFs) or genes between pairs of isolates in the phylogeny on the left side and at the top. c) Colour strips showing the continent and country of origin of the isolates and their SCs. d) A heatmap showing the number of shared accessory genes between each pair of isolates in the phylogeny (panel [a] and [e]). (PDF 4076 kb)

## Abbreviations

CI: Confidence interval
HPD: Highest posterior density
ICE: Integrative conjugative element
IPD: Invasive pneumococcal disease
PCR: Polymerase chain reaction
PCV: Pneumococcal conjugate vaccine
SC: Sequence cluster
SNP: Single nucleotide polymorphism
