# Supplementary material for: Understanding pneumococcal serotype 1 biology through population genomic analysis
Source: BMC Infect Dis. 2016 Nov 8;16:649. doi: 10.1186/s12879-016-1987-z (PMC5100261; doi:10.1186/s12879-016-1987-z)
Supplement: Additional file 1: — Characteristics and assembly statistics for the ST217 study isolates. (DOCX 166 kb) [file 12879_2016_1987_MOESM1_ESM.docx]

| **Sample ID** | **Illumina Lane** | **Year** | **Country** | **Region** | **Continent** | **Age (Yrs)** | **Source** | **SC** | **Accession** |
| --- | --- | --- | --- | --- | --- | --- | --- | --- | --- |
|  |  |  |  |  |  |  |  |  |  |
| 21A150705 | 4525_2_10 | 2005 | South Africa | Gauteng | Africa | 30 | invasive | SC1 | ERS005545 |
| 22A110705 | 4525_2_11 | 2005 | South Africa | Gauteng | Africa | 24 | invasive | SC1 | ERS005554 |
| 23A310705 | 4525_2_12 | 2005 | South Africa |  | Africa | 33 | invasive | SC1 | ERS005552 |
| 25A270805 | 4525_2_3 | 2005 | South Africa | Gauteng | Africa | 23 | invasive | SC1 | ERS005540 |
| 26P291005 | 4525_2_4 | 2005 | South Africa | Western Cape | Africa | 12 | invasive | SC1 | ERS005547 |
| 16P210205 | 4525_2_6 | 2005 | South Africa | Western Cape | Africa |  | invasive | SC1 | ERS005550 |
| 17P270205 | 4525_2_7 | 2005 | South Africa | Gauteng | Africa | 14 | invasive | SC1 | ERS005543 |
| 19P150405 | 4525_2_8 | 2005 | South Africa | Eastern Cape | Africa | 10 | invasive | SC1 | ERS005544 |
| 27P091105 | 4525_3_1 | 2005 | South Africa |  | Africa |  | invasive | SC1 | ERS005557 |
| 33A040706 | 4525_3_10 | 2006 | South Africa | Gauteng | Africa | 17 | invasive | SC1 | ERS005564 |
| 35P050906 | 4525_3_12 | 2006 | South Africa | Mpumalanga | Africa |  | invasive | SC1 | ERS005563 |
| 36P280806 | 4525_3_2 | 2006 | South Africa | Mpumalanga | Africa | 10 | invasive | SC1 | ERS005548 |
| 37P270906 | 4525_3_3 | 2006 | South Africa | Gauteng | Africa |  | invasive | SC1 | ERS005551 |
| 38P071206 | 4525_3_4 | 2006 | South Africa | Gauteng | Africa |  | invasive | SC1 | ERS005555 |
| 29A310106 | 4525_3_6 | 2006 | South Africa | Gauteng | Africa | 37 | invasive | SC1 | ERS005559 |
| 30P100206 | 4525_3_7 | 2006 | South Africa | KwaZulu-Natal | Africa |  | invasive | SC1 | ERS005553 |
| 32P170306 | 4525_3_9 | 2006 | South Africa | Gauteng | Africa |  | invasive | SC1 | ERS005558 |
| 39A181206 | 4525_5_1 | 2006 | South Africa | Gauteng | Africa | 31 | invasive | SC1 | ERS005561 |
| 46P250907 | 4525_5_11 | 2007 | South Africa | Gauteng | Africa |  | invasive | SC1 | ERS005573 |
| 47P260907 | 4525_5_12 | 2007 | South Africa | Gauteng | Africa |  | invasive | SC1 | ERS005577 |
| 48A300907 | 4525_5_2 | 2007 | South Africa | Gauteng | Africa | 27 | invasive | SC1 | ERS005562 |
| 49A220807 | 4525_5_3 | 2007 | South Africa | KwaZulu-Natal | Africa | 26 | invasive | SC1 | ERS005567 |
| 50P021107 | 4525_5_4 | 2007 | South Africa | KwaZulu-Natal | Africa |  | invasive | SC1 | ERS005565 |
| 41P190507 | 4525_5_6 | 2007 | South Africa | Gauteng | Africa |  | invasive | SC1 | ERS005571 |
| 42P230507 | 4525_5_7 | 2007 | South Africa | Gauteng | Africa | 12 | invasive | SC1 | ERS005568 |
| 51P250907 | 4525_6_1 | 2007 | South Africa | KwaZulu-Natal | Africa |  | invasive | SC1 | ERS005576 |
| 52A111107 | 4525_6_5 | 2007 | South Africa | Gauteng | Africa | 34 | invasive | SC1 | ERS005581 |
| 58P290908 | 4525_6_11 | 2008 | South Africa | Gauteng | Africa |  | invasive | SC1 | ERS005584 |
| 59A061008 | 4525_6_12 | 2008 | South Africa | Gauteng | Africa | 45 | invasive | SC1 | ERS005583 |
| 60P131008 | 4525_6_2 | 2008 | South Africa | Gauteng | Africa |  | invasive | SC1 | ERS005574 |
| 61P231008 | 4525_6_3 | 2008 | South Africa | Gauteng | Africa |  | invasive | SC1 | ERS005582 |
| 62A271008 | 4525_6_4 | 2008 | South Africa | North West | Africa | 71 | invasive | SC1 | ERS005578 |
| 55P150608 | 4525_6_8 | 2008 | South Africa |  | Africa |  | invasive | SC1 | ERS005580 |
| 63P221108 | 4752_1_1 | 2008 | South Africa | Gauteng | Africa |  | invasive | SC1 | ERS005585 |
| 64P171208 | 4752_1_5 | 2008 | South Africa | Gauteng | Africa | 12 | invasive | SC1 | ERS005592 |
| PATH629 | 8793_8_48 | 2006 | Mozambique |  | Africa |  | invasive | SC1 | ERS133037 |
| PATH634 | 8793_8_49 | 2006 | Mozambique |  | Africa |  | invasive | SC1 | ERS133038 |
| PATH710 | 8793_8_56 | 2007 | Mozambique |  | Africa |  | invasive | SC1 | ERS133045 |
| 8P260904 | 4525_1_11 | 2004 | South Africa | KwaZulu-Natal | Africa |  | invasive | SC1 | ERS005542 |
| 10P101004 | 4525_1_12 | 2004 | South Africa | Gauteng | Africa |  | invasive | SC1 | ERS005535 |
| 11A051104 | 4525_1_2 | 2004 | South Africa | Gauteng | Africa | 83 | invasive | SC1 | ERS005530 |
| 2P190204 | 4525_1_5 | 2004 | South Africa | Gauteng | Africa |  | invasive | SC1 | ERS005531 |
| 3P200204 | 4525_1_6 | 2004 | South Africa | Western Cape | Africa | 10 | invasive | SC1 | ERS005536 |
| 6P090904 | 4525_1_9 | 2004 | South Africa | Gauteng | Africa |  | invasive | SC1 | ERS005537 |
| 14P130105 | 4525_2_1 | 2005 | South Africa | Gauteng | Africa | 11 | invasive | SC1 | ERS005546 |
| 4A240304 | 4525_1_7 | 2004 | South Africa | KwaZulu-Natal | Africa | 46 | Other | SC1 | ERS005534 |
| 12A271104 | 4525_1_3 | 2004 | South Africa | Gauteng | Africa | 37 | invasive | SC1 | ERS005532 |
| 53P090408 | 4525_6_6 | 2008 | South Africa | Gauteng | Africa |  | invasive | SC1 | ERS005587 |
| 1P050204 | 4525_1_1 | 2004 | South Africa | Gauteng | Africa | 12 | invasive | SC1 | ERS005527 |
| 34P050706 | 4525_3_11 | 2006 | South Africa | Gauteng | Africa | 10 | invasive | SC1 | ERS005566 |
| PATH621 | 8786_8_47 | 2006 | Mozambique |  | Africa |  | invasive | SC1 | ERS133036_ |
| PATH616 | 8793_8_46 | 2006 | Mozambique |  | Africa |  | invasive | SC1 | ERS133035 |
| PATH621 | 8793_8_47 | 2006 | Mozambique |  | Africa | 32 | invasive | SC1 | ERS133036 |
| PATH665 | 8793_8_54 | 2007 | Mozambique |  | Africa |  | invasive | SC1 | ERS133043 |
| PATH5550 | 9517_3_4 | 2008 | Mozambique |  | Africa | 41 | invasive | SC1 | ERS194287 |
| 57P190908 | 4525_6_10 | 2008 | South Africa | KwaZulu-Natal | Africa |  | invasive | SC1 | ERS005591 |
| PATH664 | 8793_8_53 | 2007 | Mozambique |  | Africa |  | invasive | SC1 | ERS133042 |
| 7A110904 | 4525_1_10 | 2004 | South Africa | Gauteng | Africa | 22 | invasive | SC1 | ERS005541 |
| NIG284-05 | 6570_4_14 | 2005 | Niger | Niamey | Africa |  | invasive | SC2 | ERS033812 |
| NIG14-04 | 6851_2_91 | 2004 | Niger | Niamey | Africa | 18 | invasive | SC2 | ERS033839 |
| NIG378-05 | 6570_4_16 | 2005 | Niger | Niamey | Africa | 19 | invasive | SC2 | ERS033814 |
| NIG545-05 | 6570_4_19 | 2005 | Niger | Tahoua | Africa | 10 | invasive | SC2 | ERS033817 |
| NIG591-04 | 6570_4_10 | 2004 | Niger | Niamey | Africa |  | invasive | SC2 | ERS033808 |
| NIG57-04 | 6570_4_8 | 2004 | Niger | Niamey | Africa |  | invasive | SC2 | ERS033806 |
| NIG98-05 | 6570_4_12 | 2005 | Niger | Niamey | Africa | 13 | invasive | SC2 | ERS033810 |
| PATH1572 | 8728_8_10 | 2005 | Thailand |  | Asia |  | invasive | SC2 | ERS133095 |
| PATH1575 | 8728_8_12 | 2005 | Thailand |  | Asia |  | invasive | SC2 | ERS133097 |
| NIG460-09 | 6851_2_88 | 2009 | Niger | Niamey | Africa | 25 | invasive | SC2 | ERS033833 |
| N2100A | 6308_8_2 | 2009 | Nigeria | South West | Africa |  | nasopharynx | SC2 | ERS024689 |
| PATH5360 | 8728_8_83 | 1996 | Philippines |  | Asia | 4 months | invasive | SC2 | ERS133168 |
| NIG1634-06 | 6851_2_79 | 2006 | Niger | Niamey | Africa | 42 | invasive | SC2 | ERS033824 |
| NIG38-08 | 6851_2_84 | 2008 | Niger | Niamey | Africa | 40 | invasive | SC2 | ERS033829 |
| PATH5359 | 8728_8_82 | 1996 | Philippines |  | Asia | 4 months | invasive | SC2 | ERS133167 |
| SVT_23964 | 6308_8_23 | 2006 | The Gambia | Western Division | Africa | 12 | nasopharynx | SC2 | ERS024710 |
| NIG716-05 | 6851_2_74 | 2005 | Niger | Niamey | Africa | 28 | invasive | SC2 | ERS033819 |
| PATH1652 | 8728_8_19 | 2005 | Thailand |  | Asia |  | invasive | SC2 | ERS133104 |
| PATH1653 | 8728_8_20 | 2005 | Thailand |  | Asia |  | invasive | SC2 | ERS133105 |
| PATH1658 | 8728_8_21 | 2005 | Thailand |  | Asia |  | invasive | SC2 | ERS133106 |
| NIG656-08 | 6851_2_86 | 2008 | Niger | Niamey | Africa | 38 | invasive | SC2 | ERS033831 |
| NIG2010-06 | 6851_2_80 | 2006 | Niger | Niamey | Africa |  | invasive | SC2 | ERS033825 |
| NIG137-07 | 6851_2_82 | 2007 | Niger | Niamey | Africa | 40 | invasive | SC2 | ERS033827 |
| NIG779-07 | 6851_2_83 | 2007 | Niger | Niamey | Africa |  | invasive | SC2 | ERS033828 |
| NIG640-05 | 6851_2_73 | 2005 | Niger | Niamey | Africa | 31 | invasive | SC2 | ERS033818 |
| PATH1573 | 8728_8_11 | 2005 | Thailand |  | Asia |  | invasive | SC2 | ERS133096 |
| SP1600 | 7712_7_54 | 2010 | Ghana | Upper East region | Africa |  | invasive | SC2 | ERS093292 |
| SP1621 | 7712_7_58 | 2010 | Ghana | Upper East region | Africa |  | invasive | SC2 | ERS093296 |
| PATH4231 | 8728_8_67 | 2006 | Ethiopia |  | Africa |  | nasopharynx | SC2 | ERS133152 |
| REFERENCE GENOME | spn1041 (REFERENCE GENOME) | NA | Ghana |  | Africa | 10 |  | SC2 | SPN1041 |
| PATH5620 | 9517_3_12 | 2009 | Mozambique |  | Africa | 35 | invasive | SC2 | ERS194295 |
| NIG1712-03 | 6570_4_6 | 2003 | Niger | Niamey | Africa | 11 | invasive | SC2 | ERS033803 |
| NIG2196-03 | 6570_4_7 | 2003 | Niger | Niamey | Africa | 22 | invasive | SC2 | ERS033804 |
| PATH5372 | 8728_8_86 | 1994 | Thailand |  | Asia |  | sputum | SC2 | ERS133171 |
| PATH5371 | 8728_8_85 | 1996 | Thailand |  | Asia |  | sputum | SC2 | ERS133170 |
| PATH1675 | 8728_8_22 | 2005 | Thailand |  | Asia |  | invasive | SC2 | ERS133107 |
| PATH1686 | 8728_8_25 | 2005 | Thailand |  | Asia |  | invasive | SC2 | ERS133110 |
| PATH1684 | 8728_8_24 | 2005 | Thailand |  | Asia |  | invasive | SC2 | ERS133109 |
| PATH1692 | 8728_8_26 | 2005 | Thailand |  | Asia |  | invasive | SC2 | ERS133111 |
| PATH1696 | 8728_8_27 | 2005 | Thailand |  | Asia |  | invasive | SC2 | ERS133112 |
| PATH5389 | 8728_8_87 | 1998 | Thailand |  | Asia | 144 mos | invasive | SC2 | ERS133172 |
| PATH1634 | 8728_8_17 | 2005 | Thailand |  | Asia |  | invasive | SC2 | ERS133102 |
| PATH767 | 8793_8_64 | 2005 | Thailand |  | Asia |  | invasive | SC2 | ERS133053 |
| PATH1581 | 8728_8_13 | 2005 | Thailand |  | Asia |  | invasive | SC2 | ERS133098 |
| PATH1584 | 8728_8_14 | 2005 | Thailand |  | Asia |  | invasive | SC2 | ERS133099 |
| PATH1645 | 8728_8_18 | 2005 | Thailand |  | Asia |  | invasive | SC2 | ERS133103 |
| SP1610 | 7712_7_70 | 2010 | Ghana | Upper East region | Africa |  | invasive | SC2 | ERS093308 |
| NIG460-06 | 6851_2_75 | 2006 | Niger |  | Africa | 60 | invasive | SC2 | ERS033820 |
| NIG1144-06 | 6851_2_77 | 2006 | Niger | Dosso | Africa | 25 | invasive | SC2 | ERS033822 |
| NIG2645-09 | 6851_2_90 | 2009 | Niger | Niamey | Africa | 10 | invasive | SC2 | ERS033835 |
| PATH385 | 8793_8_13 | 1999 | Egypt |  | Africa |  | invasive | SC2 | ERS133002 |
| N1335A | 6308_8_3 | 2009 | Nigeria | South West | Africa |  | nasopharynx | SC2 | ERS024727 |
| 20A100605 | 4525_2_9 | 2005 | South Africa | Gauteng | Africa | 48 | invasive | SC2 | ERS005549 |
| 4691 | 9517_3_37 | 1997 | Malawi |  | Africa | 15 |  | SC3 | ERS194329 |
| 10243 | 9517_3_45 | 1998 | Malawi | Blantyre | Africa |  |  | SC3 | ERS194337 |
| A2766 | 9517_3_48 | 1998 | Malawi | Blantyre | Africa |  | invasive | SC3 | ERS194340 |
| A2844 | 9517_3_56 | 1998 | Malawi | Blantyre | Africa |  | invasive | SC3 | ERS194348 |
| A2333 | 9517_4_28 | 1998 | Malawi | Blantyre | Africa |  | invasive | SC3 | ERS194432 |
| B352 | 9517_4_31 | 1998 | Malawi | Blantyre | Africa |  | invasive | SC3 | ERS194438 |
| B022 | 9517_4_38 | 1998 | Malawi | Blantyre | Africa |  | invasive | SC3 | ERS194449 |
| C340 | 9517_3_52 | 1999 | Malawi | Blantyre | Africa |  | invasive | SC3 | ERS194344 |
| B933 | 9517_3_59 | 1999 | Malawi | Blantyre | Africa |  | invasive | SC3 | ERS194351 |
| C462 | 9517_3_68 | 1999 | Malawi | Blantyre | Africa |  | invasive | SC3 | ERS194360 |
| A4648 | 9517_3_72 | 1999 | Malawi | Blantyre | Africa |  | invasive | SC3 | ERS194364 |
| C1349 | 9517_3_75 | 1999 | Malawi | Blantyre | Africa |  | invasive | SC3 | ERS194367 |
| C474 | 9517_3_76 | 1999 | Malawi | Blantyre | Africa |  | invasive | SC3 | ERS194368 |
| D4098 | 9517_4_76 | 1999 | Malawi | Blantyre | Africa |  | invasive | SC3 | ERS194464 |
| B1810 | 9517_3_42 | 2000 | Malawi | Blantyre | Africa |  | invasive | SC3 | ERS194334 |
| A7506 | 9517_3_49 | 2000 | Malawi | Blantyre | Africa |  | invasive | SC3 | ERS194341 |
| D7954 | 9517_4_57 | 2000 | Malawi | Blantyre | Africa |  | invasive | SC3 | ERS194433 |
| A9702 | 9517_4_6 | 2000 | Malawi | Blantyre | Africa |  | invasive | SC3 | ERS194398 |
| D8394 | 9517_4_62 | 2000 | Malawi | Blantyre | Africa |  | invasive | SC3 | ERS194446 |
| D8420 | 9517_4_67 | 2000 | Malawi | Blantyre | Africa |  | invasive | SC3 | ERS194455 |
| D4676 | 9517_4_80 | 2000 | Malawi | Blantyre | Africa |  | invasive | SC3 | ERS194468 |
| C3389 | 9517_3_44 | 2001 | Malawi | Blantyre | Africa |  | invasive | SC3 | ERS194336 |
| C3734 | 9517_3_60 | 2001 | Malawi | Blantyre | Africa |  | invasive | SC3 | ERS194352 |
| A12283 | 9517_4_11 | 2001 | Malawi | Blantyre | Africa |  | invasive | SC3 | ERS194405 |
| B2737 | 9517_4_15 | 2001 | Malawi | Blantyre | Africa |  | invasive | SC3 | ERS194413 |
| A13889 | 9517_4_27 | 2001 | Malawi | Blantyre | Africa |  | invasive | SC3 | ERS194430 |
| A14775 | 9517_4_35 | 2001 | Malawi | Blantyre | Africa |  | invasive | SC3 | ERS194443 |
| C3381 | 9517_4_40 | 2001 | Malawi | Blantyre | Africa |  | invasive | SC3 | ERS194453 |
| A17111 | 9517_3_71 | 2002 | Malawi | Blantyre | Africa |  |  | SC3 | ERS194363 |
| A17660 | 9517_3_79 | 2002 | Malawi | Blantyre | Africa |  | invasive | SC3 | ERS194371 |
| C5496 | 9517_4_1 | 2002 | Malawi | Blantyre | Africa |  | invasive | SC3 | ERS194389 |
| A13323 | 9517_4_19 | 2002 | Malawi | Blantyre | Africa |  | invasive | SC3 | ERS194417 |
| D17314 | 9517_4_41 | 2002 | Malawi | Blantyre | Africa |  | invasive | SC3 | ERS194390 |
| D17566 | 9517_4_51 | 2002 | Malawi | Blantyre | Africa |  | invasive | SC3 | ERS194418 |
| A25947 | 9517_3_40 | 2003 | Malawi | Blantyre | Africa |  | invasive | SC3 | ERS194332 |
| B5845 | 9517_3_51 | 2003 | Malawi | Blantyre | Africa |  | invasive | SC3 | ERS194343 |
| A24282 | 9517_4_36 | 2003 | Malawi | Blantyre | Africa |  | invasive | SC3 | ERS194445 |
| A21059 | 9517_4_4 | 2003 | Malawi | Blantyre | Africa |  | invasive | SC3 | ERS194395 |
| D20140 | 9517_4_61 | 2003 | Malawi | Blantyre | Africa |  | invasive | SC3 | ERS194444 |
| B10454 | 4525_7_11 | 2004 | Malawi | Blantyre | Africa |  | invasive | SC3 | ERS005615 |
| B10261 | 4525_7_6 | 2004 | Malawi | Blantyre | Africa | 23 | invasive | SC3 | ERS005611 |
| D28368 | 4525_7_8 | 2004 | Malawi | Blantyre | Africa |  | invasive | SC3 | ERS005613 |
| D31094 | 4525_7_1 | 2005 | Malawi | Blantyre | Africa |  | invasive | SC3 | ERS005602 |
| C11680 | 4752_1_10 | 2005 | Malawi |  | Africa |  | invasive | SC3 | ERS005594 |
| B11955a | 4752_1_12 | 2005 | Malawi |  | Africa | 33 | invasive | SC3 | ERS005599 |
| D31030 | 4752_1_6 | 2005 | Malawi | Blantyre | Africa |  | invasive | SC3 | ERS005596 |
| A37225 | 4880_1_10 | 2005 | Malawi | Blantyre | Africa | 25 | invasive | SC3 | ERS005635 |
| A42174_A | 11250_1_23 | 2006 | Malawi | Blantyre | Africa |  | invasive | SC3 | ERS353645 |
| B13691 | 4752_1_8 | 2006 | Malawi | Blantyre | Africa | 20 | invasive | SC3 | ERS005598 |
| C14599 | 4752_1_9 | 2006 | Malawi | Blantyre | Africa | 12 | invasive | SC3 | ERS005597 |
| D43846 | 4525_7_2 | 2007 | Malawi | Blantyre | Africa |  | invasive | SC3 | ERS005600 |
| A47157 | 4525_8_11 | 2007 | Malawi | Blantyre | Africa | 22 | invasive | SC3 | ERS005628 |
| C17094 | 4525_8_8 | 2007 | Malawi | Blantyre | Africa | 12 | invasive | SC3 | ERS005623 |
| C19295 | 4525_7_5 | 2008 | Malawi | Blantyre | Africa |  | invasive | SC3 | ERS005610 |
| C18896 | 4525_8_10 | 2008 | Malawi | Blantyre | Africa |  | invasive | SC3 | ERS005625 |
| A53636 | 4525_8_3 | 2008 | Malawi | Blantyre | Africa | 23 | invasive | SC3 | ERS005621 |
| D47858 | 4752_1_2 | 2008 | Malawi | Southern | Africa |  | invasive | SC3 | ERS005589 |
| D48334 | 4880_1_8 | 2008 | Malawi | Blantyre | Africa |  | invasive | SC3 | ERS005637 |
| D48470 | 4880_1_9 | 2008 | Malawi | Blantyre | Africa |  | invasive | SC3 | ERS005634 |
| B19220 | 4525_7_9 | 2009 | Malawi | Blantyre | Africa | 48 | invasive | SC3 | ERS005617 |
| D51831 | 4525_8_1 | 2009 | Malawi | Blantyre | Africa |  | invasive | SC3 | ERS005616 |
| A59803 | 9517_3_41 | 2010 | Malawi | Blantyre | Africa | 29 | invasive | SC3 | ERS194333 |
| A58029 | 9517_4_13 | 2010 | Malawi | Blantyre | Africa |  | invasive | SC3 | ERS194409 |
| BBW12U | 9517_3_67 | 2011 | Malawi | Blantyre | Africa |  | invasive | SC3 | ERS194359 |
| 1008637 | 9517_3_69 | 2011 | Malawi | Blantyre | Africa |  |  | SC3 | ERS194361 |
| 1010094 | 9517_3_85 | 2011 | Malawi | Blantyre | Africa |  |  | SC3 | ERS194377 |
| PATH597 | 8786_8_41 | 2006 | Mozambique |  | Africa |  | invasive | SC3 | ERS133030 |
| PATH602 | 8786_8_42 | 2006 | Mozambique |  | Africa |  | invasive | SC3 | ERS133031 |
| PATH611 | 8786_8_43 | 2006 | Mozambique |  | Africa |  | invasive | SC3 | ERS133032 |
| PATH497 | 8793_8_21 | 2006 | Mozambique |  | Africa |  | invasive | SC3 | ERS133010 |
| PATH505 | 8793_8_22 | 2006 | Mozambique |  | Africa |  | invasive | SC3 | ERS133011 |
| PATH612 | 8793_8_44 | 2006 | Mozambique |  | Africa |  | invasive | SC3 | ERS133033 |
| PATH614 | 8793_8_45 | 2006 | Mozambique |  | Africa | 32 | invasive | SC3 | ERS133034 |
| PATH637 | 8793_8_50 | 2006 | Mozambique |  | Africa |  | invasive | SC3 | ERS133039 |
| PATH645 | 8793_8_51 | 2006 | Mozambique |  | Africa |  | invasive | SC3 | ERS133040 |
| 45A260807 | 4525_5_10 | 2007 | Mozambique | Gauteng | Africa | 38 | invasive | SC3 | ERS005575 |
| PATH658 | 8793_8_52 | 2007 | Mozambique |  | Africa |  | invasive | SC3 | ERS133041 |
| PATH666 | 8793_8_55 | 2007 | Mozambique |  | Africa |  | invasive | SC3 | ERS133044 |
| PATH720 | 8793_8_57 | 2007 | Mozambique |  | Africa |  | invasive | SC3 | ERS133046 |
| PATH722 | 8793_8_58 | 2007 | Mozambique |  | Africa | 14 | invasive | SC3 | ERS133047 |
| PATH734 | 8793_8_60 | 2007 | Mozambique |  | Africa | 11 | invasive | SC3 | ERS133049 |
| PATH741 | 8793_8_61 | 2007 | Mozambique |  | Africa |  | invasive | SC3 | ERS133050 |
| PATH742 | 8793_8_62 | 2007 | Mozambique |  | Africa |  | invasive | SC3 | ERS133051 |
| PATH753 | 8793_8_63 | 2007 | Mozambique |  | Africa |  | invasive | SC3 | ERS133052 |
| PATH5604 | 9517_3_10 | 2008 | Mozambique |  | Africa | 34 | invasive | SC3 | ERS194293 |
| PATH5524 | 9517_3_3 | 2008 | Mozambique |  | Africa | 29 | invasive | SC3 | ERS194286 |
| PATH5565 | 9517_3_5 | 2008 | Mozambique |  | Africa |  | invasive | SC3 | ERS194288 |
| PATH5579 | 9517_3_6 | 2008 | Mozambique |  | Africa |  | invasive | SC3 | ERS194289 |
| PATH5598 | 9517_3_8 | 2008 | Mozambique |  | Africa | 16 months | invasive | SC3 | ERS194291 |
| PATH5602 | 9517_3_9 | 2008 | Mozambique |  | Africa | 61 months | invasive | SC3 | ERS194292 |
| PATH5420 | 8728_8_89 | 2010 | Mozambique |  | Africa | 160 moths | invasive | SC3 | ERS133174 |
| PATH5433 | 8728_8_92 | 2010 | Mozambique |  | Africa | 148 months | invasive | SC3 | ERS133177 |
| PATH5445 | 8728_8_93 | 2010 | Mozambique |  | Africa | 21 months | invasive | SC3 | ERS133178 |
| PATH5446 | 8728_8_94 | 2010 | Mozambique |  | Africa | 21 months | invasive | SC3 | ERS133179 |
| PATH5491 | 9517_3_1 | 2010 | Mozambique |  | Africa | 79 | invasive | SC3 | ERS194284 |
| 44A170607 | 4525_5_9 | 2007 | South Africa | Gauteng | Africa | 31 | invasive | SC3 | ERS005572 |
| PNI_643 | 6309_7_3 | 2005 | The Gambia | Western Division | Africa | 34 | invasive | SC3 | ERS024711 |
| PSP_1881 | 6308_7_7 | 2009 | The Gambia | Upper Region Division | Africa |  | invasive | SC3 | ERS024670 |
| PNI_830 | 6309_7_20 | 2009 | The Gambia | Western Division | Africa | 45 | lung aspirate | SC3 | ERS024659 |
| A32245 | 4752_1_11 | 2004 | Malawi |  | Africa | 30 | invasive | SC3 | ERS005601 |
| D28324 | 4880_1_1 | 2004 | Malawi | Blantyre | Africa |  | invasive | SC3 | ERS005626 |
| A59440 | 9517_4_29 | 2010 | Malawi | Blantyre | Africa |  | invasive | SC3 | ERS194434 |
| PATH5489 | 8728_8_96 | 2010 | Mozambique |  | Africa |  | invasive | SC3 | ERS133181 |
| MLAB4946 | 4880_1_7 | 2005 | Malawi | Blantyre | Africa | 30 | Carriage | SC3 | ERS005632 |
| C14269 | 4525_7_10 | 2006 | Malawi | Blantyre | Africa | 12 | invasive | SC3 | ERS005614 |
| D38050 | 4880_1_5 | 2006 | Malawi | Blantyre | Africa |  | invasive | SC3 | ERS005633 |
| B16942a | 4525_8_12 | 2007 | Malawi | Blantyre | Africa | 20 | invasive | SC3 | ERS005638 |
| 1018666 | 9517_3_70 | 2011 | Malawi | Blantyre | Africa |  |  | SC3 | ERS194362 |
| 1009310 | 9517_3_77 | 2011 | Malawi | Blantyre | Africa |  |  | SC3 | ERS194369 |
| A23188 | 9517_4_20 | 2003 | Malawi | Blantyre | Africa |  | invasive | SC3 | ERS194419 |
| PATH4006 | 8728_8_58 | 2009 | Qatar |  | Asia | 4 months | invasive | SC4 | ERS133143 |
| PATH357 | 8793_8_10 | 2008 | India |  | Asia |  |  | SC4 | ERS132999 |
| PATH345 | 8793_8_8 | 2008 | India |  | Asia |  |  | SC4 | ERS132997 |
| PATH346 | 8793_8_9 | 2008 | India |  | Asia | 50 |  | SC4 | ERS132998 |
| PATH4156 | 8728_8_66 | 2006 | Ethiopia |  | Africa |  | nasopharynx | SC4 | ERS133151 |
| PATH1768 | 8728_8_30 | 2007 | India |  | Asia |  |  | SC5 | ERS133115 |
