# Supplementary material for: Understanding pneumococcal serotype 1 biology through population genomic analysis
Source: BMC Infect Dis. 2016 Nov 8;16:649. doi: 10.1186/s12879-016-1987-z (PMC5100261; doi:10.1186/s12879-016-1987-z)
Supplement: Additional file 3: — Estimated evolutionary parameters for all the clades. Each parameter estimate was calculated using MEGA. (DOC 80 kb) [file 12879_2016_1987_MOESM3_ESM.doc]

| **Lineage** | **Number of Isolates (N)** | **Polymorphic Sites (S)** | **S per Isolate (S/N)** | **Proportion of S (P_s_)** | **Expected Sequence Diversity (π)** | **Observed Sequence Diversity (θ)** | **Tajima’s D** |
| --- | --- | --- | --- | --- | --- | --- | --- |
| **SC1-SA** | 58 | 854 | 14.72 | 4.55×10^-4^ | 9.80×10^-5^ | 3.00×10^-5^ | -2.77 |
| **SC2-WA** | 53 | 6028 | 113.74 | 3.09×10^-3^ | 6.82×10^-4^ | 1.63×10^-4^ | -2.79 |
| **SC3-SEA** | 110 | 4616 | 41.96 | 2.46×10^-3^ | 4.67×10^-4^ | 1.00×10^-4^ | -2.66 |
