# Supplementary figures and images for: Understanding pneumococcal serotype 1 biology through population genomic analysis
Source: BMC Infect Dis. 2016 Nov 8;16:649. doi: 10.1186/s12879-016-1987-z (PMC5100261; doi:10.1186/s12879-016-1987-z)

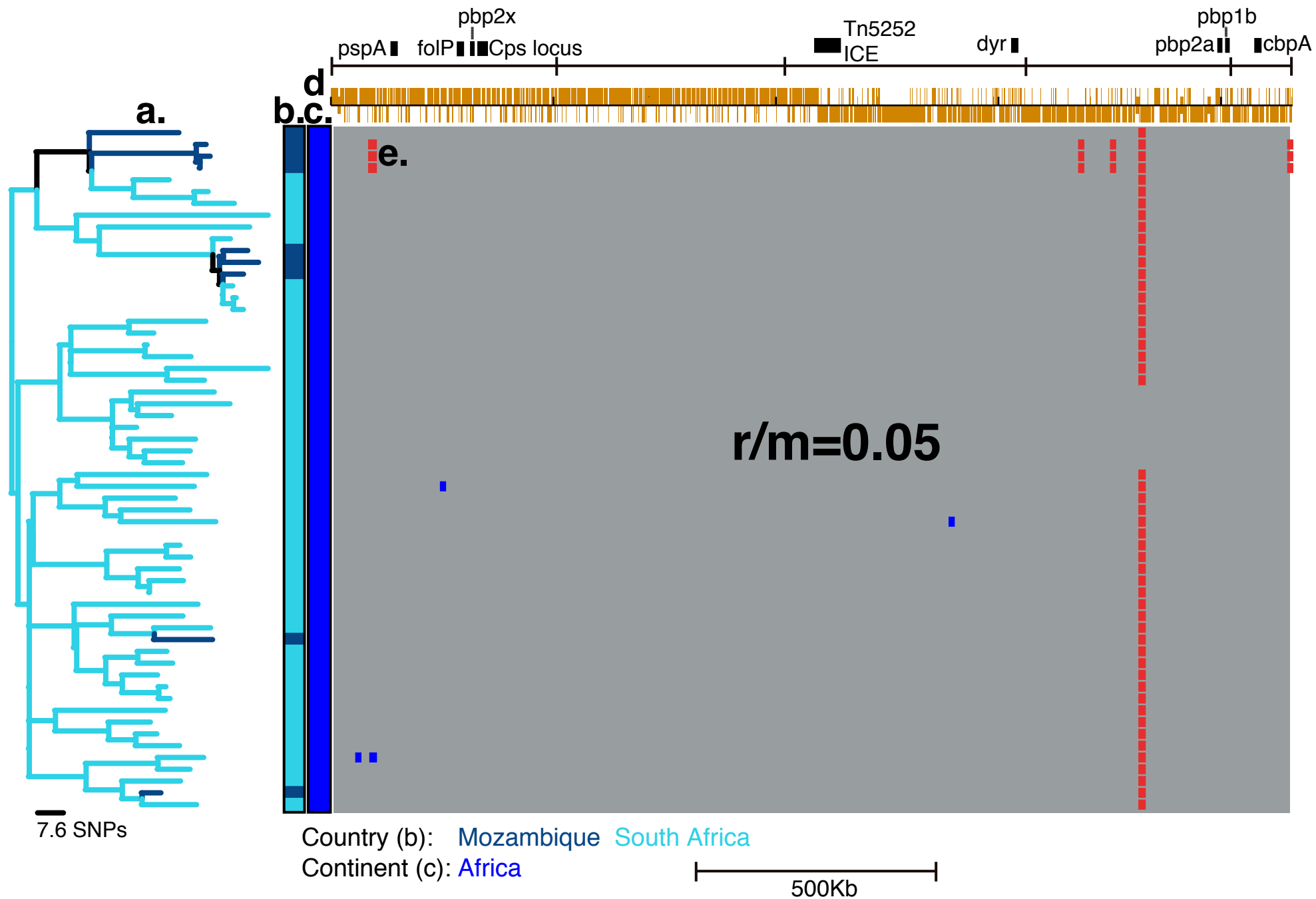

Supplement: Additional file 4: — Recombination events identified in the South African clade (SC1-SA). (a) Maximum likelihood phylogeny showing genetic relationship of the isolates in the SC. Color strips labeled (b) and (c) shows country and continent of origin of the isolates respectively. (d) Annotations in the reference serotype 1 genome. (e) Horizontal tracks from the phylogenetic tips represent each genome. The coloured blocks shows locations of the recombination events in the chromosome. Red blocks show shared recombination events identified in at least two isolate while the blue blocks show strain specific (unique) recombination events. (PDF 641 kb) [file 12879_2016_1987_MOESM4_ESM.pdf]

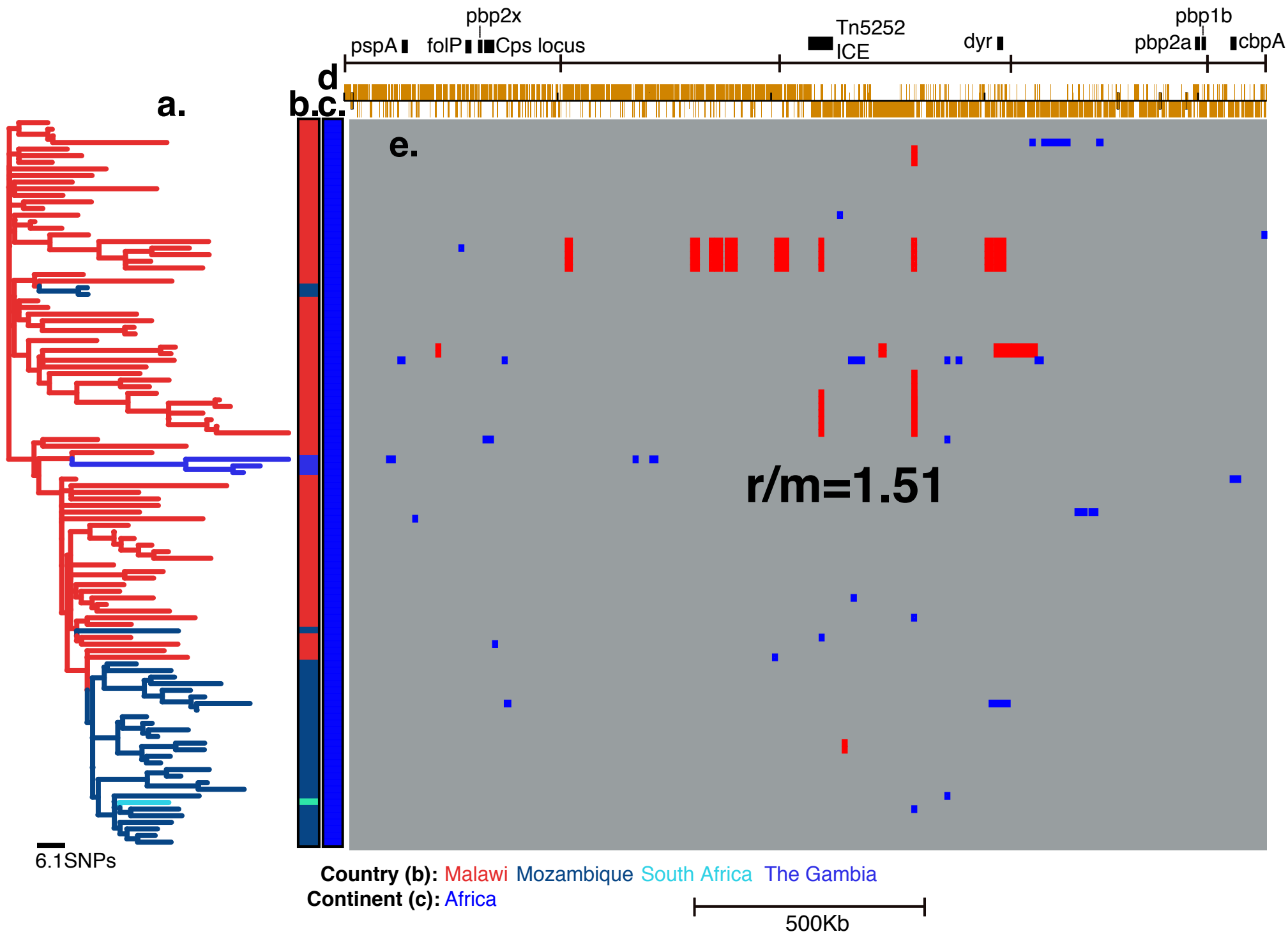

Supplement: Additional file 5: — Recombination events identified in the West African clade (SC2-WA). (a) Maximum likelihood phylogeny showing genetic relationship of the isolates in the SC. Color strips labeled (b) and (c) shows country and continent of origin of the isolates respectively. (d) Annotations in the reference serotype 1 genome. (e) Horizontal tracks from the phylogenetic tips represent each genome. The coloured blocks shows locations of the recombination events in the chromosome. Red blocks show shared recombination events identified in at least two isolate while the blue blocks show strain specific (unique) recombination events. (PDF 1452 kb) [file 12879_2016_1987_MOESM5_ESM.pdf]

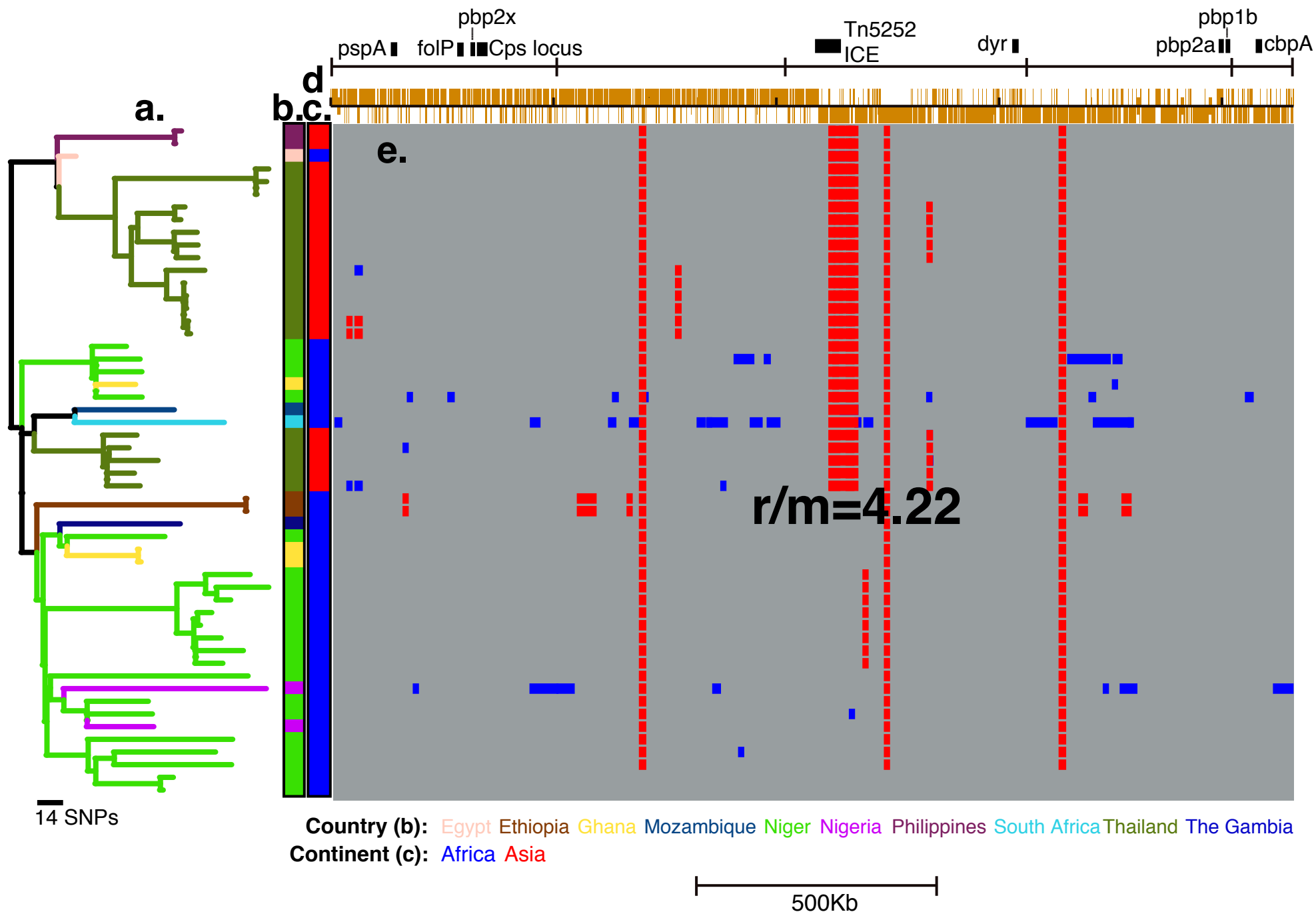

Supplement: Additional file 6: — Recombination events identified in the South East African clade (SC3-SEA). (a) Maximum likelihood phylogeny showing genetic relationship of the isolates in the SC. Color strips labeled (b) and (c) shows country and continent of origin of the isolates respectively. (d) Annotations in the reference serotype 1 genome. (e) Horizontal tracks from the phylogenetic tips represent each genome. The coloured blocks shows locations of the recombination events in the chromosome. Red blocks show shared recombination events identified in at least two isolate while the blue blocks show strain specific (unique) recombination events. (PDF 1077 kb) [file 12879_2016_1987_MOESM6_ESM.pdf]

# Strain ERS194295

Prophage (P1031)

aqaS

rpsD

46200

83600

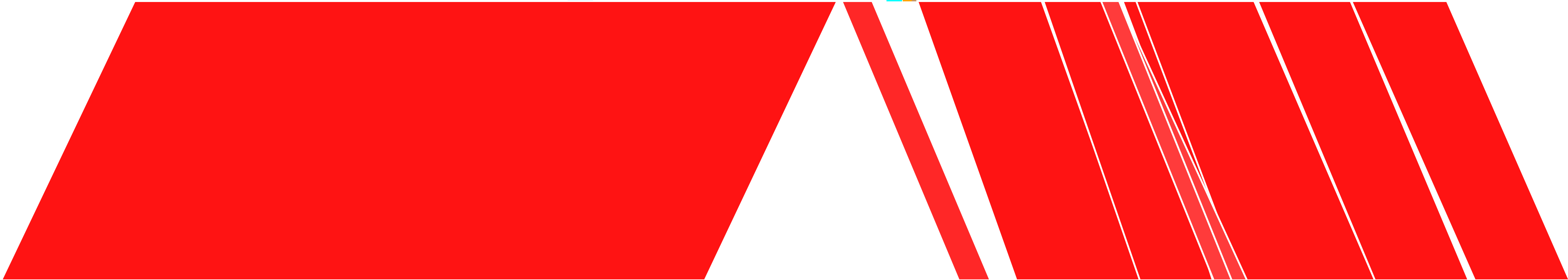

iga

rpsD

57200

94600

TIGR4

Supplement: Additional file 12: — Deletion of the immunoglobulin A (iga) protease gene in the ST217 isolates. Genomic comparison of one of the serotype 1 isolates (ERS194295) that lacked the iga gene against the TIGR4 reference S. pneumoniae genome with an intact iga gene in its chromosome to determine the location and structure of the genomic deletion in the ST217 isolates. Sequence comparison was performed by BLASTN and visualised with Artemis Comparison Tool (ACT). (PDF 528 kb) [file 12879_2016_1987_MOESM12_ESM.pdf]

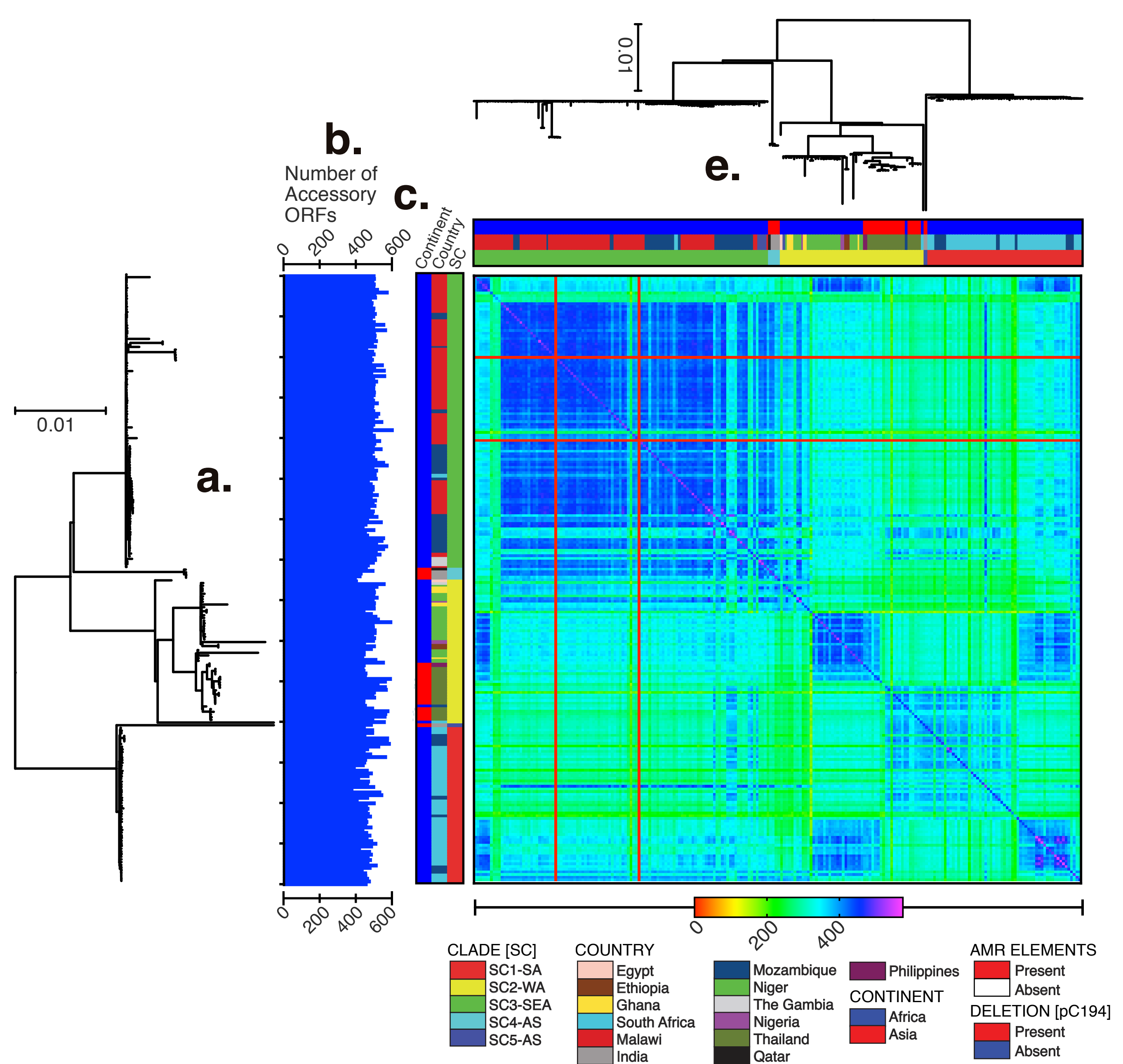

Supplement: Additional file 13: — Distribution of the accessory genes and antibiotic resistance conferring elements in the ST217 isolates. a) Maximum likelihood phylogeny of the isolates rooted using isolates from ST615 as an outgroup (not shown). b) Number of shared accessory open reading frames (ORFs) or genes between pairs of isolates in the phylogeny on the left side and at the top. c) Colour strips showing the continent and country of origin of the isolates and their SCs. d) A heatmap showing the number of shared accessory genes between each pair of isolates in the phylogeny (panel [a] and [e]). (PDF 4076 kb) [file 12879_2016_1987_MOESM13_ESM.pdf]
