# Supplementary material for: Understanding pneumococcal serotype 1 biology through population genomic analysis
Source: BMC Infect Dis. 2016 Nov 8;16:649. doi: 10.1186/s12879-016-1987-z (PMC5100261; doi:10.1186/s12879-016-1987-z)
Supplement: Additional file 7: — Summary of the genes found in the genetic recombination regions in clade SC1-SA. Genes present in the regions with recombination events in each clade are summarised. (DOCX 85 kb) [file 12879_2016_1987_MOESM7_ESM.docx]

| **Recombination Start** | **Recombination End** | **Feature Type** | **Feature Start** | **Feature End** | **Gene** | **Locus Name** | **D39 Ortholog** | **TIGR4 Ortholog** | **Notes/Comments** | **Product Name** |
| --- | --- | --- | --- | --- | --- | --- | --- | --- | --- | --- |
| 34942 | 34942 | rRNA | 24224 | 59696 |  |  |  |  | Prophage (P1031) |  |
| 34942 | 34942 | rRNA | 24224 | 59696 |  |  |  |  | Prophage (P1031) |  |
| 154357 | 154357 | CDS | 153382 | 155338 | *pspA* | INV10400930 |  |  |  | Pneumococcal surface protein A |
| 53142 | 53142 | rRNA | 24224 | 59696 |  |  |  |  | Prophage (P1031) |  |
| 53142 | 53142 | rRNA | 24224 | 59696 |  |  |  |  | Prophage (P1031) |  |
| 53142 | 53142 | CDS | 49920 | 53142 |  |  |  |  |  | Phage hyaluronidase |
| 1054409 | 1054409 | CDS | 1052773 | 1059254 | *zmpA* | INV10409960 | SPD_1018 | SP_1154 |  | IgA-protease |
| 1054409 | 1054409 | rRNA | 1052773 | 1117795 |  |  |  |  | Integrative and conjugative element. |  |
| 1054409 | 1054409 | rRNA | 1052773 | 1117795 |  |  |  |  | Integrative and conjugative element. |  |
| 1349189 | 1349189 | CDS | 1349139 | 1349809 |  | INV10412180 | SPD_1256 | SP_1427 |  | putative peptidase |
| 1099325 | 1099325 | rRNA | 1052773 | 1117795 |  |  |  |  | Integrative and conjugative element. |  |
| 1099325 | 1099325 | rRNA | 1052773 | 1117795 |  |  |  |  | Integrative and conjugative element. |  |
| 1099325 | 1099325 | CDS | 1098227 | 1104459 |  |  |  |  |  | SNF2 family protein |
