# Supplementary material for: Understanding pneumococcal serotype 1 biology through population genomic analysis
Source: BMC Infect Dis. 2016 Nov 8;16:649. doi: 10.1186/s12879-016-1987-z (PMC5100261; doi:10.1186/s12879-016-1987-z)
Supplement: Additional file 8: — Summary of the genes found in the genetic recombination regions in clade SC2-WA. Genes present in the regions with recombination events in each clade are summarised. (DOCX 124 kb) [file 12879_2016_1987_MOESM8_ESM.docx]

| **Recombination Start** | **Recombination End** | **Feature Type** | **Feature Start** | **Feature End** | **Gene** | **Locus Name** | **D39 Ortholog** | **TIGR4 Ortholog** | **Notes/Comments** | **Product Name** |
| --- | --- | --- | --- | --- | --- | --- | --- | --- | --- | --- |
| 1108676 | 1108676 | rRNA | 1052773 | 1117795 |  |  |  |  | Integrative and conjugative element. |  |
| 1108676 | 1108676 | rRNA | 1052773 | 1117795 |  |  |  |  | Integrative and conjugative element. |  |
| 1108676 | 1108676 | CDS | 1108282 | 1108761 |  |  |  |  |  | transcription regulator, probable |
| 483330 | 483330 | rRNA | 476902 | 485073 |  |  |  |  |  | mobile element protein + integrase + Type I restriction-modification system, DNA-methyltransferase subunit M |
| 483330 | 483330 | rRNA | 476902 | 485073 |  |  |  |  |  | mobile element protein + integrase + Type I restriction-modification system, DNA-methyltransferase subunit M |
| 483330 | 483330 | CDS | 483197 | 484661 |  |  |  |  |  | Type I restriction-modification system, DNA-methyltransferase subunit M (EC 2.1.1.72) |
| 1687751 | 1687751 | CDS | 1687364 | 1688141 | trpA | INV10415590 | SPD_1596 | SP_1811 |  | tryptophan synthase alpha chain |
| 154357 | 154357 | CDS | 153382 | 155338 | pspA | INV10400930 |  |  |  | Pneumococcal surface protein A |
| 1276220 | 1276220 | rRNA | 1258405 | 1278977 |  |  |  |  | Integrative and conjugative element remnant. |  |
| 1276220 | 1276220 | rRNA | 1258405 | 1278977 |  |  |  |  | Integrative and conjugative element remnant. |  |
| 51654 | 51654 | rRNA | 24224 | 59696 |  |  |  |  | Prophage (P1031) |  |
| 51654 | 51654 | rRNA | 24224 | 59696 |  |  |  |  | Prophage (P1031) |  |
| 51654 | 51654 | CDS | 49920 | 53142 |  |  |  |  |  | Phage hyaluronidase |
| 833718 | 833718 | CDS | 833551 | 834081 |  | INV10407660 |  |  |  | putative repressor protein (pseudogene) |
| 34178 | 34178 | rRNA | 24224 | 59696 |  |  |  |  | Prophage (P1031) |  |
| 34178 | 34178 | rRNA | 24224 | 59696 |  |  |  |  | Prophage (P1031) |  |
| 34178 | 34178 | CDS | 34004 | 34334 |  |  |  |  |  | Phage protein |
| 1275210 | 1275210 | rRNA | 1258405 | 1278977 |  |  |  |  | Integrative and conjugative element remnant. |  |
| 1275210 | 1275210 | rRNA | 1258405 | 1278977 |  |  |  |  | Integrative and conjugative element remnant. |  |
| 1275210 | 1275210 | CDS | 1275200 | 1275788 |  | INV10411440 | SPD_1180 | SP_1346 |  | putative membrane protein |
| 1089285 | 1089285 | rRNA | 1052773 | 1117795 |  |  |  |  | Integrative and conjugative element. |  |
| 1089285 | 1089285 | rRNA | 1052773 | 1117795 |  |  |  |  | Integrative and conjugative element. |  |
| 1089285 | 1089285 | CDS | 1088251 | 1090133 |  |  |  |  |  | FIG01116389: hypothetical protein |
| 1630414 | 1630414 | CDS | 1629356 | 1630421 |  | INV10414790 | SPD_1544 | SP_1734 |  | putative ribosomal RNA small subunit methyltransferase |
| 805194 | 805194 | CDS | 804942 | 806895 | fruA | INV10407250 | SPD_0773 | SP_0877 |  | putative fructose-specific phosphotransferase system (PTS), IIABC component |
| 638362 | 638362 | CDS | 638043 | 638667 |  | INV10405640 | SPD_0585 | SP_0673 |  | conserved hypothetical protein |
| 593689 | 593689 | CDS | 593659 | 600095 |  | INV10405360 | SPD_0558 | SP_0641 |  | putative surface-anchored serine protease |
| 932919 | 932919 | CDS | 932691 | 932964 |  |  |  |  |  | Ferrochelatase, protoheme ferro-lyase (EC 4.99.1.1) |
| 426614 | 426614 | CDS | 425867 | 426890 | ilvC | INV10403850 | SPD_0406 | SP_0447 |  | ketol-acid reductoisomerase |
| 896750 | 896750 | CDS | 896309 | 897509 |  |  |  |  |  | Multidrug resistance efflux pump PmrA |
| 1139810 | 1139810 | CDS | 1137415 | 1139950 | phpA | INV10410130 | SPD_1037 |  |  | putative streptococcal histidine triad protein PhpA |
| 1120161 | 1120161 | CDS | 1119485 | 1121036 |  | INV10409990 | SPD_1021 | SP_1157 |  | voltage gated chloride channel family protein |
| 8835 | 8835 | CDS | 8767 | 9136 |  | INV10400080 | SPD_0008 | SP_0008 |  | putative septum formation initiator protein |
| 783111 | 783111 | CDS | 782763 | 785199 | parC | INV10407060 | SPD_0748 | SP_0855 |  | topoisomerase IV subunit A |
| 1067113 | 1067113 | rRNA | 1052773 | 1117795 |  |  |  |  | Integrative and conjugative element. |  |
| 1067113 | 1067113 | rRNA | 1052773 | 1117795 |  |  |  |  | Integrative and conjugative element. |  |
| 1067113 | 1067113 | CDS | 1066095 | 1067454 |  |  |  |  |  | DNA-cytosine methyltransferase (EC 2.1.1.37) |
| 1274733 | 1274733 | rRNA | 1258405 | 1278977 |  |  |  |  | Integrative and conjugative element remnant. |  |
| 1274733 | 1274733 | rRNA | 1258405 | 1278977 |  |  |  |  | Integrative and conjugative element remnant. |  |
| 1274733 | 1274733 | CDS | 1274480 | 1275071 |  |  |  |  |  | FIG01114899: hypothetical protein |
| 1101845 | 1101845 | rRNA | 1052773 | 1117795 |  |  |  |  | Integrative and conjugative element. |  |
| 1101845 | 1101845 | rRNA | 1052773 | 1117795 |  |  |  |  | Integrative and conjugative element. |  |
| 1101845 | 1101845 | CDS | 1098227 | 1104459 |  |  |  |  |  | SNF2 family protein |
| 862202 | 862202 | CDS | 860820 | 862704 | cbpE | INV10407940 | SPD_0821 | SP_0930 |  | choline binding protein E |
| 1575395 | 1575395 | CDS | 1575331 | 1576660 |  | INV10414330 | SPD_1495 | SP_1683 |  | extracellular solute-binding lipoprotein |
| 1672579 | 1672579 | CDS | 1672539 | 1674021 |  | INV10415380 | SPD_1587 | SP_1800 |  | putative Mga-like regulatory protein |
| 926714 | 926714 | CDS | 925195 | 928315 | phtE | INV10408630 | SPD_0890 | SP_1004 |  | pneumococcal histidine triad protein E (Bvh-3) |
| 163471 | 163471 | CDS | 162998 | 163682 |  | INV10401020 | SPD_0134 | SP_0127 |  | glycoprotease family protein |
| 666315 | 666315 | CDS | 666002 | 666785 | thiM | INV10405960 | SPD_0623 | SP_0717 |  | hydroxyethylthiazole kinase |
| 1955524 | 1955524 | CDS | 1955335 | 1955635 |  | INV10418010 |  | SP_2089 |  | putative transposase fragment |
| 1955524 | 1955524 | rRNA | 1955335 | 1955635 |  |  |  |  | putative transposase fragment |  |
| 1621038 | 1621038 | CDS | 1620720 | 1621686 | scrR | INV10414700 | SPD_1535 | SP_1725 |  | putative sucrose operon repressor |
| 250098 | 250098 | CDS | 249438 | 251877 | pflD | INV10402120 | SPD_0235 | SP_0251 |  | putative formate acetyltransferase |
| 1273975 | 1273975 | rRNA | 1258405 | 1278977 |  |  |  |  | Integrative and conjugative element remnant. |  |
| 1273975 | 1273975 | rRNA | 1258405 | 1278977 |  |  |  |  | Integrative and conjugative element remnant. |  |
| 1273975 | 1273975 | CDS | 1273644 | 1274481 |  |  |  |  |  | hypothetical protein |
| 1274110 | 1274110 | rRNA | 1258405 | 1278977 |  |  |  |  | Integrative and conjugative element remnant. |  |
| 1274110 | 1274110 | rRNA | 1258405 | 1278977 |  |  |  |  | Integrative and conjugative element remnant. |  |
| 1274110 | 1274110 | CDS | 1273644 | 1274481 |  |  |  |  |  | hypothetical protein |
| 51569 | 51569 | rRNA | 24224 | 59696 |  |  |  |  | Prophage (P1031) |  |
| 51569 | 51569 | rRNA | 24224 | 59696 |  |  |  |  | Prophage (P1031) |  |
| 51569 | 51569 | CDS | 49920 | 53142 |  |  |  |  |  | Phage hyaluronidase |
| 34205 | 34205 | rRNA | 24224 | 59696 |  |  |  |  | Prophage (P1031) |  |
| 34205 | 34205 | rRNA | 24224 | 59696 |  |  |  |  | Prophage (P1031) |  |
| 34205 | 34205 | CDS | 34004 | 34334 |  |  |  |  |  | Phage protein |
| 51569 | 51569 | rRNA | 24224 | 59696 |  |  |  |  | Prophage (P1031) |  |
| 51569 | 51569 | rRNA | 24224 | 59696 |  |  |  |  | Prophage (P1031) |  |
| 51569 | 51569 | CDS | 49920 | 53142 |  |  |  |  |  | Phage hyaluronidase |
| 1274733 | 1274733 | rRNA | 1258405 | 1278977 |  |  |  |  | Integrative and conjugative element remnant. |  |
| 1274733 | 1274733 | rRNA | 1258405 | 1278977 |  |  |  |  | Integrative and conjugative element remnant. |  |
| 1274733 | 1274733 | CDS | 1274480 | 1275071 |  |  |  |  |  | FIG01114899: hypothetical protein |
| 1070455 | 1070455 | rRNA | 1052773 | 1117795 |  |  |  |  | Integrative and conjugative element. |  |
| 1070455 | 1070455 | rRNA | 1052773 | 1117795 |  |  |  |  | Integrative and conjugative element. |  |
| 1070455 | 1070455 | CDS | 1069272 | 1070490 |  |  |  |  |  | putative transposon integrase; Tn916 ORF3-like |
| 1065037 | 1065037 | rRNA | 1052773 | 1117795 |  |  |  |  | Integrative and conjugative element. |  |
| 1065037 | 1065037 | rRNA | 1052773 | 1117795 |  |  |  |  | Integrative and conjugative element. |  |
| 1075682 | 1075682 | rRNA | 1052773 | 1117795 |  |  |  |  | Integrative and conjugative element. |  |
| 1075682 | 1075682 | rRNA | 1052773 | 1117795 |  |  |  |  | Integrative and conjugative element. |  |
| 1075682 | 1075682 | CDS | 1075384 | 1076320 |  |  |  |  |  | FIG00628965: hypothetical protein |
| 1101848 | 1101848 | rRNA | 1052773 | 1117795 |  |  |  |  | Integrative and conjugative element. |  |
| 1101848 | 1101848 | rRNA | 1052773 | 1117795 |  |  |  |  | Integrative and conjugative element. |  |
| 1101848 | 1101848 | CDS | 1098227 | 1104459 |  |  |  |  |  | SNF2 family protein |
| 1073237 | 1073237 | rRNA | 1052773 | 1117795 |  |  |  |  | Integrative and conjugative element. |  |
| 1073237 | 1073237 | rRNA | 1052773 | 1117795 |  |  |  |  | Integrative and conjugative element. |  |
| 1073237 | 1073237 | CDS | 1073088 | 1075008 |  |  |  |  |  | Tetracycline resistance protein TetM |
| 1101848 | 1101848 | rRNA | 1052773 | 1117795 |  |  |  |  | Integrative and conjugative element. |  |
| 1101848 | 1101848 | rRNA | 1052773 | 1117795 |  |  |  |  | Integrative and conjugative element. |  |
| 1101848 | 1101848 | CDS | 1098227 | 1104459 |  |  |  |  |  | SNF2 family protein |
| 1108326 | 1108326 | rRNA | 1052773 | 1117795 |  |  |  |  | Integrative and conjugative element. |  |
| 1108326 | 1108326 | rRNA | 1052773 | 1117795 |  |  |  |  | Integrative and conjugative element. |  |
| 1108326 | 1108326 | CDS | 1108282 | 1108761 |  |  |  |  |  | transcription regulator, probable |
| 1065774 | 1065774 | rRNA | 1052773 | 1117795 |  |  |  |  | Integrative and conjugative element. |  |
| 1065774 | 1065774 | rRNA | 1052773 | 1117795 |  |  |  |  | Integrative and conjugative element. |  |
| 1065774 | 1065774 | CDS | 1065216 | 1065994 |  |  |  |  |  | FIG01114020: hypothetical protein |
| 1087214 | 1087214 | rRNA | 1052773 | 1117795 |  |  |  |  | Integrative and conjugative element. |  |
| 1087214 | 1087214 | rRNA | 1052773 | 1117795 |  |  |  |  | Integrative and conjugative element. |  |
| 1101890 | 1101890 | rRNA | 1052773 | 1117795 |  |  |  |  | Integrative and conjugative element. |  |
| 1101890 | 1101890 | rRNA | 1052773 | 1117795 |  |  |  |  | Integrative and conjugative element. |  |
| 1101890 | 1101890 | CDS | 1098227 | 1104459 |  |  |  |  |  | SNF2 family protein |
| 526978 | 526978 | CDS | 526349 | 527153 |  | INV10404770 | SPD_0496 | SP_0571 |  | cell filamentation protein fic-related protein |
| 1691517 | 1691517 | CDS | 1690684 | 1691689 | trpD | INV10415630 | SPD_1600 | SP_1815 |  | anthranilate phosphoribosyltransferase |
| 1599325 | 1599325 | CDS | 1598793 | 1601307 | secA | INV10414500 | SPD_1512 | SP_1702 |  | putative preprotein SecA subunit |
| 633290 | 633290 | CDS | 632576 | 633575 |  | INV10405580 |  |  |  | hypothetical protein |
| 154357 | 154357 | CDS | 153382 | 155338 | pspA | INV10400930 |  |  |  | Pneumococcal surface protein A |
| 1064854 | 1064854 | rRNA | 1052773 | 1117795 |  |  |  |  | Integrative and conjugative element. |  |
| 1064854 | 1064854 | rRNA | 1052773 | 1117795 |  |  |  |  | Integrative and conjugative element. |  |
| 1137734 | 1137734 | CDS | 1137415 | 1139950 | phpA | INV10410130 | SPD_1037 |  |  | putative streptococcal histidine triad protein PhpA |
| 659720 | 659720 | CDS | 659430 | 660108 |  | INV10405900 | SPD_0617 | SP_0710 |  | amino-acid ABC transporter integral membrane protein |
| 1557161 | 1557161 | CDS | 1557097 | 1557361 |  | INV10414140 | SPD_1476 | SP_1663 |  | putative membrane protein |
