# Supplementary material for: Understanding pneumococcal serotype 1 biology through population genomic analysis
Source: BMC Infect Dis. 2016 Nov 8;16:649. doi: 10.1186/s12879-016-1987-z (PMC5100261; doi:10.1186/s12879-016-1987-z)
Supplement: Additional file 9: — Summary of the genes found in the genetic recombination regions in clade SC3-SEA. Genes present in the regions with recombination events in each clade are summarised. (DOCX 133 kb) [file 12879_2016_1987_MOESM9_ESM.docx]

| **Recombination Start** | **Recombination End** | **Feature Type** | **Feature Start** | **Feature End** | **Gene** | **Locus Name** | **D39 Ortholog** | **TIGR4 Ortholog** | **Notes/Comments** | **Product Name** |
| --- | --- | --- | --- | --- | --- | --- | --- | --- | --- | --- |
| 1276422 | 1276422 | rRNA | 1258405 | 1278977 |  |  |  |  | Integrative and conjugative element remnant. |  |
| 1276422 | 1276422 | rRNA | 1258405 | 1278977 |  |  |  |  | Integrative and conjugative element remnant. |  |
| 1276422 | 1276422 | CDS | 1276409 | 1276859 |  | INV10411470 | SPD_1183 | SP_1349 |  | putative uncharacterized protein |
| 1067199 | 1067199 | rRNA | 1052773 | 1117795 |  |  |  |  | Integrative and conjugative element. |  |
| 1067199 | 1067199 | rRNA | 1052773 | 1117795 |  |  |  |  | Integrative and conjugative element. |  |
| 1067199 | 1067199 | CDS | 1066095 | 1067454 |  |  |  |  |  | DNA-cytosine methyltransferase (EC 2.1.1.37) |
| 1553027 | 1553027 | CDS | 1552378 | 1555171 | *ileS* | INV10414110 | SPD_1472 | SP_1659 |  | isoleucyl-tRNA synthetase |
| 1375663 | 1375663 | CDS | 1375559 | 1375784 |  | INV10412410 | SPD_1288 | SP_1459 |  | putative membrane protein |
| 355450 | 355450 | CDS | 354766 | 356749 | *aliA* | INV10403150 | SPD_0334 | SP_0366 |  | putative extracellular oligopeptide-binding protein |
| 1350495 | 1350495 | CDS | 1350446 | 1351733 |  | INV10412190 | SPD_1258 | SP_1429 |  | putative peptidase |
| 1202033 | 1202033 | CDS | 1201732 | 1202527 |  | INV10410720 | SPD_1103 | SP_1246 |  | putative haloacid dehalogenase-like hydrolase |
| 1500520 | 1500520 | CDS | 1500129 | 1500759 |  | INV10413680 | SPD_1426 | SP_1601 |  | putative membrane protein |
| 1460890 | 1460890 | CDS | 1460070 | 1461255 |  | INV10413260 | SPD_1384 | SP_1552 |  | cation efflux family protein |
| 206242 | 206242 | CDS | 206107 | 208939 | *uvrA* | INV10401530 | SPD_0176 | SP_0186 |  | UvrABC system protein A (UvrA protein) |
| 1276029 | 1276029 | rRNA | 1258405 | 1278977 |  |  |  |  | Integrative and conjugative element remnant. |  |
| 1276029 | 1276029 | rRNA | 1258405 | 1278977 |  |  |  |  | Integrative and conjugative element remnant. |  |
| 1567926 | 1567926 | CDS | 1566149 | 1568192 | *penA* | INV10414240 | SPD_1486 | SP_1673 |  | penicillin-binding protein 2b |
| 1691255 | 1691255 | CDS | 1690684 | 1691689 | *trpD* | INV10415630 | SPD_1600 | SP_1815 |  | anthranilate phosphoribosyltransferase |
| 1541481 | 1541481 | CDS | 1540986 | 1541709 | *mtsB* | INV10414010 | SPD_1461 | SP_1648 |  | metal cation ABC transporter ATP-binding protein |
| 1350495 | 1350495 | CDS | 1350446 | 1351733 |  | INV10412190 | SPD_1258 | SP_1429 |  | putative peptidase |
| 1449822 | 1449822 | CDS | 1448555 | 1449899 | *asnS* | INV10413140 | SPD_1371 | SP_1542 |  | asparaginyl-tRNA synthetase |
| 360176 | 360176 | CDS | 357049 | 362353 |  | INV10403160 | SPD_0335 | SP_0368 |  | cell wall surface anchored protein |
| 1276029 | 1276029 | rRNA | 1258405 | 1278977 |  |  |  |  | Integrative and conjugative element remnant. |  |
| 1276029 | 1276029 | rRNA | 1258405 | 1278977 |  |  |  |  | Integrative and conjugative element remnant. |  |
| 1350495 | 1350495 | CDS | 1350446 | 1351733 |  | INV10412190 | SPD_1258 | SP_1429 |  | putative peptidase |
| 1119684 | 1119684 | CDS | 1119485 | 1121036 |  | INV10409990 | SPD_1021 | SP_1157 |  | voltage gated chloride channel family protein |
| 962973 | 962973 | rRNA | 948567 | 979552 |  |  |  |  | GC-poor region with Tn-Element (P1031) |  |
| 962973 | 962973 | rRNA | 948567 | 979552 |  |  |  |  | GC-poor region with Tn-Element (P1031) |  |
| 962973 | 962973 | CDS | 961741 | 963481 |  | INV10409100 | SPD_0927 |  |  | alpha-amylase (pseudogene) |
| 962973 | 962973 | CDS | 962072 | 969737 |  | INV10409110 |  |  |  | Tn5252 relaxase |
| 1139620 | 1139620 | CDS | 1137415 | 1139950 | *phpA* | INV10410130 | SPD_1037 |  |  | putative streptococcal histidine triad protein PhpA |
| 1067888 | 1067888 | rRNA | 1052773 | 1117795 |  |  |  |  | Integrative and conjugative element. |  |
| 1067888 | 1067888 | rRNA | 1052773 | 1117795 |  |  |  |  | Integrative and conjugative element. |  |
| 333846 | 333846 | CDS | 333456 | 335767 | *tnp* | INV10402960 |  |  |  | putative IS630-Spn1 transposase (pseudogene) |
| 333846 | 333846 | rRNA | 333456 | 354568 |  |  |  |  | GC poor region, enclosed by transposases |  |
| 333846 | 333846 | rRNA | 333456 | 354568 |  |  |  |  | GC poor region, enclosed by transposases |  |
| 333846 | 333846 | CDS | 333604 | 334912 | *tnp* | INV10402970 |  |  |  | putative IS1167 transposase (pseudogene) |
| 333846 | 333846 | CDS | 333604 | 334949 |  | INV10402900 |  |  |  | IS1167 transposase (pseudogene) |
| 1275573 | 1275573 | rRNA | 1258405 | 1278977 |  |  |  |  | Integrative and conjugative element remnant. |  |
| 1275573 | 1275573 | rRNA | 1258405 | 1278977 |  |  |  |  | Integrative and conjugative element remnant. |  |
| 1275573 | 1275573 | CDS | 1275200 | 1275788 |  | INV10411440 | SPD_1180 | SP_1346 |  | putative membrane protein |
| 154357 | 154357 | CDS | 153382 | 155338 | *pspA* | INV10400930 |  |  |  | Pneumococcal surface protein A |
| 1642813 | 1642813 | CDS | 1642542 | 1643043 |  | INV10414880 | SPD_1555 | SP_1745 |  | putative isochorismatase |
| 1991516 | 1991516 | CDS | 1991297 | 1992528 |  |  |  |  |  | Macrolide-efflux protein |
| 687141 | 687141 | CDS | 686744 | 687590 |  | INV10406150 | SPD_0646 | SP_0742 |  | putative fatty-acid binding protein |
| 95421 | 95421 | CDS | 94962 | 98901 | *strH* | INV10400480 | SPD_0063 | SP_0057 |  | beta-N-acetylhexosaminidase precursor (ec 3.2.1.52) (sortase-sorted) |
| 649609 | 649609 | CDS | 648923 | 650274 | *murD* | INV10405760 | SPD_0598 | SP_0688 |  | UDP-N-acetylmuramoylalanine--D-glutamate ligase |
| 1276029 | 1276029 | rRNA | 1258405 | 1278977 |  |  |  |  | Integrative and conjugative element remnant. |  |
| 1276029 | 1276029 | rRNA | 1258405 | 1278977 |  |  |  |  | Integrative and conjugative element remnant. |  |
| 497063 | 497063 | CDS | 496155 | 497205 |  | INV10404400 | SPD_0465 | SP_0523 |  | ABC transporter protein EcsB |
| 856667 | 856667 | CDS | 856305 | 857115 |  | INV10407890 | SPD_0816 | SP_0923 |  | haloacid dehalogenase-like hydrolase |
| 967961 | 967961 | rRNA | 948567 | 979552 |  |  |  |  | GC-poor region with Tn-Element (P1031) |  |
| 967961 | 967961 | rRNA | 948567 | 979552 |  |  |  |  | GC-poor region with Tn-Element (P1031) |  |
| 967961 | 967961 | CDS | 962072 | 969737 |  | INV10409110 |  |  |  | Tn5252 relaxase |
| 967961 | 967961 | CDS | 967895 | 968255 |  |  |  |  |  | Tn5252, Orf 10 protein |
| 1461651 | 1461651 | CDS | 1461397 | 1463269 |  | INV10413270 | SPD_1385 | SP_1553 |  | ABC transporter ATP-binding protein |
| 1440505 | 1440505 | CDS | 1440178 | 1441624 | *murE* | INV10413020 | SPD_1359 | SP_1530 |  | UDP-N-acetylmuramoylalanyl-D-glutamate--2,6-dia minopimelate ligase |
| 778873 | 778873 | rRNA | 778203 | 779542 |  |  |  |  | transposon |  |
| 778873 | 778873 | CDS | 778718 | 778992 |  |  |  |  |  | IS861, transposase (orf2), IS3 family, truncated |
| 1275666 | 1275666 | rRNA | 1258405 | 1278977 |  |  |  |  | Integrative and conjugative element remnant. |  |
| 1275666 | 1275666 | rRNA | 1258405 | 1278977 |  |  |  |  | Integrative and conjugative element remnant. |  |
| 1275666 | 1275666 | CDS | 1275200 | 1275788 |  | INV10411440 | SPD_1180 | SP_1346 |  | putative membrane protein |
| 820992 | 820992 | CDS | 820068 | 823419 |  | INV10407370 | SPD_0784 | SP_0892 |  | putative type I restriction modification system restriction protein |
| 1067094 | 1067094 | rRNA | 1052773 | 1117795 |  |  |  |  | Integrative and conjugative element. |  |
| 1067094 | 1067094 | rRNA | 1052773 | 1117795 |  |  |  |  | Integrative and conjugative element. |  |
| 1067094 | 1067094 | CDS | 1066095 | 1067454 |  |  |  |  |  | DNA-cytosine methyltransferase (EC 2.1.1.37) |
| 2062904 | 2062904 | CDS | 2062099 | 2063851 | *cbpA* | INV10418920 | SPD_2017 | SP_2190 |  | choline-binding surface protein A |
