# Supplementary material for: Understanding pneumococcal serotype 1 biology through population genomic analysis
Source: BMC Infect Dis. 2016 Nov 8;16:649. doi: 10.1186/s12879-016-1987-z (PMC5100261; doi:10.1186/s12879-016-1987-z)
Supplement: Additional file 10: — Intragenic number of observed and expected numbers of SNPs in the ST217 isolates. All the genes in the ST217 isolates, sizes, observed (OSNP) and expected (ESNP) number of SNPs, ratio OSNP to ESNP and products of each gene are summarised. (DOCX 315 kb) [file 12879_2016_1987_MOESM10_ESM.docx]

| **Genomic Feature** | **Length (bp)** | **Length (bp)** | **Length (bp)** | **(Observed+1)/(Expected+1)** | **Log_2_((Observed+1)/(Expected+1))** | **Feature Product** |
| --- | --- | --- | --- | --- | --- | --- |
| LargeSubunitRibosomalRNAlsuRNALSUrRNA | 2903 | 0 | 33.75 | 0.03 | -5.12 | Putative Membrane Protein Putative |
| spxB | 1776 | 0 | 20.65 | 0.05 | -4.44 | 50S Ribosomal Protein L650S |
| alsS | 1701 | 0 | 19.78 | 0.05 | -4.38 | Acetolactate Synthase Large Subunit Acetolactate |
| 1728377_1730066 | 1689 | 0 | 19.64 | 0.05 | -4.37 |  |
| INV10403820 | 1635 | 0 | 19.01 | 0.05 | -4.32 | Heparinase II/III-Like Proteinheparinase |
| INV10404050 | 1428 | 0 | 16.6 | 0.06 | -4.14 |  |
| FIG01118148-hypotheticalprotein | 1410 | 0 | 16.39 | 0.06 | -4.12 | FIG01116089: Hypothetical Protein Fig01116089: |
| SPn1Transposase | 1354 | 0 | 15.74 | 0.06 | -4.07 | 50S Ribosomal Protein L450S |
| vicK | 1350 | 0 | 15.7 | 0.06 | -4.06 | Tn916, Transcriptional Regulator, Putative Tn916, |
| 1433608_1434955 | 1347 | 0 | 15.66 | 0.06 | -4.06 |  |
| INV10407400 | 1347 | 0 | 15.66 | 0.06 | -4.06 | Conserved Hypothetical Proteinconserved |
| xseA | 1341 | 0 | 15.59 | 0.06 | -4.05 | Transcriptional Regulator, XRE Family Transcriptional |
| INV10404700 | 1335 | 0 | 15.52 | 0.06 | -4.05 | Putative Uncharacterized Protein Putative |
| wzy | 1305 | 0 | 15.17 | 0.06 | -4.02 | Transcriptional Regulator, Arsr Family Transcriptional |
| malC | 1293 | 0 | 15.03 | 0.06 | -4 | IS1381, Transposase Orfais1381, |
| hom | 1287 | 0 | 14.96 | 0.06 | -4 | Grpe Protein (HSP-70 Cofactor) Grpe |
| 2083564_2084815 | 1251 | 0 | 14.54 | 0.06 | -3.96 |  |
| INV10419260 | 1251 | 0 | 14.54 | 0.06 | -3.96 | Putative Exported Glycosyl Hydrolase Putative |
| INV10400800 | 1167 | 0 | 13.57 | 0.07 | -3.86 | Putative Membrane Protein Putative |
| INV10411620 | 1158 | 0 | 13.46 | 0.07 | -3.85 | Putative Uncharacterized Protein Putative |
| wchB | 1137 | 0 | 13.22 | 0.07 | -3.83 | Putative IS1167 Transposase Putative |
| mutS2 | 2324 | 1 | 27.02 | 0.07 | -3.81 | Putative Maltodextrose Utilization Protein Mala Putative |
| INV10412980 | 1095 | 0 | 12.73 | 0.07 | -3.78 | Putative Membrane Protein Putative |
| INV10407260 | 2226 | 1 | 25.88 | 0.07 | -3.75 | Putative Oxidoreductase Putative |
| aroB | 1068 | 0 | 12.42 | 0.07 | -3.75 | 3-Phosphoshikimate 1-Carboxyvinyltransferase3-Phosphoshikimate |
| INV10401540 | 1062 | 0 | 12.35 | 0.07 | -3.74 | Conserved Hypothetical Proteinconserved |
| INV10417710 | 1059 | 0 | 12.31 | 0.08 | -3.73 | Tatd Related Dnasetatd |
| INV10400680 | 1053 | 0 | 12.24 | 0.08 | -3.73 | Putative ATP-Binding PP-Loop Family Protein Putative |
| INV10416320 | 1044 | 0 | 12.14 | 0.08 | -3.72 | ABC Transporter, ATP-Binding/Permease Protein ABC |
| tsf | 1041 | 0 | 12.1 | 0.08 | -3.71 | Tetracycline Resistance Protein Tetm Tetracycline |
| UDP-glucose4-epimerase(EC5 | 1029 | 0 | 11.96 | 0.08 | -3.7 | Putative Thiamin Pyrophosphokinase Putative |
| gpsA | 1017 | 0 | 11.82 | 0.08 | -3.68 | Glutamate Racemaseglutamate |
| fni | 1011 | 0 | 11.75 | 0.08 | -3.67 | FIG01117752: Hypothetical Protein Fig01117752: |
| 1419440_1420449 | 1009 | 0 | 11.73 | 0.08 | -3.67 |  |
| INV10412880 | 1009 | 0 | 11.73 | 0.08 | -3.67 | Putative Membrane Protein Putative |
| IS1380-Spn1_transposase | 1008 | 0 | 11.72 | 0.08 | -3.67 | Putative Membrane Protein Putative |
| penA | 2043 | 1 | 23.75 | 0.08 | -3.63 | DNA Mismatch Repair Protein Muts Dna |
| HollidayjunctionDNAhelicaseRuvB | 972 | 0 | 11.3 | 0.08 | -3.62 | 10 Kda Chaperonin10 |
| prsA1 | 969 | 0 | 11.27 | 0.08 | -3.62 | Peptidase Tpeptidase |
| glkA | 960 | 0 | 11.16 | 0.08 | -3.6 | Glutamyl-Trna Amidotransferase Subunit Aglutamyl-Trna |
| INV10417690 | 942 | 0 | 10.95 | 0.08 | -3.58 | Putative DNA-Binding Protein Putative |
| pyrB | 924 | 0 | 10.74 | 0.09 | -3.55 | Putative Alkaline Phosphatase Synthesis Sensor Protein Putative |
| INV10411930 | 918 | 0 | 10.67 | 0.09 | -3.54 | Conserved Hypothetical Protein Conserved |
| rpmA | 918 | 0 | 10.67 | 0.09 | -3.54 | Phosphoribosylaminoimidazole-Succinocarboxamide Synthasephosphoribosylaminoimidazole-Succinocarboxamide |
| INV10400770 | 912 | 0 | 10.6 | 0.09 | -3.54 | Putative Membrane Protein (Pseudogene) Putative |
| INV10409640 | 1902 | 1 | 22.11 | 0.09 | -3.53 | Putative Uncharacterized Protein Putative |
| uvrA | 2832 | 2 | 32.92 | 0.09 | -3.5 | Tn5252, Orf 10 Proteintn5252, |
| fba | 882 | 0 | 10.25 | 0.09 | -3.49 | Enolaseenolase |
| mvaK1 | 879 | 0 | 10.22 | 0.09 | -3.49 | Putative Mannose-Specific Phosphotransferase System (PTS), IIAB Component Putative |
| metF | 867 | 0 | 10.08 | 0.09 | -3.47 | Galactose-6-Phosphate Isomerase Lacb Subunit 1galactose-6-Phosphate |
| INV10417730 | 1818 | 1 | 21.14 | 0.09 | -3.47 | Putative Aminotransferase Putative |
| INV10400140 | 865 | 0 | 10.06 | 0.09 | -3.47 | Hypothetical Proteinhypothetical |
| INV10417520 | 864 | 0 | 10.04 | 0.09 | -3.46 | Putative Membrane Protein Putative |
| INV10417750 | 858 | 0 | 9.98 | 0.09 | -3.46 | Putative Membrane Protein Putative |
| INV10404070 | 849 | 0 | 9.87 | 0.09 | -3.44 | Putative Membrane Protein Putative |
| INV10409610 | 849 | 0 | 9.87 | 0.09 | -3.44 | Conserved Hypothetical Proteinconserved |
| INV10418140 | 849 | 0 | 9.87 | 0.09 | -3.44 | Conserved Hypothetical Proteinconserved |
| malD | 843 | 0 | 9.8 | 0.09 | -3.43 | IS1381, Transposase Orfbis1381, |
| INV10419210 | 840 | 0 | 9.77 | 0.09 | -3.43 | Putative Petidase Putative |
| CysteineABCtransporter_substrate-bindingprotein | 831 | 0 | 9.66 | 0.09 | -3.41 | Transcriptional Regulatortranscriptional |
| INV10415870 | 828 | 0 | 9.63 | 0.09 | -3.41 | ABC Transporterabc |
| INV10419240 | 822 | 0 | 9.56 | 0.09 | -3.4 | Putative Membrane Protein Putative |
| INV10411130 | 807 | 0 | 9.38 | 0.1 | -3.38 | Ion Channel Transport Proteinion |
| INV10415980 | 807 | 0 | 9.38 | 0.1 | -3.38 | Conserved Hypothetical Proteinconserved |
| Integraserecombinase(XerCCodVfamily) | 798 | 0 | 9.28 | 0.1 | -3.36 | Hypothetical Phage Proteinhypothetical |
| INV10417500 | 1674 | 1 | 19.46 | 0.1 | -3.35 | Putative Uncharacterized Protein Putative |
| fhs1 | 1671 | 1 | 19.43 | 0.1 | -3.35 | Putative Putative Malonyl Coa-Acyl Carrier Protein Transacylase Putative |
| rplB | 789 | 0 | 9.17 | 0.1 | -3.35 | Protein Of Unknown Function DUF208Protein |
| mutS | 2535 | 2 | 29.47 | 0.1 | -3.34 | Macrolide-Efflux Protein Macrolide-Efflux |
| INV10405330 | 786 | 0 | 9.14 | 0.1 | -3.34 | Beta-Glucoside-Specific Phosphotransferase System (PTS), IIABC Componentbeta-Glucoside-Specific |
| rpsB | 780 | 0 | 9.07 | 0.1 | -3.33 | Putative Dihydroorotase Putative |
| 20393_22039 | 1646 | 1 | 19.14 | 0.1 | -3.33 |  |
| trpA | 777 | 0 | 9.03 | 0.1 | -3.33 | Pyruvate Oxidasepyruvate |
| INV10417060 | 774 | 0 | 9 | 0.1 | -3.32 | Putative PAP2 Superfamily Membrane Protein Putative |
| gyrA | 2469 | 2 | 28.7 | 0.1 | -3.31 | Guanylate Kinaseguanylate |
| INV10419000 | 759 | 0 | 8.82 | 0.1 | -3.3 | Putative Sugar-Specific Permease, Sgat/Ulaa Family Putative |
| glnQ1 | 741 | 0 | 8.61 | 0.1 | -3.26 | Glutamyl-Trna Amidotransferase Subunit Cglutamyl-Trna |
| INV10403450 | 2367 | 2 | 27.52 | 0.11 | -3.25 | Putative Membrane Protein Putative |
| purH | 1548 | 1 | 18 | 0.11 | -3.25 | Phage Proteinphage |
| INV10418980 | 729 | 0 | 8.48 | 0.11 | -3.24 | Putative Transketolase Subunit Putative |
| INV10401630 | 1533 | 1 | 17.82 | 0.11 | -3.23 | Putative Transposase Putative |
| malQ | 1518 | 1 | 17.65 | 0.11 | -3.22 | IS1381, Transposase Orfbis1381, |
| TrsE-likeprotein | 2316 | 2 | 26.93 | 0.11 | -3.22 | DNA-3-Methyladenine Glycosylase IDNA-3-Methyladenine |
| ccdA | 711 | 0 | 8.27 | 0.11 | -3.21 | Choline Binding Protein Jcholine |
| phoP | 708 | 0 | 8.23 | 0.11 | -3.21 | Sialidase B Precursor (Neuraminidase B)Sialidase |
| INV10417510 | 705 | 0 | 8.2 | 0.11 | -3.2 | Putative Transport Protein Putative |
| INV10400670 | 699 | 0 | 8.13 | 0.11 | -3.19 | Putative Exported Protein Putative |
| guaB | 1479 | 1 | 17.19 | 0.11 | -3.19 | Glycyl-Trna Synthetase Beta Chainglycyl-Trna |
| ftsE | 693 | 0 | 8.06 | 0.11 | -3.18 | Peptide Deformylasepeptide |
| gpmA | 693 | 0 | 8.06 | 0.11 | -3.18 | Alpha-Glycerophosphate Oxidasealpha-Glycerophosphate |
| wzd | 693 | 0 | 8.06 | 0.11 | -3.18 | Triosephosphate Isomerasetriosephosphate |
| gatA | 1467 | 1 | 17.06 | 0.11 | -3.17 | Putative Cell Division Protein Putative |
| pncO | 690 | 0 | 8.02 | 0.11 | -3.17 | Oligopeptide Transport System Permease Protein Oppb (TC 3.A.1.5.1)Oligopeptide |
| gatB | 1443 | 1 | 16.78 | 0.11 | -3.15 | Putative Signal Recognition Particle 54 Ftsy Putative |
| sdhB | 672 | 0 | 7.81 | 0.11 | -3.14 | Ribosomal Large Subunit Pseudouridine Synthase Bribosomal |
| INV10417530 | 663 | 0 | 7.71 | 0.11 | -3.12 | Putative Exported Protein Putative |
| INV10418950 | 654 | 0 | 7.6 | 0.12 | -3.1 | Putative Membrane Protein Putative |
| INV10417760 | 645 | 0 | 7.5 | 0.12 | -3.09 | Putative Universal Stress Protein Putative |
| INV10412760 | 642 | 0 | 7.46 | 0.12 | -3.08 | Putative ABC Transporter Putative |
| tmk | 639 | 0 | 7.43 | 0.12 | -3.08 | 50S Ribosomal Protein L3550S |
| ribE | 636 | 0 | 7.39 | 0.12 | -3.07 | Spermidine/Putrescine Extracellular Binding Protein Spermidine/Putrescine |
| 1134692_1136046 | 1354 | 1 | 15.74 | 0.12 | -3.07 |  |
| INV10412750 | 630 | 0 | 7.32 | 0.12 | -3.06 | Putative Membrane Protein Putative |
| INV10406330 | 1344 | 1 | 15.63 | 0.12 | -3.06 | Putative Uncharacterized Protein Putative |
| INV10418940 | 1341 | 1 | 15.59 | 0.12 | -3.05 | Putative Ribosomal RNA Large Subunit Methyltransferase A Putative |
| rplC | 627 | 0 | 7.29 | 0.12 | -3.05 | Ribose-Phosphate Pyrophosphokinase 1ribose-Phosphate |
| rpsC | 627 | 0 | 7.29 | 0.12 | -3.05 | Putative Dihydroorotate Dehydrogenase Putative |
| murC | 1335 | 1 | 15.52 | 0.12 | -3.05 | Lipoprotein, NLP/P60 Familylipoprotein, |
| INV10400250 | 624 | 0 | 7.25 | 0.12 | -3.04 | Hypothetical Proteinhypothetical |
| USGprotein | 2748 | 3 | 31.95 | 0.12 | -3.04 | Tn5252, Orf 10 Proteintn5252, |
| INV10405030 | 1329 | 1 | 15.45 | 0.12 | -3.04 | Putative HIT-Family Nucleotide-Binding Protein Putative |
| INV10417800 | 621 | 0 | 7.22 | 0.12 | -3.04 | Puttaive Ftsx-Family Transport Proteinputtaive |
| INV10419380 | 1326 | 1 | 15.42 | 0.12 | -3.04 | Marr-Family Regulatory Protein Marr-Family |
| INV10405100 | 2025 | 2 | 23.54 | 0.12 | -3.03 | Conserved Hypothetical Proteinconserved |
| INV10415360 | 1314 | 1 | 15.28 | 0.12 | -3.03 | ABC Transporter, ATP-Binding Protein ABC |
| speA | 1311 | 1 | 15.24 | 0.12 | -3.02 | 50S Ribosomal Protein L150S |
| cbpG | 611 | 0 | 7.1 | 0.12 | -3.02 | Choline Binding Protein Dcholine |
| eno | 1305 | 1 | 15.17 | 0.12 | -3.02 | DNA Polymerase III Alpha Subunit (EC 2.7.7.7); DNA Polymerase III Epsilon Chain (EC 2.7.7.7)DNA |
| INV10411950 | 1305 | 1 | 15.17 | 0.12 | -3.02 | Putative Polysaccharide Biosynthesis Flippase Putative |
| INV10410100 | 603 | 0 | 7.01 | 0.12 | -3 | Conserved Hypothetical Proteinconserved |
| stk1 | 1980 | 2 | 23.02 | 0.12 | -3 | 50S Ribosomal Protein L1350S |
| tdk | 600 | 0 | 6.98 | 0.13 | -3 | 50S Ribosomal Protein L1950S |
| INV10419250 | 1286 | 1 | 14.95 | 0.13 | -3 | Probable Alcohol Dehydrogenaseprobable |
| pyrP | 1284 | 1 | 14.93 | 0.13 | -2.99 | Plasmid Addiction System Poison Protein Plasmid |
| tig | 1284 | 1 | 14.93 | 0.13 | -2.99 | 50S Ribosomal Protein L33 150S |
| recR | 597 | 0 | 6.94 | 0.13 | -2.99 | Polyribonucleotide Nucleotidyltransferasepolyribonucleotide |
| INV10404990 | 1278 | 1 | 14.86 | 0.13 | -2.99 | Type I Restriction-Modification System Protein Type |
| INV10411640 | 1275 | 1 | 14.82 | 0.13 | -2.98 | Putative Conserved Hypothetical Protein (Pseudogene) Putative |
| serS | 1275 | 1 | 14.82 | 0.13 | -2.98 | Dtdp-4-Keto-L-Rhamnose Reductase Rmlddtdp-4-Keto-L-Rhamnose |
| INV10400780 | 594 | 0 | 6.91 | 0.13 | -2.98 | Putative Uncharacterized Protein (Pseudogene) Putative |
| thrS | 1944 | 2 | 22.6 | 0.13 | -2.98 | 50S Ribosomal Protein L3150S |
| IntramembraneproteaseRasPYluC_implicatedincelldivisionbasedonFtsLcleavage | 1260 | 1 | 14.65 | 0.13 | -2.97 | Hypothetical Phage Protein Hypothetical |
| cinA | 1257 | 1 | 14.61 | 0.13 | -2.96 | Sensor Histidine Kinase Sensor |
| Phagemajortailprotein | 579 | 0 | 6.73 | 0.13 | -2.95 | N-Ethylammeline Chlorohydrolasen-Ethylammeline |
| INV10409050 | 567 | 0 | 6.59 | 0.13 | -2.92 | Conserved Hypothetical Protein Conserved |
| INV10410590 | 564 | 0 | 6.56 | 0.13 | -2.92 | Transposase, Orf 1Transposase, |
| INV10411060 | 561 | 0 | 6.52 | 0.13 | -2.91 | Hemolysin-Like Protein Hemolysin-Like |
| Phageportalprotein | 1203 | 1 | 13.99 | 0.13 | -2.91 | N-Ethylammeline Chlorohydrolasen-Ethylammeline |
| pgk | 1197 | 1 | 13.92 | 0.13 | -2.9 | N-Ethylammeline Chlorohydrolasen-Ethylammeline |
| tufA | 1197 | 1 | 13.92 | 0.13 | -2.9 | Queuine Trna-Ribosyltransferasequeuine |
| sms | 1194 | 1 | 13.88 | 0.13 | -2.9 | Ribonuclease P Protein Componentribonuclease |
| coaC | 552 | 0 | 6.42 | 0.13 | -2.89 | Cytidylate Kinasecytidylate |
| INV10419170 | 1179 | 1 | 13.71 | 0.14 | -2.88 | Putative Alpha-1,2-Mannosidase Putative |
| purN | 546 | 0 | 6.35 | 0.14 | -2.88 | Phage Terminase, Small Subunit Phage |
| clpC | 2433 | 3 | 28.29 | 0.14 | -2.87 | Response Regulator Protein Response |
| INV10411740 | 1173 | 1 | 13.64 | 0.14 | -2.87 | Putative Membrane Protein Putative |
| prophageLambdaSa2_site-specificrecombinase_phageintegrasefamily | 543 | 0 | 6.31 | 0.14 | -2.87 | Putative Endopeptidase O Putative |
| rplE | 543 | 0 | 6.31 | 0.14 | -2.87 | Pneumococcal Surface Protein Apneumococcal |
| INV10412780 | 1167 | 1 | 13.57 | 0.14 | -2.86 | Putative Transposase (Pseudogene) Putative |
| recA | 1167 | 1 | 13.57 | 0.14 | -2.86 | Mannose-6-Phosphate Isomerasemannose-6-Phosphate |
| atpH | 537 | 0 | 6.24 | 0.14 | -2.86 | ATP Synthase B Chainatp |
| rplF | 537 | 0 | 6.24 | 0.14 | -2.86 | Phosphate Import ATP-Binding Protein 1phosphate |
| wchD | 1155 | 1 | 13.43 | 0.14 | -2.85 | DNA Topoisomerase IDNA |
| INV10416420 | 534 | 0 | 6.21 | 0.14 | -2.85 | Type II Restriction Enzymetype |
| INV10406020 | 525 | 0 | 6.1 | 0.14 | -2.83 | Putative Rrna Methylase Putative |
| pyrR | 522 | 0 | 6.07 | 0.14 | -2.82 | Pleiotropic Regulator Of Exopolysaccharide Synthesis, Competence And Biofilm Formation Ftr, XRE Familypleiotropic |
| infB | 1731 | 2 | 20.12 | 0.14 | -2.82 | Heat-Inducible Transcription Repressor Hrcaheat-Inducible |
| INV10400200 | 516 | 0 | 6 | 0.14 | -2.81 | Hypothetical Protein Hypothetical |
| INV10400040 | 1116 | 1 | 12.97 | 0.14 | -2.8 | Hypothetical Protein Hypothetical |
| INV10405050 | 1114 | 1 | 12.95 | 0.14 | -2.8 | ABC Transporter ATP-Binding Protein ABC |
| INV10400970 | 1680 | 2 | 19.53 | 0.15 | -2.77 | Sugar Phosphotransferase System (PTS), Mannose/Fructose/Sorbose Family, IID Component Sugar |
| LSUribosomalproteinL10p(P0) | 501 | 0 | 5.82 | 0.15 | -2.77 | Putative Response Regulator Protein Putative |
| carA | 1080 | 1 | 12.56 | 0.15 | -2.76 | Cadmium Resistance Protein Cadmium |
| INV10405110 | 1662 | 2 | 19.32 | 0.15 | -2.76 | Conserved Hypothetical Protein Conserved |
| INV10402030 | 1079 | 1 | 12.54 | 0.15 | -2.76 | Putative Azlc-Family Transport Protein Putative |
| atpF | 495 | 0 | 5.75 | 0.15 | -2.75 | ATP Synthase Beta Chain Atp |
| rpsE | 495 | 0 | 5.75 | 0.15 | -2.75 | Orotate Phosphoribosyltransferaseorotate |
| INV10417540 | 486 | 0 | 5.65 | 0.15 | -2.73 | Putative Methylase Putative |
| Competence-specificsigmafactorComX | 481 | 0 | 5.59 | 0.15 | -2.72 | Putative Late Competence Protein Putative |
| INV10419400 | 480 | 0 | 5.58 | 0.15 | -2.72 | Putative Membrane Protein Putative |
| ddlA | 1044 | 1 | 12.14 | 0.15 | -2.72 | Cytidine Deaminase (EC 3.5.4.5) Cytidine |
| wzx | 1041 | 1 | 12.1 | 0.15 | -2.71 | Transcriptional Regulator Transcriptional |
| ilvH | 477 | 0 | 5.55 | 0.15 | -2.71 | Putative DNA Polymerase III, Delta' Subunit Putative |
| INV10410670 | 2166 | 3 | 25.18 | 0.15 | -2.71 | Putative DNA-Binding Protein Putative |
| INV10415940 | 1038 | 1 | 12.07 | 0.15 | -2.71 | Putative Mga-Like Regulatory Protein Putative |
| INV10407220 | 1032 | 1 | 12 | 0.15 | -2.7 | Padr-Like Family Regulator Proteinpadr-Like |
| INV10403260 | 472 | 0 | 5.49 | 0.15 | -2.7 | Sugar Phosphotransferase System (PTS), Lactose/Cellobiose-Specific Family, IIB Component Sugar |
| rpsG | 471 | 0 | 5.48 | 0.15 | -2.7 | Putative CTP Synthase Putative |
| INV10400180 | 468 | 0 | 5.44 | 0.16 | -2.69 | Hypothetical Protein Hypothetical |
| mutX | 465 | 0 | 5.41 | 0.16 | -2.68 | Putative Maltose/Maltodextrin ABC Transport System Permease Protein Putative |
| INV10412790 | 1014 | 1 | 11.79 | 0.16 | -2.68 | Gntr Family Regulatory Protein Gntr |
| ABCtransportermembrane-spanningpermease-aminoacidtransport | 2111 | 3 | 24.54 | 0.16 | -2.67 | ABC Transporter Membrane-Spanning Permease - Amino Acid Transportabc |
| INV10417640 | 462 | 0 | 5.37 | 0.16 | -2.67 | Cell Wall Surface Anchored Protein Cell |
| INV10418990 | 1008 | 1 | 11.72 | 0.16 | -2.67 | Putative Transketolase Subunit Putative |
| INV10400950 | 456 | 0 | 5.3 | 0.16 | -2.66 | Sugar Phosphotransferase System (PTS), Sorbose-Specific Family, IIC Component Sugar |
| INV10407010 | 1536 | 2 | 17.86 | 0.16 | -2.65 | ABC Transporter ATP-Binding Protein ABC |
| pdhB | 993 | 1 | 11.54 | 0.16 | -2.65 | DNA Mismatch Repair Protein Mutl Dna |
| INV10417190 | 453 | 0 | 5.27 | 0.16 | -2.65 | Putative Uncharacterized Protein Putative |
| INV10411610 | 987 | 1 | 11.47 | 0.16 | -2.64 | GMP Reductase Gmp |
| INV10417580 | 987 | 1 | 11.47 | 0.16 | -2.64 | Membrane Protein Oxaa 2 Precursor Membrane |
| ldh | 987 | 1 | 11.47 | 0.16 | -2.64 | ABC Transporter ATP-Binding Protein ABC |
| PTSsystem_IIAcomponent | 450 | 0 | 5.23 | 0.16 | -2.64 | Phage Capsid Protein Phage |
| argR | 447 | 0 | 5.2 | 0.16 | -2.63 | Arginine Repressor Arginine |
| INV10409750 | 447 | 0 | 5.2 | 0.16 | -2.63 | Tn5252 Relaxase Tn5252 |
| rplO | 441 | 0 | 5.13 | 0.16 | -2.62 | Putative Phosphate Acetyltransferase Putative |
| INV10416060 | 960 | 1 | 11.16 | 0.16 | -2.6 | Sugar Phosphotransferase System (PTS), IIC Component Sugar |
| INV10407030 | 957 | 1 | 11.13 | 0.16 | -2.6 | Putative Uncharacterized Protein Putative |
| INV10403580 | 435 | 0 | 5.06 | 0.17 | -2.6 | Putative Hyaluronate Lyase Putative |
| INV10417620 | 954 | 1 | 11.09 | 0.17 | -2.6 | Putative 3'-5' Exoribonuclease Putative |
| INV10410350 | 432 | 0 | 5.02 | 0.17 | -2.59 | Putative IS1381 Transposase (Pseudogene) Putative |
| INV10414100 | 431 | 0 | 5.01 | 0.17 | -2.59 | Isochorismatase Family Protein Isochorismatase |
| INV10401520 | 945 | 1 | 10.99 | 0.17 | -2.58 | Metallo-Beta-Lactamase Superfamily Protein Metallo-Beta-Lactamase |
| PTSsystem_nitrogenregulatorycomponentIIA_ putative | 429 | 0 | 4.99 | 0.17 | -2.58 | Phage Endolysin Phage |
| INV10417560 | 1458 | 2 | 16.95 | 0.17 | -2.58 | Spou Rrna Methylase Family Protein Spou |
| Integralmembraneprotein | 942 | 1 | 10.95 | 0.17 | -2.58 | Serine Protease Serine |
| mutL | 1950 | 3 | 22.67 | 0.17 | -2.56 | Putative Endo-Beta-N-Acetylglucosaminidase Putative |
| INV10406600 | 927 | 1 | 10.78 | 0.17 | -2.56 | Putative Hydroxyethylthiazole Kinase Putative |
| atpC | 420 | 0 | 4.88 | 0.17 | -2.56 | ATP Synthase Alpha Chain Atp |
| conservedhypotheticalprotein | 420 | 0 | 4.88 | 0.17 | -2.56 | Conjugative Transposon Membrane Protein Conjugative |
| topA | 1932 | 3 | 22.46 | 0.17 | -2.55 | 30S Ribosomal Protein S430S |
| cysM | 921 | 1 | 10.71 | 0.17 | -2.55 | Putative Cysteine Desulfurase Putative |
| INV10402170 | 417 | 0 | 4.85 | 0.17 | -2.55 | Putative Nrdi-Like Protein Putative |
| N-acetylneuraminatelyase(EC4 | 918 | 1 | 10.67 | 0.17 | -2.54 | Membrane Protein, Putative Membrane |
| INV10406310 | 3426 | 6 | 39.83 | 0.17 | -2.54 | Putative Membrane Protein Putative |
| INV10407350 | 414 | 0 | 4.81 | 0.17 | -2.54 | Putative Exonuclease Putative |
| gidA | 1914 | 3 | 22.25 | 0.17 | -2.54 | Putative Fuculokinase Putative |
| INV10412400 | 912 | 1 | 10.6 | 0.17 | -2.54 | Putative Extracellular Amino Acid-Binding Protein Putative |
| INV10418090 | 912 | 1 | 10.6 | 0.17 | -2.54 | Conserved Hypothetical Protein Conserved |
| INV10405080 | 897 | 1 | 10.43 | 0.18 | -2.51 | Phosphotransferase Enzyme Family Protein Phosphotransferase |
| INV10403210 | 1388 | 2 | 16.14 | 0.18 | -2.51 | Putative Sugar-Binding Regulatory Protein Putative |
| INV10415900 | 405 | 0 | 4.71 | 0.18 | -2.51 | Sugar Binding Transcriptional Regulator Sugar |
| spxA | 402 | 0 | 4.67 | 0.18 | -2.5 | 50S Ribosomal Protein L550S |
| INV10411920 | 885 | 1 | 10.29 | 0.18 | -2.5 | Putative Zinc-Binding Dehydrogenase Putative |
| rpsH | 399 | 0 | 4.64 | 0.18 | -2.5 | Uridylate Kinaseuridylate |
| INV10413620 | 883 | 1 | 10.27 | 0.18 | -2.49 | Putative Polysaccharide Biosynthesis Protein Putative |
| INV10419310 | 1365 | 2 | 15.87 | 0.18 | -2.49 | PTS System, Iid Component Pts |
| uvrC | 1846 | 3 | 21.46 | 0.18 | -2.49 | Tn5252, Orf 9 Protein Tn5252, |
| INV10410060 | 1362 | 2 | 15.83 | 0.18 | -2.49 | ABC Transporter ATP-Binding Protein ABC |
| pstS | 879 | 1 | 10.22 | 0.18 | -2.49 | Putative Formate Acetyltransferase Putative |
| INV10416450 | 396 | 0 | 4.6 | 0.18 | -2.49 | DNA Methylase Dna |
| INV10417960 | 876 | 1 | 10.18 | 0.18 | -2.48 | Putative Phosphotyrosine Protein Phosphatase Putative |
| addB | 3276 | 6 | 38.09 | 0.18 | -2.48 | Putative ATP-Dependent Exonuclease Subunit B Putative |
| sdhA | 873 | 1 | 10.15 | 0.18 | -2.48 | 16S Rrna Processing Protein 16s |
| Phageterminase_smallsubunit | 393 | 0 | 4.57 | 0.18 | -2.48 | N-Ethylammeline Chlorohydrolasen-Ethylammeline |
| CholinebindingproteinA | 390 | 0 | 4.53 | 0.18 | -2.47 | Cell Wall-Associated Murein Hydrolase Lytacell |
| INV10403730 | 390 | 0 | 4.53 | 0.18 | -2.47 | Putative Sugar-Phosphate Isomerase Putative |
| INV10403470 | 866 | 1 | 10.07 | 0.18 | -2.47 | Putative Ribosomal Small Subunit Pseudouridine Synthase B Putative |
| INV10416410 | 864 | 1 | 10.04 | 0.18 | -2.46 | Hypothetical Protein Hypothetical |
| Phagetaillengthtape-measureprotein | 2760 | 5 | 32.09 | 0.18 | -2.46 | N-Ethylammeline Chlorohydrolasen-Ethylammeline |
| ciaH | 1335 | 2 | 15.52 | 0.18 | -2.46 | Chlorohydrolase Chlorohydrolase |
| INV10415340 | 856 | 1 | 9.95 | 0.18 | -2.45 | Putative Membrane Protein Putative |
| INV10415890 | 855 | 1 | 9.94 | 0.18 | -2.45 | ABC Transporter, Substrate Binding Protein ABC |
| FIG00627694-hypotheticalprotein | 384 | 0 | 4.46 | 0.18 | -2.45 | 3-Oxoacyl-[Acyl-Carrier Protein] Reductase3-Oxoacyl-[Acyl-Carrier |
| gapN | 1320 | 2 | 15.35 | 0.18 | -2.45 | Putative Cell Division Protein Putative |
| INV10405690 | 381 | 0 | 4.43 | 0.18 | -2.44 | Putative Extracellular Solute-Binding Protein Putative |
| 1403638_1404484 | 846 | 1 | 9.84 | 0.18 | -2.44 |  |
| INV10411630 | 846 | 1 | 9.84 | 0.18 | -2.44 | Putative Endonuclease Putative |
| INV10412710 | 846 | 1 | 9.84 | 0.18 | -2.44 | Putative ABC Transporter Putative |
| INV10409620 | 840 | 1 | 9.77 | 0.19 | -2.43 | Putative Site-Specific Recombinase (Pseudogene) Putative |
| relA | 2223 | 4 | 25.84 | 0.19 | -2.42 | Positive Transcriptional Regulator, Mutr Family Positive |
| dnaA | 1299 | 2 | 15.1 | 0.19 | -2.42 | Dephospho-Coa Kinase (EC 2.7.1.24) Dephospho-Coa |
| INV10412740 | 837 | 1 | 9.73 | 0.19 | -2.42 | Putative Membrane Protein Putative |
| ftsY | 1290 | 2 | 15 | 0.19 | -2.42 | GTP Cyclohydrolase IGTP |
| INV10417590 | 831 | 1 | 9.66 | 0.19 | -2.41 | Pyridoxal-Dependent Decarboxylasepyridoxal-Dependent |
| INV10403550 | 372 | 0 | 4.32 | 0.19 | -2.41 | Hypothetical Proteinhypothetical |
| purA | 1287 | 2 | 14.96 | 0.19 | -2.41 | Phage Hyaluronidasephage |
| INV10419270 | 369 | 0 | 4.29 | 0.19 | -2.4 | Conserved Hypothetical Protein Conserved |
| Phagetranscriptionalregulator_CroCIfamily | 369 | 0 | 4.29 | 0.19 | -2.4 | Putative Nicotinate-Nucleotide Pyrophosphorylase Putative |
| rplN | 369 | 0 | 4.29 | 0.19 | -2.4 | Putative Phosphate ABC Transporter, Extracellular Phosphate-Binding Lipoprotein Putative |
| Phageterminaselargesubunit | 1731 | 3 | 20.12 | 0.19 | -2.4 | N-Ethylammeline Chlorohydrolasen-Ethylammeline |
| INV10403810 | 366 | 0 | 4.26 | 0.19 | -2.4 | Putative Membrane Protein Putative |
| INV10417700 | 366 | 0 | 4.26 | 0.19 | -2.4 | Conserved Hypothetical Proteinconserved |
| rpsM | 366 | 0 | 4.26 | 0.19 | -2.4 | DNA Repair Protein Radcdna |
| pabB | 1722 | 3 | 20.02 | 0.19 | -2.39 | Multidrug Resistance Efflux Pump Pmra Multidrug |
| INV10400530 | 816 | 1 | 9.49 | 0.19 | -2.39 | Translation Initiation Factor IF-3translation |
| INV10406590 | 816 | 1 | 9.49 | 0.19 | -2.39 | Putative Membrane Protein Putative |
| INV10417980 | 816 | 1 | 9.49 | 0.19 | -2.39 | Conserved Hypothetical Protein Conserved |
| INV10404730 | 364 | 0 | 4.23 | 0.19 | -2.39 | PTS Transporterpts |
| INV10417000 | 1251 | 2 | 14.54 | 0.19 | -2.37 | Putative Cell Wall-Binding Protein Putative |
| INV10406030 | 804 | 1 | 9.35 | 0.19 | -2.37 | Putative Uncharacterized Protein Putative |
| Phosphatidatecytidylyltransferase(EC2 | 804 | 1 | 9.35 | 0.19 | -2.37 | Nucleoside Diphosphate Kinase Nucleoside |
| INV10405530 | 1693 | 3 | 19.68 | 0.19 | -2.37 | Putative Flavocytochrome Putative |
| argS | 1692 | 3 | 19.67 | 0.19 | -2.37 | Putative Arginine Repressor Putative |
| INV10418650 | 801 | 1 | 9.31 | 0.19 | -2.37 | ABC Transporter ATP-Binding Membrane Protein ABC |
| malA | 801 | 1 | 9.31 | 0.19 | -2.37 | IS1381, Transposase Orfais1381, |
| rplR | 357 | 0 | 4.15 | 0.19 | -2.36 | Hpr Kinase/Phosphorylase Hpr |
| INV10404570 | 795 | 1 | 9.24 | 0.2 | -2.36 | Conserved Hypothetical Protein Conserved |
| INV10408980 | 795 | 1 | 9.24 | 0.2 | -2.36 | Tetrapyrrole (Corrin/Porphyrin) Methylase Family Protein Tetrapyrrole |
| murN | 1233 | 2 | 14.33 | 0.2 | -2.35 | LSU Ribosomal Protein L7/L12 (P1/P2) LSU |
| ugd | 1233 | 2 | 14.33 | 0.2 | -2.35 | Homoserine Kinasehomoserine |
| recN | 1668 | 3 | 19.39 | 0.2 | -2.35 | Putative Bacteeriocin ABC Transporter Transmembrane Domain Blpy (CAAX Protease) Putative |
| INV10406730 | 1224 | 2 | 14.23 | 0.2 | -2.34 | Tetr Family Regulatory Protein Tetr |
| murM | 1221 | 2 | 14.2 | 0.2 | -2.34 | LSU Ribosomal Protein L27plsu |
| dnaH | 1656 | 3 | 19.25 | 0.2 | -2.34 | D-Alanine--Poly(Phosphoribitol) Ligase Subunit 1D-Alanine--Poly(Phosphoribitol) |
| putativetransposonintegraseTn916ORF3-like | 1218 | 2 | 14.16 | 0.2 | -2.34 | Phenylalanyl-Trna Synthetase Beta Chainphenylalanyl-Trna |
| INV10412730 | 348 | 0 | 4.05 | 0.2 | -2.34 | Putative ABC Transporter Putative |
| prophagepi2protein37 | 348 | 0 | 4.05 | 0.2 | -2.34 | Putative Xaa-Pro Dipeptidase Putative |
| rplS | 348 | 0 | 4.05 | 0.2 | -2.34 | PTS System, Fructose-Specific IIA Component (EC 2.7.1.69) / PTS System, Fructose-Specific IIB Component (EC 2.7.1.69) / PTS System, Fructose-Specific IIC Component (EC 2.7.1.69) PTS |
| thiI | 1215 | 2 | 14.13 | 0.2 | -2.33 | 50S Ribosomal Protein L2750S |
| INV10400150 | 781 | 1 | 9.08 | 0.2 | -2.33 | Hypothetical Protein Hypothetical |
| INV10405120 | 781 | 1 | 9.08 | 0.2 | -2.33 | Ribosomal Protein L7Ae/L30e/S12e/Gadd45 Family Protein Ribosomal |
| INV10412770 | 1212 | 2 | 14.09 | 0.2 | -2.33 | Putative Transposase Remnant (Pseudogene) Putative |
| ribA | 1206 | 2 | 14.02 | 0.2 | -2.32 | Putative Spermidine/Putrescine ABC Transporter ATP-Binding Protein Putative |
| INV10418120 | 2064 | 4 | 24 | 0.2 | -2.32 | Putative Ssdna-Binding Protein Putative |
| Zetatoxin | 772 | 1 | 8.98 | 0.2 | -2.32 | Transcriptional Regulator Spxa2transcriptional |
| OligopeptideABCtransporter_periplasmicoligopeptide-bindingproteinOppA(TC3 | 1629 | 3 | 18.94 | 0.2 | -2.32 | Multiple Sugar-Binding Transport System Permease Protein Multiple |
| INV10415880 | 771 | 1 | 8.96 | 0.2 | -2.32 | ABC Transporter Abc |
| ugl | 1191 | 2 | 13.85 | 0.2 | -2.31 | Threonyl-Trna Synthetasethreonyl-Trna |
| pbp1B | 2466 | 5 | 28.67 | 0.2 | -2.31 | UDP-N-Acetylmuramoylalanyl-D-Glutamate--2,6-Dia Minopimelate Ligaseudp-N-Acetylmuramoylalanyl-D-Glutamate--2,6-Dia |
| INV10416460 | 762 | 1 | 8.86 | 0.2 | -2.3 | DNA Methylasedna |
| htrA | 1182 | 2 | 13.74 | 0.2 | -2.3 | DNA Gyrase Subunit ADNA |
| rmlD | 754 | 1 | 8.77 | 0.2 | -2.29 | Putative Gamma-Glutamyl Phosphate Reductase Putative |
| Phagecapsidprotein | 1173 | 2 | 13.64 | 0.2 | -2.29 | N-Ethylammeline Chlorohydrolasen-Ethylammeline |
| pstB3 | 753 | 1 | 8.75 | 0.21 | -2.29 | Formate Acetyltransferaseformate |
| INV10411100 | 333 | 0 | 3.87 | 0.21 | -2.28 | Putative Bacteriocin Immunity Protein Putative |
| INV10400290 | 1170 | 2 | 13.6 | 0.21 | -2.28 | Putative Gluconate 5-Dehydrogenase Putative |
| CelldivisionproteinGpsB_coordinatestheswitchbetweencylindricalandseptalcellwallsynthesisbyre-localizationofPBP1 | 330 | 0 | 3.84 | 0.21 | -2.28 | Cytidine Deaminasecytidine |
| OligopeptidetransportATP-bindingproteinOppD(TC3 | 1983 | 4 | 23.05 | 0.21 | -2.27 | Multiple Sugar-Binding Transport System Permease Protein (Pseudogene) Multiple |
| Transcriptionalregulator_MerRfamily | 741 | 1 | 8.61 | 0.21 | -2.26 | 30S Ribosomal Protein S1130S |
| tkt | 1977 | 4 | 22.98 | 0.21 | -2.26 | 50S Ribosomal Protein L3450S |
| INV10405520 | 738 | 1 | 8.58 | 0.21 | -2.26 | Two-Component System, Sensor Histidine Kinase Two-Component |
| xerD | 735 | 1 | 8.55 | 0.21 | -2.26 | Transcriptional Regulator, Cro/CI Family Transcriptional |
| fabG | 732 | 1 | 8.51 | 0.21 | -2.25 | Putative Competence Associated Endonuclease Putative |
| INV10413000 | 1959 | 4 | 22.78 | 0.21 | -2.25 | ABC Transporter Permease Protein ABC |
| lig | 1959 | 4 | 22.78 | 0.21 | -2.25 | Putative Membrane Protein Putative |
| INV10410330 | 729 | 1 | 8.48 | 0.21 | -2.24 | Putative Metallopeptidase Putative |
| INV10411600 | 1134 | 2 | 13.18 | 0.21 | -2.24 | Putative Haloacid Dehalogenase-Like Hydrolase Putative |
| FIG01115816-hypotheticalprotein | 723 | 1 | 8.41 | 0.21 | -2.23 | FIG01114767: Hypothetical Proteinfig01114767: |
| INV10411030 | 318 | 0 | 3.7 | 0.21 | -2.23 | Calcineurin-Like Phosphoesterase Calcineurin-Like |
| INV10416480 | 318 | 0 | 3.7 | 0.21 | -2.23 | Zinc-Binding Dehydrogenasezinc-Binding |
| ribC | 318 | 0 | 3.7 | 0.21 | -2.23 | Putative Spermidine/Putrescine ABC Transporter Permease Protein Putative |
| atpB | 717 | 1 | 8.34 | 0.21 | -2.22 | Aspartate Aminotransferaseaspartate |
| LSUribosomalproteinL21p | 315 | 0 | 3.66 | 0.21 | -2.22 | Putative Sensor Histidine Kinase (Pseudogene) Putative |
| proB | 1110 | 2 | 12.9 | 0.22 | -2.21 | Penicillin-Binding Protein 2bpenicillin-Binding |
| PTSsystem_fructose-specificIIAcomponent(EC2 | 312 | 0 | 3.63 | 0.22 | -2.21 | Phage Antirepressor Proteinphage |
| 1940076_1940387 | 311 | 0 | 3.62 | 0.22 | -2.21 |  |
| INV10417880 | 311 | 0 | 3.62 | 0.22 | -2.21 | Putative Exported PTS System Protein Putative |
| INV10411960 | 708 | 1 | 8.23 | 0.22 | -2.21 | Putative Phosphotransferase Licd1 Putative |
| INV10402100 | 309 | 0 | 3.59 | 0.22 | -2.2 | Putative Histidine Sensor Kinase Putative |
| INV10407320 | 1494 | 3 | 17.37 | 0.22 | -2.2 | Conserved Hypothetical Protein Conserved |
| recF | 1098 | 2 | 12.77 | 0.22 | -2.2 | Putative Immunity Protein Putative |
| INV10417490 | 1092 | 2 | 12.7 | 0.22 | -2.19 | Putative ABC Transporter ATP-Binding Protein Putative |
| INV10418080 | 699 | 1 | 8.13 | 0.22 | -2.19 | Putative Sugar-Specific Permease, Sgat/Ulaa Family Putative |
| N-acetylmannosamine-6-phosphate2-epimerase(EC5 | 699 | 1 | 8.13 | 0.22 | -2.19 | Adapter Protein Mecaadapter |
| INV10401600 | 306 | 0 | 3.56 | 0.22 | -2.19 | Putative Glycoprotease Putative |
| INV10402160 | 696 | 1 | 8.09 | 0.22 | -2.18 | Putative Membrane Protein Putative |
| INV10407870 | 1086 | 2 | 12.63 | 0.22 | -2.18 | Conserved Hypothetical Protein Conserved |
| INV10409460 | 1083 | 2 | 12.59 | 0.22 | -2.18 | Conserved Hypothetical Protein Conserved |
| INV10401260 | 693 | 1 | 8.06 | 0.22 | -2.18 | Putative Membrane Protein Putative |
| dpfB | 690 | 1 | 8.02 | 0.22 | -2.17 | DNA Polymerase III Alpha Subunit Dna |
| INV10401560 | 690 | 1 | 8.02 | 0.22 | -2.17 | Putative Membrane Protein Putative |
| 1013649_1014727 | 1078 | 2 | 12.53 | 0.22 | -2.17 |  |
| zmpA | 6481 | 16 | 75.35 | 0.22 | -2.17 | Transcription Regulator, Probable Transcription |
| INV10404620 | 300 | 0 | 3.49 | 0.22 | -2.17 | Conserved Hypothetical Protein Conserved |
| INV10409540 | 300 | 0 | 3.49 | 0.22 | -2.17 | Putative Transposase (Fragment) Putative |
| INV10417470 | 300 | 0 | 3.49 | 0.22 | -2.17 | Transporter |
| FIG01114045-hypotheticalprotein | 297 | 0 | 3.45 | 0.22 | -2.15 | Ferrous Iron Transport Peroxidase Efeb Ferrous |
| rplW | 297 | 0 | 3.45 | 0.22 | -2.15 | Adenylosuccinate Synthetase Adenylosuccinate |
| INV10405070 | 678 | 1 | 7.88 | 0.23 | -2.15 | Conserved Hypothetical Protein Conserved |
| 74892_75187 | 295 | 0 | 3.43 | 0.23 | -2.15 |  |
| INV10400330 | 295 | 0 | 3.43 | 0.23 | -2.15 | Threonine Dehydratase Biosynthetic Threonine |
| ciaR | 675 | 1 | 7.85 | 0.23 | -2.15 | Choline Binding Protein Acholine |
| LSUribosomalproteinL27p | 294 | 0 | 3.42 | 0.23 | -2.14 | Conserved Hypothetical Protein Conserved |
| rpsK | 294 | 0 | 3.42 | 0.23 | -2.14 | Pyrr Bifunctional Protein [Includes: Pyrimidine Operon Regulatory Protein; Uracil Phosphoribosyltransferase]Pyrr |
| 846743_847794 | 1051 | 2 | 12.22 | 0.23 | -2.14 |  |
| INV10407810 | 1051 | 2 | 12.22 | 0.23 | -2.14 | Putative Membrane Protein Putative |
| INV10402260 | 1050 | 2 | 12.21 | 0.23 | -2.14 | Putative Integral Membrane Protein Putative |
| INV10416490 | 291 | 0 | 3.38 | 0.23 | -2.13 | Merr Family Regulatory Protein Merr |
| INV10419010 | 291 | 0 | 3.38 | 0.23 | -2.13 | Sugar Phosphotransferase System (PTS), Lactose/Cellobiose-Specific Family, IIB Subunit Protein Sugar |
| rpsF | 291 | 0 | 3.38 | 0.23 | -2.13 | Orotidine 5'-Phosphate Decarboxylase Orotidine |
| Degeneratetransposase | 290 | 0 | 3.37 | 0.23 | -2.13 | D-Alanyl-D-Alanine Carboxypeptidase (EC 3.4.16.4)D-Alanyl-D-Alanine |
| INV10400630 | 666 | 1 | 7.74 | 0.23 | -2.13 | Putative Putative GTP-Binding Protein Putative |
| comFC | 663 | 1 | 7.71 | 0.23 | -2.12 | Putative Competence Protein Putative |
| INV10406700 | 288 | 0 | 3.35 | 0.23 | -2.12 | Sodium:Neurotransmitter Symporter Family Protein (Pseudogene) Sodium:Neurotransmitter |
| INV10411590 | 1035 | 2 | 12.03 | 0.23 | -2.12 | Haloacid Dehalogenase-Like Hydrolase Haloacid |
| INV10400350 | 2154 | 5 | 25.04 | 0.23 | -2.12 | Ketol-Acid Reductoisomeraseketol-Acid |
| INV10413100 | 1401 | 3 | 16.29 | 0.23 | -2.11 | Putative NADPH-Dependent FMN Reductase Putative |
| IroncompoundABCuptaketransportersubstrate-bindingproteinPiaA | 1026 | 2 | 11.93 | 0.23 | -2.11 | Major Facilitator Family Transport Protein Major |
| phoU | 654 | 1 | 7.6 | 0.23 | -2.1 | Anaerobic Ribonucleoside-Triphosphate Reductaseanaerobic |
| INV10406000 | 651 | 1 | 7.57 | 0.23 | -2.1 | Putative Membrane Protein Putative |
| INV10418000 | 651 | 1 | 7.57 | 0.23 | -2.1 | Putative Transcriptional Antiterminator Putative |
| INV10417550 | 282 | 0 | 3.28 | 0.23 | -2.1 | Putative Membrane Protein Putative |
| Orf46 | 282 | 0 | 3.28 | 0.23 | -2.1 | Metal ABC Transporter Substrate-Binding Lipoprotein Precursormetal |
| rpsS | 282 | 0 | 3.28 | 0.23 | -2.1 | Putative DNA Repair Protein Putative |
| INV10412480 | 648 | 1 | 7.53 | 0.23 | -2.09 | Haloacid Dehalogenase-Like Hydrolasehaloacid |
| Beta-glucosidase(EC3 | 1380 | 3 | 16.04 | 0.23 | -2.09 | Bacteriocin Immunity Protein Blplbacteriocin |
| INV10400620 | 1380 | 3 | 16.04 | 0.23 | -2.09 | Conserved Hypothetical Proteinconserved |
| INV10400280 | 1011 | 2 | 11.75 | 0.24 | -2.09 | Hypothetical Proteinhypothetical |
| INV10411970 | 279 | 0 | 3.24 | 0.24 | -2.08 | Putative Phosphotransferase Licd2 Putative |
| INV10418130 | 279 | 0 | 3.24 | 0.24 | -2.08 | Membrane Protein Oxaa 1 Precursormembrane |
| murF | 1374 | 3 | 15.97 | 0.24 | -2.08 | LSU Ribosomal Protein L10p (P0)LSU |
| trmE | 1374 | 3 | 15.97 | 0.24 | -2.08 | S-Adenosylhomocysteine Deaminase (EC 3.5.4.28); Methylthioadenosine Deaminases-Adenosylhomocysteine |
| Membrane-boundprotease_CAAXfamily | 642 | 1 | 7.46 | 0.24 | -2.08 | IS861, Transposase (Orf1), IS3 Family, Truncatedis861, |
| INV10403560 | 1365 | 3 | 15.87 | 0.24 | -2.08 | Putative Glycosylhydrolase Putative |
| adk | 639 | 1 | 7.43 | 0.24 | -2.08 | Adenylate Kinaseadenylate |
| udk | 639 | 1 | 7.43 | 0.24 | -2.08 | Hydroxyethylthiazole Kinasehydroxyethylthiazole |
| INV10411140 | 1000 | 2 | 11.63 | 0.24 | -2.07 | Putative Plasmid Addiction System, Toxin Protein Putative |
| pgmA | 1719 | 4 | 19.99 | 0.24 | -2.07 | N-Ethylammeline Chlorohydrolasen-Ethylammeline |
| INV10415370 | 996 | 2 | 11.58 | 0.24 | -2.07 | Putative Membrane Protein Putative |
| dnaC | 1353 | 3 | 15.73 | 0.24 | -2.06 | Diacylglycerol Kinase (EC 2.7.1.107)Diacylglycerol |
| INV10402010 | 993 | 2 | 11.54 | 0.24 | -2.06 | Putative Membrane Protease Putative |
| INV10412220 | 993 | 2 | 11.54 | 0.24 | -2.06 | Putative Membrane Protein Putative |
| rpsP | 273 | 0 | 3.17 | 0.24 | -2.06 | Reca Recombinase (Recombinase A) Reca |
| INV10400360 | 1350 | 3 | 15.7 | 0.24 | -2.06 | Dihydroxy-Acid Dehydratasedihydroxy-Acid |
| INV10404060 | 1350 | 3 | 15.7 | 0.24 | -2.06 | Choline Binding Protein (Fragment) Choline |
| nth | 630 | 1 | 7.32 | 0.24 | -2.06 | Mobile Element Proteinmobile |
| INV10419120 | 271 | 0 | 3.15 | 0.24 | -2.05 | ROK Family Proteinrok |
| INV10401840 | 270 | 0 | 3.14 | 0.24 | -2.05 | Hypothetical Protein (Fragment) Hypothetical |
| INV10401990 | 1338 | 3 | 15.56 | 0.24 | -2.05 | Hypothetical Proteinhypothetical |
| INV10412680 | 625 | 1 | 7.27 | 0.24 | -2.05 | Putative Restriction Endonuclease (Pseudogene) Putative |
| INV10400890 | 978 | 2 | 11.37 | 0.24 | -2.04 | Gntr Family Regulatory Proteingntr |
| prfB | 978 | 2 | 11.37 | 0.24 | -2.04 | Pyrrolidone-Carboxylate Peptidasepyrrolidone-Carboxylate |
| glyS | 2037 | 5 | 23.68 | 0.24 | -2.04 | Putative Glutamine Transporter, ATP-Binding Protein 3 Putative |
| INV10400070 | 267 | 0 | 3.1 | 0.24 | -2.04 | Hypothetical Proteinhypothetical |
| INV10401580 | 267 | 0 | 3.1 | 0.24 | -2.04 | Glycoprotease Family Proteinglycoprotease |
| INV10401980 | 267 | 0 | 3.1 | 0.24 | -2.04 | Putative Acetyltransferase Putative |
| INV10403510 | 1323 | 3 | 15.38 | 0.24 | -2.03 | Putative CAAX Amino Terminal Protease Family Membrane Protein Putative |
| INV10405570 | 618 | 1 | 7.18 | 0.24 | -2.03 | Putative Amino Acid ABC Transporter, Permease Protein Putative |
| INV10411990 | 618 | 1 | 7.18 | 0.24 | -2.03 | Conserved Hypothetical Proteinconserved |
| 600828_601795 | 967 | 2 | 11.24 | 0.25 | -2.03 |  |
| INV10401190 | 615 | 1 | 7.15 | 0.25 | -2.03 | Conserved Hypothetical Proteinconserved |
| secY | 1311 | 3 | 15.24 | 0.25 | -2.02 | Dtdp-D-Glucose 4,6-Dehydratase Rmlbdtdp-D-Glucose |
| pncP | 612 | 1 | 7.12 | 0.25 | -2.02 | Oligopeptide Transport System Permease Protein Oppc (TC 3.A.1.5.1)Oligopeptide |
| rpsQ | 261 | 0 | 3.03 | 0.25 | -2.01 | DNA Replication And Repair Protein Recfdna |
| INV10410660 | 954 | 2 | 11.09 | 0.25 | -2.01 | ABC Transporter ATP-Binding Protein ABC |
| OligopeptidetransportsystempermeaseproteinOppB(TC3 | 951 | 2 | 11.06 | 0.25 | -2.01 | Multiple Sugar-Binding Transport ATP-Binding Proteinmultiple |
| 476902_485073 | 8171 | 23 | 95 | 0.25 | -2 |  |
| Histidyl-tRNAsynthetase(EC6 | 1290 | 3 | 15 | 0.25 | -2 | 2,3-Bisphosphoglycerate-Dependent Phosphoglycerate Mutase2,3-Bisphosphoglycerate-Dependent |
| treA | 1626 | 4 | 18.9 | 0.25 | -1.99 | 30S Ribosomal Protein S2130S |
| rmlC | 597 | 1 | 6.94 | 0.25 | -1.99 | Putative Primosomal Protein N' Putative |
| INV10404710 | 255 | 0 | 2.96 | 0.25 | -1.99 | Putative Uncharacterized Protein Putative |
| birA | 936 | 2 | 10.88 | 0.25 | -1.99 | Beta-Glucosidase (EC 3.2.1.21)Beta-Glucosidase |
| INV10411200 | 936 | 2 | 10.88 | 0.25 | -1.99 | Mutt/NUDIX Hydrolase Family Proteinmutt/NUDIX |
| rpoA | 936 | 2 | 10.88 | 0.25 | -1.99 | Putative Methyltransferase Putative |
| FIG01117121-hypotheticalprotein | 933 | 2 | 10.85 | 0.25 | -1.98 | FIG01115786: Hypothetical Proteinfig01115786: |
| INV10411870 | 1272 | 3 | 14.79 | 0.25 | -1.98 | Putative Licb-Family Membrane Protein Putative |
| gyrB | 1947 | 5 | 22.64 | 0.25 | -1.98 | 6-Phosphogluconate Dehydrogenase, Decarboxylating6-Phosphogluconate |
| rnz | 930 | 2 | 10.81 | 0.25 | -1.98 | Prolyl-Trna Synthetase (EC 6.1.1.15), Bacterial Typeprolyl-Trna |
| nrdG | 591 | 1 | 6.87 | 0.25 | -1.98 | Mobile Element Proteinmobile |
| INV10409680 | 2280 | 6 | 26.51 | 0.25 | -1.97 | Putative Hydrolase Putative |
| ftsX | 927 | 2 | 10.78 | 0.25 | -1.97 | Fold Bifunctional Protein [Includes: Methylenetetrahydrofolate Dehydrogenase/Methenyltetrahydrofolate Cyclohydrolase]Fold |
| INV10416290 | 927 | 2 | 10.78 | 0.25 | -1.97 | Putative Degt/Dnrj/Eryc1/Strs Family Amino Sugar Synthetase Putative |
| INV10409550 | 1939 | 5 | 22.54 | 0.25 | -1.97 | Putative Uncharacterized Protein Putative |
| sufD | 1263 | 3 | 14.68 | 0.26 | -1.97 | 50S Ribosomal Protein L1750S |
| INV10409580 | 250 | 0 | 2.91 | 0.26 | -1.97 | Putative Uncharacterized Protein Putative |
| tyrS | 1257 | 3 | 14.61 | 0.26 | -1.96 | Putative Thiamine Biosynthesis Protein Putative |
| INV10406200 | 249 | 0 | 2.89 | 0.26 | -1.96 | Conserved Hypothetical Proteinconserved |
| INV10416190 | 249 | 0 | 2.89 | 0.26 | -1.96 | Putative Cation Transporter Putative |
| glgP | 2259 | 6 | 26.26 | 0.26 | -1.96 | Putative NADP-Dependent Glyceraldehyde-3-Phosphate Dehydrogenase Putative |
| INV10410770 | 1914 | 5 | 22.25 | 0.26 | -1.95 | Conserved Hypothetical Proteinconserved |
| INV10409140 | 1909 | 5 | 22.19 | 0.26 | -1.95 | ABC Transporter ATP-Binding Protein ABC |
| INV10405300 | 246 | 0 | 2.86 | 0.26 | -1.95 | Cell Filamentation Protein Fic-Related Proteincell |
| INV10407210 | 1242 | 3 | 14.44 | 0.26 | -1.95 | Putative Permease Putative |
| ffh | 1572 | 4 | 18.28 | 0.26 | -1.95 | Septation Ring Formation Regulatorseptation |
| INV10405630 | 1239 | 3 | 14.4 | 0.26 | -1.94 | Putative Phosphoesterase Putative |
| INV10419320 | 2553 | 7 | 29.68 | 0.26 | -1.94 | PTS System, Iic Componentpts |
| INV10418150 | 573 | 1 | 6.66 | 0.26 | -1.94 | Putative Exported Protein Putative |
| INV10410510 | 243 | 0 | 2.83 | 0.26 | -1.94 | Putative RNA Pseudouridylate Synthase Putative |
| INV10403530 | 900 | 2 | 10.46 | 0.26 | -1.93 | Conserved Hypothetical Proteinconserved |
| pth | 570 | 1 | 6.63 | 0.26 | -1.93 | Phosphoglycerate Kinasephosphoglycerate |
| FIG01114589-hypotheticalprotein | 567 | 1 | 6.59 | 0.26 | -1.92 | FIG00628965: Hypothetical Proteinfig00628965: |
| INV10405500 | 567 | 1 | 6.59 | 0.26 | -1.92 | Two-Component System, Response Regulatortwo-Component |
| INV10415400 | 567 | 1 | 6.59 | 0.26 | -1.92 | Putative Membrane Protein Putative |
| prfC | 1545 | 4 | 17.96 | 0.26 | -1.92 | Putative ATP-Dependent DNA Helicase Putative |
| INV10403360 | 240 | 0 | 2.79 | 0.26 | -1.92 | Putative Plasmid Stabilization System Antitoxin Protein Putative |
| INV10415960 | 891 | 2 | 10.36 | 0.26 | -1.92 | Putative Membrane Protein Putative |
| INV10405540 | 564 | 1 | 6.56 | 0.26 | -1.92 | Putative Amino Acid ABC Transporter, Permease Protein Putative |
| INV10410640 | 564 | 1 | 6.56 | 0.26 | -1.92 | Conserved Hypothetical Proteinconserved |
| INV10405980 | 562 | 1 | 6.53 | 0.27 | -1.91 | Putative Uncharacterized Protein Putative |
| INV10404950 | 885 | 2 | 10.29 | 0.27 | -1.91 | Putative Na+/Pi-Cotransporter Protein Putative |
| INV10404740 | 561 | 1 | 6.52 | 0.27 | -1.91 |  |
| FIG01120216-hypotheticalprotein | 237 | 0 | 2.76 | 0.27 | -1.91 | FIG01116413: Hypothetical Proteinfig01116413: |
| INV10400160 | 237 | 0 | 2.76 | 0.27 | -1.91 | Hypothetical Proteinhypothetical |
| INV10407340 | 237 | 0 | 2.76 | 0.27 | -1.91 | Putative Peptidase Putative |
| INV10400820 | 1851 | 5 | 21.52 | 0.27 | -1.91 | Putative Acyl Carrier Protein Putative |
| INV10411040 | 1527 | 4 | 17.75 | 0.27 | -1.91 | Putative Repressor Protein Putative |
| INV10407880 | 876 | 2 | 10.18 | 0.27 | -1.9 | Putative D-Alanyl-D-Alanine Carboxypeptidase Putative |
| INV10412500 | 876 | 2 | 10.18 | 0.27 | -1.9 | Putative Exported Hydrophilic Protein Putative |
| INV10406740 | 555 | 1 | 6.45 | 0.27 | -1.9 | Putative Cytidine And Deoxycytidylate Deaminase Putative |
| INV10400320 | 234 | 0 | 2.72 | 0.27 | -1.9 | Isoleucyl-Trna Synthetaseisoleucyl-Trna |
| INV10404690 | 234 | 0 | 2.72 | 0.27 | -1.9 | Putative Transposase (Pseudogene) Putative |
| PreproteintranslocasesubunitSecG(TC3 | 234 | 0 | 2.72 | 0.27 | -1.9 | Penicillin Binding Protein 2xpenicillin |
| ackA | 1191 | 3 | 13.85 | 0.27 | -1.89 | Acetate Kinaseacetate |
| metK | 1191 | 3 | 13.85 | 0.27 | -1.89 | Putative Lactose-Specific Phosphotransferase System (PTS), IIBC Component 2 Putative |
| INV10409360 | 552 | 1 | 6.42 | 0.27 | -1.89 | Putative Methyltransferase Putative |
| wchC | 552 | 1 | 6.42 | 0.27 | -1.89 | Putative IS1670 Transposase (Pseudogene) Putative |
| dnaK | 1824 | 5 | 21.21 | 0.27 | -1.89 | DNA-Cytosine Methyltransferase (EC 2.1.1.37)DNA-Cytosine |
| INV10403520 | 549 | 1 | 6.38 | 0.27 | -1.88 | Putative 2-Amino-4-Hydroxy-6-Hydroxymethyldihydropteridine Pyrophosphokinase Putative |
| INV10419070 | 549 | 1 | 6.38 | 0.27 | -1.88 | Putative Membrane Protein Putative |
| INV10405680 | 231 | 0 | 2.69 | 0.27 | -1.88 | Putative Uncharacterized Protein Putative |
| INV10406810 | 231 | 0 | 2.69 | 0.27 | -1.88 | Branched-Chain Amino Acid Transport System Permease Proteinbranched-Chain |
| INV10407120 | 231 | 0 | 2.69 | 0.27 | -1.88 | Bioy Family Membrane Proteinbioy |
| INV10412700 | 231 | 0 | 2.69 | 0.27 | -1.88 | Putative Arac-Family Transcriptional Regulator Putative |
| INV10413630 | 548 | 1 | 6.37 | 0.27 | -1.88 | Csbd-Like Proteincsbd-Like |
| INV10416300 | 1497 | 4 | 17.4 | 0.27 | -1.88 | Probable Sugar Transferaseprobable |
| galK | 1179 | 3 | 13.71 | 0.27 | -1.88 | Putative Cell Division ATP-Binding Protein Putative |
| msmR | 862 | 2 | 10.02 | 0.27 | -1.88 | 3-Isopropylmalate Dehydrogenase3-Isopropylmalate |
| mutY | 1176 | 3 | 13.67 | 0.27 | -1.87 | Putative 4-Alpha-Glucanotransferase Putative |
| INV10419360 | 543 | 1 | 6.31 | 0.27 | -1.87 | ABC Transporter Permease Protein ABC |
| INV10417820 | 1485 | 4 | 17.26 | 0.27 | -1.87 | Dicarboxylate Carrier Proteindicarboxylate |
| aroE | 855 | 2 | 9.94 | 0.27 | -1.87 | 3-Dehydroquinate Dehydratase3-Dehydroquinate |
| INV10412970 | 1167 | 3 | 13.57 | 0.27 | -1.86 | Putative Thioredoxin Reductase Putative |
| INV10400500 | 1788 | 5 | 20.79 | 0.28 | -1.86 | Induced During Competenceinduced |
| INV10417680 | 849 | 2 | 9.87 | 0.28 | -1.86 | Putative Membrane Protein Putative |
| INV10415970 | 537 | 1 | 6.24 | 0.28 | -1.86 | Putative Membrane Protein Putative |
| nusG | 537 | 1 | 6.24 | 0.28 | -1.86 | Rod Shape-Determining Protein Mrecrod |
| acpP | 225 | 0 | 2.62 | 0.28 | -1.86 | Acyl Carrier Protein (ACP)Acyl |
| INV10413670 | 1152 | 3 | 13.39 | 0.28 | -1.85 | Putative Uncharacterized Protein Putative |
| INV10417720 | 1152 | 3 | 13.39 | 0.28 | -1.85 | Putative Surface Anchored Protein (Pseudogene) Putative |
| Phageintegrase-site-specificrecombinase | 1149 | 3 | 13.36 | 0.28 | -1.84 | N-Ethylammeline Chlorohydrolasen-Ethylammeline |
| INV10411250 | 531 | 1 | 6.17 | 0.28 | -1.84 | Conserved Hypothetical Proteinconserved |
| Aspartyl-tRNAsynthetase(EC6 | 1764 | 5 | 20.51 | 0.28 | -1.84 | Putative L-Asparaginase Putative |
| INV10409190 | 222 | 0 | 2.58 | 0.28 | -1.84 | Putative Membrane Protein Putative |
| INV10417450 | 222 | 0 | 2.58 | 0.28 | -1.84 | Putative Transcriptional Regulator (Pseudogene) Putative |
| Phagereplicationinitiationprotein | 837 | 2 | 9.73 | 0.28 | -1.84 | N-Ethylammeline Chlorohydrolasen-Ethylammeline |
| glgC | 1143 | 3 | 13.29 | 0.28 | -1.84 | Galactose-1-Phosphate Uridylyltransferasegalactose-1-Phosphate |
| INV10418930 | 528 | 1 | 6.14 | 0.28 | -1.84 | Putative Exported Protein Putative |
| msmG | 835 | 2 | 9.71 | 0.28 | -1.84 | GTP-Binding Protein Lepagtp-Binding |
| INV10409720 | 1140 | 3 | 13.25 | 0.28 | -1.83 | Conserved Hypothetical Proteinconserved |
| ABCtransporter_ATP-bindingpermeaseprotein | 1749 | 5 | 20.33 | 0.28 | -1.83 | ABC Transporter, ATP-Binding/Permease Protein ABC |
| INV10416330 | 1443 | 4 | 16.78 | 0.28 | -1.83 | Hypothetical Protein (Pseudogene)Hypothetical |
| dnaN | 1137 | 3 | 13.22 | 0.28 | -1.83 | DNA-Cytosine Methyltransferase (EC 2.1.1.37)DNA-Cytosine |
| INV10400810 | 1137 | 3 | 13.22 | 0.28 | -1.83 | Putative Aminotransferase Putative |
| grpE | 525 | 1 | 6.1 | 0.28 | -1.83 | Glycerate Kinase (EC 2.7.1.31)Glycerate |
| INV10408930 | 219 | 0 | 2.55 | 0.28 | -1.83 | Lysr-Family Regulatory Proteinlysr-Family |
| nrdH | 219 | 0 | 2.55 | 0.28 | -1.83 | Mobile Element Proteinmobile |
| INV10419220 | 828 | 2 | 9.63 | 0.28 | -1.83 | Putative Surface Protein (Fragment) Putative |
| hemN | 1131 | 3 | 13.15 | 0.28 | -1.82 | Gp9gp9 |
| glgA | 1434 | 4 | 16.67 | 0.28 | -1.82 | Galactokinasegalactokinase |
| INV10409530 | 520 | 1 | 6.05 | 0.28 | -1.82 | Ferric Siderophore ABC Transporter, ATP-Binding Proteinferric |
| INV10408020 | 519 | 1 | 6.03 | 0.28 | -1.81 | Conserved Hypothetical Proteinconserved |
| galT | 1425 | 4 | 16.57 | 0.28 | -1.81 | Putative Cell Division Protein Putative |
| gnd | 1425 | 4 | 16.57 | 0.28 | -1.81 | Putative Lactoylglutathione Lyase Putative |
| INV10400940 | 1122 | 3 | 13.04 | 0.28 | -1.81 | Sugar Phosphotransferase System (PTS), Sorbose Subfamily, IIB Componentsugar |
| INV10408950 | 2027 | 6 | 23.57 | 0.28 | -1.81 | Putative RNA Pseudouridylate Synthase Putative |
| INV10405320 | 819 | 2 | 9.52 | 0.29 | -1.81 | Transcription Antiterminatortranscription |
| INV10410610 | 516 | 1 | 6 | 0.29 | -1.81 | Putative Membrane Protein (Pseudogene) Putative |
| INV10410360 | 816 | 2 | 9.49 | 0.29 | -1.81 | Putative Peptidase Putative |
| INV10417970 | 816 | 2 | 9.49 | 0.29 | -1.81 | Putative Exported Protein Putative |
| msrAB | 1113 | 3 | 12.94 | 0.29 | -1.8 | Leucyl-Trna Synthetaseleucyl-Trna |
| INV10408770 | 510 | 1 | 5.93 | 0.29 | -1.79 | ABC Transporter, Permease Protein ABC |
| AminoacidABCtransporter_aminoacid-bindingpermeaseprotein | 808 | 2 | 9.39 | 0.29 | -1.79 | Amino Acid ABC Transporter, Amino Acid-Binding/Permease Proteinamino |
| ribD | 1101 | 3 | 12.8 | 0.29 | -1.79 | Spermidine/Putrescine ABC Transporter Permease Proteinspermidine/Putrescine |
| INV10409000 | 210 | 0 | 2.44 | 0.29 | -1.78 | Putative Replication Initiation Protein (Pseudogene) Putative |
| INV10400230 | 801 | 2 | 9.31 | 0.29 | -1.78 | Hypothetical Proteinhypothetical |
| FIG01115429-hypotheticalprotein | 1095 | 3 | 12.73 | 0.29 | -1.78 | FIG01114468: Hypothetical Proteinfig01114468: |
| INV10410450 | 798 | 2 | 9.28 | 0.29 | -1.78 | Putative Cysteine Desulfurase Putative |
| INV10413770 | 798 | 2 | 9.28 | 0.29 | -1.78 | Putative NADPH-Dependent FMN Reductase Putative |
| FIG00628088-hypotheticalprotein | 1386 | 4 | 16.11 | 0.29 | -1.77 | 3-Oxoacyl-[Acyl-Carrier-Protein] Synthase III3-Oxoacyl-[Acyl-Carrier-Protein] |
| FIG01115415-hypotheticalprotein | 207 | 0 | 2.41 | 0.29 | -1.77 | FIG01114344: Hypothetical Proteinfig01114344: |
| rpmC | 207 | 0 | 2.41 | 0.29 | -1.77 | Putative Phosphoribosylaminoimidazole Carboxylase Catalytic Subunit Putative |
| INV10407140 | 498 | 1 | 5.79 | 0.29 | -1.76 | ABC Transporter ATP-Binding Protein ABC |
| INV10401220 | 1082 | 3 | 12.58 | 0.29 | -1.76 | Putative Uncharacterized Protein Putative |
| INV10403570 | 786 | 2 | 9.14 | 0.3 | -1.76 | Putative Glutathione Peroxidase Putative |
| INV10412920 | 495 | 1 | 5.75 | 0.3 | -1.75 | GDSL-Like Lipase/Acylhydrolase Family Proteingdsl-Like |
| Bacteriocin-likepeptideNBlpN | 204 | 0 | 2.37 | 0.3 | -1.75 | Bacteriocin, Putativebacteriocin, |
| N-acetylmannosaminekinase(EC2 | 204 | 0 | 2.37 | 0.3 | -1.75 | Membrane-Bound Protease, CAAX Familymembrane-Bound |
| putativetransposonexcisionaseTn916ORF1-like | 204 | 0 | 2.37 | 0.3 | -1.75 | Phenylalanyl-Trna Synthetase Alpha Chainphenylalanyl-Trna |
| INV10416400 | 1354 | 4 | 15.74 | 0.3 | -1.74 | Putative Membrane Protein (Pseudo) Putative |
| glgB | 1929 | 6 | 22.43 | 0.3 | -1.74 | Galactose Operon Repressorgalactose |
| INV10402060 | 777 | 2 | 9.03 | 0.3 | -1.74 | Putative ABC Transporter ATP-Binding Protein Putative |
| pnpA | 2214 | 7 | 25.74 | 0.3 | -1.74 | Orf17orf17 |
| atpE | 201 | 0 | 2.34 | 0.3 | -1.74 | ATP Synthase Epsilon Chainatp |
| rpmI | 201 | 0 | 2.34 | 0.3 | -1.74 | Putative Pur Operon Repressor Putative |
| thiD | 774 | 2 | 9 | 0.3 | -1.74 | 50S Ribosomal Protein L2350S |
| INV10400880 | 1059 | 3 | 12.31 | 0.3 | -1.73 | Putative Phosphoribosylformylglycinamidine Synthase Protein Putative |
| ABC_transporter_ATP-bindingprotein | 771 | 2 | 8.96 | 0.3 | -1.73 | ABC, Transporter, ATP-Binding Protein ABC, |
| INV10413010 | 1623 | 5 | 18.87 | 0.3 | -1.73 | Arsc Family Proteinarsc |
| greA | 483 | 1 | 5.62 | 0.3 | -1.73 | Glutamyl-Trna Synthetaseglutamyl-Trna |
| luxS | 483 | 1 | 5.62 | 0.3 | -1.73 | IS1167, Transposaseis1167, |
| phoR | 1332 | 4 | 15.49 | 0.3 | -1.72 | Putative N-Acetylneuraminate Lyase Putative |
| transcriptionregulator_probable | 479 | 1 | 5.57 | 0.3 | -1.72 | 30S Ribosomal Protein S1630S |
| INV10409290 | 1044 | 3 | 12.14 | 0.3 | -1.72 | Pneumococcal Histidine Triad Proteinpneumococcal |
| brnQ | 1326 | 4 | 15.42 | 0.3 | -1.72 | Putative Immunity Protein Blpx Putative |
| pyrG | 1608 | 5 | 18.69 | 0.3 | -1.71 | Putative Streptococcal Histidine Triad Protein Phpa Putative |
| INV10405090 | 759 | 2 | 8.82 | 0.31 | -1.71 | Trna (Guanine-N(7)-)-Methyltransferasetrna |
| parB | 759 | 2 | 8.82 | 0.31 | -1.71 | UDP-N-Acetylglucosamine 1-Carboxyvinyltransferase 1UDP-N-Acetylglucosamine |
| Undecaprenylpyrophosphatesynthetase(EC2 | 759 | 2 | 8.82 | 0.31 | -1.71 | Trigger Factor (Prolyl Isomerase)Trigger |
| INV10400510 | 477 | 1 | 5.55 | 0.31 | -1.71 | Translation Initiation Factor IF-1translation |
| FIG01114205-hypotheticalprotein | 195 | 0 | 2.27 | 0.31 | -1.71 | Formate--Tetrahydrofolate Ligase 1formate--Tetrahydrofolate |
| INV10400030 | 195 | 0 | 2.27 | 0.31 | -1.71 | Hypothetical Proteinhypothetical |
| INV10404760 | 756 | 2 | 8.79 | 0.31 | -1.71 | 6 Phospho Beta Galactosidase6 |
| 1947729_1948204 | 475 | 1 | 5.52 | 0.31 | -1.7 |  |
| IS1381_transposaseOrfB | 475 | 1 | 5.52 | 0.31 | -1.7 | Putative DNA-Binding Protein Putative |
| INV10400560 | 1036 | 3 | 12.04 | 0.31 | -1.7 | Integral Membrane Proteinintegral |
| ABCtransportermembrane-spanningpermease-macrolideefflux | 753 | 2 | 8.75 | 0.31 | -1.7 | ABC Transporter Membrane-Spanning Permease - Macrolide Effluxabc |
| INV10419370 | 753 | 2 | 8.75 | 0.31 | -1.7 | ABC Transporter ATP-Binding Protein ABC |
| INV10401500 | 1032 | 3 | 12 | 0.31 | -1.7 | Putative Trna (5-Methylaminomethyl-2-Thiouridylate)-Methyltransferase Putative |
| INV10411760 | 750 | 2 | 8.72 | 0.31 | -1.7 | Cutc Family Proteincutc |
| ribH | 468 | 1 | 5.44 | 0.31 | -1.69 | Manganese-Dependent Inorganic Pyrophosphatasemanganese-Dependent |
| comFA | 1299 | 4 | 15.1 | 0.31 | -1.69 | Putative Competence Protein Putative |
| cbpJ | 1020 | 3 | 11.86 | 0.31 | -1.68 | Choline Binding Protein Echoline |
| INV10414090 | 190 | 0 | 2.21 | 0.31 | -1.68 | Putative Uncharacterized Protein Putative |
| INV10416740 | 1017 | 3 | 11.82 | 0.31 | -1.68 | Transport System Permease Protein (Pseudogene)Transport |
| INV10418050 | 189 | 0 | 2.2 | 0.31 | -1.68 | Putative Hexulose-6-Phosphate Synthase Putative |
| rpmB | 189 | 0 | 2.2 | 0.31 | -1.68 | Phosphoribosylamine-Glycine Ligasephosphoribosylamine-Glycine |
| mecA | 738 | 2 | 8.58 | 0.31 | -1.68 | IS861, Transposase (Orf1), IS3 Family, Truncatedis861, |
| INV10401040 | 1011 | 3 | 11.75 | 0.31 | -1.67 | Putative Gene Fragment Putative |
| FIG01114344-hypotheticalprotein | 735 | 2 | 8.55 | 0.31 | -1.67 | FIG00627241: Hypothetical Proteinfig00627241: |
| galR | 1008 | 3 | 11.72 | 0.31 | -1.67 | Putative Putative Cell Division Protease Ftsh Putative |
| INV10408960 | 1008 | 3 | 11.72 | 0.31 | -1.67 | Initiation-Control Protein Yabainitiation-Control |
| mvaK2 | 1008 | 3 | 11.72 | 0.31 | -1.67 | Putative Mannose-Specific Phosphotransferase System (PTS), IIC Component Putative |
| INV10417830 | 1278 | 4 | 14.86 | 0.32 | -1.67 | Putative Transposase (Fragment) Putative |
| ctsR | 459 | 1 | 5.34 | 0.32 | -1.66 | Conserved Domain Proteinconserved |
| INV10416620 | 459 | 1 | 5.34 | 0.32 | -1.66 | Siderophore Uptake ATP-Binding Proteinsiderophore |
| INV10410430 | 1275 | 4 | 14.82 | 0.32 | -1.66 | Conserved Hypothetical Proteinconserved |
| carB | 3177 | 11 | 36.94 | 0.32 | -1.66 | Calcium-Binding Protein, Putativecalcium-Binding |
| malX | 1272 | 4 | 14.79 | 0.32 | -1.66 | IS1381, Transposase Orfbis1381, |
| INV10403330 | 996 | 3 | 11.58 | 0.32 | -1.65 | Acetyltransferase (GNAT) Family Proteinacetyltransferase |
| pbuX | 1263 | 4 | 14.68 | 0.32 | -1.65 | Putative Peptidoglycan Pentaglycine Interpeptide Biosynthesis Protein Putative |
| plsX | 993 | 3 | 11.54 | 0.32 | -1.65 | Transcription Antitermination Factortranscription |
| INV10407850 | 1260 | 4 | 14.65 | 0.32 | -1.65 | Conserved Hypothetical Proteinconserved |
| rpmD | 183 | 0 | 2.13 | 0.32 | -1.65 | Putative Amidophosphoribosyltransferase Precursor Putative |
| rpmF | 183 | 0 | 2.13 | 0.32 | -1.65 | Putative Phosphoribosylaminoimidazole Carboxylase Atpase Subunit Putative |
| S-adenosylmethionine-dependentmethyltransferase | 183 | 0 | 2.13 | 0.32 | -1.65 | Putative Redox-Sensing Transcriptional Repressor Putative |
| malR | 987 | 3 | 11.47 | 0.32 | -1.64 | IS1381, Transposase Orfbis1381, |
| INV10402080 | 981 | 3 | 11.41 | 0.32 | -1.63 | Putative Membrane Protein Putative |
| Phageantirepressorprotein | 714 | 2 | 8.3 | 0.32 | -1.63 | N-Ethylammeline Chlorohydrolasen-Ethylammeline |
| rplM | 447 | 1 | 5.2 | 0.32 | -1.63 | Phosphoenolpyruvate-Protein Phosphotransferasephosphoenolpyruvate-Protein |
| FIG01116413-hypotheticalprotein | 180 | 0 | 2.09 | 0.32 | -1.63 | FIG01115429: Hypothetical Proteinfig01115429: |
| pncM | 180 | 0 | 2.09 | 0.32 | -1.63 | Oligopeptide Transport ATP-Binding Protein Oppd (TC 3.A.1.5.1)Oligopeptide |
| deoD | 711 | 2 | 8.27 | 0.32 | -1.63 | Degenerate Transposasedegenerate |
| INV10412910 | 1506 | 5 | 17.51 | 0.32 | -1.63 | Conserved Hypothetical Proteinconserved |
| pyk | 1506 | 5 | 17.51 | 0.32 | -1.63 | Alkaline Phosphatase Synthesis Transcriptional Regulatory Proteinalkaline |
| INV10400190 | 444 | 1 | 5.16 | 0.32 | -1.62 | Hypothetical Proteinhypothetical |
| INV10411190 | 444 | 1 | 5.16 | 0.32 | -1.62 | Putative Small-Molecule-Binding Protein Putative |
| INV10417810 | 1236 | 4 | 14.37 | 0.33 | -1.62 | Putative IS630-Spn1 Transposase (Pseudogene) Putative |
| Glycerophosphoryldiesterphosphodiesterase(EC3 | 1764 | 6 | 20.51 | 0.33 | -1.62 | Putative Glutamine ABC Transporter, ATP-Binding Protein 1 Putative |
| pcrA | 2292 | 8 | 26.65 | 0.33 | -1.62 | UDP-N-Acetylglucosamine 1-Carboxyvinyltransferase 2UDP-N-Acetylglucosamine |
| INV10403250 | 1233 | 4 | 14.33 | 0.33 | -1.62 | Sugar Phosphotransferase System (PTS), Lactose/Cellobiose-Specific Family, IIA Componentsugar |
| pdhA | 969 | 3 | 11.27 | 0.33 | -1.62 | Mutator Mutt Protein (7,8-Dihydro-8-Oxoguanine-Triphosphatase) (EC 3.6.1.-)Mutator |
| INV10406660 | 441 | 1 | 5.13 | 0.33 | -1.62 | Putative Glycosyl Hydrolase Putative |
| pepN | 2547 | 9 | 29.61 | 0.33 | -1.61 | Mutt/Nudix Family Proteinmutt/Nudix |
| INV10409310 | 177 | 0 | 2.06 | 0.33 | -1.61 | Putative Uncharacterized Protein (Pseudogene) Putative |
| INV10417290 | 177 | 0 | 2.06 | 0.33 | -1.61 | Putative Uncharacterized Protein Putative |
| rpsU | 177 | 0 | 2.06 | 0.33 | -1.61 | Recombination Protein Recrrecombination |
| INV10403790 | 439 | 1 | 5.1 | 0.33 | -1.61 | Putative N-Acetylgalactosamine-Specific Phosphotransferase System (PTS), IIA Component Putative |
| INV10400270 | 438 | 1 | 5.09 | 0.33 | -1.61 | Hypothetical Proteinhypothetical |
| INV10404000 | 1224 | 4 | 14.23 | 0.33 | -1.61 | Cell Wall Surface Anchored Proteincell |
| INV10403320 | 699 | 2 | 8.13 | 0.33 | -1.61 | Putative GNAT-Family Acetyltransferase Putative |
| INV10406620 | 699 | 2 | 8.13 | 0.33 | -1.61 | Putative Exported Protein Putative |
| INV10417170 | 1215 | 4 | 14.13 | 0.33 | -1.6 | Putative Membrane Protein Putative |
| FIG01115489-hypotheticalprotein | 174 | 0 | 2.02 | 0.33 | -1.59 | FIG01114476: Hypothetical Proteinfig01114476: |
| INV10415780 | 693 | 2 | 8.06 | 0.33 | -1.59 | Ribosomal Protein L11 Methyltransferaseribosomal |
| Transcriptionalantiterminatoroflichenanoperon_BglGfamily | 1464 | 5 | 17.02 | 0.33 | -1.59 | 30S Ribosomal Protein S730S |
| INV10412890 | 429 | 1 | 4.99 | 0.33 | -1.58 | Putative Beta-Lactamase Putative |
| INV10417460 | 429 | 1 | 4.99 | 0.33 | -1.58 | Putative Secreted Protein Putative |
| INV10405610 | 171 | 0 | 1.99 | 0.33 | -1.58 | Putative Glutamine Transporter, ATP-Binding Protein (Pseudogene) Putative |
| INV10416690 | 171 | 0 | 1.99 | 0.33 | -1.58 | Sugar Phosphotransferase System (PTS), IIABC Componentsugar |
| INV10401510 | 684 | 2 | 7.95 | 0.34 | -1.58 | Mutt/NUDIX Hydrolase Family Proteinmutt/NUDIX |
| INV10416950 | 684 | 2 | 7.95 | 0.34 | -1.58 | Putative NADP-Dependent L-Serine/L-Allo-Threonine Dehydrogenase Putative |
| PeptidemethioninesulfoxidereductaseMsrA(EC1 | 939 | 3 | 10.92 | 0.34 | -1.58 | Mevalonate Kinasemevalonate |
| ptsK | 936 | 3 | 10.88 | 0.34 | -1.57 | Putative CDP-Diacylglycerol--Glycerol-3-Phosphate 3-Phosphatidyltransferase Putative |
| fruA | 1953 | 7 | 22.71 | 0.34 | -1.57 | FIG01120216: Hypothetical Proteinfig01120216: |
| FIG01116379-hypotheticalprotein | 168 | 0 | 1.95 | 0.34 | -1.56 | FIG01115377: Hypothetical Proteinfig01115377: |
| pbp2A | 2196 | 8 | 25.53 | 0.34 | -1.56 | Putative UDP-N-Acetylmuramoyl-Tripeptide--D-Alanyl-D-Alanine Ligase Putative |
| INV10409950 | 1179 | 4 | 13.71 | 0.34 | -1.56 | Putative DNA_Binding Protein Putative |
| INV10413250 | 2697 | 10 | 31.36 | 0.34 | -1.56 | Putative RNA Helicase Putative |
| Sialicacid-inducedtransmembraneproteinYjhT(NanM)_possiblemutarotase | 924 | 3 | 10.74 | 0.34 | -1.55 | Ribonuclease Iiiribonuclease |
| fsaA | 669 | 2 | 7.78 | 0.34 | -1.55 | FIG086557: Conjugation Related Proteinfig086557: |
| Phageendolysin | 417 | 1 | 4.85 | 0.34 | -1.55 | N-Ethylammeline Chlorohydrolasen-Ethylammeline |
| glyQ | 918 | 3 | 10.67 | 0.34 | -1.54 | Putative Putative Glutamine ABC Transporter, ATP-Binding Protein 2 Putative |
| TetracyclineresistanceproteinTetM | 1920 | 7 | 22.32 | 0.34 | -1.54 | 50S Ribosomal Protein L2150S |
| ply | 1416 | 5 | 16.46 | 0.34 | -1.54 | Putative Methylated-DNA--Protein-Cysteine Methyltransferase Putative |
| rplP | 414 | 1 | 4.81 | 0.34 | -1.54 | Peptidyl-Trna Hydrolasepeptidyl-Trna |
| rpsL | 414 | 1 | 4.81 | 0.34 | -1.54 | S-Adenosylmethionine:Trna Ribosyltransferase-Isomerases-Adenosylmethionine:Trna |
| rpoC | 3651 | 14 | 42.45 | 0.35 | -1.53 | Putative Transposon Excisionase; Tn916 ORF1-Like Putative |
| atpD | 1407 | 5 | 16.36 | 0.35 | -1.53 | ATP Synthase A Chainatp |
| INV10403200 | 1158 | 4 | 13.46 | 0.35 | -1.53 | Deor Family Regulatory Proteindeor |
| INV10401590 | 411 | 1 | 4.78 | 0.35 | -1.53 | Acetyltransferase (GNAT) Family Proteinacetyltransferase |
| INV10404380 | 411 | 1 | 4.78 | 0.35 | -1.53 | Hypothetical Proteinhypothetical |
| INV10409030 | 162 | 0 | 1.88 | 0.35 | -1.53 | Putative Uncharacterized Protein (Pseudogene) Putative |
| INV10405020 | 657 | 2 | 7.64 | 0.35 | -1.53 | Conserved Hypothetical Proteinconserved |
| INV10400900 | 904 | 3 | 10.51 | 0.35 | -1.52 | Putative Beta-Galactosidase Putative |
| Cytidinedeaminase(EC3 | 408 | 1 | 4.74 | 0.35 | -1.52 | O-Acetylhomoserine (Thiol)-Lyase (Pseudogene)O-Acetylhomoserine |
| INV10417110 | 900 | 3 | 10.46 | 0.35 | -1.52 | Marr Family Regulatory Protein (Pseudogene)Marr |
| INV10411750 | 1635 | 6 | 19.01 | 0.35 | -1.52 | Putative Conserved Hypothetical Protein Putative |
| FIG01115840-hypotheticalprotein | 159 | 0 | 1.85 | 0.35 | -1.51 | FIG01114768: Hypothetical Proteinfig01114768: |
| FIG01117752-hypotheticalprotein | 159 | 0 | 1.85 | 0.35 | -1.51 | FIG01115816: Hypothetical Proteinfig01115816: |
| INV10412520 | 894 | 3 | 10.39 | 0.35 | -1.51 | Putative Exported Protein Putative |
| INV10412960 | 3099 | 12 | 36.03 | 0.35 | -1.51 | Spou Rrna Methylase Family Proteinspou |
| INV10405000 | 648 | 2 | 7.53 | 0.35 | -1.51 | Putative Membrane Protein Putative |
| INV10408030 | 648 | 2 | 7.53 | 0.35 | -1.51 | Putative Type I RM Modification Enzyme Putative |
| INV10405010 | 1380 | 5 | 16.04 | 0.35 | -1.51 | Putative Membrane Protein Putative |
| INV10404290 | 1130 | 4 | 13.14 | 0.35 | -1.5 | Putative C4-Dicarboxylate Transporter Putative |
| INV10407860 | 1128 | 4 | 13.11 | 0.35 | -1.5 | Putative 30S Ribosomal Protein S1 Putative |
| ABCtransporter_ATP-bindingprotein | 885 | 3 | 10.29 | 0.35 | -1.5 | ABC Transporter, ATP-Binding Protein ABC |
| miaA | 885 | 3 | 10.29 | 0.35 | -1.5 | PTS System, Lactose-Specific IIA Componentpts |
| TranscriptionalregulatorSpxA2 | 399 | 1 | 4.64 | 0.35 | -1.5 | 30S Ribosomal Protein S1530S |
| INV10409010 | 156 | 0 | 1.81 | 0.36 | -1.49 | Putative IS1381 Transposase (Pseudogene) Putative |
| pncG | 156 | 0 | 1.81 | 0.36 | -1.49 | Oligopeptide ABC Transporter, Periplasmic Oligopeptide-Binding Protein Oppa (TC 3.A.1.5.1)Oligopeptide |
| INV10410780 | 398 | 1 | 4.63 | 0.36 | -1.49 | Putative Phage Integrase (Pseudogene) Putative |
| INV10402040 | 1123 | 4 | 13.06 | 0.36 | -1.49 | ABC Transporter, Substrate Binding Protein ABC |
| INV10404720 | 396 | 1 | 4.6 | 0.36 | -1.49 | ROK Family Regulatorrok |
| INV10406060 | 396 | 1 | 4.6 | 0.36 | -1.49 | Putative Sodium Hydrogen Exchange Transporter Putative |
| INV10403340 | 636 | 2 | 7.39 | 0.36 | -1.48 | Luciferase-Like Monooxygenaseluciferase-Like |
| INV10419100 | 636 | 2 | 7.39 | 0.36 | -1.48 | Putative Uncharacterized Protein Putative |
| INV10400830 | 633 | 2 | 7.36 | 0.36 | -1.48 | Putative IS1381 Transposase (Pseudogene) Putative |
| pgi | 1350 | 5 | 15.7 | 0.36 | -1.48 | N-Ethylammeline Chlorohydrolasen-Ethylammeline |
| INV10401000 | 153 | 0 | 1.78 | 0.36 | -1.48 | Putative Aldose 1-Epimerase Putative |
| 2-hydroxy-3-oxopropionatereductase(EC1 | 870 | 3 | 10.11 | 0.36 | -1.47 | 2-Hydroxy-3-Oxopropionate Reductase (EC 1.1.1.60)2-Hydroxy-3-Oxopropionate |
| INV10406220 | 870 | 3 | 10.11 | 0.36 | -1.47 | Lysr Family Regulatory Proteinlysr |
| gdh | 1347 | 5 | 15.66 | 0.36 | -1.47 | Putative L-Fucose Isomerase Putative |
| lepA | 1824 | 7 | 21.21 | 0.36 | -1.47 | ABC Transporter ATP-Binding Protein ABC |
| INV10413680 | 630 | 2 | 7.32 | 0.36 | -1.47 | Conserved Hypothetical Proteinconserved |
| INV10413980 | 630 | 2 | 7.32 | 0.36 | -1.47 | Putative Exported Protein Putative |
| INV10412690 | 1107 | 4 | 12.87 | 0.36 | -1.47 | Putative DNA Modification Methylase Putative |
| INV10406790 | 868 | 3 | 10.09 | 0.36 | -1.47 | Branched-Chain Amino Acid Transport System Permease Proteinbranched-Chain |
| accD | 867 | 3 | 10.08 | 0.36 | -1.47 | Acetyl-Coenzyme A Carboxylase Carboxyl Transferase Subunit Betaacetyl-Coenzyme |
| INV10414320 | 867 | 3 | 10.08 | 0.36 | -1.47 | Hypothetical Proteinhypothetical |
| Bacteriocin-likepeptideOBlpO | 152 | 0 | 1.77 | 0.36 | -1.47 | Bacteriocin-Like Peptide M Blpmbacteriocin-Like |
| INV10402020 | 627 | 2 | 7.29 | 0.36 | -1.47 | Putative Transport Protein Putative |
| INV10416470 | 627 | 2 | 7.29 | 0.36 | -1.47 | Thioesterase Superfamily Proteinthioesterase |
| INV10409650 | 864 | 3 | 10.04 | 0.36 | -1.46 | Putative Uncharacterized Protein Putative |
| INV10405480 | 861 | 3 | 10.01 | 0.36 | -1.46 | ABC Transporter, Permease Protein ABC |
| INV10405640 | 624 | 2 | 7.25 | 0.36 | -1.46 | Metallo-Beta-Lactamase Superfamily Proteinmetallo-Beta-Lactamase |
| rplD | 624 | 2 | 7.25 | 0.36 | -1.46 | Putative Ribose-Phosphate Pyrophosphokinase 2 Putative |
| rplQ | 387 | 1 | 4.5 | 0.36 | -1.46 | Histidine-Containing Phosphocarrier Protein (Hpr)Histidine-Containing |
| blpX | 385 | 1 | 4.48 | 0.37 | -1.45 | Response Regulator Protein Blprresponse |
| OligopeptidetransportsystempermeaseproteinOppC(TC3 | 852 | 3 | 9.91 | 0.37 | -1.45 | Arac Family Regulatory Proteinarac |
| addA | 3651 | 15 | 42.45 | 0.37 | -1.44 | Putative ATP-Dependent Exonuclease Subunit A Putative |
| FIG01115961-hypotheticalprotein | 381 | 1 | 4.43 | 0.37 | -1.44 | FIG01114872: Hypothetical Proteinfig01114872: |
| INV10409920 | 381 | 1 | 4.43 | 0.37 | -1.44 | Putative Lantibiotic Export Protein (Pseudogene) Putative |
| INV10413390 | 381 | 1 | 4.43 | 0.37 | -1.44 | Putative Membrane Protein Putative |
| INV10415580 | 381 | 1 | 4.43 | 0.37 | -1.44 | Cora-Like Mg2+ Transporter Proteincora-Like |
| gp5 | 147 | 0 | 1.71 | 0.37 | -1.44 | Glpg Protein (Membrane Protein Of Glp Regulon)Glpg |
| Phosphoenolpyruvate-dihydroxyacetonephosphotransferase(EC2 | 147 | 0 | 1.71 | 0.37 | -1.44 | Oxygen-Insensitive NADPH Nitroreductaseoxygen-Insensitive |
| INV10406080 | 2244 | 9 | 26.09 | 0.37 | -1.44 | Putative Redoxin Putative |
| def | 612 | 2 | 7.12 | 0.37 | -1.44 | Cytidine/Deoxycytidylate Deaminase Family Proteincytidine/Deoxycytidylate |
| INV10401640 | 1539 | 6 | 17.89 | 0.37 | -1.43 | Putative Transposase (Pseudogene) Putative |
| INV10416610 | 609 | 2 | 7.08 | 0.37 | -1.43 | Putative Iron Compound ABC Transporter, Permease Protein Putative |
| INV10411170 | 375 | 1 | 4.36 | 0.37 | -1.42 | Putative Membrane Protein Putative |
| Inducedduringcompetence | 144 | 0 | 1.67 | 0.37 | -1.42 | Homoserine Dehydrogenasehomoserine |
| IS1167_transposase | 144 | 0 | 1.67 | 0.37 | -1.42 | Putative Reverse Transcriptase - Group II Intron (Pseudogene) Putative |
| rpoE | 603 | 2 | 7.01 | 0.37 | -1.42 | Putative Pyruvate Kinase Putative |
| pflD | 2439 | 10 | 28.36 | 0.37 | -1.42 | N-Acetylneuraminate Lyase (EC 4.1.3.3)N-Acetylneuraminate |
| nrdD | 2208 | 9 | 25.67 | 0.37 | -1.42 | Mobile Element Proteinmobile |
| INV10406070 | 372 | 1 | 4.32 | 0.38 | -1.41 | Ribonuclease BN-Like Family Proteinribonuclease |
| rnpA | 372 | 1 | 4.32 | 0.38 | -1.41 | Putative Pyrroline-5-Carboxylate Reductase Putative |
| INV10418630 | 3117 | 13 | 36.24 | 0.38 | -1.41 | Putative Uncharacterized Protein Putative |
| INV10407000 | 1053 | 4 | 12.24 | 0.38 | -1.4 | Vanz Like Family Proteinvanz |
| rmlB | 1050 | 4 | 12.21 | 0.38 | -1.4 | Peptide Chain Release Factor 3peptide |
| INV10414070 | 2412 | 10 | 28.04 | 0.38 | -1.4 | Putative Methyltransferase Putative |
| INV10401660 | 141 | 0 | 1.64 | 0.38 | -1.4 | Putative Glycosyl Transferase (Pseudogene) Putative |
| ruvA | 594 | 2 | 6.91 | 0.38 | -1.4 | Regulatory Protein Recxregulatory |
| INV10410710 | 819 | 3 | 9.52 | 0.38 | -1.4 | Putative Membrane Transport Protein Putative |
| mreC | 819 | 3 | 9.52 | 0.38 | -1.4 | Large Subunit Ribosomal RNA; Lsurna; LSU Rrnalarge |
| INV10411110 | 366 | 1 | 4.26 | 0.38 | -1.4 | Conserved Hypothetical Proteinconserved |
| INV10414610 | 366 | 1 | 4.26 | 0.38 | -1.4 | Putative Membrane Protein Putative |
| acoC | 1044 | 4 | 12.14 | 0.38 | -1.39 | E2 Component Of Acetoin Dehydrogenase Enzyme System (Dihydrolipoamide Acetyltransferase)E2 |
| INV10402430 | 814 | 3 | 9.46 | 0.38 | -1.39 | Putative Membrane Protein Putative |
| INV10417630 | 363 | 1 | 4.22 | 0.38 | -1.38 | Rmuc Family Proteinrmuc |
| INV10410960 | 812 | 3 | 9.44 | 0.38 | -1.38 | Putative Transposase (Pseudogene) Putative |
| ftsZ | 1260 | 5 | 14.65 | 0.38 | -1.38 | Dihydropteroate Synthasedihydropteroate |
| murZ | 1260 | 5 | 14.65 | 0.38 | -1.38 | S-Ribosylhomocysteinases-Ribosylhomocysteinase |
| S-adenosylhomocysteinedeaminase(EC3 | 1260 | 5 | 14.65 | 0.38 | -1.38 | GTP Pyrophosphokinasegtp |
| FIG01114476-hypotheticalprotein | 138 | 0 | 1.6 | 0.38 | -1.38 | FIG00627694: Hypothetical Proteinfig00627694: |
| INV10415380 | 1482 | 6 | 17.23 | 0.38 | -1.38 | Putative Uncharacterized Protein Putative |
| aroF | 1032 | 4 | 12 | 0.38 | -1.38 | Shikimate 5-Dehydrogenaseshikimate |
| INV10418740 | 807 | 3 | 9.38 | 0.39 | -1.38 | ABC Transporter Permease Protein ABC |
| N-ethylammelinechlorohydrolase | 582 | 2 | 6.77 | 0.39 | -1.37 | Membrane Proteins Related To Metalloendopeptidasesmembrane |
| INV10404770 | 804 | 3 | 9.35 | 0.39 | -1.37 | PTS Transporter, IIBPTS |
| INV10415690 | 1692 | 7 | 19.67 | 0.39 | -1.37 | Putative Membrane Protein Putative |
| ilvC | 1023 | 4 | 11.89 | 0.39 | -1.37 | Hit Family Proteinhit |
| INV10410920 | 1023 | 4 | 11.89 | 0.39 | -1.37 | Pneumococcal Histidine Triad Proteinpneumococcal |
| INV10412440 | 357 | 1 | 4.15 | 0.39 | -1.36 | Putative Myo-Inositol-1(Or 4)-Monophosphatase Putative |
| INV10409320 | 135 | 0 | 1.57 | 0.39 | -1.36 | Gtra-Like Proteingtra-Like |
| Membraneproteinsrelatedtometalloendopeptidases | 576 | 2 | 6.7 | 0.39 | -1.36 | Fes Assembly Nifu-Like Proteinfes |
| fabF | 1236 | 5 | 14.37 | 0.39 | -1.36 | Elongation Factor P (EF-P)Elongation |
| INV10419200 | 795 | 3 | 9.24 | 0.39 | -1.36 | C4-Dicarboxylate Anaerobic Carrier Proteinc4-Dicarboxylate |
| INV10411770 | 1455 | 6 | 16.92 | 0.39 | -1.36 | Putative Membrane Protein Putative |
| INV10415950 | 354 | 1 | 4.12 | 0.39 | -1.36 | Putative Transglysylase-Associated Membrane Protein Putative |
| FIG01114146-hypotheticalprotein | 573 | 2 | 6.66 | 0.39 | -1.35 | Signal Recognition Particle Proteinsignal |
| INV10400860 | 573 | 2 | 6.66 | 0.39 | -1.35 | Bacteriocin Transport/Processing ATP-Binding Proteinbacteriocin |
| INV10413240 | 792 | 3 | 9.21 | 0.39 | -1.35 | Polysaccharide Deacetylase Family Proteinpolysaccharide |
| INV10415770 | 1227 | 5 | 14.27 | 0.39 | -1.35 | Conserved Hypothetical Proteinconserved |
| INV10410910 | 789 | 3 | 9.17 | 0.39 | -1.35 | Sugar Phosphotransferase System (PTS), Fructose Family, IIA Componentsugar |
| lgt | 789 | 3 | 9.17 | 0.39 | -1.35 | Conserved Hypothetical Proteinconserved |
| rbfA | 351 | 1 | 4.08 | 0.39 | -1.34 | Pneumolysin (Thiol-Activated Cytolysin)Pneumolysin |
| INV10417380 | 132 | 0 | 1.53 | 0.39 | -1.34 | Lytr Family Regulatory Proteinlytr |
| INV10404670 | 785 | 3 | 9.13 | 0.4 | -1.34 | Glutamine ABC Transporter, Glutamine-Binding Protein/Permease Proteinglutamine |
| INV10406750 | 564 | 2 | 6.56 | 0.4 | -1.33 | Conserved Hypothetical Proteinconserved |
| deoB | 1212 | 5 | 14.09 | 0.4 | -1.33 | D-Alanine--D-Alanine Ligased-Alanine--D-Alanine |
| efp | 561 | 2 | 6.52 | 0.4 | -1.33 | Putative Primosomal Protein Putative |
| FIG01117889-hypotheticalprotein | 345 | 1 | 4.01 | 0.4 | -1.32 | FIG01115840: Hypothetical Proteinfig01115840: |
| FIG139598-Potentialribosomalprotein | 345 | 1 | 4.01 | 0.4 | -1.32 | FIG01116966: Hypothetical Proteinfig01116966: |
| INV10401080 | 1207 | 5 | 14.03 | 0.4 | -1.32 | Putative Uncharacterized Protein Putative |
| INV10418660 | 774 | 3 | 9 | 0.4 | -1.32 | Degenerate Transposasedegenerate |
| recO | 771 | 3 | 8.96 | 0.4 | -1.32 | ABC Transporter ATP Binding Domain Pncp (CAAX Protease)ABC |
| sufC | 771 | 3 | 8.96 | 0.4 | -1.32 | 50S Ribosomal Protein L1650S |
| clpE | 2259 | 10 | 26.26 | 0.4 | -1.31 | Cina-Like Proteincina-Like |
| INV10419340 | 340 | 1 | 3.95 | 0.4 | -1.31 | PTS System, Iia Componentpts |
| ABC-typemultidrugtransportsystem_permeasecomponent | 766 | 3 | 8.91 | 0.4 | -1.31 | ABC-Type Multidrug Transport System, Permease Componentabc-Type |
| INV10400540 | 339 | 1 | 3.94 | 0.4 | -1.3 | Integral Membrane Proteinintegral |
| INV10406490 | 339 | 1 | 3.94 | 0.4 | -1.3 | Amino-Acid ABC Transporter Integral Membrane Proteinamino-Acid |
| INV10411680 | 339 | 1 | 3.94 | 0.4 | -1.3 | Putative 3-Isopropylmalate Dehydratase Small Subunit Putative |
| INV10412990 | 1401 | 6 | 16.29 | 0.4 | -1.3 | ABC Transporter ATP-Binding Protein ABC |
| comC2 | 126 | 0 | 1.46 | 0.41 | -1.3 | Phosphopantetheine Adenylyltransferasephosphopantetheine |
| putativemethyltransferase | 126 | 0 | 1.46 | 0.41 | -1.3 | Phage Transcriptional Regulator, Cro/CI Familyphage |
| INV10403490 | 549 | 2 | 6.38 | 0.41 | -1.3 | Putative Permease Putative |
| INV10406720 | 2451 | 11 | 28.5 | 0.41 | -1.3 | Putative Fatty-Acid Binding Protein Putative |
| pstB1 | 759 | 3 | 8.82 | 0.41 | -1.3 | Xaa-Pro Dipeptidyl-Peptidasexaa-Pro |
| INV10402750 | 336 | 1 | 3.91 | 0.41 | -1.3 | Putative Regulator Putative |
| ADP-ribosepyrophosphatase(EC3 | 546 | 2 | 6.35 | 0.41 | -1.29 | ADP-Ribose Pyrophosphatase (EC 3.6.1.13)ADP-Ribose |
| INV10401110 | 546 | 2 | 6.35 | 0.41 | -1.29 | Cell Wall Surface Anchor Family Protein (Pseudogene)Cell |
| pgsA | 546 | 2 | 6.35 | 0.41 | -1.29 | N-Ethylammeline Chlorohydrolasen-Ethylammeline |
| INV10407360 | 966 | 4 | 11.23 | 0.41 | -1.29 | Putative Peptidoglycan Biosynthesis Membrane Protein Putative |
| asnB | 963 | 4 | 11.2 | 0.41 | -1.29 | Aspartate-Semialdehyde Dehydrogenaseaspartate-Semialdehyde |
| hpt | 543 | 2 | 6.31 | 0.41 | -1.28 | GTP-Binding Protein Eragtp-Binding |
| INV10411020 | 543 | 2 | 6.31 | 0.41 | -1.28 | Putative Sugar Phosphotransferase System (PTS), IIB Component (Pseudogene) Putative |
| prsA2 | 960 | 4 | 11.16 | 0.41 | -1.28 | Peptide Methionine Sulfoxide Reductase Msra (EC 1.8.4.11) / Peptide Methionine Sulfoxide Reductase Msrb (EC 1.8.4.12)Peptide |
| INV10400090 | 123 | 0 | 1.43 | 0.41 | -1.28 | Hypothetical Proteinhypothetical |
| INV10413800 | 750 | 3 | 8.72 | 0.41 | -1.28 | Cation Transporting Atpasecation |
| INV10416700 | 957 | 4 | 11.13 | 0.41 | -1.28 | Gntr Family Regulatory Proteingntr |
| CompetenceproteinCoiA | 956 | 4 | 11.11 | 0.41 | -1.28 | Putative Late Competence Protein Putative |
| INV10402070 | 747 | 3 | 8.68 | 0.41 | -1.28 | Putative ABC Transporter System Permease Protein Putative |
| Degenerativetransposase | 330 | 1 | 3.84 | 0.41 | -1.28 | Dihydrodipicolinate Synthasedihydrodipicolinate |
| INV10411160 | 330 | 1 | 3.84 | 0.41 | -1.28 | Conserved Hypothetical Proteinconserved |
| INV10406520 | 537 | 2 | 6.24 | 0.41 | -1.27 | Putative Coenzyme Putative |
| atpA | 1365 | 6 | 15.87 | 0.41 | -1.27 | Aspartyl-Trna Synthetase (EC 6.1.1.12)Aspartyl-Trna |
| INV10400790 | 327 | 1 | 3.8 | 0.42 | -1.26 | Putative Coa-Binding Protein Putative |
| INV10416310 | 1977 | 9 | 22.98 | 0.42 | -1.26 | ABC Transporter, ATP-Binding/Permease Protein ABC |
| INV10417870 | 1768 | 8 | 20.55 | 0.42 | -1.26 | Putative Beta-Glucosidase Putative |
| INV10403190 | 528 | 2 | 6.14 | 0.42 | -1.25 | Putative Pyruvate Formate-Lyase Activating Enzyme Putative |
| aldB | 732 | 3 | 8.51 | 0.42 | -1.25 | Alpha-Acetolactate Decarboxylasealpha-Acetolactate |
| ppaC | 936 | 4 | 10.88 | 0.42 | -1.25 | Putative Penicillin-Binding Protein 1A Putative |
| 333456_354568 | 21112 | 103 | 245.45 | 0.42 | -1.24 |  |
| INV10406580 | 321 | 1 | 3.73 | 0.42 | -1.24 | Putative Coenzyme Putative |
| ORF17 | 321 | 1 | 3.73 | 0.42 | -1.24 | Peptide Methionine Sulfoxide Reductasepeptide |
| INV10416860 | 1332 | 6 | 15.49 | 0.42 | -1.24 | Putative RNA Methyltransferase Putative |
| INV10402370 | 723 | 3 | 8.41 | 0.43 | -1.23 | Putative Carboxypeptidase, Possible Microcin Immunity Protein Putative |
| dpr | 519 | 2 | 6.03 | 0.43 | -1.23 | DNA Primasedna |
| rimM | 519 | 2 | 6.03 | 0.43 | -1.23 | Preprotein Translocase Subunit Secg (TC 3.A.5.1.1)Preprotein |
| INV10412240 | 1722 | 8 | 20.02 | 0.43 | -1.22 | Putative Membrane Protein Putative |
| FIG00627241-hypotheticalprotein | 315 | 1 | 3.66 | 0.43 | -1.22 | Biotin Carboxyl Carrier Protein Of Acetyl-Coa Carboxylasebiotin |
| Glyceratekinase(EC2 | 1116 | 5 | 12.97 | 0.43 | -1.22 | Putative Glutamine Synthetase Putative |
| INV10412580 | 714 | 3 | 8.3 | 0.43 | -1.22 | Putative Membrane Protein Putative |
| INV10411120 | 1305 | 6 | 15.17 | 0.43 | -1.21 | Putative Formate/Nitrate Transport Protein Putative |
| INV10404830 | 510 | 2 | 5.93 | 0.43 | -1.21 | ABC Transporter ATP-Binding Protein ABC |
| INV10418820 | 906 | 4 | 10.53 | 0.43 | -1.21 | Putative DNA-Binding Protein (Pseudogene) Putative |
| murB | 906 | 4 | 10.53 | 0.43 | -1.21 | Lipid A Export ATP-Binding/Permease Protein Msbalipid |
| INV10417890 | 1695 | 8 | 19.71 | 0.43 | -1.2 | Putative Lactose-Specific Phosphotransferase System (PTS), IIA Component 2 Putative |
| INV10402110 | 1299 | 6 | 15.1 | 0.43 | -1.2 | Response Regulator Proteinresponse |
| vicR | 705 | 3 | 8.2 | 0.43 | -1.2 | Putative IS630-Spn1 Transposase (Pseudogene) Putative |
| rpsJ | 309 | 1 | 3.59 | 0.44 | -1.2 | Uracil Permeaseuracil |
| htpX | 900 | 4 | 10.46 | 0.44 | -1.2 | Inosine-5'-Monophosphate Dehydrogenaseinosine-5'-Monophosphate |
| INV10400580 | 111 | 0 | 1.29 | 0.44 | -1.2 | Integraseintegrase |
| acoL | 1686 | 8 | 19.6 | 0.44 | -1.19 | Dihydrolipoamide Dehydrogenasedihydrolipoamide |
| pepXP | 2274 | 11 | 26.44 | 0.44 | -1.19 | 3-Hydroxy-3-Methylglutaryl Coenzyme A Synthase3-Hydroxy-3-Methylglutaryl |
| INV10414350 | 700 | 3 | 8.14 | 0.44 | -1.19 | NIF3 (NGG1p Interacting Factor 3) Family Proteinnif3 |
| INV10405380 | 306 | 1 | 3.56 | 0.44 | -1.19 | Putative Uncharacterized Protein Putative |
| rplX | 306 | 1 | 3.56 | 0.44 | -1.19 | Adenylosuccinate Lyaseadenylosuccinate |
| gldA | 1089 | 5 | 12.66 | 0.44 | -1.19 | UDP-Glucose 4-Epimeraseudp-Glucose |
| INV10406630 | 501 | 2 | 5.82 | 0.44 | -1.18 | Putative Metal Transporting P-Type Atpase Putative |
| INV10410600 | 501 | 2 | 5.82 | 0.44 | -1.18 | Conserved Hypothetical Proteinconserved |
| INV10410310 | 305 | 1 | 3.55 | 0.44 | -1.19 | Putative DNA-Binding Protein Putative |
| INV10412260 | 696 | 3 | 8.09 | 0.44 | -1.18 | Putative Glycosyl Transferase Putative |
| Ribulose-phosphate3-epimerase(EC5 | 696 | 3 | 8.09 | 0.44 | -1.18 | Putative Inorganic Polyphosphate/ATP-NAD Kinase Putative |
| INV10409370 | 1278 | 6 | 14.86 | 0.44 | -1.18 | Conserved Hypothetical Proteinconserved |
| INV10406010 | 693 | 3 | 8.06 | 0.44 | -1.18 | Putative Radical SAM Superfamily Protein (Pseudogene) Putative |
| Metal-dependenthydrolaseYbeY_involvedinrRNAandorribosomematurationandassembly | 498 | 2 | 5.79 | 0.44 | -1.18 | Dimethyladenosine Transferasedimethyladenosine |
| gatC | 303 | 1 | 3.52 | 0.44 | -1.18 | Cell Division Protein Ftszcell |
| INV10403480 | 303 | 1 | 3.52 | 0.44 | -1.18 | Putative Haloacid Dehalogenase-Like Hydrolase Putative |
| polA | 2634 | 13 | 30.62 | 0.44 | -1.18 | Orf46Orf46 |
| INV10419180 | 495 | 2 | 5.75 | 0.44 | -1.17 | Putative Fucosidase Putative |
| pyrC | 1269 | 6 | 14.75 | 0.44 | -1.17 | Phosphatidate Cytidylyltransferase (EC 2.7.7.41)Phosphatidate |
| FIG01116089-hypotheticalprotein | 301 | 1 | 3.5 | 0.44 | -1.17 | FIG01114899: Hypothetical Proteinfig01114899: |
| INV10402270 | 3771 | 19 | 43.84 | 0.45 | -1.16 | Putative Membrane Protein Putative |
| potD | 1071 | 5 | 12.45 | 0.45 | -1.16 | Topoisomerase IV Subunit Btopoisomerase |
| INV10406820 | 684 | 3 | 7.95 | 0.45 | -1.16 | Branched-Chain Amino Acid Transport ATP-Binding Proteinbranched-Chain |
| INV10416280 | 1068 | 5 | 12.42 | 0.45 | -1.16 | Sortase-Sorted Surface Anchored Proteinsortase-Sorted |
| cysD | 1260 | 6 | 14.65 | 0.45 | -1.16 | Conserved Domain Proteinconserved |
| gla | 870 | 4 | 10.11 | 0.45 | -1.15 | Elongation Factor G (EF-G)Elongation |
| GlpGprotein(membraneproteinofglpregulon) | 678 | 3 | 7.88 | 0.45 | -1.15 | Glycerol Facilitator-Aquaporinglycerol |
| INV10401490 | 678 | 3 | 7.88 | 0.45 | -1.15 |  |
| INV10401610 | 1251 | 6 | 14.54 | 0.45 | -1.15 |  |
| INV10407020 | 1059 | 5 | 12.31 | 0.45 | -1.15 | Putative Cyclophilin Type Peptidyl-Prolyl Cis-Trans Isomerase Putative |
| fabE | 486 | 2 | 5.65 | 0.45 | -1.15 | Dihydrofolate Reductasedihydrofolate |
| INV10402720 | 294 | 1 | 3.42 | 0.45 | -1.14 | Acetyltransferase (GNAT) Family Proteinacetyltransferase |
| INV10404610 | 294 | 1 | 3.42 | 0.45 | -1.14 | Hypothetical Proteinhypothetical |
| FIG01116802-hypotheticalprotein | 672 | 3 | 7.81 | 0.45 | -1.14 | FIG01115489: Hypothetical Proteinfig01115489: |
| INV10414160 | 672 | 3 | 7.81 | 0.45 | -1.14 | Putative Aminotransferase Putative |
| Beta-1_3-glucosyltransferase | 861 | 4 | 10.01 | 0.45 | -1.14 | Bacteriocin-Like Peptide O Blpobacteriocin-Like |
| OligoendopeptidaseF(EC3 | 1803 | 9 | 20.96 | 0.46 | -1.13 | Multiple Sugar-Binding Protein Precursormultiple |
| rmlA | 858 | 4 | 9.98 | 0.46 | -1.13 | Peptide Chain Release Factor 2peptide |
| 2_3-butanedioldehydrogenase_R-alcoholforming_(R)-and(S)-acetoin-specific(EC1 | 1044 | 5 | 12.14 | 0.46 | -1.13 | 2,3-Butanediol Dehydrogenase, R-Alcohol Forming, (R)- And (S)-Acetoin-Specific (EC 1.1.1.4)2,3-Butanediol |
| INV10416500 | 102 | 0 | 1.19 | 0.46 | -1.13 | Cation Efflux Family Proteincation |
| INV10401170 | 666 | 3 | 7.74 | 0.46 | -1.13 | Binding-Protein-Dependent Transport System Membrane Proteinbinding-Protein-Dependent |
| rpiA | 666 | 3 | 7.74 | 0.46 | -1.13 | Prophage Lambdasa2, Site-Specific Recombinase, Phage Integrase Familyprophage |
| aroK | 477 | 2 | 5.55 | 0.46 | -1.13 | Phospho-2-Dehydro-3-Deoxyheptonate Aldolase, Tyr-Sensitivephospho-2-Dehydro-3-Deoxyheptonate |
| INV10400220 | 477 | 2 | 5.55 | 0.46 | -1.13 | Hypothetical Proteinhypothetical |
| INV10404370 | 288 | 1 | 3.35 | 0.46 | -1.12 | Marr Family Regulatory Proteinmarr |
| INV10414600 | 474 | 2 | 5.51 | 0.46 | -1.12 | Putative Membrane Protein (Pseudogene) Putative |
| INV10405060 | 660 | 3 | 7.67 | 0.46 | -1.12 | ABC Transporter Protein Ecsbabc |
| ftsH | 1959 | 10 | 22.78 | 0.46 | -1.11 | Methionyl-Trna Formyltransferasemethionyl-Trna |
| INV10418670 | 471 | 2 | 5.48 | 0.46 | -1.11 | ABC Transporter ATP-Binding Membrane Protein ABC |
| ssb | 471 | 2 | 5.48 | 0.46 | -1.11 | 50S Ribosomal Protein L1150S |
| bacteriocin_ putative | 285 | 1 | 3.31 | 0.46 | -1.11 | Putative ATP Synthase Delta Chain Putative |
| groES | 285 | 1 | 3.31 | 0.46 | -1.11 | Serine Hydroxymethyltransferaseserine |
| INV10418390 | 285 | 1 | 3.31 | 0.46 | -1.11 | Putative Competence Protein Putative |
| ung | 654 | 3 | 7.6 | 0.46 | -1.1 | Putative Transketolase Putative |
| INV10406170 | 468 | 2 | 5.44 | 0.47 | -1.1 | Putative Gtpase Putative |
| INV10410620 | 468 | 2 | 5.44 | 0.47 | -1.1 | Conserved Hypothetical Proteinconserved |
| INV10417310 | 837 | 4 | 9.73 | 0.47 | -1.1 | Putative Uncharacterized Protein Putative |
| arcB | 1017 | 5 | 11.82 | 0.47 | -1.1 | Ornithine Carbamoyltransferaseornithine |
| INV10410490 | 648 | 3 | 7.53 | 0.47 | -1.09 | Adenylate Cyclase Family Proteinadenylate |
| purR | 828 | 4 | 9.63 | 0.47 | -1.09 | Phage Terminase Large Subunitphage |
| pcp | 645 | 3 | 7.5 | 0.47 | -1.09 | Putative Peptidoglycan Branched Peptide Synthesis Protein Putative |
| adhE | 2652 | 14 | 30.83 | 0.47 | -1.09 | Aldehyde-Alcohol Dehydrogenase 2 [Includes: Alcohol Dehydrogenase; Acetaldehyde Dehydrogenase]Aldehyde-Alcohol |
| INV10416820 | 279 | 1 | 3.24 | 0.47 | -1.08 | Sucrose Phosphorylasesucrose |
| ftsA | 1374 | 7 | 15.97 | 0.47 | -1.08 | FIG139598: Potential Ribosomal Proteinfig139598: |
| pfk | 1008 | 5 | 11.72 | 0.47 | -1.08 | N-Acetylglucosamine-1-Phosphate Uridyltransferase (EC 2.7.7.23) / Glucosamine-1-Phosphate N-Acetyltransferase (EC 2.3.1.157)N-Acetylglucosamine-1-Phosphate |
| ABCtransporterATP-bindingprotein | 642 | 3 | 7.46 | 0.47 | -1.08 | ABC Transporter ATP-Binding Protein ABC |
| DNAreplicationproteinDnaC | 642 | 3 | 7.46 | 0.47 | -1.08 | Replication Initiation And Membrane Attachment Proteinreplication |
| accC | 1368 | 7 | 15.9 | 0.47 | -1.08 | Biotin Carboxylase Subunit Of Acetyl-Coa Carboxylasebiotin |
| ezrA | 1728 | 9 | 20.09 | 0.47 | -1.08 | Putative Pantothenate Metabolism Flavoprotein Putative |
| INV10415660 | 1002 | 5 | 11.65 | 0.47 | -1.08 | Putative Mechanosensitive Ion Channel Protein Putative |
| hlpA | 276 | 1 | 3.21 | 0.48 | -1.07 | Transcription Elongation Factortranscription |
| INV10409660 | 1181 | 6 | 13.73 | 0.48 | -1.07 | Putative Uncharacterized Protein Putative |
| trpE | 1362 | 7 | 15.83 | 0.48 | -1.07 | Beta-N-Acetylhexosaminidase Precursor (Ec 3.2.1.52) (Sortase-Sorted)Beta-N-Acetylhexosaminidase |
| INV10406410 | 1542 | 8 | 17.93 | 0.48 | -1.07 | Putative Membrane Protein Putative |
| INV10404580 | 636 | 3 | 7.39 | 0.48 | -1.07 | Putative Phosphatase Putative |
| INV10415300 | 453 | 2 | 5.27 | 0.48 | -1.06 | ATP-Binding Proteinatp-Binding |
| rplI | 453 | 2 | 5.27 | 0.48 | -1.06 | Phosphate Import ATP-Binding Protein 2phosphate |
| Ferrochelatase_protohemeferro-lyase(EC4 | 273 | 1 | 3.17 | 0.48 | -1.06 | Putative Competence Associated Protein Putative |
| agaS | 1167 | 6 | 13.57 | 0.48 | -1.06 | Putative Tagatose-6-Phosphate Aldose/Ketose Isomerase Putative |
| INV10407310 | 1167 | 6 | 13.57 | 0.48 | -1.06 | Putative PEP-Utilizing Enzyme Putative |
| pstB2 | 804 | 4 | 9.35 | 0.48 | -1.05 | 6-Phosphofructokinase6-Phosphofructokinase |
| tRNA-Ser-TGA | 91 | 0 | 1.06 | 0.49 | -1.04 | Putative Arginine Decarboxylase Putative |
| fabK | 975 | 5 | 11.34 | 0.49 | -1.04 | Putative GTP-Binding Protein Putative |
| INV10416770 | 621 | 3 | 7.22 | 0.49 | -1.04 | Putative Oligopeptide Transporter ATP-Binding Protein Putative |
| INV10411180 | 267 | 1 | 3.1 | 0.49 | -1.04 | Putative Membrane Protein Putative |
| veg | 267 | 1 | 3.1 | 0.49 | -1.04 | Tn916, Hypothetical Proteintn916, |
| parE | 1854 | 10 | 21.55 | 0.49 | -1.04 | UDP-N-Acetylmuramate--Alanine Ligaseudp-N-Acetylmuramate--Alanine |
| INV10416170 | 972 | 5 | 11.3 | 0.49 | -1.04 | Hypothetical Proteinhypothetical |
| glr | 795 | 4 | 9.24 | 0.49 | -1.03 | 1,4-Alpha-Glucan Branching Enzyme1,4-Alpha-Glucan |
| pflC | 795 | 4 | 9.24 | 0.49 | -1.03 | N-Acetylmannosamine Kinase (EC 2.7.1.60)N-Acetylmannosamine |
| INV10401690 | 618 | 3 | 7.18 | 0.49 | -1.03 | Asparagine Synthase Family Proteinasparagine |
| INV10401360 | 441 | 2 | 5.13 | 0.49 | -1.03 | Leader Peptidaseleader |
| INV10418760 | 441 | 2 | 5.13 | 0.49 | -1.03 | Phosphate Transport System Proteinphosphate |
| INV10402330 | 264 | 1 | 3.07 | 0.49 | -1.03 | Putative DNA-Binding Protein Putative |
| INV10410500 | 264 | 1 | 3.07 | 0.49 | -1.03 | Conserved Hypothetical Proteinconserved |
| MutatormutTprotein(7_8-dihydro-8-oxoguanine-triphosphatase)(EC3 | 264 | 1 | 3.07 | 0.49 | -1.03 | Lysyl-Trna Synthetaselysyl-Trna |
| Plasmidaddictionsystempoisonprotein | 264 | 1 | 3.07 | 0.49 | -1.03 | Putative Endonuclease Iii Putative |
| INV10414120 | 789 | 4 | 9.17 | 0.49 | -1.02 | DEAD Box Helicase Family Proteindead |
| nusA | 1137 | 6 | 13.22 | 0.49 | -1.02 | S-Adenosyl-Methyltransferase Mraws-Adenosyl-Methyltransferase |
| 747532_754257 | 6725 | 38 | 78.19 | 0.49 | -1.02 |  |
| tRNA-Ser-GCT | 88 | 0 | 1.02 | 0.49 | -1.01 | Superoxide Dismutase [Mn]Superoxide |
| INV10415420 | 609 | 3 | 7.08 | 0.5 | -1.01 | Putative Protein Phosphatase Putative |
| metG | 1998 | 11 | 23.23 | 0.5 | -1.01 | Tagatose-6-Phosphate Kinase 2tagatose-6-Phosphate |
| pheS | 1128 | 6 | 13.11 | 0.5 | -1.01 | Glucosamine-6-Phosphate Isomeraseglucosamine-6-Phosphate |
| mvaD | 954 | 5 | 11.09 | 0.5 | -1.01 | Putative Maltose/Maltodextrin-Binding Protein Precursor Putative |
| purB | 1299 | 7 | 15.1 | 0.5 | -1.01 | Phage Integrase: Site-Specific Recombinasephage |
| INV10404300 | 2335 | 13 | 27.15 | 0.5 | -1.01 | Conserved Hypothetical Proteinconserved |
| pbp1A | 2160 | 12 | 25.11 | 0.5 | -1.01 | UDP-N-Acetylmuramoylalanine--D-Glutamate Ligaseudp-N-Acetylmuramoylalanine--D-Glutamate |
| INV10408580 | 432 | 2 | 5.02 | 0.5 | -1 | Putative Permease Putative |
| tRNA-Leu-TAA | 86 | 0 | 1 | 0.5 | -1 | Sialic Acid-Induced Transmembrane Protein Yjht(Nanm), Possible Mutarotasesialic |
| INV10417130 | 774 | 4 | 9 | 0.5 | -1 | Putative Uncharacterized Protein Putative |
| MutTnudixfamilyprotein | 429 | 2 | 4.99 | 0.5 | -1 | Putative Maltose/Maltodextrin ABC Transport System Permease Protein Putative |
| putativeparvulintypepeptidyl-prolylisomerase_similaritywithPrsAfoldase | 942 | 5 | 10.95 | 0.5 | -0.99 | Prephenate Dehydrataseprephenate |
| rex | 426 | 2 | 4.95 | 0.5 | -0.99 | Positive Transcriptional Regulator, Mutr Familypositive |
| INV10405460 | 255 | 1 | 2.96 | 0.5 | -0.99 | ABC Transporter, Permease Protein ABC |
| INV10406240 | 765 | 4 | 8.89 | 0.51 | -0.98 | Putative Membrane Protein Putative |
| tRNA-Leu-CAA | 84 | 0 | 0.98 | 0.51 | -0.99 | Seryl-Trna Synthetaseseryl-Trna |
| fabZ | 423 | 2 | 4.92 | 0.51 | -0.98 | Probable Gtpase Engc (Pseudogene)Probable |
| INV10400100 | 1269 | 7 | 14.75 | 0.51 | -0.98 | Hypothetical Proteinhypothetical |
| INV10406710 | 930 | 5 | 10.81 | 0.51 | -0.98 | Conserved Hypothetical Proteinconserved |
| rpsT | 252 | 1 | 2.93 | 0.51 | -0.97 | DNA Repair Protein Recodna |
| INV10413080 | 759 | 4 | 8.82 | 0.51 | -0.97 | Putative Pyridoxine Biosynthesis Protein Putative |
| INV10416270 | 927 | 5 | 10.78 | 0.51 | -0.97 | Hypotheical Proteinhypotheical |
| Argininosuccinatesynthase(EC6 | 420 | 2 | 4.88 | 0.51 | -0.97 | Argininosuccinate Synthase (EC 6.3.4.5)Argininosuccinate |
| INV10407100 | 924 | 5 | 10.74 | 0.51 | -0.97 | Conserved Hypothetical Proteinconserved |
| tRNA-Tyr-GTA | 82 | 0 | 0.95 | 0.51 | -0.96 | Spn1 Transposasespn1 |
| INV10411850 | 753 | 4 | 8.75 | 0.51 | -0.96 | SMF Family Proteinsmf |
| INV10413950 | 416 | 2 | 4.84 | 0.51 | -0.96 | P-Loop Atpase Protein Family Proteinp-Loop |
| INV10402440 | 1420 | 8 | 16.51 | 0.51 | -0.96 | Cora-Like Mg2+ Transporter Proteincora-Like |
| INV10412490 | 582 | 3 | 6.77 | 0.52 | -0.96 | Putative Acyl-ACP Thioesterase Putative |
| pepQ | 1083 | 6 | 12.59 | 0.52 | -0.96 | Putative A/G-Specific Adenine Glycosylase Putative |
| FIG01117917-hypotheticalprotein | 1245 | 7 | 14.47 | 0.52 | -0.95 | FIG01115961: Hypothetical Proteinfig01115961: |
| INV10405210 | 579 | 3 | 6.73 | 0.52 | -0.95 | Conserved Hypothetical Proteinconserved |
| INV10417400 | 1410 | 8 | 16.39 | 0.52 | -0.95 | Acetyltransferase (GNAT) Family Proteinacetyltransferase |
| 1676971_1677217 | 246 | 1 | 2.86 | 0.52 | -0.95 |  |
| INV10415440 | 246 | 1 | 2.86 | 0.52 | -0.95 | Putative Phosphohydrolase Putative |
| INV10400520 | 906 | 5 | 10.53 | 0.52 | -0.94 | Translation Initiation Factor IF-2translation |
| INV10402760 | 243 | 1 | 2.83 | 0.52 | -0.94 | Conserved Hypothetical Proteinconserved |
| INV10400760 | 1065 | 6 | 12.38 | 0.52 | -0.93 | Putative Carbonic Anhydrase Putative |
| csdB | 1227 | 7 | 14.27 | 0.52 | -0.93 | Conserved Domain Proteinconserved |
| INV10401570 | 570 | 3 | 6.63 | 0.52 | -0.93 | Putative Membrane Protein Putative |
| INV10409480 | 570 | 3 | 6.63 | 0.52 | -0.93 | Putative Regulatory Protein Putative |
| scpB | 570 | 3 | 6.63 | 0.52 | -0.93 | Riboflavin Biosynthesis Protein [Includes: Diaminohydroxyphosphoribosylaminopyrimidine Deaminase And 5-Amino-6-(5- Phosphoribosylamino)Uracil Reductase]Riboflavin |
| wzh | 732 | 4 | 8.51 | 0.53 | -0.93 | Transcriptional Regulatortranscriptional |
| INV10412290 | 241 | 1 | 2.8 | 0.53 | -0.93 | Cell Envelope-Related Transcriptional Attenuator Domain Proteincell |
| Mobileelementprotein | 241 | 1 | 2.8 | 0.53 | -0.93 | 6-Phospho-Beta-Galactosidase 26-Phospho-Beta-Galactosidase |
| INV10406450 | 240 | 1 | 2.79 | 0.53 | -0.92 | ABC Transporter Amino Acid-Binding Protein ABC |
| INV10406510 | 240 | 1 | 2.79 | 0.53 | -0.92 | Putative L-Lactate Oxidase-Related Protein Putative |
| rpsR | 240 | 1 | 2.79 | 0.53 | -0.92 | ATP-Dependent DNA Helicaseatp-Dependent |
| INV10418400 | 2031 | 12 | 23.61 | 0.53 | -0.92 | Putative Competence Protein Putative |
| INV10417120 | 561 | 3 | 6.52 | 0.53 | -0.91 | Conserved Hypothetical Proteinconserved |
| LipidAexportATP-bindingpermeaseproteinMsbA | 561 | 3 | 6.52 | 0.53 | -0.91 | IS3-Spn1 Transposaseis3-Spn1 |
| INV10406610 | 237 | 1 | 2.76 | 0.53 | -0.91 | Putative Regulator Putative |
| tRNA-Ile-GAT | 75 | 0 | 0.87 | 0.53 | -0.9 | Preprotein Translocase Secy Subunitpreprotein |
| tRNA-Met-CAT | 75 | 0 | 0.87 | 0.53 | -0.9 | Putative Chromosome Partition Protein Putative |
| atpG | 879 | 5 | 10.22 | 0.53 | -0.9 | ATP Synthase C Chainatp |
| truB | 879 | 5 | 10.22 | 0.53 | -0.9 | Tellurite Resistance Protein Tehbtellurite |
| INV10415990 | 1521 | 9 | 17.68 | 0.54 | -0.9 | Csbd-Like Proteincsbd-Like |
| INV10406360 | 1038 | 6 | 12.07 | 0.54 | -0.9 | Putative Membrane Protein Putative |
| fps | 876 | 5 | 10.18 | 0.54 | -0.9 | FIG01119302: Hypothetical Proteinfig01119302: |
| tRNA-Arg-TCT | 74 | 0 | 0.86 | 0.54 | -0.9 | Segregation And Condensation Protein Bsegregation |
| tRNA-Phe-GAA | 74 | 0 | 0.86 | 0.54 | -0.9 | Putative DNA Repair Protein Putative |
| tRNA-Pro-TGG | 74 | 0 | 0.86 | 0.54 | -0.9 | SNF2 Family Proteinsnf2 |
| INV10400980 | 234 | 1 | 2.72 | 0.54 | -0.9 | Sugar Phosphotransferase System (PTS), Fructose Family, IIA Componentsugar |
| gidB | 714 | 4 | 8.3 | 0.54 | -0.9 | Fuscose Operon Fucu Proteinfuscose |
| INV10419350 | 714 | 4 | 8.3 | 0.54 | -0.9 | Putative Fucose Phosphotransferase System Repressor Putative |
| tRNA-His-GTG | 73 | 0 | 0.85 | 0.54 | -0.89 | Putative Preprotein Seca Subunit Putative |
| tRNA-Thr-GGT | 73 | 0 | 0.85 | 0.54 | -0.89 | Putative Spermidine Synthase Putative |
| INV10413180 | 550 | 3 | 6.39 | 0.54 | -0.89 | Putative Uncharacterized Protein Putative |
| cysS | 1344 | 8 | 15.63 | 0.54 | -0.89 | Response Regulator Proteinresponse |
| INV10413260 | 1185 | 7 | 13.78 | 0.54 | -0.89 | Putative Transposase (Pseudogene) Putative |
| INV10410930 | 708 | 4 | 8.23 | 0.54 | -0.88 | Putative Ribonucleoside-Diphosphate Reductase Alpha Chain Putative |
| nagB | 708 | 4 | 8.23 | 0.54 | -0.88 | 5-Methyltetrahydropteroyltriglutamate--Homocyst Eine Methyltransferase5-Methyltetrahydropteroyltriglutamate--Homocyst |
| purM | 1023 | 6 | 11.89 | 0.54 | -0.88 | Phage Tail Length Tape-Measure Proteinphage |
| INV10402050 | 72 | 0 | 0.84 | 0.54 | -0.88 | Putative Peptidase Putative |
| tRNA-Arg-CCG | 72 | 0 | 0.84 | 0.54 | -0.88 | Metal-Dependent Transcriptional Regulatormetal-Dependent |
| tRNA-Arg-CCT | 72 | 0 | 0.84 | 0.54 | -0.88 | Segregation And Condensation Protein Asegregation |
| tRNA-Gln-TTG | 72 | 0 | 0.84 | 0.54 | -0.88 | Fructokinasefructokinase |
| tRNA-Gly-GCC | 72 | 0 | 0.84 | 0.54 | -0.88 | Putative L-Serine Dehydratase, Alpha Chain Putative |
| tRNA-Gly-TCC | 72 | 0 | 0.84 | 0.54 | -0.88 | Putative L-Serine Dehydratase, Beta Chain Putative |
| INV10409380 | 861 | 5 | 10.01 | 0.54 | -0.88 | Acetyltransferase (GNAT) Family Proteinacetyltransferase |
| INV10414060 | 702 | 4 | 8.16 | 0.55 | -0.87 | Putative DNA Replication Protein Dnad Putative |
| zwf | 1488 | 9 | 17.3 | 0.55 | -0.87 | Transposasetransposase |
| folD | 858 | 5 | 9.98 | 0.55 | -0.87 | FIG01117917: Hypothetical Proteinfig01117917: |
| tRNA-Cys-GCA | 71 | 0 | 0.83 | 0.55 | -0.87 | Putative Sucrose-6-Phosphate Hydrolase Putative |
| tRNA-Trp-CCA | 71 | 0 | 0.83 | 0.55 | -0.87 | Putative Signal Peptidase I Putative |
| INV10408130 | 228 | 1 | 2.65 | 0.55 | -0.87 | CAAX Amino Protease Family Proteincaax |
| lytB | 2109 | 13 | 24.52 | 0.55 | -0.87 | IS1167, Transposaseis1167, |
| CatabolitecontrolproteinA | 1011 | 6 | 11.75 | 0.55 | -0.87 | Carbamoyl-Phosphate Synthase Small Chaincarbamoyl-Phosphate |
| INV10412040 | 384 | 2 | 4.46 | 0.55 | -0.86 | Putative DNA-Binding Protein Putative |
| clpL | 2106 | 13 | 24.48 | 0.55 | -0.86 | Putative Stress Response-Related Clp Atpase Putative |
| INV10414150 | 540 | 3 | 6.28 | 0.55 | -0.86 | Mur Ligase Family Proteinmur |
| INV10414620 | 696 | 4 | 8.09 | 0.55 | -0.86 | Putative Membrane Protein (Pseudogene) Putative |
| INV10403350 | 538 | 3 | 6.25 | 0.55 | -0.86 | Putative Surface-Anchored Pullulanase Putative |
| INV10409230 | 1005 | 6 | 11.68 | 0.55 | -0.86 | Putative Uncharacterized Protein (Pseudogene) Putative |
| pheA | 849 | 5 | 9.87 | 0.55 | -0.86 | NH(3)-Dependent NAD(+) Synthetasenh(3)-Dependent |
| INV10412410 | 225 | 1 | 2.62 | 0.55 | -0.86 | Putative Phosphate ABC Transporter Permease Protein Putative |
| INV10406150 | 846 | 5 | 9.84 | 0.55 | -0.85 | Hypothetical Proteinhypothetical |
| csrR | 690 | 4 | 8.02 | 0.55 | -0.85 | Conserved Domain Proteinconserved |
| rplA | 690 | 4 | 8.02 | 0.55 | -0.85 | Prophage Pi2 Protein 37prophage |
| INV10415190 | 222 | 1 | 2.58 | 0.56 | -0.84 | Putative Permease Component Of ABC Transporter Putative |
| 165155_167375 | 2220 | 14 | 25.81 | 0.56 | -0.84 |  |
| INV10401050 | 2220 | 14 | 25.81 | 0.56 | -0.84 | Phosphorylase Family Proteinphosphorylase |
| INV10418640 | 2220 | 14 | 25.81 | 0.56 | -0.84 | Putative Peptidase Putative |
| manL | 990 | 6 | 11.51 | 0.56 | -0.84 | IS630-Spn1, Transposase Orf1IS630-Spn1, |
| INV10414380 | 834 | 5 | 9.7 | 0.56 | -0.83 | Putative Cation-Transporting Atpase Putative |
| INV10401290 | 1599 | 10 | 18.59 | 0.56 | -0.83 | Putative Glycosyl Transferase Putative |
| INV10404100 | 831 | 5 | 9.66 | 0.56 | -0.83 | Putative DNA Alkylation Repair Enzyme (Pseudogene) Putative |
| aroD | 678 | 4 | 7.88 | 0.56 | -0.83 | 3-Dehydroquinate Synthase3-Dehydroquinate |
| Transcriptionalregulator_ArsRfamily | 678 | 4 | 7.88 | 0.56 | -0.83 | 30S Ribosomal Protein S930S |
| INV10403980 | 219 | 1 | 2.55 | 0.56 | -0.83 | Group II Intron Maturasegroup |
| INV10411880 | 828 | 5 | 9.63 | 0.56 | -0.83 | Putative Kinase Putative |
| murA1 | 1284 | 8 | 14.93 | 0.57 | -0.82 | Lipid A Export ATP-Binding/Permease Protein Msbalipid |
| nadE | 825 | 5 | 9.59 | 0.57 | -0.82 | Metal-Dependent Hydrolase Ybey, Involved In Rrna And/Or Ribosome Maturation And Assemblymetal-Dependent |
| LSUribosomalproteinL7L12(P1P2) | 369 | 2 | 4.29 | 0.57 | -0.82 | Iron Compound ABC Uptake Transporter Substrate-Binding Protein Piaairon |
| cmk | 672 | 4 | 7.81 | 0.57 | -0.82 | Putative ATP-Dependent Clp Protease Proteolytic Subunit Putative |
| INV10409490 | 672 | 4 | 7.81 | 0.57 | -0.82 | Putative Iron-Siderophore Binding Lipoprotein Putative |
| fabH | 975 | 6 | 11.34 | 0.57 | -0.82 | GTP-Binding Protein Engagtp-Binding |
| INV10408100 | 216 | 1 | 2.51 | 0.57 | -0.81 | Putative Type I Restriction Modification System Restriction Protein Putative |
| INV10405290 | 215 | 1 | 2.5 | 0.57 | -0.81 | Putative Uncharacterized Protein Putative |
| prfA | 1569 | 10 | 18.24 | 0.57 | -0.81 | Putative Xanthine Permease Putative |
| idnO | 816 | 5 | 9.49 | 0.57 | -0.81 | Ferrochelataseferrochelatase |
| INV10413720 | 213 | 1 | 2.48 | 0.58 | -0.8 | Cyclophilin Type Peptidyl-Prolyl Cis-Trans Isomerase Proteincyclophilin |
| rpoD | 1110 | 7 | 12.9 | 0.58 | -0.8 | Putative Transposon Integrase; Tn916 ORF3-Like Putative |
| Prolyl-tRNAsynthetase(EC6 | 1854 | 12 | 21.55 | 0.58 | -0.79 | Putative Lysyl-Aminopeptidase Putative |
| INV10406260 | 657 | 4 | 7.64 | 0.58 | -0.79 | Bipa Family Gtpasebipa |
| ilvA | 1251 | 8 | 14.54 | 0.58 | -0.79 | Histidyl-Trna Synthetase (EC 6.1.1.21)Histidyl-Trna |
| INV10407790 | 1987 | 13 | 23.1 | 0.58 | -0.78 | Putative Branched-Chain-Amino-Acid Aminotransferase Putative |
| INV10405180 | 801 | 5 | 9.31 | 0.58 | -0.78 | Conserved Hypothetical Proteinconserved |
| Phagelysin_glycosylhydrolase_family25 | 801 | 5 | 9.31 | 0.58 | -0.78 | N-Ethylammeline Chlorohydrolasen-Ethylammeline |
| aliA | 1983 | 13 | 23.05 | 0.58 | -0.78 | Putative Extracellular Oligopeptide-Binding Protein Putative |
| glnR | 357 | 2 | 4.15 | 0.58 | -0.78 | Glucose Inhibited Division Protein Aglucose |
| INV10403870 | 357 | 2 | 4.15 | 0.58 | -0.78 | Membrane Proteinmembrane |
| INV10419060 | 1969 | 13 | 22.89 | 0.59 | -0.77 | Putative PTS Multi-Domain Regulator Putative |
| INV10414870 | 354 | 2 | 4.12 | 0.59 | -0.77 | Mutt/NUDIX Hydrolase Family Proteinmutt/NUDIX |
| blpO | 207 | 1 | 2.41 | 0.59 | -0.77 | Peptide Pheromone Blpc (Bacteriocin-Like Peptide)Peptide |
| INV10419130 | 60 | 0 | 0.7 | 0.59 | -0.77 | Putative Glycosyl Hydrolase Putative |
| pepT | 1228 | 8 | 14.28 | 0.59 | -0.76 | Mevalonate Diphosphate Decarboxylasemevalonate |
| codY | 789 | 5 | 9.17 | 0.59 | -0.76 | Putative Phosphopantothenoylcysteine Decarboxylase Putative |
| INV10407040 | 642 | 4 | 7.46 | 0.59 | -0.76 | Conserved Hypothetical Proteinconserved |
| INV10412460 | 495 | 3 | 5.75 | 0.59 | -0.75 | Conserved Hypothetical Proteinconserved |
| INV10412270 | 1366 | 9 | 15.88 | 0.59 | -0.76 | Putative Glycosyl Transferase Putative |
| INV10401060 | 930 | 6 | 10.81 | 0.59 | -0.75 | Trkh-Family Cation Transport Proteintrkh-Family |
| 24224_59696 | 35472 | 246 | 412.4 | 0.6 | -0.74 |  |
| INV10413320 | 1353 | 9 | 15.73 | 0.6 | -0.74 | Putative Uncharacterized Protein Putative |
| fabD | 921 | 6 | 10.71 | 0.6 | -0.74 | Dps-Like Peroxide Resistance Protein Dprdps-Like |
| pyrE | 633 | 4 | 7.36 | 0.6 | -0.74 | Phosphoglycerate Mutase Family 5Phosphoglycerate |
| INV10410630 | 489 | 3 | 5.69 | 0.6 | -0.74 | Putative Membrane Protein Putative |
| rplV | 345 | 2 | 4.01 | 0.6 | -0.74 | Putative Purine Nucleoside Phosphorylase Putative |
| INV10407130 | 1203 | 8 | 13.99 | 0.6 | -0.74 | Putative Exported Protein Putative |
| INV10413580 | 1344 | 9 | 15.63 | 0.6 | -0.73 | Putative Cystathionine Beta-Lyase Putative |
| FIG01114767-hypotheticalprotein | 198 | 1 | 2.3 | 0.61 | -0.72 | FIG01114010: Hypothetical Proteinfig01114010: |
| gp9 | 198 | 1 | 2.3 | 0.61 | -0.72 | Glycerol Kinaseglycerol |
| INV10416650 | 198 | 1 | 2.3 | 0.61 | -0.72 | Conserved Hypothetical Proteinconserved |
| INV10417570 | 624 | 4 | 7.25 | 0.61 | -0.72 | Putative Acylphosphatase Putative |
| INV10416010 | 766 | 5 | 8.91 | 0.61 | -0.72 | Putative Membrane Protein Putative |
| INV10405390 | 1476 | 10 | 17.16 | 0.61 | -0.72 | Putative Acetyltransferase, GNAT Family Protein Putative |
| INV10412210 | 1191 | 8 | 13.85 | 0.61 | -0.72 | Putative ABC Transporter ATP-Binding Protein Putative |
| INV10414840 | 1614 | 11 | 18.76 | 0.61 | -0.72 | Conserved Hypothetical Proteinconserved |
| INV10414330 | 1329 | 9 | 15.45 | 0.61 | -0.72 | Membrane Glycosyl Transferasemembrane |
| pepO | 1893 | 13 | 22.01 | 0.61 | -0.72 | Mutator Mutx Protein (7,8-Dihydro-8-Oxoguanine-Triphosphatase)Mutator |
| zmpB | 5568 | 39 | 64.73 | 0.61 | -0.72 | Transketolase (Ec 2.2.1.1)Transketolase |
| GTP-bindingproteinEra | 900 | 6 | 10.46 | 0.61 | -0.71 | Glycerophosphoryl Diester Phosphodiesterase (EC 3.1.4.46)Glycerophosphoryl |
| INV10409070 | 759 | 5 | 8.82 | 0.61 | -0.71 | Phoh-Like Proteinphoh-Like |
| INV10414660 | 618 | 4 | 7.18 | 0.61 | -0.71 | ABC Transporter ATP-Binding Protein ABC |
| INV10414750 | 336 | 2 | 3.91 | 0.61 | -0.71 | Integral Membrane Proteinintegral |
| aga | 2163 | 15 | 25.15 | 0.61 | -0.71 | Alpha-Galactosidasealpha-Galactosidase |
| FIG01119302-hypotheticalprotein | 195 | 1 | 2.27 | 0.61 | -0.71 | FIG01116389: Hypothetical Proteinfig01116389: |
| INV10410180 | 2160 | 15 | 25.11 | 0.61 | -0.71 | Putative Membrane Protein Putative |
| INV10406140 | 1176 | 8 | 13.67 | 0.61 | -0.7 |  |
| INV10401280 | 615 | 4 | 7.15 | 0.61 | -0.7 | Putative Major Facilitator Superfamily Protein (Pseudogene) Putative |
| INV10413220 | 1596 | 11 | 18.56 | 0.61 | -0.7 | Aldo/Keto Reductase Family Proteinaldo/Keto |
| hrcA | 1035 | 7 | 12.03 | 0.61 | -0.7 | GMP Synthase [Glutamine-Hydrolyzing]GMP |
| INV10414650 | 894 | 6 | 10.39 | 0.61 | -0.7 | ABC Transporter Permease Protein (Permease)ABC |
| rpsD | 612 | 4 | 7.12 | 0.62 | -0.7 | Dihydroorotate Dehydrogenase, Catalytic Subunitdihydroorotate |
| INV10401320 | 471 | 3 | 5.48 | 0.62 | -0.7 | Lysm Domain Proteinlysm |
| INV10414390 | 885 | 6 | 10.29 | 0.62 | -0.69 | Putative Acyltransferase Putative |
| INV10409280 | 330 | 2 | 3.84 | 0.62 | -0.69 | Putative Amino Acid Permease Putative |
| INV10406210 | 1161 | 8 | 13.5 | 0.62 | -0.69 | Short Chain Dehydrogenaseshort |
| INV10412930 | 466 | 3 | 5.42 | 0.62 | -0.68 | Haloacid Dehalogenase-Like Hydrolasehaloacid |
| comYC | 327 | 2 | 3.8 | 0.62 | -0.68 | Competence-Specific Sigma Factor Comxcompetence-Specific |
| INV10415570 | 1152 | 8 | 13.39 | 0.63 | -0.68 | Putative Hydrolase Putative |
| INV10409060 | 189 | 1 | 2.2 | 0.63 | -0.68 | Conserved Hypothetical Proteinconserved |
| guaA | 1563 | 11 | 18.17 | 0.63 | -0.68 | Glycyl-Trna Synthetase Alpha Chainglycyl-Trna |
| INV10404960 | 600 | 4 | 6.98 | 0.63 | -0.67 | Putative Endo-Beta-N-Acetylglucosaminidase Putative |
| Two-componentresponseregulator | 600 | 4 | 6.98 | 0.63 | -0.67 | Phosphomethylpyrimidine Kinasephosphomethylpyrimidine |
| INV10401620 | 462 | 3 | 5.37 | 0.63 | -0.67 | Putative Transposase (Pseudogene) Putative |
| INV10418900 | 873 | 6 | 10.15 | 0.63 | -0.67 | Putative Membrane Protein Putative |
| INV10404940 | 324 | 2 | 3.77 | 0.63 | -0.67 | Putative Uncharacterized Protein Putative |
| INV10418490 | 870 | 6 | 10.11 | 0.63 | -0.67 | Putative N-Acetylglucosamine-6-Phosphate Deacetylase Putative |
| INV10401010 | 187 | 1 | 2.17 | 0.63 | -0.66 | Glyoxalase/Bleomycin Resistance Protein/Dioxygenase Superfamily Proteinglyoxalase/Bleomycin |
| deoA | 1278 | 9 | 14.86 | 0.63 | -0.67 | Putative Dihydrodipicolinate Reductase Putative |
| msmF | 867 | 6 | 10.08 | 0.63 | -0.66 | L-Lactate Dehydrogenasel-Lactate |
| INV10404850 | 1002 | 7 | 11.65 | 0.63 | -0.66 | Putative Transport Protein Putative |
| INV10416000 | 729 | 5 | 8.48 | 0.63 | -0.66 | Putative Transposase (Pseudogene) Putative |
| INV10416780 | 864 | 6 | 10.04 | 0.63 | -0.66 | Putative Oligopeptide Transporter Permease Protein Putative |
| INV10415480 | 1135 | 8 | 13.2 | 0.63 | -0.66 | Putative Methyltransferase Putative |
| DNApolymeraseIIIalphasubunit(EC2 | 591 | 4 | 6.87 | 0.64 | -0.65 | Chromosomal Replication Initiator Proteinchromosomal |
| INV10401330 | 1266 | 9 | 14.72 | 0.64 | -0.65 | Hypothetical Protein (Pseudogene)Hypothetical |
| INV10415530 | 1263 | 9 | 14.68 | 0.64 | -0.65 | Conserved Hypothetical Proteinconserved |
| asnA | 993 | 7 | 11.54 | 0.64 | -0.65 | Putative Shikimate Kinase Putative |
| INV10413340 | 858 | 6 | 9.98 | 0.64 | -0.65 | Conserved Hypothetical Proteinconserved |
| engB | 588 | 4 | 6.84 | 0.64 | -0.65 | Chaperone Protein Dnak (Heat Shock Protein 70)Chaperone |
| msmE | 1260 | 9 | 14.65 | 0.64 | -0.65 | L-Lactate Oxidasel-Lactate |
| INV10404680 | 183 | 1 | 2.13 | 0.64 | -0.65 | Putative Membrane Protein Putative |
| Methylaseinvolvedinubiquinonemenaquinonebiosynthesis | 183 | 1 | 2.13 | 0.64 | -0.65 | Tagatose 1,6-Diphosphate Aldolase 2tagatose |
| INV10410010 | 990 | 7 | 11.51 | 0.64 | -0.65 | ABC Transporter Permease Protein ABC |
| INV10414250 | 852 | 6 | 9.91 | 0.64 | -0.64 | IS3-Spn1 Orf A (Pseudogene)IS3-Spn1 |
| pfl | 2325 | 17 | 27.03 | 0.64 | -0.64 | N-Acetylmannosamine-6-Phosphate 2-Epimerase (EC 5.1.3.9)N-Acetylmannosamine-6-Phosphate |
| INV10415200 | 315 | 2 | 3.66 | 0.64 | -0.64 | Putative ABC Transport Protein, Solute-Binding Component Putative |
| rpoZ | 315 | 2 | 3.66 | 0.64 | -0.64 | Aspartate Carbamoyltransferaseaspartate |
| xpt | 582 | 4 | 6.77 | 0.64 | -0.64 | Transcriptional Regulator, Merr Familytranscriptional |
| INV10410880 | 849 | 6 | 9.87 | 0.64 | -0.63 | Putative Uncharacterized Protein Putative |
| dltB | 1246 | 9 | 14.49 | 0.65 | -0.63 | Thymidine Phosphorylasethymidine |
| INV10404200 | 846 | 6 | 9.84 | 0.65 | -0.63 | Conserved Hypothetical Proteinconserved |
| INV10403270 | 180 | 1 | 2.09 | 0.65 | -0.63 | Sugar Phosphotransferase System (PTS), IIC Componentsugar |
| glpK | 1509 | 11 | 17.54 | 0.65 | -0.63 | Glycerol Dehydrogenaseglycerol |
| INV10404970 | 578 | 4 | 6.72 | 0.65 | -0.63 | Putative Membrane Protein Putative |
| INV10414910 | 312 | 2 | 3.63 | 0.65 | -0.63 | Putative Uncharacterized Protein Putative |
| purC | 708 | 5 | 8.23 | 0.65 | -0.62 | Phage Lysin, Glycosyl Hydrolase, Family 25Phage |
| tyrA | 1104 | 8 | 12.84 | 0.65 | -0.62 | Thiamin-Phosphate Pyrophosphorylasethiamin-Phosphate |
| iscU | 441 | 3 | 5.13 | 0.65 | -0.62 | Putative Two-Component System, Response Regulator Putative |
| INV10402740 | 966 | 7 | 11.23 | 0.65 | -0.61 | 30S Ribosomal Protein S1430S |
| INV10406550 | 702 | 5 | 8.16 | 0.65 | -0.61 | Putative ABC Transporter, ATP-Binding Protein Putative |
| INV10416050 | 702 | 5 | 8.16 | 0.65 | -0.61 | Sugar Phosphotransferase System (PTS), Lactose/Cellobiose-Specific Family, IIB Componentsugar |
| 553904_554080 | 176 | 1 | 2.05 | 0.66 | -0.61 |  |
| INV10404980 | 176 | 1 | 2.05 | 0.66 | -0.61 | Type I Restriction-Modification System M Proteintype |
| INV10405440 | 958 | 7 | 11.14 | 0.66 | -0.6 | Putative Uncharacterized Protein (Pseudogene) Putative |
| aliB | 174 | 1 | 2.02 | 0.66 | -0.59 | Putative Oligopeptide-Binding Protein Alib (Pseudogene) Putative |
| 1507612_1517795 | 10183 | 78 | 118.39 | 0.66 | -0.6 |  |
| INV10417650 | 303 | 2 | 3.52 | 0.66 | -0.59 | Hypothetical Protein (Pseudogene)Hypothetical |
| INV10414400 | 1338 | 10 | 15.56 | 0.66 | -0.59 | Conserved Hypothetical Protein (Pseudogene)Conserved |
| INV10407090 | 690 | 5 | 8.02 | 0.67 | -0.59 | Conserved Hypothetical Proteinconserved |
| INV10409420 | 690 | 5 | 8.02 | 0.67 | -0.59 | Putative Membrane Protein Putative |
| INV10417860 | 690 | 5 | 8.02 | 0.67 | -0.59 | Putative Nucleotide-Binding Protein Putative |
| ppnK | 819 | 6 | 9.52 | 0.67 | -0.59 | Penicillin-Binding Protein 2apenicillin-Binding |
| INV10415410 | 172 | 1 | 2 | 0.67 | -0.58 | Putative Membrane Protein Putative |
| INV10406390 | 688 | 5 | 8 | 0.67 | -0.58 | ABC Transporter ATP-Binding Protein ABC |
| INV10406160 | 558 | 4 | 6.49 | 0.67 | -0.58 | Putative Exported Protein Putative |
| INV10409040 | 1588 | 12 | 18.46 | 0.67 | -0.58 | Protein Gid Homologprotein |
| 1955335_1955635 | 300 | 2 | 3.49 | 0.67 | -0.58 |  |
| INV10406780 | 300 | 2 | 3.49 | 0.67 | -0.58 | Branched-Chain Amino Acid ABC Transporter, Amino Acid-Binding Proteinbranched-Chain |
| INV10413070 | 300 | 2 | 3.49 | 0.67 | -0.58 | SNO Glutamine Amidotransferase Family Proteinsno |
| INV10418010 | 300 | 2 | 3.49 | 0.67 | -0.58 | L-Ribulose 5-Phosphate 4-Epimerasel-Ribulose |
| infA | 171 | 1 | 1.99 | 0.67 | -0.58 | Hypoxanthine-Guanine Phosphoribosyltransferasehypoxanthine-Guanine |
| INV10409300 | 1326 | 10 | 15.42 | 0.67 | -0.58 | Pneumococcal Histidine Triad Protein E (Pseudogene)Pneumococcal |
| pepA | 1065 | 8 | 12.38 | 0.67 | -0.57 | Putative DNA Mismatch Repair Protein Putative |
| wzg | 1446 | 11 | 16.81 | 0.67 | -0.57 | Transcriptional Antiterminator Of Lichenan Operon, Bglg Familytranscriptional |
| INV10418360 | 933 | 7 | 10.85 | 0.68 | -0.57 | Putative Membrane Protein Putative |
| FIG011945-O-methyltransferasefamilyprotein | 678 | 5 | 7.88 | 0.68 | -0.57 | FIG01116415: Hypothetical Proteinfig01116415: |
| INV10406420 | 804 | 6 | 9.35 | 0.68 | -0.56 | Putative Membrane Protein Putative |
| INV10417180 | 294 | 2 | 3.42 | 0.68 | -0.56 | Putative Uncharacterized Protein Putative |
| INV10401070 | 547 | 4 | 6.36 | 0.68 | -0.56 | Trka Family Cation Transport Proteintrka |
| INV10409330 | 1305 | 10 | 15.17 | 0.68 | -0.56 | Putative Membrane Protein Putative |
| parC | 2436 | 19 | 28.32 | 0.68 | -0.55 | UDP-N-Acetylenolpyruvoylglucosamine Reductaseudp-N-Acetylenolpyruvoylglucosamine |
| 906385_910327 | 3942 | 31 | 45.83 | 0.68 | -0.55 |  |
| nanH | 918 | 7 | 10.67 | 0.69 | -0.54 | Methylase Involved In Ubiquinone/Menaquinone Biosynthesismethylase |
| ndk | 414 | 3 | 4.81 | 0.69 | -0.54 | S-Adenosylmethionine Synthetases-Adenosylmethionine |
| fruK | 912 | 7 | 10.6 | 0.69 | -0.54 | FIG011945: O-Methyltransferase Family Proteinfig011945: |
| manN | 912 | 7 | 10.6 | 0.69 | -0.54 | IS861, Transposase (Orf1), IS3 Family, Truncatedis861, |
| INV10414130 | 786 | 6 | 9.14 | 0.69 | -0.53 | Major Facilitator Superfamily Protein (Pseudogene)Major |
| INV10418840 | 661 | 5 | 7.68 | 0.69 | -0.53 | 5-Formyltetrahydrofolate Cyclo-Ligase Family Protein5-Formyltetrahydrofolate |
| INV10415720 | 660 | 5 | 7.67 | 0.69 | -0.53 | Putative Thioredoxin Putative |
| INV10406880 | 411 | 3 | 4.78 | 0.69 | -0.53 | Conserved Hypothetical Proteinconserved |
| dltA | 1527 | 12 | 17.75 | 0.69 | -0.53 | Degenerative Transposasedegenerative |
| INV10401310 | 534 | 4 | 6.21 | 0.69 | -0.53 | Putative 5'-Nucleotidase Putative |
| INV10400110 | 1278 | 10 | 14.86 | 0.69 | -0.53 | Hypothetical Proteinhypothetical |
| queA | 1029 | 8 | 11.96 | 0.69 | -0.53 | Glyceraldehyde-3-Phosphate Dehydrogenase, Plasmin Receptorglyceraldehyde-3-Phosphate |
| INV10403990 | 285 | 2 | 3.31 | 0.7 | -0.52 | Conserved Hypothetical Proteinconserved |
| dltD | 1269 | 10 | 14.75 | 0.7 | -0.52 | Deoxyribose-Phosphate Aldolasedeoxyribose-Phosphate |
| IS630-Spn1_transposaseOrf1 | 160 | 1 | 1.86 | 0.7 | -0.52 | 33 Kda Chaperonin (Heat Shock Protein 33 Homolog)33 |
| INV10417200 | 1389 | 11 | 16.15 | 0.7 | -0.52 | Putative Uncharacterized Protein Putative |
| INV10412000 | 774 | 6 | 9 | 0.7 | -0.51 | Putative Uncharacterized Protein (Pseudogene) Putative |
| scaR | 651 | 5 | 7.57 | 0.7 | -0.51 | Riboflavin Biosynthesis Proteinriboflavin |
| INV10404260 | 528 | 4 | 6.14 | 0.7 | -0.51 | Putative Decarboxylase Putative |
| INV10414930 | 528 | 4 | 6.14 | 0.7 | -0.51 | Putative Transport System Permease Putative |
| INV10417660 | 405 | 3 | 4.71 | 0.7 | -0.51 | ABC Transporter ATP-Binding Protein ABC |
| INV10400650 | 1017 | 8 | 11.82 | 0.7 | -0.51 | Putative Septum Formation Initiator Protein Putative |
| INV10409340 | 159 | 1 | 1.85 | 0.7 | -0.51 | Putative 4-Oxalocrotonate Tautomerase Putative |
| rplU | 159 | 1 | 1.85 | 0.7 | -0.51 | PTS System, Nitrogen Regulatory Component IIA, Putativepts |
| 916657_917306 | 649 | 5 | 7.55 | 0.7 | -0.51 |  |
| INV10404230 | 1627 | 13 | 18.92 | 0.7 | -0.51 | Putative Bacteriocin Production Protein Putative |
| N-acetylglucosamine-1-phosphateuridyltransferase(EC2 | 1380 | 11 | 16.04 | 0.7 | -0.51 | Maturase-Related Proteinmaturase-Related |
| INV10411220 | 402 | 3 | 4.67 | 0.71 | -0.5 | Acetyltransferase (GNAT) Family Proteinacetyltransferase |
| msmK | 1131 | 9 | 13.15 | 0.71 | -0.5 | Putative 2-Isopropylmalate Synthase (Pseudogene) Putative |
| INV10416970 | 279 | 2 | 3.24 | 0.71 | -0.5 | Putative Thioredoxin Putative |
| INV10413930 | 521 | 4 | 6.06 | 0.71 | -0.5 | Conserved Hypothetical Proteinconserved |
| trpD | 1005 | 8 | 11.68 | 0.71 | -0.49 | Serine/Threonine-Protein Kinaseserine/Threonine-Protein |
| TypeIrestriction-modificationsystem_specificitysubunitS(EC3 | 641 | 5 | 7.45 | 0.71 | -0.49 | Thiamine-Phosphate Pyrophosphorylasethiamine-Phosphate |
| lacR1 | 762 | 6 | 8.86 | 0.71 | -0.49 | Putative Transposase Putative |
| INV10402640 | 1002 | 8 | 11.65 | 0.71 | -0.49 | Putative Cardiolipin Synthetase Putative |
| INV10413500 | 759 | 6 | 8.82 | 0.71 | -0.49 | Putative Aminodeoxychorismate Lyase Putative |
| INV10414760 | 759 | 6 | 8.82 | 0.71 | -0.49 | Putative Cell-Division Protein Diviva Putative |
| INV10405580 | 999 | 8 | 11.61 | 0.71 | -0.49 | Putative Glutamine-Binding Protein Precursor Putative |
| INV10405710 | 1842 | 15 | 21.42 | 0.71 | -0.49 | Uracil DNA Glycosylase Superfamily Proteinuracil |
| nadC | 873 | 7 | 10.15 | 0.72 | -0.48 | Homoserine O-Succinyltransferasehomoserine |
| INV10405850 | 753 | 6 | 8.75 | 0.72 | -0.48 | Putative Membrane Protein Putative |
| apt | 513 | 4 | 5.96 | 0.72 | -0.48 | Adenine Phosphoribosyltransferaseadenine |
| INV10402350 | 153 | 1 | 1.78 | 0.72 | -0.48 | Putative Protease Putative |
| 434957_435826 | 869 | 7 | 10.1 | 0.72 | -0.47 |  |
| INV10403930 | 869 | 7 | 10.1 | 0.72 | -0.47 | IS1167 Transposase (Pseudogene)IS1167 |
| ilvD | 1704 | 14 | 19.81 | 0.72 | -0.47 | DNA-Binding Protein HUDNA-Binding |
| asnS | 1344 | 11 | 15.63 | 0.72 | -0.47 | Aspartate--Ammonia Ligaseaspartate--Ammonia |
| INV10412030 | 390 | 3 | 4.53 | 0.72 | -0.47 | Lema Family Proteinlema |
| strH | 3939 | 33 | 45.79 | 0.73 | -0.46 | 50S Ribosomal Protein L1450S |
| TelluriteresistanceproteinTehB | 861 | 7 | 10.01 | 0.73 | -0.46 | 50S Ribosomal Protein L2050S |
| INV10414780 | 741 | 6 | 8.61 | 0.73 | -0.46 | Conserved Hypothetical Proteinconserved |
| ccs1 | 150 | 1 | 1.74 | 0.73 | -0.45 | Cytochrome C-Type Biogenesis Protein Ccdacytochrome |
| rpmG1 | 150 | 1 | 1.74 | 0.73 | -0.45 | Phosphoribosylformylglycinamidine Cyclo-Ligasephosphoribosylformylglycinamidine |
| INV10404090 | 1683 | 14 | 19.57 | 0.73 | -0.46 | Response Regulator Proteinresponse |
| INV10408880 | 975 | 8 | 11.34 | 0.73 | -0.46 | Putative Carboxynorspermidine Decarboxylase Putative |
| INV10400490 | 739 | 6 | 8.59 | 0.73 | -0.45 | Impb/Mucb/Samb Family Proteinimpb/Mucb/Samb |
| Phosphoglyceratemutasefamily5 | 621 | 5 | 7.22 | 0.73 | -0.45 | NADH Oxidasenadh |
| unknown | 384 | 3 | 4.46 | 0.73 | -0.45 | Thymidylate Kinasethymidylate |
| INV10412630 | 1083 | 9 | 12.59 | 0.74 | -0.44 | Putative ABC Transporter ATP-Binding Protein (Pseudogene) Putative |
| cadD | 615 | 5 | 7.15 | 0.74 | -0.44 | Blpz Protein, Fusionblpz |
| ptsH | 264 | 2 | 3.07 | 0.74 | -0.44 | Putative Phosphomannomutase Putative |
| INV10414200 | 613 | 5 | 7.13 | 0.74 | -0.44 | IS3-Spn1 Orf BIS3-Spn1 |
| INV10412390 | 729 | 6 | 8.48 | 0.74 | -0.44 | Gntr Family Regulatory Proteingntr |
| INV10412340 | 726 | 6 | 8.44 | 0.74 | -0.43 | ABC Transporter ATP-Binding Protein ABC |
| INV10406230 | 957 | 8 | 11.13 | 0.74 | -0.43 | Conserved Hypothetical Proteinconserved |
| INV10416070 | 957 | 8 | 11.13 | 0.74 | -0.43 | Putative ROK-Family Repressor Protein Putative |
| INV10403710 | 725 | 6 | 8.43 | 0.74 | -0.43 | Pfkb Family Carbohydrate Kinasepfkb |
| INV10414310 | 840 | 7 | 9.77 | 0.74 | -0.43 | Putative Membrane Protein Putative |
| thyA | 840 | 7 | 9.77 | 0.74 | -0.43 | 50S Ribosomal Protein L3250S |
| rluB | 724 | 6 | 8.42 | 0.74 | -0.43 | Peptide Chain Release Factor 1peptide |
| INV10410000 | 1071 | 9 | 12.45 | 0.74 | -0.43 | Putative ABC Transporter Substrate Binding Protein Putative |
| INV10412160 | 375 | 3 | 4.36 | 0.75 | -0.42 | Crcb-Like Proteincrcb-Like |
| INV10418890 | 1410 | 12 | 16.39 | 0.75 | -0.42 | Putative Transferase Putative |
| INV10400600 | 144 | 1 | 1.67 | 0.75 | -0.42 | Integrase/Recombinase (Xerc/Codv Family)Integrase/Recombinase |
| INV10401250 | 1062 | 9 | 12.35 | 0.75 | -0.42 | Putative Exported Protein Putative |
| INV10416580 | 717 | 6 | 8.34 | 0.75 | -0.42 | Putative UDP-Glucose 4-Epimerase Putative |
| INV10418020 | 487 | 4 | 5.66 | 0.75 | -0.41 | Putative Hexulose-6-Phosphate Isomerase (Pseudogene) Putative |
| metA | 945 | 8 | 10.99 | 0.75 | -0.41 | Putative KHG/KDPG Aldolase [Includes: 4-Hydroxy-2-Oxoglutarate Aldolase; 2- Dehydro-3-Deoxy-Phosphogluconate Aldolase] Putative |
| INV10401120 | 601 | 5 | 6.99 | 0.75 | -0.41 | Response Regulator Proteinresponse |
| aroA | 1284 | 11 | 14.93 | 0.75 | -0.41 | Arginyl-Trna Synthetasearginyl-Trna |
| endA | 825 | 7 | 9.59 | 0.76 | -0.4 | DNA Integration/Recombination/Invertion Proteindna |
| INV10415680 | 711 | 6 | 8.27 | 0.76 | -0.41 | Putative Sodium:Dicarboxylate Symporter Family Protein Putative |
| recU | 597 | 5 | 6.94 | 0.76 | -0.4 | DNA Polymerase IDNA |
| INV10413450 | 1506 | 13 | 17.51 | 0.76 | -0.4 | IS1239 Transposaseis1239 |
| fmt | 936 | 8 | 10.88 | 0.76 | -0.4 | FIG01117121: Hypothetical Proteinfig01117121: |
| INV10416560 | 822 | 7 | 9.56 | 0.76 | -0.4 | Putative Marr-Family Transcriptional Regulator Putative |
| rpoB | 3429 | 30 | 39.87 | 0.76 | -0.4 | Putative Parvulin Type Peptidyl-Prolyl Isomerase, Similarity With Prsa Foldase Putative |
| agaD | 819 | 7 | 9.52 | 0.76 | -0.4 | Putative N-Acetylgalactosamine-Specific Phosphotransferase System (PTS), IID Component Putative |
| alaS | 2619 | 23 | 30.45 | 0.76 | -0.39 | Alanyl-Trna Synthetasealanyl-Trna |
| INV10413610 | 477 | 4 | 5.55 | 0.76 | -0.39 | Putative Extracellular Oligopeptide-Binding Protein Putative |
| INV10405150 | 702 | 6 | 8.16 | 0.76 | -0.39 | Putative Membrane Protein (Pseudogene) Putative |
| INV10402930 | 1485 | 13 | 17.26 | 0.77 | -0.38 | Putative Extracellular Solute-Binding Protein (Pseudogene) Putative |
| INV10406560 | 1260 | 11 | 14.65 | 0.77 | -0.38 | Putative Membrane Protein Putative |
| vicX | 810 | 7 | 9.42 | 0.77 | -0.38 | Putative IS1167 Transposase (Pseudogene) Putative |
| INV10414730 | 249 | 2 | 2.89 | 0.77 | -0.37 | Conserved Hypothetical Proteinconserved |
| INV10413090 | 360 | 3 | 4.19 | 0.77 | -0.38 | Apbe Family Proteinapbe |
| rplT | 360 | 3 | 4.19 | 0.77 | -0.38 | PTS System, IIA Componentpts |
| INV10417100 | 2031 | 18 | 23.61 | 0.77 | -0.37 | Putative ABC Transporter, ATP-Binding/Permease Protein Putative |
| 5'-methylthioadenosinenucleosidase(EC3 | 693 | 6 | 8.06 | 0.77 | -0.37 | 5'-Methylthioadenosine Nucleosidase (EC 3.2.2.16) / S-Adenosylhomocysteine Nucleosidase (EC 3.2.2.9)5'-Methylthioadenosine |
| INV10402000 | 693 | 6 | 8.06 | 0.77 | -0.37 | CAAX Amino Terminal Protease Family Proteincaax |
| dnaJ | 1137 | 10 | 13.22 | 0.77 | -0.37 | Putative D-Alanyl-Lipoteichoic Acid Biosynthesis Protein Putative |
| INV10412430 | 801 | 7 | 9.31 | 0.78 | -0.37 | NOL1/NOP2/Sun Family Proteinnol1/NOP2/Sun |
| pyrK | 801 | 7 | 9.31 | 0.78 | -0.37 | Pneumococcal Histidine Triad Protein E Alternate (Pseudogene)Pneumococcal |
| INV10400660 | 2574 | 23 | 29.93 | 0.78 | -0.37 | Conserved Hypothetical Proteinconserved |
| INV10415810 | 246 | 2 | 2.86 | 0.78 | -0.36 | Phage-Like Proteinphage-Like |
| galE | 1020 | 9 | 11.86 | 0.78 | -0.36 | Putative Cell Division Protein Putative |
| INV10407910 | 909 | 8 | 10.57 | 0.78 | -0.36 | DNA Translocase Ftskdna |
| INV10413530 | 576 | 5 | 6.7 | 0.78 | -0.36 | Acetyltransferase, Gnat Family Protein. Acetyltransferase, Gnat Family Proteinacetyltransferase, |
| glnA | 1347 | 12 | 15.66 | 0.78 | -0.36 | Glutamyl-Trna Amidotransferase Subunit Bglutamyl-Trna |
| INV10418380 | 1347 | 12 | 15.66 | 0.78 | -0.36 | Putative Competence Protein Putative |
| INV10400700 | 24 | 0 | 0.28 | 0.78 | -0.36 | Putative IS630-Spn1 Transposase Putative |
| FIG00630611-hypotheticalprotein | 354 | 3 | 4.12 | 0.78 | -0.36 | (3R)-Hydroxymyristoyl-[Acyl Carrier Protein] Dehydratase(3R)-Hydroxymyristoyl-[Acyl |
| radC | 684 | 6 | 7.95 | 0.78 | -0.35 | Fatty Acid/Phospholipid Synthesis Proteinfatty |
| INV10416100 | 243 | 2 | 2.83 | 0.78 | -0.35 | Putative DNA-Binding Protein Putative |
| rpmE | 243 | 2 | 2.83 | 0.78 | -0.35 | Bifunctional Purine Biosynthesis Protein Purh [Includes: Phosphoribosylaminoimidazolecarboxamide Formyltransferase; IMP Cyclohydrolase]Bifunctional |
| arcA | 1230 | 11 | 14.3 | 0.78 | -0.35 | Arginine Deiminasearginine |
| INV10404020 | 1887 | 17 | 21.94 | 0.78 | -0.35 | Putative RNA Methylase Family Protein Putative |
| INV10416150 | 462 | 4 | 5.37 | 0.78 | -0.35 | Conserved Hypothetical Proteinconserved |
| INV10418410 | 1008 | 9 | 11.72 | 0.79 | -0.35 | Conserved Hypothetical Proteinconserved |
| INV10418910 | 1008 | 9 | 11.72 | 0.79 | -0.35 | Putative Cation Transporting Atpase Putative |
| INV10401160 | 678 | 6 | 7.88 | 0.79 | -0.34 | Binding-Protein-Dependent Transport System Membrane Proteinbinding-Protein-Dependent |
| INV10413880 | 678 | 6 | 7.88 | 0.79 | -0.34 | Putative Phosphoglucosamine Mutase Putative |
| INV10418810 | 132 | 1 | 1.53 | 0.79 | -0.34 | Putative Transposase Fragment Putative |
| dltC | 240 | 2 | 2.79 | 0.79 | -0.34 | Phosphopentomutasephosphopentomutase |
| INV10413590 | 783 | 7 | 9.1 | 0.79 | -0.34 | ABC Transporter ATP-Binding Protein (Pseudogene)ABC |
| INV10406100 | 348 | 3 | 4.05 | 0.79 | -0.34 | Response Regulator Proteinresponse |
| agaW | 780 | 7 | 9.07 | 0.79 | -0.33 | Putative N-Acetylgalactosamine-Specific Phosphotransferase System (PTS), IIC Component Putative |
| INV10405310 | 993 | 9 | 11.54 | 0.8 | -0.33 | Putative Type III Restriction Endonuclease Putative |
| INV10409090 | 885 | 8 | 10.29 | 0.8 | -0.33 | Putative Competence-Related Protein Putative |
| hypotheticalprotein | 345 | 3 | 4.01 | 0.8 | -0.32 | UTP-Glucose-1-Phosphate Uridylyltransferaseutp-Glucose-1-Phosphate |
| INV10410290 | 345 | 3 | 4.01 | 0.8 | -0.32 | Conserved Hypothetical Proteinconserved |
| INV10401100 | 236 | 2 | 2.74 | 0.8 | -0.32 | Putative Glycosyl Transferase (Pseudogene) Putative |
| Transposase | 343 | 3 | 3.99 | 0.8 | -0.32 | 30S Ribosomal Protein S1830S |
| INV10419140 | 21 | 0 | 0.24 | 0.8 | -0.31 | Conserved Hypothetical Proteinconserved |
| infC | 342 | 3 | 3.98 | 0.8 | -0.32 | Putative Protease Htpx Homolog Putative |
| 120011_120352 | 341 | 3 | 3.96 | 0.81 | -0.31 |  |
| BlpZprotein_fusion | 234 | 2 | 2.72 | 0.81 | -0.31 | Regulatory Protein Blpsregulatory |
| INV10406850 | 873 | 8 | 10.15 | 0.81 | -0.31 | Branched-Chain Amino Acid Transport ATP-Binding Proteinbranched-Chain |
| INV10413370 | 978 | 9 | 11.37 | 0.81 | -0.31 | Putative Amino Acid ABC Transporter Permease Protein Putative |
| oxidoreductase_GfoIdhMocAfamily | 978 | 9 | 11.37 | 0.81 | -0.31 | Metal Cation ABC Transporter ATP-Binding Proteinmetal |
| INV10413960 | 445 | 4 | 5.17 | 0.81 | -0.3 | Endoribonuclease L-PSP Family Proteinendoribonuclease |
| INV10405880 | 869 | 8 | 10.1 | 0.81 | -0.3 | Putative Uncharacterized Protein Putative |
| INV10416020 | 444 | 4 | 5.16 | 0.81 | -0.3 | Sugar Phosphotransferase System (PTS), IIC Componentsugar |
| INV10416760 | 444 | 4 | 5.16 | 0.81 | -0.3 | Putative Oligopeptide Transporter ATP-Binding Protein Putative |
| cca | 1185 | 11 | 13.78 | 0.81 | -0.3 | Choline Binding Protein Gcholine |
| INV10416900 | 231 | 2 | 2.69 | 0.81 | -0.3 | Recx Family Proteinrecx |
| INV10412200 | 548 | 5 | 6.37 | 0.81 | -0.3 | Putative Uncharacterized Protein (Pseudogene) Putative |
| INV10401300 | 1287 | 12 | 14.96 | 0.81 | -0.3 | Polysaccharide Biosynthesis Proteinpolysaccharide |
| INV10412600 | 1392 | 13 | 16.18 | 0.81 | -0.3 | Putative Membrane Protein (Pseudogene) Putative |
| INV10415540 | 1179 | 11 | 13.71 | 0.82 | -0.29 | Putative Nicotinate-Nucleotide Adenylyltransferase Putative |
| INV10416090 | 966 | 9 | 11.23 | 0.82 | -0.29 | Type IV Prepilin Peptidase Family Proteintype |
| INV10410940 | 1488 | 14 | 17.3 | 0.82 | -0.29 | Putative Ribonucleoside-Diphosphate Reductase Beta Chain Putative |
| INV10410190 | 963 | 9 | 11.2 | 0.82 | -0.29 | Putative GTP-Binding Protein Putative |
| 1258405_1278977 | 20572 | 196 | 239.17 | 0.82 | -0.29 |  |
| INV10413900 | 438 | 4 | 5.09 | 0.82 | -0.28 | Putative Membrane Protein Putative |
| INV10409080 | 857 | 8 | 9.96 | 0.82 | -0.28 | Putative Acetyltransferase (Pseudogene) Putative |
| INV10402670 | 435 | 4 | 5.06 | 0.83 | -0.28 | Conserved Hypothetical Proteinconserved |
| INV10413300 | 849 | 8 | 9.87 | 0.83 | -0.27 | Cell Wall Surface Anchored Proteincell |
| mtsC | 849 | 8 | 9.87 | 0.83 | -0.27 | PTS System, Lichenan-Specific Iic Componentpts |
| INV10415260 | 744 | 7 | 8.65 | 0.83 | -0.27 | ABC Transporter, ATP-Binding Protein ABC |
| transcriptionalregulator_XREfamily | 225 | 2 | 2.62 | 0.83 | -0.27 | 30S Ribosomal Protein S1230S |
| fucA | 639 | 6 | 7.43 | 0.83 | -0.27 | Formamidopyrimidine-DNA Glycosylase (EC 3.2.2.23)Formamidopyrimidine-DNA |
| arcC | 948 | 9 | 11.02 | 0.83 | -0.27 | Carbamate Kinasecarbamate |
| INV10407230 | 741 | 7 | 8.61 | 0.83 | -0.26 | Putative Membrane Protein Putative |
| INV10400380 | 3726 | 36 | 43.32 | 0.83 | -0.26 | Acetolactate Synthase Small Subunitacetolactate |
| FIG01116303-hypotheticalprotein | 531 | 5 | 6.17 | 0.84 | -0.26 | FIG01114970: Hypothetical Proteinfig01114970: |
| INV10406430 | 222 | 2 | 2.58 | 0.84 | -0.25 | Putative Membrane Protein Putative |
| INV10412330 | 936 | 9 | 10.88 | 0.84 | -0.25 | Putative Membrane Protein (Pseudogene) Putative |
| INV10415800 | 1746 | 17 | 20.3 | 0.85 | -0.24 | Conserved Hypothetical Proteinconserved |
| lacC2 | 931 | 9 | 10.82 | 0.85 | -0.24 | DHH Family Proteindhh |
| INV10408060 | 1236 | 12 | 14.37 | 0.85 | -0.24 | Phage/Plasmid Maintenance Toxin/Antidote System Protein (Toxin)Phage/Plasmid |
| aroF1 | 1032 | 10 | 12 | 0.85 | -0.24 | Chorismate Synthasechorismate |
| INV10400710 | 930 | 9 | 10.81 | 0.85 | -0.24 | Putative IS1167 Transposase (Pseudogene) Putative |
| mtsA | 930 | 9 | 10.81 | 0.85 | -0.24 | Prolipoprotein Diacylglyceryl Transferaseprolipoprotein |
| INV10418070 | 1131 | 11 | 13.15 | 0.85 | -0.24 | Sugar Phosphotransferase System (PTS), Lactose/Cellobiose-Specific Family, IIB Subunit Proteinsugar |
| INV10416160 | 522 | 5 | 6.07 | 0.85 | -0.24 | Putative Laci-Family Transcriptional Regulator (Catabolite Control Protein) Putative |
| trsA | 1026 | 10 | 11.93 | 0.85 | -0.23 | Fes Assembly Protein Sufdfes |
| ftsL | 318 | 3 | 3.7 | 0.85 | -0.23 | Isopentenyl-Diphosphate Delta-Isomeraseisopentenyl-Diphosphate |
| INV10407070 | 1023 | 10 | 11.89 | 0.85 | -0.23 | Putative Uncharacterized Protein Putative |
| trpB | 1224 | 12 | 14.23 | 0.85 | -0.23 | Sortase Srtasortase |
| coaA | 921 | 9 | 10.71 | 0.85 | -0.23 | ATP-Dependent Clp Protease ATP-Binding Subunit Clpxatp-Dependent |
| INV10409440 | 216 | 2 | 2.51 | 0.85 | -0.23 | Putative Exported Protein Putative |
| INV10412950 | 618 | 6 | 7.18 | 0.86 | -0.22 | Putative Membrane Protein (Pseudogene) Putative |
| INV10404810 | 1416 | 14 | 16.46 | 0.86 | -0.22 | Putative Membrane Protein Putative |
| INV10413550 | 1113 | 11 | 12.94 | 0.86 | -0.22 | Putative ATP-Binding Protein Putative |
| INV10413050 | 513 | 5 | 5.96 | 0.86 | -0.21 | Haemolysin-III Related Membrane Proteinhaemolysin-III |
| INV10415700 | 1011 | 10 | 11.75 | 0.86 | -0.21 | Putative Marr-Family Transcriptional Regulator Putative |
| INV10416220 | 711 | 7 | 8.27 | 0.86 | -0.21 | Putative Iron ABC Transporter, ATP-Binding Protein Putative |
| vanZ | 511 | 5 | 5.94 | 0.86 | -0.21 | Tn5252, Relaxasetn5252, |
| INV10415350 | 909 | 9 | 10.57 | 0.86 | -0.21 | Putative Membrane Protein Putative |
| plr | 1008 | 10 | 11.72 | 0.86 | -0.21 | N Utilization Substance Protein B Homolog (Nusb Protein)N |
| Tn916_transcriptionalregulator_ putative | 1206 | 12 | 14.02 | 0.87 | -0.21 | 30S Ribosomal Protein S230S |
| INV10416230 | 804 | 8 | 9.35 | 0.87 | -0.2 | Putative Iron ABC Transporter, Solute-Binding Protein Putative |
| INV10401670 | 507 | 5 | 5.89 | 0.87 | -0.2 | Hypothetical Protein (Fragment)Hypothetical |
| INV10417420 | 309 | 3 | 3.59 | 0.87 | -0.2 | Putative P-Loop Hydrolase Putative |
| INV10417430 | 309 | 3 | 3.59 | 0.87 | -0.2 | Putative Membrane Protein Putative |
| scrA | 1884 | 19 | 21.9 | 0.87 | -0.2 | Riboflavin Synthase Alpha Chainriboflavin |
| INV10404110 | 504 | 5 | 5.86 | 0.87 | -0.19 | Hypothetical Proteinhypothetical |
| INV10408120 | 501 | 5 | 5.82 | 0.88 | -0.18 | Putative IS4-Family Transposase (Pseudogene) Putative |
| INV10413380 | 891 | 9 | 10.36 | 0.88 | -0.18 | Conserved Hypothetical Proteinconserved |
| Transcriptionalregulator_CroCIfamily | 207 | 2 | 2.41 | 0.88 | -0.18 | 30S Ribosomal Protein S1030S |
| INV10410750 | 987 | 10 | 11.47 | 0.88 | -0.18 | Putative Glycogen Biosynthesis Protein Putative |
| INV10410410 | 303 | 3 | 3.52 | 0.88 | -0.18 | Conserved Hypothetical Proteinconserved |
| INV10404920 | 885 | 9 | 10.29 | 0.89 | -0.18 | PAP2 Superfamily Proteinpap2 |
| clpP | 591 | 6 | 6.87 | 0.89 | -0.17 | Putative ATP-Dependent Clp Protease ATP-Binding Subunit Putative |
| pta | 975 | 10 | 11.34 | 0.89 | -0.17 | Glucose-6-Phosphate Isomeraseglucose-6-Phosphate |
| INV10413330 | 780 | 8 | 9.07 | 0.89 | -0.16 | Putative Transposase (Pseudogene) Putative |
| 1158723_1159406 | 683 | 7 | 7.94 | 0.89 | -0.16 |  |
| INV10410300 | 683 | 7 | 7.94 | 0.89 | -0.16 | Acetyltransferase (GNAT) Family Proteinacetyltransferase |
| INV10400590 | 297 | 3 | 3.45 | 0.9 | -0.15 | Integraseintegrase |
| INV10404310 | 201 | 2 | 2.34 | 0.9 | -0.15 | Putative Aspartokinase Putative |
| cbpD | 1347 | 14 | 15.66 | 0.9 | -0.15 | Catabolite Control Protein Acatabolite |
| INV10407370 | 3351 | 35 | 38.96 | 0.9 | -0.15 | DJ-1/Pfpi Family Proteindj-1/Pfpi |
| INV10406960 | 582 | 6 | 6.77 | 0.9 | -0.15 | SAM-Dependent Methyltransferasesam-Dependent |
| INV10411790 | 486 | 5 | 5.65 | 0.9 | -0.15 | Putative Membrane Protein Putative |
| INV10409240 | 867 | 9 | 10.08 | 0.9 | -0.15 | Putative Cytochrome C-Type Biogenesis Protein Putative |
| 680188_681241 | 1053 | 11 | 12.24 | 0.91 | -0.14 |  |
| INV10406400 | 483 | 5 | 5.62 | 0.91 | -0.14 | Putative Uncharacterized Protein Putative |
| INV10405350 | 198 | 2 | 2.3 | 0.91 | -0.14 | Acetyltransferase (GNAT) Family Proteinacetyltransferase |
| INV10402730 | 1902 | 20 | 22.11 | 0.91 | -0.14 | Putative Phosphoribulokinase Putative |
| INV10418370 | 858 | 9 | 9.98 | 0.91 | -0.13 | Putative Membrane Protein Putative |
| INV10400570 | 855 | 9 | 9.94 | 0.91 | -0.13 | Integraseintegrase |
| Tn5252_Orf23 | 855 | 9 | 9.94 | 0.91 | -0.13 | DNA-Directed RNA Polymerase Beta' Chaindna-Directed |
| IS861_transposase(orf1)_IS3family_truncated | 196 | 2 | 2.28 | 0.91 | -0.13 | Isoprenylcysteine Carboxyl Methyltransferase (ICMT) Family Proteinisoprenylcysteine |
| ftsW | 1230 | 13 | 14.3 | 0.92 | -0.13 | Putative Putative Folylpolyglutamate Synthase Putative |
| INV10418570 | 1512 | 16 | 17.58 | 0.92 | -0.13 | Marr Family Regulatory Proteinmarr |
| INV10410870 | 945 | 10 | 10.99 | 0.92 | -0.12 | Putative Multidrug Resistance Protein Putative |
| INV10401270 | 570 | 6 | 6.63 | 0.92 | -0.12 | Padr-Like Family Regulatory Proteinpadr-Like |
| Transketolase(EC2 | 1971 | 21 | 22.91 | 0.92 | -0.12 | 30S Ribosomal Protein S1730S |
| gloA | 381 | 4 | 4.43 | 0.92 | -0.12 | Methyltransferase Gidbmethyltransferase |
| INV10415180 | 381 | 4 | 4.43 | 0.92 | -0.12 | Putative Permease Component Of ABC Transporter Putative |
| bacA | 846 | 9 | 9.84 | 0.92 | -0.12 | ATP Synthase Gamma Chainatp |
| INV10414050 | 2709 | 29 | 31.49 | 0.92 | -0.12 | Putative Exported Choline-Binding Glycosyl Hydrolase Putative |
| INV10412100 | 750 | 8 | 8.72 | 0.93 | -0.11 | Conserved Hypothetical Proteinconserved |
| INV10410830 | 657 | 7 | 7.64 | 0.93 | -0.11 | Voltage Gated Chloride Channel Family Proteinvoltage |
| phtE | 564 | 6 | 6.56 | 0.93 | -0.11 | Glutaredoxin-Like Proteinglutaredoxin-Like |
| ppc | 2697 | 29 | 31.36 | 0.93 | -0.11 | Penicillin-Binding Protein 1Bpenicillin-Binding |
| INV10408600 | 1392 | 15 | 16.18 | 0.93 | -0.1 | Sprt-Like Proteinsprt-Like |
| srtA | 744 | 8 | 8.65 | 0.93 | -0.1 | 50S Ribosomal Protein L950S |
| INV10413310 | 375 | 4 | 4.36 | 0.93 | -0.1 |  |
| INV10408140 | 1112 | 12 | 12.93 | 0.93 | -0.1 | Putative Repressor Protein (Pseudogene) Putative |
| INV10404130 | 651 | 7 | 7.57 | 0.93 | -0.1 | Ribonuclease Hiiiribonuclease |
| INV10405340 | 282 | 3 | 3.28 | 0.93 | -0.1 | 6-Phospho-Beta-Glucosidase6-Phospho-Beta-Glucosidase |
| INV10405450 | 558 | 6 | 6.49 | 0.93 | -0.1 | Putative Transposase (Fragment) Putative |
| INV10414640 | 1200 | 13 | 13.95 | 0.94 | -0.09 | Metallo-Beta-Lactamase Superfamily Proteinmetallo-Beta-Lactamase |
| ccmA | 648 | 7 | 7.53 | 0.94 | -0.09 | CCA-Adding Enzymecca-Adding |
| INV10404800 | 1839 | 20 | 21.38 | 0.94 | -0.09 | Conserved Hypothetical Proteinconserved |
| INV10404590 | 372 | 4 | 4.32 | 0.94 | -0.09 | Hypothetical Protein (Pseudogene)Hypothetical |
| INV10412360 | 828 | 9 | 9.63 | 0.94 | -0.09 | Conserved Hypothetical Proteinconserved |
| INV10416800 | 2016 | 22 | 23.44 | 0.94 | -0.09 | Putative Extracellular Oligopeptide-Binding Protein Putative |
| INV10413640 | 462 | 5 | 5.37 | 0.94 | -0.09 | Conserved Hypothetical Protein (Pseudogene)Conserved |
| INV10417090 | 642 | 7 | 7.46 | 0.95 | -0.08 | Putative ABC Transporter, ATP-Binding/Permease Protein (Pseudogene) Putative |
| trcF | 3511 | 39 | 40.82 | 0.96 | -0.06 | 30S Ribosomal Protein S2030S |
| INV10410840 | 633 | 7 | 7.36 | 0.96 | -0.06 | Tyrosine Recombinase Xerctyrosine |
| metE | 2250 | 25 | 26.16 | 0.96 | -0.06 | Galactose-6-Phosphate Isomerase Laca Subunitgalactose-6-Phosphate |
| INV10416540 | 363 | 4 | 4.22 | 0.96 | -0.06 | ABC-Type Glycine Betaine Transport System ATP-Binding Protein ABC-Type |
| INV10404240 | 4941 | 55 | 57.44 | 0.96 | -0.06 | Sodium:Alanine Symporter Family Proteinsodium:Alanine |
| punA | 810 | 9 | 9.42 | 0.96 | -0.06 | Phage Holinphage |
| INV10409740 | 541 | 6 | 6.29 | 0.96 | -0.06 | Alpha-Amylase (Pseudogene)Alpha-Amylase |
| trmD | 720 | 8 | 8.37 | 0.96 | -0.06 | Holliday Junction DNA Helicase Ruvaholliday |
| INV10400740 | 987 | 11 | 11.47 | 0.96 | -0.06 | Deoxyuridine 5'-Triphosphate Nucleotidohydrolasedeoxyuridine |
| INV10414340 | 1518 | 17 | 17.65 | 0.97 | -0.05 | FAD Dependent Oxidoreductasefad |
| fms | 270 | 3 | 3.14 | 0.97 | -0.05 | FIG01116987: Hypothetical Proteinfig01116987: |
| nfsA | 715 | 8 | 8.31 | 0.97 | -0.05 | Trna Delta(2)-Isopentenylpyrophosphate Transferasetrna |
| INV10412470 | 447 | 5 | 5.2 | 0.97 | -0.05 | Putative Membrane Protein Putative |
| scrK | 888 | 10 | 10.32 | 0.97 | -0.04 | Ribosomal RNA Large Subunit Methyltransferase N (EC 2.1.1.-)Ribosomal |
| INV10404030 | 1413 | 16 | 16.43 | 0.98 | -0.04 | Putative Membrane Protein Putative |
| INV10416550 | 972 | 11 | 11.3 | 0.98 | -0.04 | Conserved Hypothetical Proteinconserved |
| INV10404010 | 1323 | 15 | 15.38 | 0.98 | -0.03 | Conserved Hypothetical Proteinconserved |
| INV10405670 | 969 | 11 | 11.27 | 0.98 | -0.03 | Putative Protease Putative |
| INV10414190 | 705 | 8 | 8.2 | 0.98 | -0.03 | Putative Acetyltransferase (GNAT) Family Protein Putative |
| cbpA | 1752 | 20 | 20.37 | 0.98 | -0.03 | Carbamoyl-Phosphate Synthase Large Chaincarbamoyl-Phosphate |
| Phagehyaluronidase | 613 | 7 | 7.13 | 0.98 | -0.02 | N-Ethylammeline Chlorohydrolasen-Ethylammeline |
| INV10407670 | 873 | 10 | 10.15 | 0.99 | -0.02 | Putative Amino Acid ABC Transporter Permease Protein Putative |
| Cytidinedeoxycytidylatedeaminasefamilyprotein | 435 | 5 | 5.06 | 0.99 | -0.01 | Serine Acetyltransferaseserine |
| INV10409450 | 348 | 4 | 4.05 | 0.99 | -0.01 | Putative RNA Methyltransferase Putative |
| tRNA-Leu-AAG | 87 | 1 | 1.01 | 0.99 | -0.01 | Sensor Histidine Kinasesensor |
| INV10416750 | 519 | 6 | 6.03 | 1 | -0.01 | Putative Substrate-Binding Protein (Pseudogene) Putative |
| INV10415270 | 951 | 11 | 11.06 | 1 | -0.01 | Putative Membrane Protein Putative |
| INV10408860 | 432 | 5 | 5.02 | 1 | 0 | Saccharopine Dehydrogenase Family Proteinsaccharopine |
| INV10414550 | 345 | 4 | 4.01 | 1 | 0 | Putative Metallophosphoesterase Protein Putative |
| Glucosamine--fructose-6-phosphateaminotransferase[isomerizing](EC2 | 1809 | 21 | 21.03 | 1 | 0 | Putative Glycogen Phosphorylase Putative |
|  |  |  | 0 | 1 | 0 | Transposasetransposase |
|  |  |  | 0 | 1 | 0 | Transposasetransposase |
|  |  |  | 0 | 1 | 0 | Transposasetransposase |
|  |  |  | 0 | 1 | 0 | Transposasetransposase |
|  |  |  | 0 | 1 | 0 | Transposasetransposase |
|  |  |  | 0 | 1 | 0 | Transposasetransposase |
|  |  |  | 0 | 1 | 0 | Transposasetransposase |
|  |  |  | 0 | 1 | 0 | Transposasetransposase |
|  |  |  | 0 | 1 | 0 | Transposasetransposase |
|  |  |  | 0 | 1 | 0 | Transposase And Inactivated Derivativetransposase |
|  |  |  | 0 | 1 | 0 | Transposase And Inactivated Derivativetransposase |
|  |  |  | 0 | 1 | 0 | Putative Putative Transcription-Repair Coupling Factor Putative |
|  |  |  | 0 | 1 | 0 | Putative Trehalose-6-Phosphate Hydrolase Putative |
|  |  |  | 0 | 1 | 0 | Gntr-Family Transcriptional Regulatorgntr-Family |
|  |  |  | 0 | 1 | 0 | Trna (Guanine-N(1)-)-Methyltransferasetrna |
|  |  |  | 0 | 1 | 0 | Trna Modification Gtpase Trmetrna |
|  |  |  | 0 | 1 | 0 | Trna-Arg-Acgtrna-Arg-ACG |
|  |  |  | 0 | 1 | 0 | Trna-Arg-Acgtrna-Arg-ACG |
|  |  |  | 0 | 1 | 0 | Trna-Arg-Ccgtrna-Arg-CCG |
|  |  |  | 0 | 1 | 0 | Trna-Arg-Ccttrna-Arg-CCT |
|  |  |  | 0 | 1 | 0 | Trna-Arg-Tcttrna-Arg-TCT |
|  |  |  | 0 | 1 | 0 | Trna-Asn-Gtttrna-Asn-GTT |
|  |  |  | 0 | 1 | 0 | Trna-Asn-Gtttrna-Asn-GTT |
|  |  |  | 0 | 1 | 0 | Trna-Cys-Gcatrna-Cys-GCA |
|  |  |  | 0 | 1 | 0 | Trna-Gln-Ttgtrna-Gln-TTG |
|  |  |  | 0 | 1 | 0 | Trna-Gln-Ttgtrna-Gln-TTG |
|  |  |  | 0 | 1 | 0 | Trna-Glu-Ttctrna-Glu-TTC |
|  |  |  | 0 | 1 | 0 | Trna-Glu-Ttctrna-Glu-TTC |
|  |  |  | 0 | 1 | 0 | Trna-Glu-Ttctrna-Glu-TTC |
|  |  |  | 0 | 1 | 0 | Trna-Gly-Gcctrna-Gly-GCC |
|  |  |  | 0 | 1 | 0 | Trna-Gly-Gcctrna-Gly-GCC |
|  |  |  | 0 | 1 | 0 | Trna-Gly-Tcctrna-Gly-TCC |
|  |  |  | 0 | 1 | 0 | Trna-Gly-Tcctrna-Gly-TCC |
|  |  |  | 0 | 1 | 0 | Trna-His-Gtgtrna-His-GTG |
|  |  |  | 0 | 1 | 0 | Trna-Ile-Gattrna-Ile-GAT |
|  |  |  | 0 | 1 | 0 | Trna-Ile-Gattrna-Ile-GAT |
|  |  |  | 0 | 1 | 0 | Trna-Leu-Aagtrna-Leu-AAG |
|  |  |  | 0 | 1 | 0 | Trna-Leu-Caatrna-Leu-CAA |
|  |  |  | 0 | 1 | 0 | Trna-Leu-Taatrna-Leu-TAA |
|  |  |  | 0 | 1 | 0 | Trna-Leu-Taatrna-Leu-TAA |
|  |  |  | 0 | 1 | 0 | Trna-Met-Cattrna-Met-CAT |
|  |  |  | 0 | 1 | 0 | Trna-Met-Cattrna-Met-CAT |
|  |  |  | 0 | 1 | 0 | Trna-Met-Cattrna-Met-CAT |
|  |  |  | 0 | 1 | 0 | Trna-Phe-Gaatrna-Phe-GAA |
|  |  |  | 0 | 1 | 0 | Trna-Phe-Gaatrna-Phe-GAA |
|  |  |  | 0 | 1 | 0 | Trna-Pro-Tggtrna-Pro-TGG |
|  |  |  | 0 | 1 | 0 | Trna-Pro-Tggtrna-Pro-TGG |
|  |  |  | 0 | 1 | 0 | Trna-Ser-Gcttrna-Ser-GCT |
|  |  |  | 0 | 1 | 0 | Trna-Ser-Tgatrna-Ser-TGA |
|  |  |  | 0 | 1 | 0 | Trna-Ser-Tgatrna-Ser-TGA |
|  |  |  | 0 | 1 | 0 | Trna-Thr-Ggttrna-Thr-GGT |
|  |  |  | 0 | 1 | 0 | Trna-Trp-Ccatrna-Trp-CCA |
|  |  |  | 0 | 1 | 0 | Trna-Tyr-Gtatrna-Tyr-GTA |
|  |  |  | 0 | 1 | 0 | Trna-Tyr-Gtatrna-Tyr-GTA |
|  |  |  | 0 | 1 | 0 | Trna-Val-Tactrna-Val-TAC |
|  |  |  | 0 | 1 | 0 | Tryptophan Synthase Alpha Chaintryptophan |
|  |  |  | 0 | 1 | 0 | Tryptophan Synthase Beta Chaintryptophan |
|  |  |  | 0 | 1 | 0 | Indole-3-Glycerol Phosphate Synthaseindole-3-Glycerol |
|  |  |  | 0 | 1 | 0 | Anthranilate Phosphoribosyltransferaseanthranilate |
|  |  |  | 0 | 1 | 0 | Anthranilate Synthase Component Ianthranilate |
|  |  |  | 0 | 1 | 0 | N-(5'-Phosphoribosyl)Anthranilate Isomerasen-(5'-Phosphoribosyl)Anthranilate |
|  |  |  | 0 | 1 | 0 | Anthranilate Synthase Component Iianthranilate |
|  |  |  | 0 | 1 | 0 | Tryptophanyl-Trna Synthetasetryptophanyl-Trna |
|  |  |  | 0 | 1 | 0 | Trse-Like Proteintrse-Like |
|  |  |  | 0 | 1 | 0 | Trna Pseudouridine Synthase Atrna |
|  |  |  | 0 | 1 | 0 | Trna Pseudouridine Synthase Btrna |
|  |  |  | 0 | 1 | 0 | Elongation Factor Tselongation |
|  |  |  | 0 | 1 | 0 | Elongation Factor Tu (EF-Tu)Elongation |
|  |  |  | 0 | 1 | 0 | Two-Component Response Regulatortwo-Component |
|  |  |  | 0 | 1 | 0 | Type I Restriction-Modification System, DNA-Methyltransferase Subunit M (EC 2.1.1.72)Type |
|  |  |  | 0 | 1 | 0 | Type I Restriction-Modification System, Specificity Subunit S (EC 3.1.21.3)Type |
|  |  |  | 0 | 1 | 0 | Type I Restriction-Modification System, Specificity Subunit S (EC 3.1.21.3)Type |
|  |  |  | 0 | 1 | 0 | Prephenate Dehydrogenaseprephenate |
|  |  |  | 0 | 1 | 0 | Putative Tyrosyl-Trna Synthetase Putative |
|  |  |  | 0 | 1 | 0 | Uridine Kinaseuridine |
|  |  |  | 0 | 1 | 0 | UDP-Glucose 4-Epimerase (EC 5.1.3.2)UDP-Glucose |
|  |  |  | 0 | 1 | 0 | UDP-Glucose 6-Dehydrogenaseudp-Glucose |
|  |  |  | 0 | 1 | 0 | UDP-Glucose 6-Dehydrogenase Ugdudp-Glucose |
|  |  |  | 0 | 1 | 0 | Putative Unsaturated Glucuronyl Hydrolase Putative |
|  |  |  | 0 | 1 | 0 | Undecaprenyl Pyrophosphate Phosphataseundecaprenyl |
|  |  |  | 0 | 1 | 0 | Undecaprenyl Pyrophosphate Synthetase (EC 2.5.1.31)Undecaprenyl |
|  |  |  | 0 | 1 | 0 | Uracil-DNA Glycosylaseuracil-DNA |
|  |  |  | 0 | 1 | 0 | Unknownunknown |
|  |  |  | 0 | 1 | 0 | Unknownunknown |
|  |  |  | 0 | 1 | 0 | Uracil Phosphoribosyltransferaseuracil |
|  |  |  | 0 | 1 | 0 | USG Proteinusg |
|  |  |  | 0 | 1 | 0 | Uvrabc System Protein A (Uvra Protein)Uvrabc |
|  |  |  | 0 | 1 | 0 | Uvrabc System Protein B (Uvrb Protein)Uvrabc |
|  |  |  | 0 | 1 | 0 | Uvrabc DNA Repair System Protein Cuvrabc |
|  |  |  | 0 | 1 | 0 | Valyl-Trna Synthetasevalyl-Trna |
|  |  |  | 0 | 1 | 0 | Putative Vanz-Family Resistance Protein Putative |
|  |  |  | 0 | 1 | 0 | Conserved Hypothetical Proteinconserved |
|  |  |  | 0 | 1 | 0 | Sensor Histidine Kinasesensor |
|  |  |  | 0 | 1 | 0 | Response Regulator Proteinresponse |
|  |  |  | 0 | 1 | 0 | Metallo-Beta-Lactamase Superfamily Proteinmetallo-Beta-Lactamase |
|  |  |  | 0 | 1 | 0 | Galacturonosyl Transferasegalacturonosyl |
|  |  |  | 0 | 1 | 0 | Galacturonic Acid Acetyl Transferasegalacturonic |
|  |  |  | 0 | 1 | 0 | Putative Glycosyl Transferase Putative |
|  |  |  | 0 | 1 | 0 | Capsular Polysaccharide Biosynthesis Protein Wzdcapsular |
|  |  |  | 0 | 1 | 0 | Tyrosine-Protein Kinase Wzetyrosine-Protein |
|  |  |  | 0 | 1 | 0 | Integral Membrane Regulatory Protein Wzgintegral |
|  |  |  | 0 | 1 | 0 | Protein-Tyrosine Phosphatase Wzhprotein-Tyrosine |
|  |  |  | 0 | 1 | 0 | Flippase Wzxflippase |
|  |  |  | 0 | 1 | 0 | Oligosaccharide Repeat Unit Polymerase Wzyoligosaccharide |
|  |  |  | 0 | 1 | 0 | Tyrosine Recombinase Xerdtyrosine |
|  |  |  | 0 | 1 | 0 | Putative Xanthine Phosphoribosyltransferase Putative |
|  |  |  | 0 | 1 | 0 | Putative Exodeoxyribonuclease VII Large Subunit Putative |
|  |  |  | 0 | 1 | 0 | Putative Exodeoxyribonuclease VII Small Subunit Putative |
|  |  |  | 0 | 1 | 0 | Zeta Toxinzeta |
|  |  |  | 0 | 1 | 0 | Zeta Toxinzeta |
|  |  |  | 0 | 1 | 0 | Zinc Metalloprotease Zmpb Precursor (EC 3.4.24.-)Zinc |
|  |  |  | 0 | 1 | 0 | Iga-Proteaseiga-Protease |
|  |  |  | 0 | 1 | 0 | Zinc Metalloprotease Bzinc |
|  |  |  | 0 | 1 | 0 | Glucose-6-Phosphate 1-Dehydrogenaseglucose-6-Phosphate |
| INV10412550 | 258 | 3 | 3 | 1 | 0 | Nicotinate Phosphoribosyltransferase (Naprtase) Family Proteinnicotinate |
| INV10405470 | 1461 | 17 | 16.99 | 1 | 0 | ABC Transporter, ATP-Binding Protein ABC |
| INV10416250 | 945 | 11 | 10.99 | 1 | 0 | Hypotheical Proteinhypotheical |
| INV10408910 | 1632 | 19 | 18.97 | 1 | 0 | Haloacid Dehalogenase-Like Hydrolasehaloacid |
| INV10401240 | 1374 | 16 | 15.97 | 1 | 0 | Putative Membrane Protein Putative |
| INV10416710 | 1372 | 16 | 15.95 | 1 | 0 | ABC Transporter ATP-Binding Protein ABC |
| MultidrugresistanceeffluxpumpPmrA | 1200 | 14 | 13.95 | 1 | 0 | DNA Ligasedna |
| Positivetranscriptionalregulator_MutRfamily | 854 | 10 | 9.93 | 1.01 | 0.01 | Oxygen-Insensitive NAD(P)H Nitroreductase (EC 1.-.-.-) / Dihydropteridine Reductase (EC 1.5.1.34)Oxygen-Insensitive |
| groEL | 1623 | 19 | 18.87 | 1.01 | 0.01 | Glucosamine--Fructose-6-Phosphate Aminotransferase [Isomerizing] (EC 2.6.1.16)Glucosamine--Fructose-6-Phosphate |
| accA | 768 | 9 | 8.93 | 1.01 | 0.01 | Acetyl-Coenzyme A Carboxylase Carboxyl Transferase Subunit Alphaacetyl-Coenzyme |
| FIG01114020-hypotheticalprotein | 597 | 7 | 6.94 | 1.01 | 0.01 | Ferrochelatase, Protoheme Ferro-Lyase (EC 4.99.1.1)Ferrochelatase, |
| INV10400870 | 170 | 2 | 1.98 | 1.01 | 0.01 | Bacteriocin Transport Accessory Proteinbacteriocin |
| INV10405720 | 255 | 3 | 2.96 | 1.01 | 0.01 | Putative Uncharacterized Protein Putative |
| INV10405840 | 765 | 9 | 8.89 | 1.01 | 0.02 | ABC Transporter ATP-Binding Protein ABC |
| INV10413690 | 339 | 4 | 3.94 | 1.01 | 0.02 | Conserved Hypothetical Proteinconserved |
| aspC | 1188 | 14 | 13.81 | 1.01 | 0.02 | Asparaginyl-Trna Synthetaseasparaginyl-Trna |
| nusB | 423 | 5 | 4.92 | 1.01 | 0.02 | Phospho-N-Acetylmuramoyl-Pentapeptide-Transfera Sephospho-N-Acetylmuramoyl-Pentapeptide-Transfera |
| INV10407800 | 504 | 6 | 5.86 | 1.02 | 0.03 | Putative Extracellular Oligopeptide-Binding Protein Putative |
| INV10416530 | 504 | 6 | 5.86 | 1.02 | 0.03 | ABC-Type Glycine Betaine Transport System Protein ABC-Type |
| ZincmetalloproteasezmpBprecursor(EC3 | 5214 | 62 | 60.62 | 1.02 | 0.03 | Transcription Regulator, Probabletranscription |
| INV10412250 | 585 | 7 | 6.8 | 1.03 | 0.04 | Putative Membrane Protein Putative |
| INV10416660 | 585 | 7 | 6.8 | 1.03 | 0.04 | Putative Phosphoesterase Putative |
| nanB | 2093 | 25 | 24.33 | 1.03 | 0.04 | Methionyl-Trna Synthetasemethionyl-Trna |
| INV10416600 | 333 | 4 | 3.87 | 1.03 | 0.04 | Putative Iron Compound ABC Transporter, Permease Protein Putative |
| INV10417930 | 249 | 3 | 2.89 | 1.03 | 0.04 | Putative Membrane Protein Putative |
| INV10412230 | 1755 | 21 | 20.4 | 1.03 | 0.04 | Putative Uncharacterized Protein Putative |
| INV10413840 | 165 | 2 | 1.92 | 1.03 | 0.04 | ABC Transporter ATP-Binding Protein ABC |
| INV10415390 | 165 | 2 | 1.92 | 1.03 | 0.04 | Putative IS1381 Transposase Orfa (Pseudogene) Putative |
| INV10413940 | 414 | 5 | 4.81 | 1.03 | 0.05 | Conserved Hypothetical Proteinconserved |
| INV10412420 | 744 | 9 | 8.65 | 1.04 | 0.05 | Putative Phosphate ABC Transporter Permease Protein Putative |
| INV10414940 | 909 | 11 | 10.57 | 1.04 | 0.05 | Putative Transport System Permease Putative |
| ABCtransporterpermeaseprotein | 738 | 9 | 8.58 | 1.04 | 0.06 | ABC Transporter Permease Protein ABC |
| INV10418600 | 900 | 11 | 10.46 | 1.05 | 0.07 | Multi Antimicrobial Extrusion (MATE) Family Transportermulti |
| scpA | 729 | 9 | 8.48 | 1.06 | 0.08 | Putative Riboflavin Biosynthesis Protein Putative |
| INV10402090 | 321 | 4 | 3.73 | 1.06 | 0.08 | Putative Membrane Protein Putative |
| INV10406500 | 321 | 4 | 3.73 | 1.06 | 0.08 | Putative Amino-Acid ABC Transporter Integral Membrane Protein Putative |
| INV10410950 | 804 | 10 | 9.35 | 1.06 | 0.09 | Putative Aldose 1-Epimerase 2 (Pseudogene) Putative |
| hemH | 721 | 9 | 8.38 | 1.07 | 0.09 | Gp5gp5 |
| INV10402600 | 477 | 6 | 5.55 | 1.07 | 0.1 | Conserved Hypothetical Proteinconserved |
| INV10413710 | 477 | 6 | 5.55 | 1.07 | 0.1 | Putative S1 RNA Binding Domain Protein Putative |
| INV10406540 | 1200 | 15 | 13.95 | 1.07 | 0.1 | Putative Membrane Protein Putative |
| INV10416910 | 1038 | 13 | 12.07 | 1.07 | 0.1 | Conserved Hypothetical Proteinconserved |
| FIG01115377-hypotheticalprotein | 315 | 4 | 3.66 | 1.07 | 0.1 | FIG01114205: Hypothetical Proteinfig01114205: |
| folE | 555 | 7 | 6.45 | 1.07 | 0.1 | FIG01118148: Hypothetical Proteinfig01118148: |
| tRNA-Asn-GTT | 74 | 1 | 0.86 | 1.08 | 0.1 | Putative Sucrose-Specific Phosphotransferase System (PTS), IIABC Component Putative |
| tRNA-Val-TAC | 74 | 1 | 0.86 | 1.08 | 0.1 | Regulatory Protein Spxregulatory |
| INV10416810 | 633 | 8 | 7.36 | 1.08 | 0.11 | Putative Polysaccharide Repeat Unit Polymerase Putative |
| INV10408660 | 553 | 7 | 6.43 | 1.08 | 0.11 | Putative Membrane Protein Putative |
| kgdA | 630 | 8 | 7.32 | 1.08 | 0.11 | ABC Transporter, ATP-Binding Protein ABC |
| INV10414920 | 1107 | 14 | 12.87 | 1.08 | 0.11 | Conserved Hypothetical Proteinconserved |
| INV10415450 | 708 | 9 | 8.23 | 1.08 | 0.12 | Conserved Hypothetical Proteinconserved |
| INV10403160 | 5304 | 67 | 61.66 | 1.09 | 0.12 | Putative Arac-Family Transcriptional Regulator Putative |
| FIG01114689-hypotheticalprotein | 231 | 3 | 2.69 | 1.09 | 0.12 | FIG00630611: Hypothetical Proteinfig00630611: |
| gmk | 627 | 8 | 7.29 | 1.09 | 0.12 | Merr Family Regulatory Proteinmerr |
| tRNA-Glu-TTC | 72 | 1 | 0.84 | 1.09 | 0.12 | Putative Sucrose Operon Repressor Putative |
| lysS | 1491 | 19 | 17.33 | 1.09 | 0.13 | IS1167, Transposaseis1167, |
| pspA | 1956 | 25 | 22.74 | 1.1 | 0.13 | Putative Xaa-His Dipeptidase Putative |
| INV10405860 | 699 | 9 | 8.13 | 1.1 | 0.13 | Putative Membrane Protein Putative |
| INV10412310 | 699 | 9 | 8.13 | 1.1 | 0.13 | Conserved Hypothetical Proteinconserved |
| INV10405950 | 540 | 7 | 6.28 | 1.1 | 0.14 | Sugar Phosphotransferase System (PTS), Galactitol-Specific Family, IIC Componentsugar |
| INV10401350 | 459 | 6 | 5.34 | 1.1 | 0.14 |  |
| recG | 2016 | 26 | 23.44 | 1.1 | 0.14 | Putative Immunity Protein Pncm Putative |
| nanA | 3183 | 41 | 37.01 | 1.11 | 0.14 | 5,10-Methylenetetrahydrofolate Reductase5,10-Methylenetetrahydrofolate |
| INV10405890 | 769 | 10 | 8.94 | 1.11 | 0.15 | Putative Uncharacterized Protein Putative |
| INV10418850 | 147 | 2 | 1.71 | 1.11 | 0.15 | Putative Peptidase Putative |
| INV10412590 | 844 | 11 | 9.81 | 1.11 | 0.15 | Putative DNA-Binding Protein Putative |
| INV10413910 | 843 | 11 | 9.8 | 1.11 | 0.15 | Pyridine Nucleotide-Disulphide Oxidoreductase Family Proteinpyridine |
| INV10408570 | 301 | 4 | 3.5 | 1.11 | 0.15 | Putative Membrane Protein Putative |
| lmb | 918 | 12 | 10.67 | 1.11 | 0.16 | Putative Membrane Protein Putative |
| nox | 1380 | 18 | 16.04 | 1.11 | 0.16 | Mobile Element Proteinmobile |
| INV10408620 | 2532 | 33 | 29.44 | 1.12 | 0.16 | Putative Membrane Protein Putative |
| INV10406900 | 1679 | 22 | 19.52 | 1.12 | 0.16 | Putative Glucose-Specific Phosphotransferase System (PTS), IIABC Component Putative |
| INV10414790 | 1065 | 14 | 12.38 | 1.12 | 0.16 | Putative Membrane Protein Putative |
| INV10416030 | 297 | 4 | 3.45 | 1.12 | 0.17 | Putative Lactose/Cellobiose-Specific Phosphotransferase System (PTS), IIA Component Putative |
| INV10405900 | 678 | 9 | 7.88 | 1.13 | 0.17 | Putative Surface-Anchored Serine Protease Putative |
| INV10416210 | 1968 | 26 | 22.88 | 1.13 | 0.18 | Putative Inner Membrane Component Of ABC Transporter Putative |
| INV10407300 | 825 | 11 | 9.59 | 1.13 | 0.18 | Putative NUDIX Family Protein Putative |
| 1879127_1879496 | 369 | 5 | 4.29 | 1.13 | 0.18 |  |
| INV10400080 | 369 | 5 | 4.29 | 1.13 | 0.18 | Hypothetical Proteinhypothetical |
| INV10417340 | 369 | 5 | 4.29 | 1.13 | 0.18 | Putative DNA-Binding Protein Putative |
| INV10417770 | 369 | 5 | 4.29 | 1.13 | 0.18 | Haloacid Dehalogenase-Like Hydrolasehaloacid |
| INV10407900 | 672 | 9 | 7.81 | 1.13 | 0.18 | Deor Family Regulatory Proteindeor |
| INV10409510 | 897 | 12 | 10.43 | 1.14 | 0.19 | Ferric Siderophore ABC Transporter, Permease Proteinferric |
| INV10412320 | 897 | 12 | 10.43 | 1.14 | 0.19 | Conserved Hypothetical Proteinconserved |
| INV10412010 | 738 | 10 | 8.58 | 1.15 | 0.2 | ABC Transporter, ATP-Binding Protein ABC |
| phpA | 2535 | 34 | 29.47 | 1.15 | 0.2 | Putative Anaerobic Ribonucleoside-Triphosphate Reductase Activating Protein Putative |
| pepC | 1335 | 18 | 15.52 | 1.15 | 0.2 | Mutt/Nudix Family Proteinmutt/Nudix |
| xseB | 213 | 3 | 2.48 | 1.15 | 0.2 | Transcriptional Regulator Orfxtranscriptional |
| INV10414260 | 885 | 12 | 10.29 | 1.15 | 0.2 | Putative Membrane Protein Putative |
| glnQ3 | 735 | 10 | 8.55 | 1.15 | 0.2 | NADP-Specific Glutamate Dehydrogenasenadp-Specific |
| alr | 1104 | 15 | 12.84 | 1.16 | 0.21 | Alanine Racemasealanine |
| pepV | 1401 | 19 | 16.29 | 1.16 | 0.21 | Phosphomevalonate Kinasephosphomevalonate |
| INV10410790 | 360 | 5 | 4.19 | 1.16 | 0.21 | Putative Uncharacterized Protein Putative |
| INV10410850 | 360 | 5 | 4.19 | 1.16 | 0.21 | Putative Lipoate-Protein Ligase A Putative |
| Tn5252_Orf10protein | 358 | 5 | 4.16 | 1.16 | 0.22 | DNA-Directed RNA Polymerase Beta Chaindna-Directed |
| leuA | 1097 | 15 | 12.75 | 1.16 | 0.22 | Putative Membrane Protein Putative |
| INV10415760 | 2127 | 29 | 24.73 | 1.17 | 0.22 | Putative Oligopeptidase Putative |
| dexB | 1608 | 22 | 18.69 | 1.17 | 0.22 | Degenerate Transposasedegenerate |
| INV10410110 | 576 | 8 | 6.7 | 1.17 | 0.23 | Putative Glycosyltransferase Putative |
| mtsB | 723 | 10 | 8.41 | 1.17 | 0.23 | CTP:Phosphocholine Cytidylyltransferasectp:Phosphocholine |
| INV10400640 | 205 | 3 | 2.38 | 1.18 | 0.24 | S4 Domain Containing Proteins4 |
| INV10413890 | 1074 | 15 | 12.49 | 1.19 | 0.25 | Putative Exported Protein Putative |
| INV10406250 | 711 | 10 | 8.27 | 1.19 | 0.25 | Putative Ribosomal Small Subunit Pseudouridine Synthase A Putative |
| agaV | 492 | 7 | 5.72 | 1.19 | 0.25 | Putative N-Acetylgalactosamine-Specific Phosphotransferase System (PTS), IIB Component Putative |
| tpx | 492 | 7 | 5.72 | 1.19 | 0.25 | 30S Ribosomal Protein S630S |
| INV10412350 | 636 | 9 | 7.39 | 1.19 | 0.25 | Putative Alpha-Amylase Putative |
| valS | 2652 | 37 | 30.83 | 1.19 | 0.26 | Tn5252, Relaxasetn5252, |
| INV10416570 | 346 | 5 | 4.02 | 1.19 | 0.26 | Conserved Hypothetical Proteinconserved |
| INV10408560 | 201 | 3 | 2.34 | 1.2 | 0.26 | Putative Uncharacterized Protein Putative |
| INV10418420 | 201 | 3 | 2.34 | 1.2 | 0.26 | Putative Zinc-Binding Alcohol Dehydrogenase Putative |
| INV10408590 | 558 | 8 | 6.49 | 1.2 | 0.26 | Putative Transcription Accessory Protein Putative |
| INV10417080 | 414 | 6 | 4.81 | 1.2 | 0.27 | Conserved Hypothetical Proteinconserved |
| probablyaromaticringhydroxylatingenzyme_evidencedbyCOGnitorPaaD-likeprotein(DUF59)involvedinFe-Sclusterassembly | 342 | 5 | 3.98 | 1.21 | 0.27 | Putative Glutamyl-Aminopeptidase Putative |
| INV10414520 | 627 | 9 | 7.29 | 1.21 | 0.27 | Response Regulator Proteinresponse |
| INV10413360 | 912 | 13 | 10.6 | 1.21 | 0.27 | Putative Amino Acid ABC Transporter ATP-Binding Protein Putative |
| INV10410800 | 270 | 4 | 3.14 | 1.21 | 0.27 | Putative Gtpase Protein Putative |
| rpsO | 270 | 4 | 3.14 | 1.21 | 0.27 | Ribosome-Binding Factor Aribosome-Binding |
| INV10404400 | 1050 | 15 | 12.21 | 1.21 | 0.28 | Conserved Hypothetical Proteinconserved |
| fucK | 1404 | 20 | 16.32 | 1.21 | 0.28 | Putative Fructose-Specific Phosphotransferase System (PTS), IIABC Component Putative |
| INV10413870 | 975 | 14 | 11.34 | 1.22 | 0.28 | Conserved Hypothetical Proteinconserved |
| INV10404080 | 549 | 8 | 6.38 | 1.22 | 0.29 | Sensor Histidine Kinasesensor |
| hasC | 900 | 13 | 10.46 | 1.22 | 0.29 | Glutathione Reductaseglutathione |
| tpi | 759 | 11 | 8.82 | 1.22 | 0.29 | 30S Ribosomal Protein S530S |
| INV10416080 | 618 | 9 | 7.18 | 1.22 | 0.29 | Acetyltransferase, GNAT Familyacetyltransferase, |
| INV10412130 | 1461 | 21 | 16.99 | 1.22 | 0.29 | Haloacid Dehalogenase-Like Hydrolasehaloacid |
| exoA | 828 | 12 | 9.63 | 1.22 | 0.29 | Dna/Rna Helicase (Dead/Deah Box Family)Dna/Rna |
| INV10413030 | 195 | 3 | 2.27 | 1.22 | 0.29 | Acetyltransferase (GNAT) Family Proteinacetyltransferase |
| INV10408890 | 615 | 9 | 7.15 | 1.23 | 0.3 | Porphyromonas-Type Peptidyl-Arginine Deiminaseporphyromonas-Type |
| spi | 615 | 9 | 7.15 | 1.23 | 0.3 | 50S Ribosomal Protein L350S |
| INV10418500 | 2646 | 38 | 30.76 | 1.23 | 0.3 | Acyltransferase Family Proteinacyltransferase |
| INV10407770 | 192 | 3 | 2.23 | 1.24 | 0.31 | ABC Transporter Permease Protein ABC |
| INV10413470 | 678 | 10 | 7.88 | 1.24 | 0.31 | Putative Acetyltransferase Putative |
| Dephospho-CoAkinase(EC2 | 606 | 9 | 7.05 | 1.24 | 0.31 | Degenerate Transposasedegenerate |
| adhP | 1020 | 15 | 11.86 | 1.24 | 0.32 | Alcohol Dehydrogenasealcohol |
| INV10408650 | 536 | 8 | 6.23 | 1.24 | 0.32 | Putative Membrane Protein Putative |
| RibosomalRNAlargesubunitmethyltransferaseN(EC2 | 1086 | 16 | 12.63 | 1.25 | 0.32 | Putative Phosphoenolpyruvate Carboxylase Putative |
| lspA | 462 | 7 | 5.37 | 1.26 | 0.33 | Tetr Family Regulatory Proteintetr |
| manM | 804 | 12 | 9.35 | 1.26 | 0.33 | IS630-Spn1, Transposase Orf2IS630-Spn1, |
| INV10413850 | 324 | 5 | 3.77 | 1.26 | 0.33 | Degv Family Proteindegv |
| INV10406650 | 870 | 13 | 10.11 | 1.26 | 0.33 | Putative Glyoxalase Family Protein Putative |
| Bacteriocin-likepeptideMBlpM | 255 | 4 | 2.96 | 1.26 | 0.34 | Undecaprenol Kinase (Bacitracin Resistance Protein)Undecaprenol |
| INV10415790 | 1752 | 26 | 20.37 | 1.26 | 0.34 | Putative Peptidase Putative |
| 3'-to-5'exoribonucleaseRNaseR | 2355 | 35 | 27.38 | 1.27 | 0.34 | 3'-To-5' Exoribonuclease Rnase R3'-To-5' |
| FIG01115706-hypotheticalprotein | 117 | 2 | 1.36 | 1.27 | 0.35 | FIG01114589: Hypothetical Proteinfig01114589: |
| Transcriptionalregulator | 386 | 6 | 4.49 | 1.28 | 0.35 | 30S Ribosomal Protein S830S |
| INV10414410 | 453 | 7 | 5.27 | 1.28 | 0.35 | Putative Membrane Protein Putative |
| INV10402510 | 318 | 5 | 3.7 | 1.28 | 0.35 | Putative Lipoprotein Putative |
| INV10408000 | 318 | 5 | 3.7 | 1.28 | 0.35 | Putative Cysteine Desulfhydrase Putative |
| INV10415500 | 318 | 5 | 3.7 | 1.28 | 0.35 | Putative Isochorismatase Putative |
| 849941_850659 | 718 | 11 | 8.35 | 1.28 | 0.36 |  |
| INV10407830 | 718 | 11 | 8.35 | 1.28 | 0.36 | Putative Membrane Protein Putative |
| INV10415550 | 447 | 7 | 5.2 | 1.29 | 0.37 | Conserved Hypothetical Proteinconserved |
| INV10413040 | 513 | 8 | 5.96 | 1.29 | 0.37 | Conserved Hypothetical Proteinconserved |
| INV10412170 | 577 | 9 | 6.71 | 1.3 | 0.38 | Chorismate Mutase Type II Proteinchorismate |
| INV10404320 | 510 | 8 | 5.93 | 1.3 | 0.38 | Enoyl-Coa Hydratase/Isomerase Family Proteinenoyl-Coa |
| INV10402650 | 642 | 10 | 7.46 | 1.3 | 0.38 | Putative Membrane Protein Putative |
| INV10418720 | 774 | 12 | 9 | 1.3 | 0.38 | Putative Lipoprotein Putative |
| INV10404790 | 840 | 13 | 9.77 | 1.3 | 0.38 | Putative Cation Transport Protein Putative |
| INV10418750 | 705 | 11 | 8.2 | 1.3 | 0.38 | ABC Transporter Permease Protein ABC |
| fucU | 441 | 7 | 5.13 | 1.31 | 0.38 | Putative 1-Phosphofructokinase Putative |
| licC | 1296 | 20 | 15.07 | 1.31 | 0.39 | ABC Transporter, ATP-Binding Protein ABC |
| dapB | 768 | 12 | 8.93 | 1.31 | 0.39 | Cysteine ABC Transporter, Substrate-Binding Proteincysteine |
| INV10417330 | 963 | 15 | 11.2 | 1.31 | 0.39 | Putative Uncharacterized Protein Putative |
| INV10413790 | 2337 | 36 | 27.17 | 1.31 | 0.39 | Putative Glutathione S-Transferase Putative |
| INV10415840 | 240 | 4 | 2.79 | 1.32 | 0.4 | Beta-Fructofuranosidasebeta-Fructofuranosidase |
| pstI | 1734 | 27 | 20.16 | 1.32 | 0.4 | Pyruvate Formate-Lyase Activating Enzymepyruvate |
| secA | 2514 | 39 | 29.23 | 1.32 | 0.4 | Glucose-1-Phosphate Thymidylyltransferase Rmlaglucose-1-Phosphate |
| INV10413150 | 366 | 6 | 4.26 | 1.33 | 0.41 | Putative NADPH-Dependent FMN Reductase Putative |
| INV10418580 | 1332 | 21 | 15.49 | 1.33 | 0.42 | Putative Exported Protein Putative |
| rrf | 558 | 9 | 6.49 | 1.34 | 0.42 | Putative Recombination Protein U Putative |
| INV10401130 | 750 | 12 | 8.72 | 1.34 | 0.42 | Histidine Kinase Proteinhistidine |
| acpS | 363 | 6 | 4.22 | 1.34 | 0.42 | Holo-[Acyl-Carrier Protein] Synthaseholo-[Acyl-Carrier |
| INV10417780 | 426 | 7 | 4.95 | 1.34 | 0.43 | Putative Preprotein Translocase Sece Subunit Putative |
| rplK | 426 | 7 | 4.95 | 1.34 | 0.43 | Phosphate Import ATP-Binding Protein 3phosphate |
| cysE | 618 | 10 | 7.18 | 1.34 | 0.43 | Conserved Hypothetical Proteinconserved |
| INV10412140 | 489 | 8 | 5.69 | 1.35 | 0.43 | SAP Domain Proteinsap |
| INV10416640 | 552 | 9 | 6.42 | 1.35 | 0.43 | Conserved Hypothetical Proteinconserved |
| fus | 2082 | 33 | 24.21 | 1.35 | 0.43 | Putative Fructose-6-Phosphate Aldolase 1 Putative |
| INV10407330 | 1123 | 18 | 13.06 | 1.35 | 0.43 | Putative Membrane Protein Putative |
| conjugativetransposonmembraneprotein | 231 | 4 | 2.69 | 1.36 | 0.44 | Competence Protein Coiacompetence |
| pepS | 1242 | 20 | 14.44 | 1.36 | 0.44 | 3-Hydroxy-3-Methylglutaryl-Coenzyme A Reductase3-Hydroxy-3-Methylglutaryl-Coenzyme |
| INV10404040 | 1680 | 27 | 19.53 | 1.36 | 0.45 | Putative Major Facilitator Superfamily Protein Putative |
| DNAintegrationrecombinationinvertionprotein | 165 | 3 | 1.92 | 1.37 | 0.45 | D-Alanyl Carrier Proteind-Alanyl |
| INV10407760 | 165 | 3 | 1.92 | 1.37 | 0.45 | ABC Transporter Permease Protein ABC |
| treR | 729 | 12 | 8.48 | 1.37 | 0.46 | Ribosome Recycling Factorribosome |
| Maturase-relatedprotein | 351 | 6 | 4.08 | 1.38 | 0.46 | IS861, Transposase (Orf1), IS3 Family, Truncatedis861, |
| priA | 2397 | 39 | 27.87 | 1.39 | 0.47 | Putative Pyruvate Dehydrogenase E1 Component, Alpha Subunit Putative |
| INV10413350 | 969 | 16 | 11.27 | 1.39 | 0.47 | Putative Amino Acid ABC Transporter, Extracellular Amino Acid-Binding Protein Putative |
| INV10415290 | 471 | 8 | 5.48 | 1.39 | 0.47 | Putative Membrane Protein Putative |
| scrR | 966 | 16 | 11.23 | 1.39 | 0.48 | Ribulose-Phosphate 3-Epimerase (EC 5.1.3.1)Ribulose-Phosphate |
| INV10415280 | 1089 | 18 | 12.66 | 1.39 | 0.48 | Putative Membrane Protein Putative |
| INV10415320 | 1272 | 21 | 14.79 | 1.39 | 0.48 | Gntr Family Regulatory Proteingntr |
| glpO | 1827 | 30 | 21.24 | 1.39 | 0.48 | Glycogen Synthaseglycogen |
| 948567_979552 | 30985 | 504 | 360.23 | 1.4 | 0.48 |  |
| murE | 1446 | 24 | 16.81 | 1.4 | 0.49 | Lipoprotein Signal Peptidaselipoprotein |
| divIB | 1200 | 20 | 13.95 | 1.4 | 0.49 | Degenerate Transposasedegenerate |
| lctO | 1137 | 19 | 13.22 | 1.41 | 0.49 | Putative Cobalt Transport Protein Putative |
| INV10415330 | 1320 | 22 | 15.35 | 1.41 | 0.49 | ABC Transporter ATP-Binding Protein ABC |
| INV10402610 | 3204 | 53 | 37.25 | 1.41 | 0.5 | Putative Folylpolyglutamate Synthase Putative |
| INV10417360 | 1311 | 22 | 15.24 | 1.42 | 0.5 | Putative Cell Wall-Binding Amidase Putative |
| INV10415820 | 458 | 8 | 5.32 | 1.42 | 0.51 | Putative Atpase Putative |
| INV10403890 | 216 | 4 | 2.51 | 1.42 | 0.51 | Putative Membrane Protein Putative |
| pyrD | 939 | 16 | 10.92 | 1.43 | 0.51 | Phosphoenolpyruvate-Dihydroxyacetone Phosphotransferase (EC 2.7.1.121), Subunit Dham; DHA-Specific IIA Componentphosphoenolpyruvate-Dihydroxyacetone |
| thiE | 633 | 11 | 7.36 | 1.44 | 0.52 | 50S Ribosomal Protein L2450S |
| dapA | 573 | 10 | 6.66 | 1.44 | 0.52 | Cysteinyl-Trna Synthetasecysteinyl-Trna |
| ogt | 513 | 9 | 5.96 | 1.44 | 0.52 | Large-Conductance Mechanosensitive Channellarge-Conductance |
| rpsI | 393 | 7 | 4.57 | 1.44 | 0.52 | Dihydroorotate Dehydrogenase Electron Transfer Subunitdihydroorotate |
| INV10404870 | 213 | 4 | 2.48 | 1.44 | 0.52 | Putative Membrane Protein Putative |
| INV10405230 | 93 | 2 | 1.08 | 1.44 | 0.53 | Putative Uncharacterized Protein Putative |
| gcnA | 1881 | 32 | 21.87 | 1.44 | 0.53 | Fuculose-1-Phosphate Aldolasefuculose-1-Phosphate |
| INV10414360 | 1104 | 19 | 12.84 | 1.45 | 0.53 | Conserved Hypothetical Proteinconserved |
| phageprotein | 330 | 6 | 3.84 | 1.45 | 0.53 | N-Ethylammeline Chlorohydrolasen-Ethylammeline |
| INV10400720 | 924 | 16 | 10.74 | 1.45 | 0.53 | Conserved Hypothetical Proteinconserved |
| INV10407390 | 686 | 12 | 7.98 | 1.45 | 0.53 | Putative Haloacid Dehalogenase-Like Hydrolase Putative |
| 778203_779542 | 1339 | 23 | 15.57 | 1.45 | 0.53 |  |
| INV10405410 | 626 | 11 | 7.28 | 1.45 | 0.54 | Putative Ribomuclease Putative |
| INV10416960 | 741 | 13 | 8.61 | 1.46 | 0.54 | Putative Trna-Binding Protein Putative |
| pyrH | 738 | 13 | 8.58 | 1.46 | 0.55 | Pneumococcal Histidine Triad Protein E (Bvh-3)Pneumococcal |
| Tn5252_relaxase | 1830 | 32 | 21.28 | 1.48 | 0.57 | Putative DNA-Directed RNA Polymerase, Delta Subunit Putative |
| INV10408990 | 261 | 5 | 3.03 | 1.49 | 0.57 | Conserved Hypothetical Proteinconserved |
| INV10418680 | 434 | 8 | 5.05 | 1.49 | 0.57 | Conserved Hypothetical Proteinconserved |
| INV10416980 | 894 | 16 | 10.39 | 1.49 | 0.58 | Putative Exported Protein Putative |
| Macrolide-effluxprotein | 1231 | 22 | 14.31 | 1.5 | 0.59 | IS1380-Spn1, Transposaseis1380-Spn1, |
| mvaS | 1173 | 21 | 13.64 | 1.5 | 0.59 | Putative Mannose-Specific Phosphotransferase System (PTS), IID Component Putative |
| 1882167_1882309 | 142 | 3 | 1.65 | 1.51 | 0.59 |  |
| INV10417370 | 142 | 3 | 1.65 | 1.51 | 0.59 | Putative Drug/Sodium Antiporter Putative |
| INV10415670 | 369 | 7 | 4.29 | 1.51 | 0.6 | Putative Sodium:Dicarboxylate Symporter Family Protein Putative |
| INV10416240 | 878 | 16 | 10.21 | 1.52 | 0.6 | Hypotheical Proteinhypotheical |
| INV10403860 | 365 | 7 | 4.24 | 1.53 | 0.61 | Laci Family Regulatory Proteinlaci |
| purD | 1263 | 23 | 14.68 | 1.53 | 0.61 | Phage Major Tail Proteinphage |
| INV10413830 | 528 | 10 | 6.14 | 1.54 | 0.62 | Cation Efflux Family Proteincation |
| INV10400730 | 1476 | 27 | 17.16 | 1.54 | 0.62 | Putative Deaminase Putative |
| INV10405430 | 360 | 7 | 4.19 | 1.54 | 0.62 |  |
| INV10400750 | 861 | 16 | 10.01 | 1.54 | 0.63 | Putative Phosphoglycerate Mutase Family Protein Putative |
| INV10406920 | 525 | 10 | 6.1 | 1.55 | 0.63 | Putative Helicase Putative |
| INV10408090 | 855 | 16 | 9.94 | 1.55 | 0.64 | Putative Integrase/Recombinase Putative |
| INV10412370 | 1073 | 20 | 12.47 | 1.56 | 0.64 | Putative Membrane Protein Putative |
| epuA | 189 | 4 | 2.2 | 1.56 | 0.64 | DNA Replication Protein Dnacdna |
| dnaI | 897 | 17 | 10.43 | 1.58 | 0.66 | Putative Activated D-Alanine Transport Protein Putative |
| purF | 1443 | 27 | 16.78 | 1.58 | 0.66 | Phage Proteinphage |
| uvrB | 1989 | 37 | 23.12 | 1.58 | 0.66 | Tn5252, Orf23tn5252, |
| INV10417140 | 895 | 17 | 10.41 | 1.58 | 0.66 | Putative Uncharacterized Protein Putative |
| mvaA | 1275 | 24 | 14.82 | 1.58 | 0.66 | Maltose Operon Transcriptional Repressormaltose |
| INV10407930 | 888 | 17 | 10.32 | 1.59 | 0.67 | Putative Membrane Protein Putative |
| aqpZ | 669 | 13 | 7.78 | 1.59 | 0.67 | Aquaporin Zaquaporin |
| INV10414880 | 501 | 10 | 5.82 | 1.61 | 0.69 | Putative Transcription Regulator Putative |
| INV10414440 | 981 | 19 | 11.41 | 1.61 | 0.69 | Conserved Hypothetical Protein (Pseudogene)Conserved |
| INV10412540 | 1246 | 24 | 14.49 | 1.61 | 0.69 | Acetyltransferase (GNAT) Family Proteinacetyltransferase |
| INV10405370 | 498 | 10 | 5.79 | 1.62 | 0.7 | Putative IS1239 Transposase (Pseudogene) Putative |
| INV10402580 | 177 | 4 | 2.06 | 1.64 | 0.71 | Conserved Hypothetical Proteinconserved |
| INV10419330 | 490 | 10 | 5.7 | 1.64 | 0.72 | Putative PTS System, Mannose-Specific IIAB Component Putative |
| INV10416930 | 540 | 11 | 6.28 | 1.65 | 0.72 | Putative Single Stranded DNA-Binding Protein Putative |
| ProteinofunknownfunctionDUF208 | 328 | 7 | 3.81 | 1.66 | 0.73 | Aminopeptidase Pepsaminopeptidase |
| INV10418590 | 532 | 11 | 6.19 | 1.67 | 0.74 | Putative Haloacid Dehalogenase-Like Hydrolase Putative |
| holB | 891 | 18 | 10.36 | 1.67 | 0.74 | 60 Kda Chaperonin60 |
| dnaG | 1761 | 35 | 20.47 | 1.68 | 0.75 | Putative Cell Division Protein Divib/Ftsq Putative |
| lacT2 | 837 | 17 | 9.73 | 1.68 | 0.75 | Putative Amidase Putative |
| hypotheticalphageprotein | 426 | 9 | 4.95 | 1.68 | 0.75 | DNA Gyrase Subunit BDNA |
| lacA | 426 | 9 | 4.95 | 1.68 | 0.75 | ABC Transporter, Permease Protein ABC |
| INV10402900 | 1345 | 27 | 15.64 | 1.68 | 0.75 | Putative Permease Component Of ABC Transporter (Pseudogene) Putative |
| leuB | 1038 | 21 | 12.07 | 1.68 | 0.75 | Putative Protease Putative |
| bgaA | 6702 | 132 | 77.92 | 1.69 | 0.75 | Beta-1,3-Glucosyltransferasebeta-1,3-Glucosyltransferase |
| INV10405990 | 1387 | 28 | 16.13 | 1.69 | 0.76 | Putative Uncharacterized Protein Putative |
| INV10413560 | 1227 | 25 | 14.27 | 1.7 | 0.77 | Putative SNF-Family Helicase Putative |
| 1000755_1001830 | 1075 | 22 | 12.5 | 1.7 | 0.77 |  |
| INV10409390 | 1075 | 22 | 12.5 | 1.7 | 0.77 | Conserved Hypothetical Proteinconserved |
| trpG | 567 | 12 | 6.59 | 1.71 | 0.78 | Putative Fes Assembly Atpase Sufc, ABC Transporter, ATP-Binding Protein Putative |
| murG | 1059 | 22 | 12.31 | 1.73 | 0.79 | LSU Ribosomal Protein L21plsu |
| mraW | 951 | 20 | 11.06 | 1.74 | 0.8 | Lactose Phosphotransferase System Repressor 1lactose |
| adcA | 1247 | 26 | 14.5 | 1.74 | 0.8 | Zinc-Binding Protein Adca Precursorzinc-Binding |
| pyrF | 702 | 15 | 8.16 | 1.75 | 0.8 | Putative Phosphate Transport System Protein Putative |
| FIG01115561-hypotheticalprotein | 995 | 21 | 11.57 | 1.75 | 0.81 | FIG01114502: Hypothetical Proteinfig01114502: |
| rnc | 699 | 15 | 8.13 | 1.75 | 0.81 | Putative Glutamate 5-Kinase Putative |
| INV10406460 | 789 | 17 | 9.17 | 1.77 | 0.82 | Amino-Acid ABC Transporter ATP-Binding Protein (Pseudogene)Amino-Acid |
| INV10405750 | 642 | 14 | 7.46 | 1.77 | 0.83 | Putative Uncharacterized Protein Putative |
| FIG01114899-hypotheticalprotein | 591 | 13 | 6.87 | 1.78 | 0.83 | FIG01114045: Hypothetical Proteinfig01114045: |
| gltX | 1461 | 31 | 16.99 | 1.78 | 0.83 | Glucose-1-Phosphate Adenylyltransferaseglucose-1-Phosphate |
| INV10415430 | 204 | 5 | 2.37 | 1.78 | 0.83 | Putative Ribosomal RNA Small Subunit Methyltransferase Putative |
| Integrase | 1509 | 32 | 17.54 | 1.78 | 0.83 | Hypothetical Phage Proteinhypothetical |
| INV10404390 | 732 | 16 | 8.51 | 1.79 | 0.84 | Hypothetical Proteinhypothetical |
| INV10405220 | 153 | 4 | 1.78 | 1.8 | 0.85 | Conserved Hypothetical Proteinconserved |
| INV10410120 | 153 | 4 | 1.78 | 1.8 | 0.85 | Putative Glycosyltransferase Putative |
| INV10415740 | 153 | 4 | 1.78 | 1.8 | 0.85 | Conserved Hypothetical Proteinconserved |
| INV10407080 | 535 | 12 | 6.22 | 1.8 | 0.85 | ATP-Binding Proteinatp-Binding |
| INV10414900 | 630 | 14 | 7.32 | 1.8 | 0.85 | Putative Membrane Protein Putative |
| INV10407700 | 717 | 16 | 8.34 | 1.82 | 0.86 | Putative Cytolysin (Pseudogene) Putative |
| INV10402910 | 995 | 22 | 11.57 | 1.83 | 0.87 | Probable ATP-Binding Component Of ABC Transporterprobable |
| blpA2 | 2158 | 47 | 25.09 | 1.84 | 0.88 | Putative Surface Anchored Beta-Galactosidase Putative |
| rpmH | 54 | 2 | 0.63 | 1.84 | 0.88 | Phosphoribosylglycinamide Formyltransferasephosphoribosylglycinamide |
| INV10413510 | 147 | 4 | 1.71 | 1.85 | 0.88 | Acetyltransferase (GNAT) Family Proteinacetyltransferase |
| INV10416670 | 426 | 10 | 4.95 | 1.85 | 0.89 | HAM1 Protein Homologham1 |
| INV10404410 | 330 | 8 | 3.84 | 1.86 | 0.89 | Putative Glycosyl Transferase (Pseudogene) Putative |
| INV10410140 | 2451 | 54 | 28.5 | 1.86 | 0.9 | Putative Phage Shock Protein Putative |
| 527268_531467 | 4199 | 92 | 48.82 | 1.87 | 0.9 |  |
| INV10414860 | 741 | 17 | 8.61 | 1.87 | 0.91 | Putative Membrane Protein Putative |
| INV10412110 | 552 | 13 | 6.42 | 1.89 | 0.92 | Putative Phosphohydrolase Putative |
| 527268_531281 | 4013 | 89 | 46.66 | 1.89 | 0.92 |  |
| IS630-Spn1_transposaseOrf2 | 141 | 4 | 1.64 | 1.89 | 0.92 | Putative Trna-Dihydrouridine Synthase Putative |
| 1396666_1397623 | 957 | 22 | 11.13 | 1.9 | 0.92 |  |
| INV10412640 | 957 | 22 | 11.13 | 1.9 | 0.92 | Putative Peptidase Putative |
| INV10403700 | 186 | 5 | 2.16 | 1.9 | 0.93 | Putative Transposase (Pseudogene) Putative |
| engA | 1311 | 30 | 15.24 | 1.91 | 0.93 | Chaperone Protein Dnajchaperone |
| 870453_871260 | 807 | 19 | 9.38 | 1.93 | 0.95 |  |
| INV10408040 | 807 | 19 | 9.38 | 1.93 | 0.95 | Putative Type I RM Modification Enzyme Putative |
| lacD2 | 981 | 23 | 11.41 | 1.93 | 0.95 | Sigma 54 Modulation Protein / S30EA Ribosomal Proteinsigma |
| INV10405660 | 756 | 18 | 8.79 | 1.94 | 0.96 | Putative Esterase Putative |
| glpF1 | 705 | 17 | 8.2 | 1.96 | 0.97 | Putative Epimerase Putative |
| INV10414140 | 264 | 7 | 3.07 | 1.97 | 0.97 | Pyridine Nucleotide-Disulphide Oxidoreductasepyridine |
| INV10405830 | 351 | 9 | 4.08 | 1.97 | 0.98 | Putative Uncharacterized Protein Putative |
| INV10413740 | 955 | 23 | 11.1 | 1.98 | 0.99 | Conserved Hypothetical Proteinconserved |
| INV10413170 | 432 | 11 | 5.02 | 1.99 | 1 | Conserved Hypothetical Proteinconserved |
| lacB1 | 516 | 13 | 6 | 2 | 1 | Conserved Hypothetical Proteinconserved |
| INV10414530 | 642 | 16 | 7.46 | 2.01 | 1.01 | Conserved Hypothetical Proteinconserved |
| DNARNAHELICASE(DEADDEAHBOXFAMILY) | 2680 | 64 | 31.16 | 2.02 | 1.02 | Replicative DNA Helicasereplicative |
| INV10410760 | 1317 | 32 | 15.31 | 2.02 | 1.02 | Putative Hydrolase (Pseudogene) Putative |
| pheT | 2406 | 58 | 27.97 | 2.04 | 1.03 | Sialidase A (Neuraminidase A)Sialidase |
| INV10410720 | 795 | 20 | 9.24 | 2.05 | 1.04 | Putative Pullulanase Putative |
| INV10408050 | 249 | 7 | 2.89 | 2.05 | 1.04 | Putative Uncharacterized Protein Putative |
| INV10416680 | 249 | 7 | 2.89 | 2.05 | 1.04 | Putative Exported Protein Putative |
| INV10404780 | 81 | 3 | 0.94 | 2.06 | 1.04 | Putative Cation Transport Protein Putative |
| Cellwall-associatedmureinhydrolaseLytA | 957 | 24 | 11.13 | 2.06 | 1.04 | Cell Division Initiation Protein Divivacell |
| INV10414300 | 453 | 12 | 5.27 | 2.07 | 1.05 | Phna Proteinphna |
| INV10415210 | 534 | 14 | 6.21 | 2.08 | 1.06 | Conserved Hypothetical Proteinconserved |
| INV10403910 | 1566 | 39 | 18.21 | 2.08 | 1.06 |  |
| INV10402590 | 2040 | 51 | 23.72 | 2.1 | 1.07 | Putative Holliday Junction Resolvase Putative |
| chlorohydrolase | 159 | 5 | 1.85 | 2.11 | 1.07 | Cell Division Protein Gpsb, Coordinates The Switch Between Cylindrical And Septal Cell Wall Synthesis By Re-Localization Of PBP1Cell |
| INV10412530 | 606 | 16 | 7.05 | 2.11 | 1.08 | Putative Choline Binding Protein (Pseudogene) Putative |
| INV10404120 | 564 | 15 | 6.56 | 2.12 | 1.08 | Conserved Hypothetical Proteinconserved |
| CelldivisioninitiationproteinDivIVA | 117 | 4 | 1.36 | 2.12 | 1.08 | Competence-Induced Protein Ccs1 (Pseudogene)Competence-Induced |
| INV10412650 | 563 | 15 | 6.55 | 2.12 | 1.08 | Putative Peptidase Putative |
| INV10408850 | 603 | 16 | 7.01 | 2.12 | 1.09 | Transposase (Pseudogene)Transposase |
| DNA-cytosinemethyltransferase(EC2 | 1089 | 28 | 12.66 | 2.12 | 1.09 | Putative Purine Nucleoside Phosphorylase Putative |
| INV10406130 | 1369 | 35 | 15.92 | 2.13 | 1.09 | Conserved Hypothetical Proteinconserved |
| dnaE | 3063 | 77 | 35.61 | 2.13 | 1.09 | DNA Polymerase IVDNA |
| IS861_transposase(orf2)_IS3family_truncated | 274 | 8 | 3.19 | 2.15 | 1.1 | Putative Two-Component System, Sensor Histidine Kinase Putative |
| INV10414540 | 792 | 21 | 9.21 | 2.16 | 1.11 | Putative Transcriptional Regulator Protein Putative |
| thrB | 870 | 23 | 10.11 | 2.16 | 1.11 | 50S Ribosomal Protein L3050S |
| cdd | 390 | 11 | 4.53 | 2.17 | 1.12 | Heme Exporter Protein A (Cytochrome C-Type Biogenesis Protein)Heme |
| INV10417020 | 942 | 25 | 10.95 | 2.18 | 1.12 | Putative Membrane Protein Putative |
| INV10407720 | 901 | 24 | 10.48 | 2.18 | 1.12 | Putative Membrane Protein (Pseudogene) Putative |
| glnQ2 | 741 | 20 | 8.61 | 2.18 | 1.13 | N-Acetyl-Beta-D-Glucosaminidasen-Acetyl-Beta-D-Glucosaminidase |
| INV10408900 | 975 | 26 | 11.34 | 2.19 | 1.13 | Carbon-Nitrogen Hydrolase Family Proteincarbon-Nitrogen |
| murD | 1351 | 36 | 15.71 | 2.21 | 1.15 | Laminin Binding Proteinlaminin |
| 1624627_1625237 | 610 | 17 | 7.09 | 2.22 | 1.15 |  |
| INV10414740 | 610 | 17 | 7.09 | 2.22 | 1.15 | Putative Uncharacterized Protein Putative |
| INV10401180 | 1227 | 33 | 14.27 | 2.23 | 1.15 | Extracellular Solute-Binding Proteinextracellular |
| INV10410900 | 879 | 24 | 10.22 | 2.23 | 1.16 | Haloacid Dehalogenase-Like Hydrolasehaloacid |
| INV10416630 | 261 | 8 | 3.03 | 2.23 | 1.16 | Siderophore Uptake Periplasmic Binding Proteinsiderophore |
| INV10418530 | 1680 | 45 | 19.53 | 2.24 | 1.16 | Putative Membrane Protein Putative |
| INV10417030 | 1257 | 34 | 14.61 | 2.24 | 1.16 | Putative DNA-Binding Protein Putative |
| INV10415750 | 297 | 9 | 3.45 | 2.25 | 1.17 | Conserved Hypothetical Proteinconserved |
| dinB | 1063 | 29 | 12.36 | 2.25 | 1.17 | Degenerate Transposasedegenerate |
| blpB | 1362 | 37 | 15.83 | 2.26 | 1.17 | Bira Bifunctional Protein [Includes: Biotin Operon Repressor; Biotin--[Acetyl-Coa-Carboxylase] Synthetase]Bira |
| INV10407780 | 759 | 22 | 8.82 | 2.34 | 1.23 | Putative Membrane Protein Putative |
| INV10416790 | 171 | 6 | 1.99 | 2.34 | 1.23 | Putative Oligopeptide Transporter Permease Protein Putative |
| 1253454_1255712 | 2258 | 63 | 26.25 | 2.35 | 1.23 |  |
| Formamidopyrimidine-DNAglycosylase(EC3 | 825 | 24 | 9.59 | 2.36 | 1.24 | FIG01118323: Hypothetical Proteinfig01118323: |
| INV10405910 | 381 | 12 | 4.43 | 2.39 | 1.26 | Putative Sugar Phosphotransferase System (PTS), IIA Component Putative |
| scrB | 1455 | 42 | 16.92 | 2.4 | 1.26 | Riboflavin Synthase Beta Chain (6,7-Dimethyl-8-Ribityllumazine Synthase)Riboflavin |
| mscL | 378 | 12 | 4.39 | 2.41 | 1.27 | Large Subunit Ribosomal RNA; Lsurna; LSU Rrnalarge |
| leuS | 2502 | 72 | 29.09 | 2.43 | 1.28 | Putative Protease Putative |
| TypeIrestriction-modificationsystem_DNA-methyltransferasesubunitM(EC2 | 1464 | 43 | 17.02 | 2.44 | 1.29 | Putative Phosphomethylpyrimidine Kinase Putative |
| INV10406950 | 405 | 13 | 4.71 | 2.45 | 1.29 | Putative DNA Polymerase III Delta Subunit Putative |
| INV10402770 | 543 | 17 | 6.31 | 2.46 | 1.3 | Phosphoglycerate Mutase Family Proteinphosphoglycerate |
| sufB | 1413 | 42 | 16.43 | 2.47 | 1.3 | 50S Ribosomal Protein L1550S |
| INV10418060 | 540 | 17 | 6.28 | 2.47 | 1.31 | Sugar Phosphotransferase System (PTS), IIA Componentsugar |
| INV10407710 | 816 | 25 | 9.49 | 2.48 | 1.31 | Putative DNA Topology Modulation Protein Putative |
| INV10407890 | 810 | 25 | 9.42 | 2.5 | 1.32 | Putative Membrane Protein Putative |
| Diacylglycerolkinase(EC2 | 396 | 13 | 4.6 | 2.5 | 1.32 | Degenerate Transposasedegenerate |
| FIG086557-Conjugationrelatedprotein | 222 | 8 | 2.58 | 2.51 | 1.33 | FIG01116802: Hypothetical Proteinfig01116802: |
| ImpBMucBSamBfamilyprotein | 1416 | 43 | 16.46 | 2.52 | 1.33 | Holliday Junction DNA Helicase Ruvbholliday |
| INV10414850 | 1098 | 34 | 12.77 | 2.54 | 1.35 | Conserved Hypothetical Proteinconserved |
| INV10408810 | 183 | 7 | 2.13 | 2.56 | 1.35 | Putative Membrane Protein (Pseudogene) Putative |
| INV10405420 | 249 | 9 | 2.89 | 2.57 | 1.36 | Leucine-Rich Proteinleucine-Rich |
| INV10412190 | 1287 | 40 | 14.96 | 2.57 | 1.36 | Conserved Hypothetical Proteinconserved |
| gor | 1347 | 42 | 15.66 | 2.58 | 1.37 | Glycerol Uptake Facilitator Protein 1glycerol |
| INV10405920 | 147 | 6 | 1.71 | 2.58 | 1.37 | Sugar Phosphotransferase System (PTS), IIB Componentsugar |
| ileS | 2793 | 86 | 32.47 | 2.6 | 1.38 | Putative Oxygen-Independent Coproporphyrinogen III Oxidase Putative |
| INV10407730 | 2130 | 66 | 24.76 | 2.6 | 1.38 | Putative Methyltransferase Putative |
| clpX | 1233 | 39 | 14.33 | 2.61 | 1.38 | Putative ATP-Dependent Protease ATP-Binding Subunit Clpl Putative |
| cbpE | 1884 | 59 | 21.9 | 2.62 | 1.39 | Choline-Binding Surface Protein Acholine-Binding |
| INV10400610 | 765 | 25 | 8.89 | 2.63 | 1.39 | Intramembrane Protease Rasp/Yluc, Implicated In Cell Division Based On Ftsl Cleavageintramembrane |
| INV10418510 | 1281 | 41 | 14.89 | 2.64 | 1.4 | Putative Membrane Protein Putative |
| upp | 630 | 21 | 7.32 | 2.64 | 1.4 | Tmrna-Binding Protein Smpbtmrna-Binding |
| lacG2 | 1407 | 45 | 16.36 | 2.65 | 1.41 | Putative Transposase (Pseudogene) Putative |
| FerrousirontransportperoxidaseEfeB | 1004 | 33 | 11.67 | 2.68 | 1.42 | Exodeoxyribonucleaseexodeoxyribonuclease |
| FIG01118323-hypotheticalprotein | 198 | 8 | 2.3 | 2.73 | 1.45 | FIG01116379: Hypothetical Proteinfig01116379: |
| 835603_835987 | 384 | 14 | 4.46 | 2.75 | 1.46 |  |
| INV10407680 | 384 | 14 | 4.46 | 2.75 | 1.46 | Carbohydrate Kinasecarbohydrate |
| wze | 666 | 23 | 7.74 | 2.75 | 1.46 | Putative Thiol Peroxidase Putative |
| INV10402780 | 195 | 8 | 2.27 | 2.75 | 1.46 | Putative Inner Membrane Component Of ABC Transporter Putative |
| IS1381_transposaseOrfA | 132 | 6 | 1.53 | 2.76 | 1.47 | Hypothetical Proteinhypothetical |
| INV10406110 | 286 | 11 | 3.33 | 2.77 | 1.47 | Sensor Histidine Kinasesensor |
| tag | 564 | 20 | 6.56 | 2.78 | 1.47 | 50S Ribosomal Protein L1850S |
| potA | 966 | 33 | 11.23 | 2.78 | 1.48 | Putative Chorismate Binding Enzyme Putative |
| INV10414890 | 594 | 21 | 6.91 | 2.78 | 1.48 | ROK Family Proteinrok |
| INV10401020 | 684 | 24 | 7.95 | 2.79 | 1.48 | Conserved Hypothetical Proteinconserved |
| tmRNA-bindingproteinSmpB | 468 | 17 | 5.44 | 2.79 | 1.48 | DNA-Directed RNA Polymerase Alpha Chaindna-Directed |
| 1799135_1799535 | 400 | 15 | 4.65 | 2.83 | 1.5 |  |
| blpS | 339 | 13 | 3.94 | 2.83 | 1.5 | Bacteriocin Blpobacteriocin |
| INV10415250 | 1797 | 62 | 20.89 | 2.88 | 1.53 | Putative Membrane Protein Putative |
| INV10408840 | 840 | 30 | 9.77 | 2.88 | 1.53 | Putative IS1239 Transposase (Pseudogene) Putative |
| INV10404860 | 445 | 17 | 5.17 | 2.92 | 1.54 | Spou Rrna Methylase Family Proteinspou |
| comEA | 651 | 24 | 7.57 | 2.92 | 1.54 | GTP-Sensing Transcriptional Pleiotropic Repressorgtp-Sensing |
| asd | 1077 | 39 | 12.52 | 2.96 | 1.56 | Putative Phospho-2-Dehydro-3-Deoxyheptonate Aldolase 1 Putative |
| thiN | 663 | 25 | 7.71 | 2.99 | 1.58 | 50S Ribosomal Protein L2950S |
| 980372_980573 | 201 | 9 | 2.34 | 3 | 1.58 |  |
| INV10409200 | 201 | 9 | 2.34 | 3 | 1.58 | Putative Uncharacterized Protein Putative |
|  |  | 2 | 0 | 3 | 1.58 | Transposasetransposase |
| INV10413420 | 171 | 8 | 1.99 | 3.01 | 1.59 | Conserved Hypothetical Proteinconserved |
| fucI | 1767 | 64 | 20.54 | 3.02 | 1.59 | Putative Geranyltranstransferase Putative |
| FIG01114010-hypotheticalprotein | 141 | 7 | 1.64 | 3.03 | 1.6 | Fructose-Bisphosphate Aldolasefructose-Bisphosphate |
| INV10402500 | 813 | 31 | 9.45 | 3.06 | 1.61 | Regulatory Protein Spxregulatory |
| INV10408920 | 279 | 12 | 3.24 | 3.06 | 1.62 | Putative Membrane Protein Putative |
| INV10412180 | 670 | 26 | 7.79 | 3.07 | 1.62 | Putative Flavodoxin Putative |
| dnaB | 1171 | 44 | 13.61 | 3.08 | 1.62 | Glucan 1,6-Alpha-Glucosidaseglucan |
| lacE2 | 1692 | 63 | 19.67 | 3.1 | 1.63 | Conserved Hypothetical Proteinconserved |
| proC | 798 | 31 | 9.28 | 3.11 | 1.64 | Aminopeptidase Caminopeptidase |
| INV10401030 | 438 | 18 | 5.09 | 3.12 | 1.64 | Conserved Hypothetical Proteinconserved |
| FIG01114768-hypotheticalprotein | 300 | 13 | 3.49 | 3.12 | 1.64 | FIG01114020: Hypothetical Proteinfig01114020: |
| INV10412150 | 240 | 11 | 2.79 | 3.17 | 1.66 | Crcb-Like Proteincrcb-Like |
| INV10405700 | 726 | 29 | 8.44 | 3.18 | 1.67 | Nitroreductase Family Proteinnitroreductase |
| speE | 861 | 34 | 10.01 | 3.18 | 1.67 | 50S Ribosomal Protein L250S |
| INV10418610 | 1152 | 45 | 13.39 | 3.2 | 1.68 | Putative Threonine Synthase Putative |
| 541745_542197 | 452 | 19 | 5.25 | 3.2 | 1.68 |  |
| 308521_308755 | 234 | 11 | 2.72 | 3.23 | 1.69 |  |
| INV10402620 | 234 | 11 | 2.72 | 3.23 | 1.69 | Putative Lipoprotein Putative |
| INV10415560 | 660 | 27 | 7.67 | 3.23 | 1.69 | GTP-Binding Proteingtp-Binding |
| 1362675_1364823 | 2148 | 83 | 24.97 | 3.23 | 1.69 |  |
| INV10409100 | 1740 | 68 | 20.23 | 3.25 | 1.7 | Putative Alanine Dehydrogenase 1 (Pseudogene) Putative |
| proA | 1263 | 50 | 14.68 | 3.25 | 1.7 | Putative Pyruvate Dehydrogenase E1 Component, Beta Subunit Putative |
| INV10417790 | 1179 | 47 | 13.71 | 3.26 | 1.71 | RNA Pseudouridylate Synthaserna |
| TranscriptionalregulatorOrfX | 492 | 21 | 5.72 | 3.27 | 1.71 | 30S Ribosomal Protein S1330S |
| coaD | 489 | 21 | 5.69 | 3.29 | 1.72 | Pantothenate Kinasepantothenate |
| potC | 774 | 32 | 9 | 3.3 | 1.72 | Topoisomerase IV Subunit Atopoisomerase |
| trpC | 768 | 32 | 8.93 | 3.32 | 1.73 | Single Strand Binding Protein (SSB)Single |
| 584299_586278 | 1979 | 79 | 23.01 | 3.33 | 1.74 |  |
| INV10407660 | 530 | 23 | 6.16 | 3.35 | 1.75 | Conserved Hypothetical Proteinconserved |
| INV10412280 | 195 | 10 | 2.27 | 3.37 | 1.75 | Putative Licd-Family Phosphotransferase Putative |
| INV10411470 | 450 | 20 | 5.23 | 3.37 | 1.75 | Putative Glutamine ABC Transporter, Glutamine-Binding Protein/Permease Protein Putative |
| INV10414630 | 768 | 33 | 8.93 | 3.42 | 1.78 | D-Tyrosyl-Trna(Tyr) Deacylased-Tyrosyl-Trna(Tyr) |
| INV10414950 | 561 | 25 | 6.52 | 3.46 | 1.79 | Extracellular Solute-Binding Lipoproteinextracellular |
| mraY | 981 | 42 | 11.41 | 3.47 | 1.79 | Transcription Antiterminator Lact 2transcription |
| folC | 1323 | 56 | 15.38 | 3.48 | 1.8 | FIG01117889: Hypothetical Proteinfig01117889: |
| INV10403920 | 1890 | 79 | 21.97 | 3.48 | 1.8 | Putative DNA-Binding Protein Putative |
| INV10413570 | 1317 | 56 | 15.31 | 3.49 | 1.81 | Putative Aminotransferase Putative |
| INV10405360 | 6436 | 266 | 74.83 | 3.52 | 1.82 | Putative Integral Membrane Protein (Possible Nuclease Activity) Putative |
| INV10414960 | 157 | 9 | 1.83 | 3.54 | 1.82 | Sugar Phosphotransferase System (PTS), IIBC Componentsugar |
| INV10408150 | 570 | 26 | 6.63 | 3.54 | 1.82 | Putative Lipoprotein Putative |
| FIG00627334-hypotheticalprotein | 2448 | 104 | 28.46 | 3.56 | 1.83 | 3-Oxoacyl-[Acyl-Carrier-Protein] Synthase II3-Oxoacyl-[Acyl-Carrier-Protein] |
| FIG01114970-hypotheticalprotein | 243 | 13 | 2.83 | 3.66 | 1.87 | FIG01114146: Hypothetical Proteinfig01114146: |
| INV10418520 | 2087 | 92 | 24.26 | 3.68 | 1.88 | Putative Pyrrolidone-Carboxylate Peptidase Putative |
| Antirestrictionprotein | 498 | 24 | 5.79 | 3.68 | 1.88 | Antirestriction Proteinantirestriction |
| INV10413270 | 1872 | 83 | 21.76 | 3.69 | 1.88 | Putative Transposase (Pseudogene) Putative |
| INV10405190 | 607 | 29 | 7.06 | 3.72 | 1.9 | Putative Uncharacterized Protein Putative |
| INV10409110 | 7665 | 336 | 89.11 | 3.74 | 1.9 | Putative Acetyltransferase (GNAT) Family Protein Putative |
| INV10418830 | 1025 | 48 | 11.92 | 3.79 | 1.92 | Rhomboid Family Membrane Proteinrhomboid |
| INV10415490 | 318 | 17 | 3.7 | 3.83 | 1.94 | Conserved Hypothetical Proteinconserved |
| INV10407750 | 273 | 15 | 3.17 | 3.83 | 1.94 | ABC Transporter ATP-Binding Protein ABC |
| FIG01116987-hypotheticalprotein | 360 | 19 | 4.19 | 3.86 | 1.95 | FIG01115706: Hypothetical Proteinfig01115706: |
| Oxygen-insensitiveNAD(P)Hnitroreductase(EC1 | 531 | 27 | 6.17 | 3.9 | 1.97 | Metal Cation ABC Transporter Membrane Proteinmetal |
| INV10408190 | 1186 | 57 | 13.79 | 3.92 | 1.97 | Putative Uncharacterized Protein Putative |
| INV10408110 | 882 | 44 | 10.25 | 4 | 2 | Type I Restriction Modification System Proteintype |
| FIG01114502-hypotheticalprotein | 450 | 24 | 5.23 | 4.01 | 2 | FIG00628088: Hypothetical Proteinfig00628088: |
| FIG01116966-hypotheticalprotein | 228 | 14 | 2.65 | 4.11 | 2.04 | FIG01115561: Hypothetical Proteinfig01115561: |
| glyA | 1257 | 64 | 14.61 | 4.16 | 2.06 | Glucokinaseglucokinase |
| INV10407740 | 450 | 25 | 5.23 | 4.17 | 2.06 | Putative Lipoprotein Putative |
| INV10405170 | 798 | 42 | 9.28 | 4.18 | 2.06 | Putative Uncharacterized Protein Putative |
| comEC | 2241 | 113 | 26.05 | 4.21 | 2.08 | Competence-Stimulating Peptide Type 2 Precursor (Csp-2)Competence-Stimulating |
| polC | 4392 | 219 | 51.06 | 4.23 | 2.08 | Oxidoreductase, Gfo/Idh/Moca Familyoxidoreductase, |
| 1052773_1117795 | 65022 | 3198 | 755.95 | 4.23 | 2.08 |  |
| BacteriocinimmunityproteinBlpL | 372 | 22 | 4.32 | 4.32 | 2.11 | Bacteriocin-Like Peptide N Blpnbacteriocin-Like |
| INV10413760 | 1104 | 59 | 12.84 | 4.34 | 2.12 | Putative Membrane Protein Putative |
| purE | 489 | 28 | 5.69 | 4.34 | 2.12 | Phage Portal Proteinphage |
| deoC | 663 | 37 | 7.71 | 4.36 | 2.13 | Polypeptide Deformylasepolypeptide |
| INV10407690 | 282 | 18 | 3.28 | 4.44 | 2.15 | Conserved Hypothetical Proteinconserved |
| Transposaseandinactivatedderivative | 417 | 25 | 4.85 | 4.45 | 2.15 | 30S Ribosomal Protein S1930S |
| INV10414280 | 645 | 37 | 7.5 | 4.47 | 2.16 | Major Facilitator Superfamily Proteinmajor |
| INV10414970 | 962 | 54 | 11.18 | 4.51 | 2.17 | Putative N-Acetylmannosamine-6-Phosphate 2-Epimerase Putative |
| FIG01114872-hypotheticalprotein | 489 | 30 | 5.69 | 4.64 | 2.21 | FIG01114020: Hypothetical Proteinfig01114020: |
| calcium-bindingprotein_ putative | 411 | 26 | 4.78 | 4.67 | 2.22 | Putative Branched-Chain Amino Acid Transport System Carrier Protein Putative |
| lacF | 318 | 21 | 3.7 | 4.68 | 2.23 | Putative Transposase Family Protein Putative |
| smc | 3540 | 199 | 41.16 | 4.74 | 2.25 | Ribonuclease Hiiribonuclease |
| tnp | 1031 | 61 | 11.99 | 4.77 | 2.25 | 30S Ribosomal Protein S330S |
| engC | 1310 | 78 | 15.23 | 4.87 | 2.28 | DNA Polymerase III, Beta Chaindna |
| INV10405730 | 405 | 27 | 4.71 | 4.9 | 2.29 | Putative Uncharacterized Protein Putative |
| HitFamilyProtein | 442 | 30 | 5.14 | 5.05 | 2.34 | Glycerol-3-Phosphate Dehydrogenase [NAD(P)+]Glycerol-3-Phosphate |
| INV10402450 | 708 | 46 | 8.23 | 5.09 | 2.35 | Putative Metallopeptidase Putative |
| INV10411440 | 588 | 39 | 6.84 | 5.1 | 2.35 | CAAX Amino Terminal Protease Family Proteincaax |
| blpR | 738 | 48 | 8.58 | 5.11 | 2.35 | Sensor Histidine Kinase Blphsensor |
| Pleiotropicregulatorofexopolysaccharidesynthesis_competenceandbiofilmformationFtr_XREfamily | 684 | 46 | 7.95 | 5.25 | 2.39 | Putative Putative N Utilization Substance Protein A Putative |
| conserveddomainprotein | 2815 | 178 | 32.73 | 5.31 | 2.41 | Putative Competence Protein Putative |
| INV10408010 | 870 | 58 | 10.11 | 5.31 | 2.41 |  |
| membraneprotein_ putative | 2117 | 135 | 24.61 | 5.31 | 2.41 | IS861, Transposase (Orf2), IS3 Family, Truncatedis861, |
| pmi | 945 | 63 | 10.99 | 5.34 | 2.42 | Oligoendopeptidase F (Ec 3.4.24.-)Oligoendopeptidase |
| ksgA | 873 | 59 | 10.15 | 5.38 | 2.43 | ABC Transporter, Substrate-Binding Protein ABC |
| purK | 1092 | 73 | 12.7 | 5.4 | 2.43 | Phage Replication Initiation Proteinphage |
| lipoprotein_NLPP60family | 1002 | 68 | 11.65 | 5.45 | 2.45 | Putative Membrane Protein Putative |
| FIG01116389-hypotheticalprotein | 1882 | 126 | 21.88 | 5.55 | 2.47 | FIG01115415: Hypothetical Proteinfig01115415: |
| FIG00628965-hypotheticalprotein | 936 | 66 | 10.88 | 5.64 | 2.5 | Enoyl-ACP Reductaseenoyl-ACP |
| RegulatoryproteinrecX | 777 | 57 | 9.03 | 5.78 | 2.53 | DNA Polymerase III Polc-Typedna |
| INV10409250 | 759 | 56 | 8.82 | 5.8 | 2.54 | Putative Redoxin Family Protein Putative |
| INV10408760 | 432 | 34 | 5.02 | 5.81 | 2.54 | ABC Transporter, ATP-Binding Protein ABC |
| INV10413780 | 678 | 51 | 7.88 | 5.85 | 2.55 | Putative Membrane Protein Putative |
| INV10416510 | 675 | 51 | 7.85 | 5.88 | 2.55 | Tetr Family Regulatory Proteintetr |
| potB | 807 | 61 | 9.38 | 5.97 | 2.58 | Putative Chromosome Partitioning Protein Parb Putative |
| INV10414290 | 279 | 25 | 3.24 | 6.13 | 2.62 | Putative Membrane Protein Putative |
| FIG01114468-hypotheticalprotein | 642 | 51 | 7.46 | 6.14 | 2.62 | FIG00627334: Hypothetical Proteinfig00627334: |
| INV10409970 | 852 | 67 | 9.91 | 6.24 | 2.64 |  |
| INV10405740 | 2022 | 152 | 23.51 | 6.24 | 2.64 | M42 Glutamyl Aminopeptidasem42 |
| INV10415230 | 793 | 63 | 9.22 | 6.26 | 2.65 | Acetyl Xylan Esterase (AXE1) Family Proteinacetyl |
| undecaprenylpyrophosphatephosphatase | 130 | 15 | 1.51 | 6.37 | 2.67 | Thymidylate Synthasethymidylate |
| pbp2x | 2253 | 176 | 26.19 | 6.51 | 2.7 | Putative UDP-N-Acetylglucosamine-N-Acetylmuramyl-(Pentapeptide)Pyr Ophosphoryl-Undecaprenol N-Acetylglucosamine Transferase Putative |
| FIG01118149-hypotheticalprotein | 297 | 28 | 3.45 | 6.51 | 2.7 | FIG01116303: Hypothetical Proteinfig01116303: |
| INV10407280 | 1143 | 93 | 13.29 | 6.58 | 2.72 | Putative Short Chain Dehydrogenase Putative |
| INV10409990 | 1551 | 126 | 18.03 | 6.67 | 2.74 | Putative Transposase (Pseudogene) Putative |
| INV10407270 | 429 | 40 | 4.99 | 6.85 | 2.77 | Conserved Hypothetical Proteinconserved |
| INV10405250 | 1039 | 89 | 12.08 | 6.88 | 2.78 | Putative Acetyltransferase Putative |
| Phageholin | 336 | 33 | 3.91 | 6.93 | 2.79 | N-Ethylammeline Chlorohydrolasen-Ethylammeline |
| INV10415710 | 1068 | 92 | 12.42 | 6.93 | 2.79 | Conserved Hypothetical Proteinconserved |
| INV10414980 | 954 | 83 | 11.09 | 6.95 | 2.8 | Putative Oxidoreductase Putative |
| D-alanyl-D-alaninecarboxypeptidase(EC3 | 717 | 65 | 8.34 | 7.07 | 2.82 | Putative Cysteine Synthase Putative |
| INV10416520 | 447 | 43 | 5.2 | 7.1 | 2.83 | Nicotinamide Mononucleotide Transporternicotinamide |
| blpH | 1341 | 117 | 15.59 | 7.11 | 2.83 | ABC Transporter Blpbabc |
| sensorhistidinekinase | 1098 | 97 | 12.77 | 7.12 | 2.83 | Dtdp-4-Keto-6-Deoxy-D-Glucose 3,5-Epimerase Rmlcdtdp-4-Keto-6-Deoxy-D-Glucose |
| SNF2familyprotein | 6232 | 528 | 72.45 | 7.2 | 2.85 | Ribonuclease Zribonuclease |
| INV10408180 | 588 | 59 | 6.84 | 7.66 | 2.94 | Putative IS1381 Transposase (Pseudogene) Putative |
| INV10407410 | 603 | 61 | 7.01 | 7.74 | 2.95 | Putative IS640-Spn1 Transposase (Pseudogene) Putative |
| blpC | 156 | 21 | 1.81 | 7.82 | 2.97 | Putative Bacteriocin Transporter C39 Protease Domain Blpa2 Putative |
| rnh | 780 | 78 | 9.07 | 7.85 | 2.97 | Probably Aromatic Ring Hydroxylating Enzyme, Evidenced By Cognitor; Paad-Like Protein (DUF59) Involved In Fe-S Cluster Assemblyprobably |
| INV10414510 | 154 | 21 | 1.79 | 7.88 | 2.98 | Sensor Histidine Kinase Proteinsensor |
| folP | 981 | 97 | 11.41 | 7.9 | 2.98 | FIG01118149: Hypothetical Proteinfig01118149: |
| thiM | 783 | 82 | 9.1 | 8.22 | 3.04 | 50S Ribosomal Protein L2850S |
| dyr | 507 | 57 | 5.89 | 8.41 | 3.07 | DNA Polymerase III Subunit Gamma/Taudna |
| FIG01115786-hypotheticalprotein | 273 | 36 | 3.17 | 8.86 | 3.15 | FIG01114689: Hypothetical Proteinfig01114689: |
| FIG01116415-hypotheticalprotein | 1086 | 130 | 12.63 | 9.61 | 3.26 | FIG01115489: Hypothetical Proteinfig01115489: |
| tgt | 1143 | 144 | 13.29 | 10.15 | 3.34 | 50S Ribosomal Protein L2250S |
| INV10401150 | 864 | 114 | 10.04 | 10.41 | 3.38 | Putative IS630-Spn1 Transposase (Pseudogene) Putative |
| trpF | 630 | 88 | 7.32 | 10.69 | 3.42 | Fes Assembly Protein Sufbfes |
| Tn5252_Orf9protein | 228 | 58 | 2.65 | 16.16 | 4.01 | RNA Polymerase Sigma Factor Rpodrna |
| truA | 750 | 178 | 8.72 | 18.42 | 4.2 | Putative Thymidine Kinase Putative |
| tRNA-Arg-ACG | 74 | 44 | 0.86 | 24.19 | 4.6 | S-Adenosylmethionine-Dependent Methyltransferases-Adenosylmethionine-Dependent |
| Tn916_hypotheticalprotein | 423 | 201 | 4.92 | 34.13 | 5.09 | DNA-Directed RNA Polymerase Omega Chaindna-Directed |
| sodA | 606 | 361 | 7.05 | 44.99 | 5.49 | Ribose 5-Phosphate Isomerase Aribose |
|  |  |  |  |  |  |  |
