# Supplementary material for: Understanding pneumococcal serotype 1 biology through population genomic analysis
Source: BMC Infect Dis. 2016 Nov 8;16:649. doi: 10.1186/s12879-016-1987-z (PMC5100261; doi:10.1186/s12879-016-1987-z)
Supplement: Additional file 11: — Distribution of genes associated with virulence and colonisation in the ST217 in serotype 1 isolates. Presence of the genes was screened in all the isolates using BLAST as described in the methods section. (PDF 1702 kb) [file 12879_2016_1987_MOESM11_ESM.pdf]

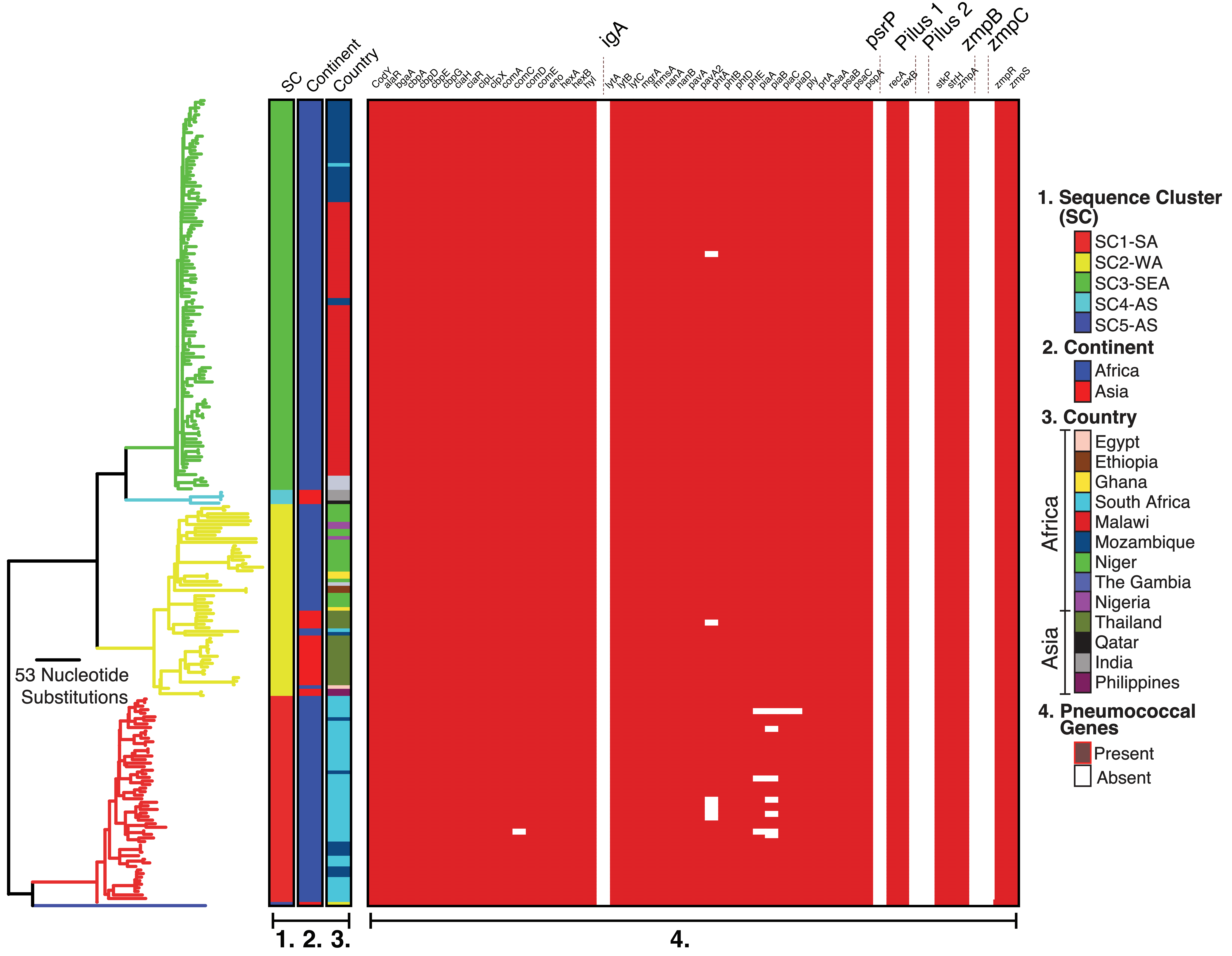

SC
Continent
Country
CodY
alaR
bgaA
cbpA
cbpD
cbpE
cbpG
ciaH
ciaR
clpL
clpX
comA
comC
comD
comE
eno
hexA
hexB
hyl
igA
lytA
lytB
lytC
mgrA
mmsA
nanA
nanB
pavA
pavA2
phtA
phtB
phtD
phtE
piaA
piaB
piaC
piaD
ply
prtA
psaA
psaB
psaC
pspA
psrP
recA
rexB
Pilus 1
Pilus 2
stkP
strH
zmpA
zmpB
zmpC
zmpR
zmpS
53 Nucleotide Substitutions
1.
2.
3.
4.
1. Sequence Cluster (SC)
SC1-SA
SC2-WA
SC3-SEA
SC4-AS
SC5-AS
2. Continent
Africa
Asia
3. Country
Africa
Asia
Egypt
Ethiopia
Ghana
South Africa
Malawi
Mozambique
Niger
The Gambia
Nigeria
Thailand
Qatar
India
Philippines
4. Pneumococcal Genes
Present
Absent
