# Supplementary material for: Understanding pneumococcal serotype 1 biology through population genomic analysis
Source: BMC Infect Dis. 2016 Nov 8;16:649. doi: 10.1186/s12879-016-1987-z (PMC5100261; doi:10.1186/s12879-016-1987-z)
Supplement: Additional file 2: — Clade specific phylogenies showing country of origin of the ST217 isolates. Maximum likelihood phylogenetic trees for clade (a) SC1-SA, (b) SC2-WA and (c) SC3-SEA. (PDF 292 kb) [file 12879_2016_1987_MOESM2_ESM.pdf]

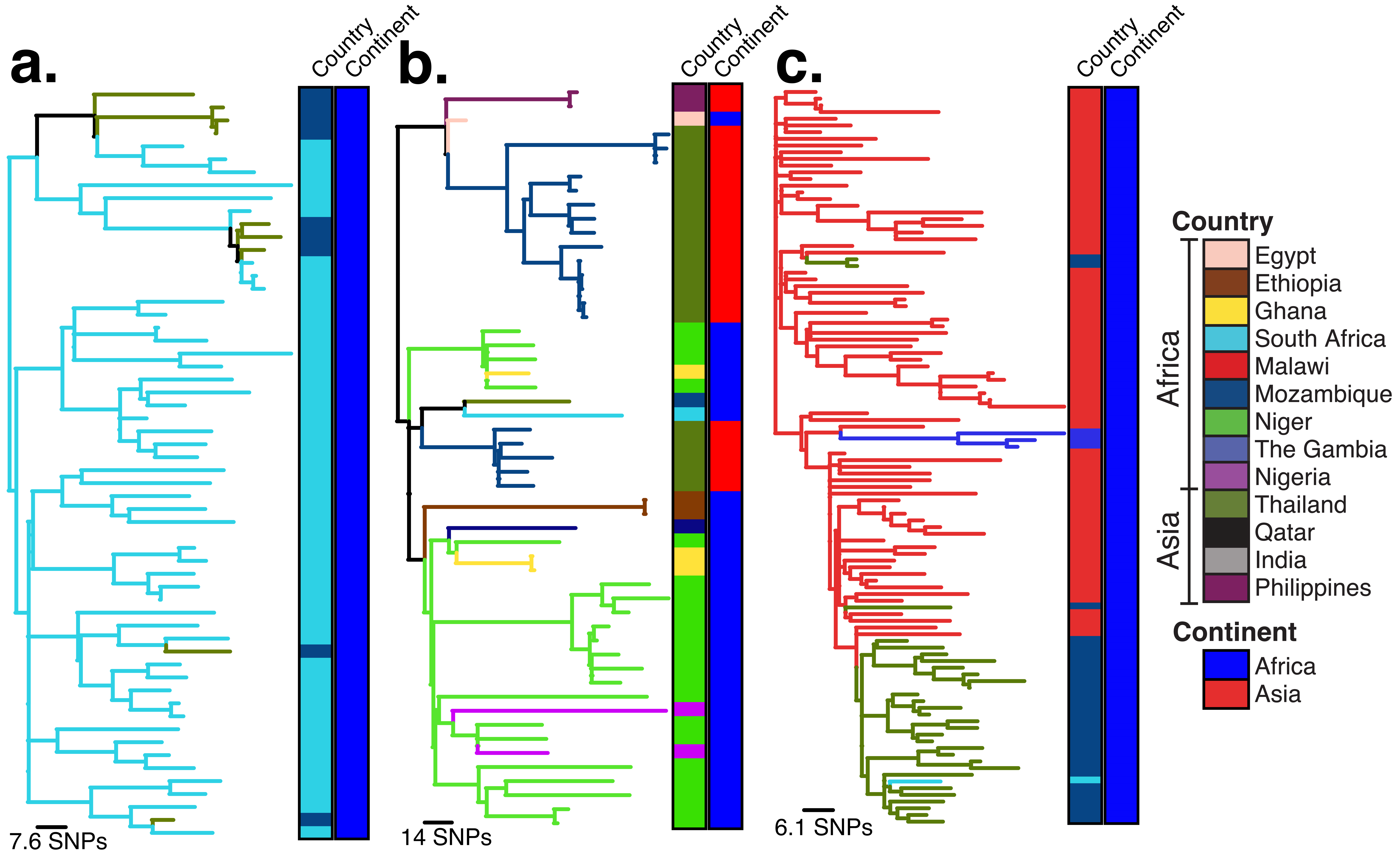
a.
b.
c.
Country
Continent
7.6 SNPs
14 SNPs
6.1 SNPs
Country
Africa
Asia
Egypt
Ethiopia
Ghana
South Africa
Malawi
Mozambique
Niger
The Gambia
Nigeria
Thailand
Qatar
India
Philippines
Continent
Africa
Asia
